# Synthesis and physical properties of tunable aryl alkyl ionic liquids based on 1-aryl-4,5-dimethylimidazolium cations

**DOI:** 10.3762/bjoc.20.110

**Published:** 2024-05-31

**Authors:** Stefan Fritsch, Thomas Strassner

**Affiliations:** 1 Physikalische Organische Chemie, Technische Universität Dresden, 01062 Dresden, Germany

**Keywords:** conductivity, electrochemical window, ionic liquids, tunable aryl alkyl ionic liquid, viscosity

## Abstract

We present a new class of tunable aryl alkyl ionic liquids (TAAILs) based on 1-aryl-4,5-dimethylimidazolium cations with electron-withdrawing and -donating substituents in different positions of the phenyl ring and the bis(trifluoromethylsulfonyl)imide (NTf_2_) anion. We investigated the effect of additional methyl groups in the backbone of the imidazolium core on the physical properties regarding viscosity, conductivity and electrochemical window. With an electrochemical window of up to 6.3 V, which is unprecedented for TAAILs with an NTf_2_ anion, this new class of TAAILs demonstrates the opportunities that arise from modifications in the backbone of the imidazolium cation.

## Introduction

Ionic liquids (ILs) are a special class of solvents generally defined as salts with melting points below 100 °C [1]. Due to their unique properties, e.g., their high thermal stability and their negligible vapor pressure [2–3], ILs have found widespread use in different fields of chemistry like synthesis [4–9], catalysis [10–14] and materials science [15–23]. ILs generally consist of an organic cation [24], such as the imidazolium or ammonium ion and an inorganic anion like a halide anion or weakly coordinating anions like bis(trifluoromethylsulfonyl)imide [NTf_2_]^−^ [25]. In addition, dicationic salts or anions containing metal complexes have been described [26–27]. Due to the numerous combinations of different anions and cations, ILs can also be described as designer solvents [28]. We introduced a new class of ionic liquids based on the 1-aryl-3-alkylimidazolium cation in 2009, the tunable aryl alkyl ionic liquids (TAAILs) [29]. This class of ionic liquids allows the modification of physical and chemical properties by variation of the functional groups present at the aryl ring of the ionic liquid in addition to the possibility to introduce alkyl chains with varying length [30]. TAAILs have already been successfully used for the synthesis of nanoparticles and as solvents in catalysis [31–32]. Recently we have described the synthesis and physical properties of TAAILs which have been blocked at the C2 position [33]. The use of a substituent at the C2 position was found to have a strong influence on the properties of these ionic liquids due to changes in the hydrogen-bonding network. Here, we investigate the properties of TAAILs based on the 1-aryl-4,5-dimethylimidazolium cation. It is well known that the hydrogen atom at the C2 carbon atom of the imidazole core is more acidic as those in the C4/C5-position, where the methyl groups are expected to also have a significant effect on the molecular interactions. Platinum complexes using the 4,5-dimethylimidazole motif as a ligand were already reported by our group [34] and piqued our interest on the performance of TAAILs based on this motif. Since the introduction of methyl groups in the backbone of the imidazolium core increases the stability and basicity of the corresponding imidazolium derivatives [35–37], we wanted to investigate the impact on the properties of ILs. The negative impact on viscosity and conductivity due to the higher mass should be comparatively low. We investigated different substituents with electron-donating as well as electron-withdrawing properties at the aryl ring and the difference between the *ortho* and *para* position of the substituent on the properties of the IL. For the [NTf_2_]^−^ salts of the TAAILs, the viscosity, conductivity and the electrochemical window were determined.

## Results and Discussion

**Scheme 1:**
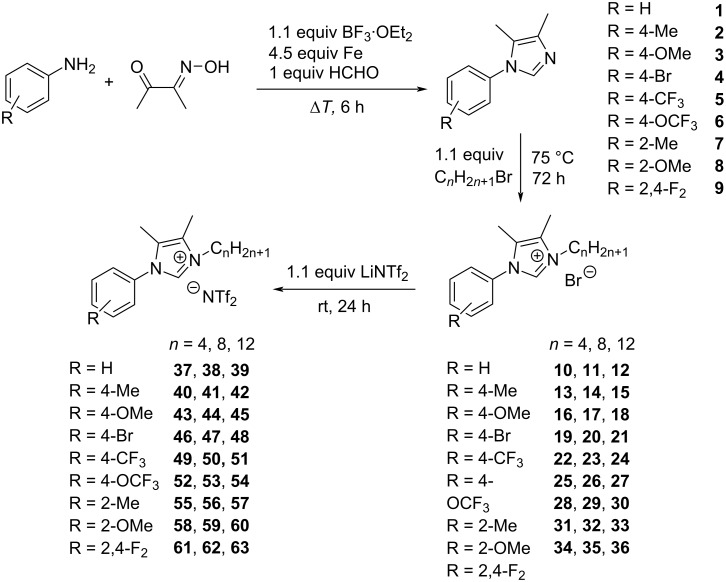
Overview of the synthesis of compounds **1**–**63**.

In this work we focused on a synthetic strategy with cost efficient starting materials and a good scalability [38–42]. The substituted imidazoles **1**–**9** were synthesized using a condensation reaction between the corresponding aniline and diacetyl monoxime (Scheme 1), since the condensation using diacetyl was not successful for electron-poor aniline derivatives.

This reaction leads to the formation of N-oxides, which were then reduced using iron powder. The imidazoles were synthesized in a one-pot procedure on a scale of up to 400 mmol. Quaternization of the imidazoles using bromoalkanes with different chain lengths provided the bromide salts **10**–**36**. Conversion to the NTf_2_ ionic liquids **37**–**63** was achieved via anion exchange using LiNTf_2_.

The synthesized TAAILs with the NTf_2_ anion are all ionic liquids with a melting point well below 100 °C and the majority of these ILs can also be described as room temperature ionic liquids (RTIL), with a melting point below room temperature. The short-term thermal stability was investigated using ramped temperature analysis. The thermal decomposition temperatures (Table 1) for the ILs with the NTf_2_ anion are in the range from 266 °C (IL **59**) to 409 °C (IL **37**). In general, most ionic liquids with a butyl chain show higher decomposition temperatures than their counterparts with octyl or dodecyl chain. An increase in alkyl chain length from octyl to dodecyl, however, does not necessarily lead to a lower thermal stability.

**Table 1:** Thermal decomposition point at 5% mass loss in °C for NTf_2_^−^ TAAILs **37**–**63** with different aryl substituents (R) and alkyl chain lengths (*n*).

R	*n* = 4	*n* = 8	*n* = 12

H	409	298	322
4-Me	396	349	316
4-OMe	300	306	363
4-Br	378	397	350
4-CF_3_	393	313	378
4-OCF_3_	383	370	307
2-Me	323	322	339
2-OMe	372	266	343
2,4-F_2_	398	364	291

With decomposition temperatures of up to 409 °C (IL **37**), the 1-aryl-4,5-dimethyl TAAILs can be described as ILs with a comparatively high thermal stability [43–45].

**Figure 1:**
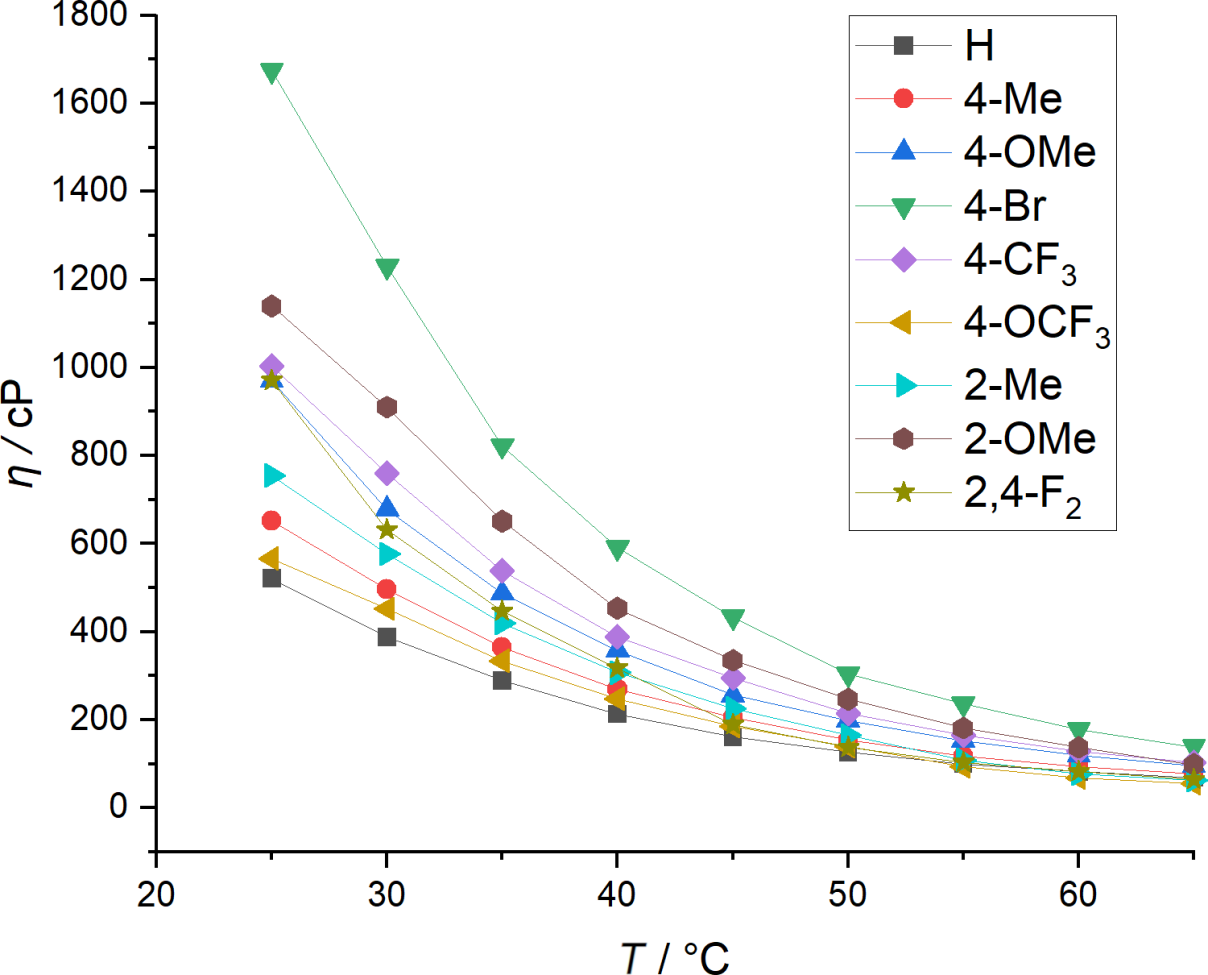
Temperature-dependent viscosity measurement of NTf_2_^−^ TAAILs **37**–**63** with a butyl chain and different aryl substitution R.

The viscosities of TAAILs with different aryl substitutions and a butyl chain are shown in Figure 1.

At 25 °C the highest measured viscosity was 1675 cP for the TAAIL with the 4-Br substitution and the lowest viscosity (521 cP) for the TAAIL with the unsubstituted aryl moiety. The viscosities of the 4-CF_3_ and 2,4-F_2_ TAAILs with butyl chains are very similar, since the viscosity is influenced by π–π and dispersion effects facilitated by the electron-withdrawing effect of the fluorine atoms [46–47]. The 4-OCF_3_ substituted TAAIL with a butyl chain, however, displayed a much lower viscosity, indicating that additional steric effects due to the 4-OCF_3_ substituent play a role for the viscosity of the TAAIL [48–49]. The viscosities of the NTf_2_ ionic liquids **37**–**63** at 25 °C are given in Table 2. In general, with increasing alkyl chain lengths we observe an increasing viscosity of the TAAILs, which can be explained by the increase in molar mass and stronger van-der-Waals interactions. The only exceptions are the methoxy and 2,4-difluoro substituted TAAILs, demonstrating that the effects of the aryl substitution can have a stronger influence on the viscosity than the alkyl chain length.

**Table 2:** Viscosity in centipoise (cP) of NTf_2_^−^ TAAILs **37**–**63** with different aryl substituents (R) and alkyl chain lengths (*n*), measured at 25 °C.

R	*n* = 4	*n* = 8	*n* = 12

H	521	570	683
4-Me	652	750	762
4-OMe	970	900	1020
4-Br	1675	1720	solid
4-CF_3_	1003	1092	solid
4-OCF_3_	566	638	solid
2-Me	755	835	919
2-OMe	1140	1134	1268
2,4-F_2_	971	891	1016

**Figure 2:**
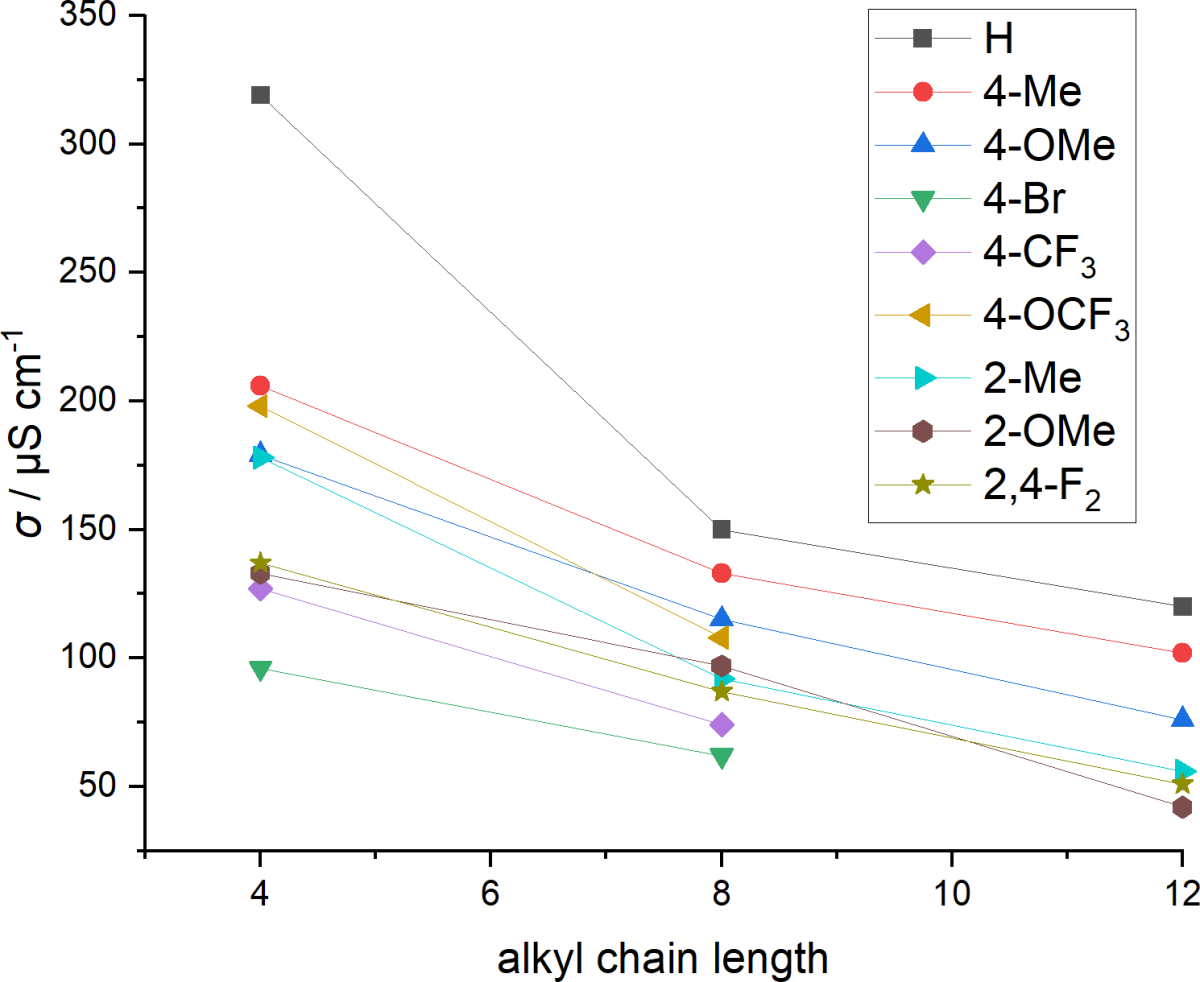
Conductivity of NTf_2_^−^ TAAILs **37**–**63** measured at 25 °C. Compounds **48**, **51**, and **54** are excluded because of their melting point.

The conductivity of the unsubstituted TAAIL **37** with 319 μS cm^−1^ is the highest among the investigated 4,5-dimethylimidazolium based TAAILs (Figure 2, Table 3). This is supported by the corresponding observation that TAAIL **37** also shows the lowest viscosity. As a result of their high viscosity, the 4-Br substituted TAAILs **46** and **47** display the lowest conductivities (96 and 62 μS cm^−1^, respectively).

**Figure 3:**
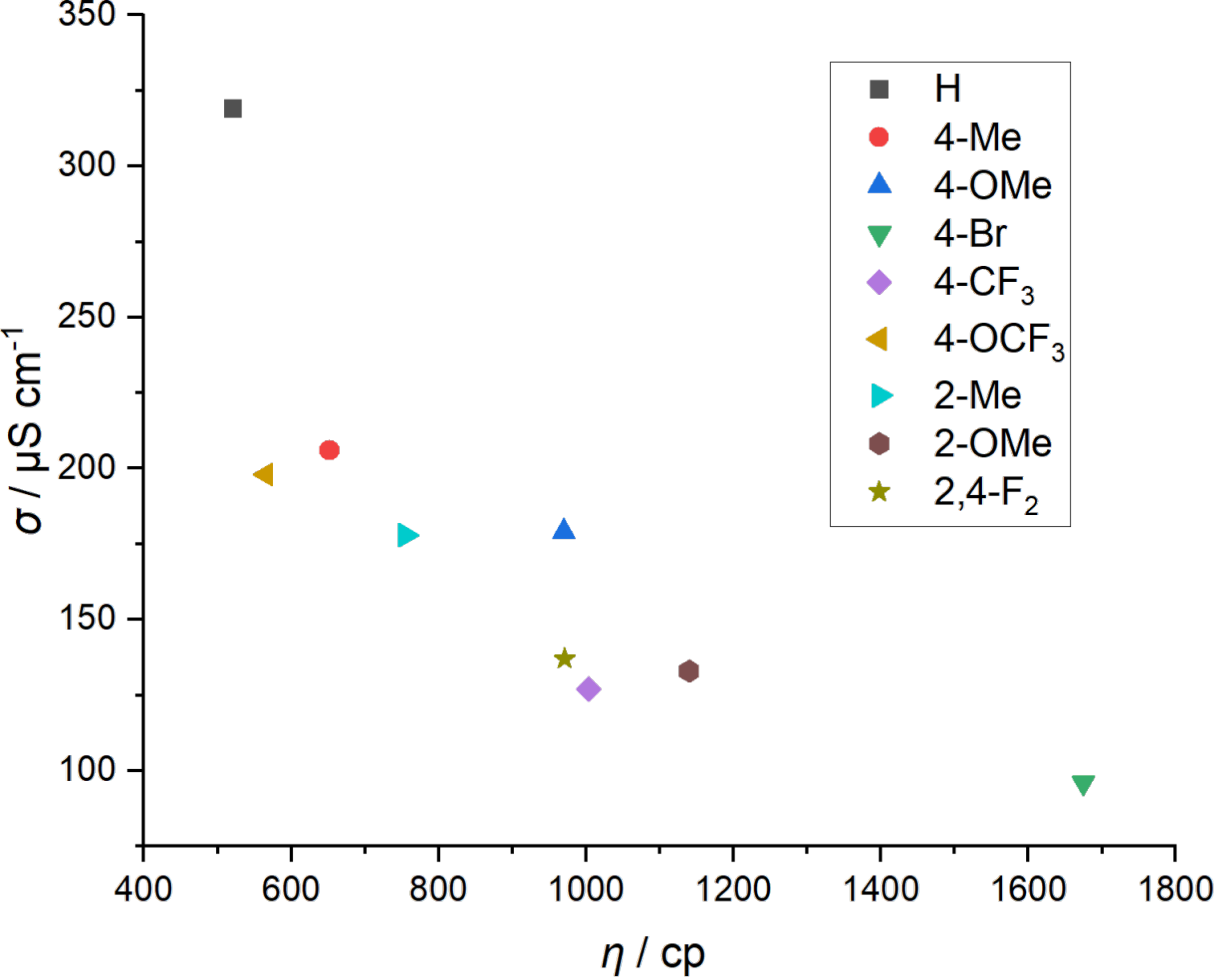
Conductivity of NTf_2_^−^ TAAILs with a butyl chain plotted against their viscosity at 25 °C.

To visualize the correlation between conductivity and viscosity, the conductivity is plotted against the viscosity in Figure 3. The 4-OCF_3_ substituted TAAIL **52** shows a similar viscosity to TAAIL **37**, the conductivity, however, is much lower (198 μS cm^−1^), demonstrating the influence of the perfluorinated 4-OCF_3_ group on the conductivity.

Changing the position of the methyl and methoxy substituent from *ortho* to *para* leads to an increase in conductivity. Overall, longer alkyl chains lead to a decreasing conductivity, with the difference between butyl and octyl being more drastic than the difference between octyl and dodecyl.

**Figure 4:**
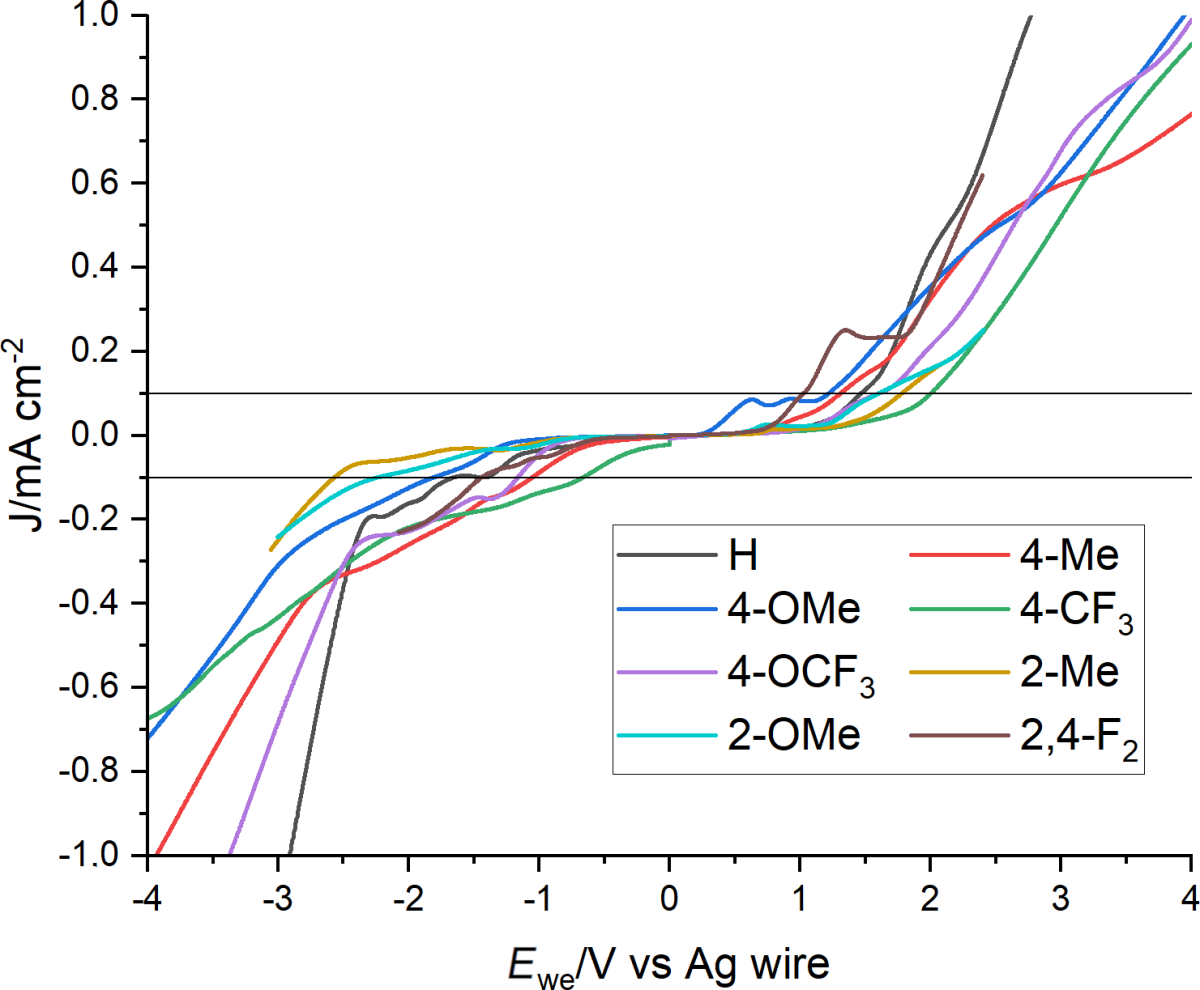
Linear sweep voltammetry of NTf_2_^−^ TAAILs with a butyl chain and different aryl substituents R. Black lines indicate cutoff. Scan rate: 50 mV s^−1^. Scan start at 0 V.

To determine the electrochemical stability of the TAAILs, their electrochemical properties were investigated using linear sweep voltammetry (LSV, Figure 4). The setup consisted of a glassy carbon working electrode, a platinum wire counter electrode and a silver wire as reference electrode. The cutoff at which the cathodic limit *E*_red_ and the anodic limit *E*_ox_ were determined was −0.1 and 0.1 mA cm^−2^. The electro-chemical window *E*_EW_ is determined from the difference between *E*_red_ and *E*_ox_.

The anodic limit *E*_ox_ is mainly influenced by the anion of the ionic liquid, resulting in similar values for *E*_ox_ for the NTf_2_-based TAAILs. The cathodic limit *E*_red_ depends on the cation and at what voltage the imidazolium cation is reduced. The substitution on the aryl ring has a strong influence on the reductive potential (*E*_red_), ranging from −0.7 V for the 4-CF_3_ substituted TAAIL **49** to −2.6 V for the 2-Me substituted TAAIL **55** with a butyl chain. The change from *ortho* to *para* substitution led to a decrease of the cathodic limit in case of the methyl and methoxy-substituted TAAILs. The majority of TAAILs with a butyl chain have a cathodic limit between −1.0 and −2.0 V. The electrochemical window for the TAAILs with a butyl chain ranges from 2.4 V to 4.4 V. In general, an increase of the alkyl chain length leads to a wider electrochemical window. The results of the electrochemical measurements of all TAAILs can be found in Table 3, with the largest electrochemical window being 6.3 V for TAAIL **57**. This is the largest electrochemical window reported for TAAILs with NTf_2_ anion so far [50–53]. The size of the electrochemical window, however, depends heavily on the measurement conditions, preventing a general comparison with previously reported electrochemical windows [54–55]. The plots of all LSV measurements can be found in Supporting Information File 1 (Figures S1–S8).

**Table 3:** Physicochemical data of NTf_2_^−^ TAAILs **37**–**63**. R: aryl substituent, *n*: alkyl chain length. Specific conductivity σ was measured at 25 °C. Anodic and cathodic cut-off limit: 0.1 mA cm^−2^. **48**, **51** and **54** are excluded due to their melting point.

Nr	R	*n*	σ [μS cm^−1^]	*E*_red_ [V]	*E*_ox_ [V]	*E*_EW_ [V]

**37**	H	4	319	−1.7	1.5	3.2
**38**	H	8	150	−2.0	1.6	3.6
**39**	H	12	120	−2.6	2.2	4.8
**40**	4-Me	4	206	−1.1	1.3	2.4
**41**	4-Me	8	133	−2.0	1.5	3.5
**42**	4-Me	12	102	−2.5	2.0	4.5
**43**	4-OMe	4	179	−1.8	1.2	3.0
**44**	4-OMe	8	115	−2.2	1.5	3.7
**45**	4-OMe	12	76	−3.0	1.7	4.7
**46**	4-Br	4	96	–^a^	–^a^	–^a^
**47**	4-Br	8	62	–^a^	–^a^	–^a^
**49**	4-CF_3_	4	127	−0.7	2.0	2.7
**50**	4-CF_3_	8	74	−2.9	2.3	5.2
**52**	4-OCF_3_	4	198	−1.2	1.6	2.8
**53**	4-OCF_3_	8	108	−2.9	2.6	5.5
**55**	2-Me	4	178	−2.6	1.8	4.4
**56**	2-Me	8	92	−2.7	2.2	4.9
**57**	2-Me	12	56	−3.4	2.9	6.3
**58**	2-OMe	4	133	−2.2	1.6	3.8
**59**	2-OMe	8	97	−2.7	1.9	4.6
**60**	2-OMe	12	42	−2.8	2.2	5.0
**61**	2,4-F_2_	4	137	−1.4	1.0	2.4
**62**	2,4-F_2_	8	87	−1.9	1.9	3.8
**63**	2,4-F_2_	12	51	−2.4	2.1	4.5

^a^No results due to decomposition.

**Figure 5:**
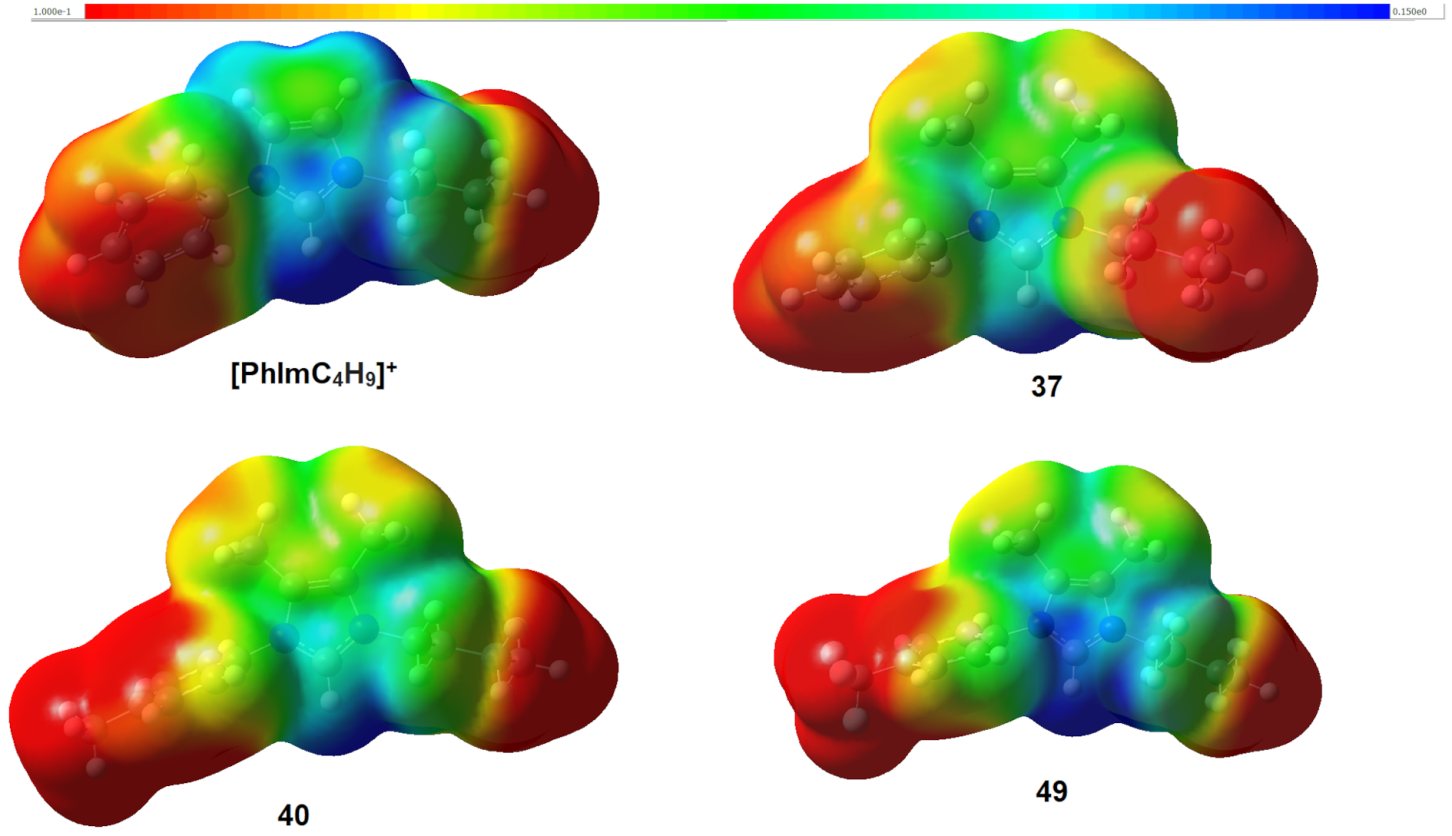
Structures of the imidazolium cations obtained by DFT calculations (B3LYP/6-311++G (d,p)). The electrostatic potentials are indicated by color (blue: positive; red: negative).

Figure 5 shows the molecular electrostatic potential (MEP) of the unsubstituted TAAIL cation [PhImC_4_H_9_]^+^, the unsubstituted 4,5-dimethyl-1-phenyl cation of TAAIL **37** and the cations of TAAILs **40** (4-CH_3_) and **49** (4-CF_3_).

The structures of the cations were optimized with Gaussian 16 [56], using the hybrid functional Becke3LYP [57–60] with the split valence triple-ζ basis set 6-311++G(d,p) [61–63] and D3 dispersion correction with the Becke–Johnson damping scheme [64–65]. All optimized structures were confirmed to be true minima by the absence of negative frequencies after harmonic vibrational modes calculation. The MEP was visualized with GaussView6. Comparing the 4,5-dimethylimidazolium cations **37**, **40** and **49** with the previously reported TAAIL cation [PhImC_4_H_9_]^+^ shows that the methyl groups in the backbone of the imidazolium cation lead to a different electrostatic potential (Figure 4). Comparison of the 4-Me substituted imidazolium cation **40** with the 4-CF_3_ substituted cation **49** shows the influence of the electronegative fluorine atoms on the electrostatic potential of the cation.

## Conclusion

Based on the synthesis of nine different 1-aryl-4,5-dimethyl substituted imidazoles we report a new class of ionic liquids with electron-donating and -withdrawing substituents, three different alkyl chain lengths and bromide as well as NTf_2_ anions. The physicochemical properties (thermal properties, viscosity, conductivity and electrochemical window) of the RTILs were investigated. The two methyl groups in the backbone of the imidazolium core lead to a slightly higher viscosity compared to the unsubstituted congeners. It is also influenced by the type of substitution (electron-withdrawing or -donating) at the aryl ring as well as the alkyl chain length. By introducing the methyl groups in the backbone of the imidazolium cation we were able to enlarge the electrochemical window up to 6.3 V, which is currently the largest window for a bis(trifluoromethylsulfonyl)imide tunable aryl alkyl ionic liquid.

## Supporting Information

File 1 Experimental procedures and characterization data.

## Data Availability

All data that supports the findings of this study is available in the published article and/or the supporting information to this article.
