# Supplementary material for: Synthesis and physical properties of tunable aryl alkyl ionic liquids based on 1-aryl-4,5-dimethylimidazolium cations
Source: Beilstein J Org Chem. 2024 May 31;20:1278–85. doi: 10.3762/bjoc.20.110 (PMC11181176; doi:10.3762/bjoc.20.110)
Supplement: File 1 — Experimental procedures and characterization data. [file Beilstein_J_Org_Chem-20-1278-s001.pdf]

BEILSTEIN JOURNAL OF ORGANIC CHEMISTRY

Supporting Information

for

Synthesis and physical properties of tunable aryl alkyl ionic liquids based on 1-aryl-4,5-dimethylimidazolium cations

Stefan Fritsch and Thomas Strassner

Beilstein J. Org. Chem. **2024,** *20,* 1278–1285. doi:10.3762/bjoc.20.110

Experimental procedures and characterization data

License and Terms: This is a supporting information file under the terms of the Creative Commons Attribution License (https://creativecommons.org/licenses/by/4.0). Please note that the reuse, redistribution and reproduction in particular requires that the author(s) and source are credited and that individual graphics may be subject to special legal provisions.
The license is subject to the *Beilstein Journal of Organic Chemistry* terms and conditions: (https://www.beilstein-journals.org/bjoc/terms)

Contents

Section S1 Synthesis of imidazole derivatives S2–S7

Section S2 Synthesis of imidazolium bromides S8–S21

Section S3 Synthesis of NTf_2-ionic liquids S22–S35

Section S4 Electrochemical measurements S36–S40

Section S5 Viscosity measurements S40–S44

Section S6 Thermogravimetric analysis S45–S58

Section S7 NMR-Spectra S59–S104

Section S8 Cartesian coordinates of optimized structures S105–S109

Section S9 References S110

S1 Synthesis of imidazole derivatives

General informations

NMR spectroscopic data were recorded at room temperature with a Bruker AV II 300 spectrometer and a Bruker AV III 600 spectrometer. Chemical shifts δ are referenced to solvent signals [^{1}H NMR: $CDCl_3$ (7.26 ppm); ^{13}C NMR: $CDCl_3$ (77.16 ppm)]. ^{19}F spectra were referenced externally to trifluoromethylbenzene in $CDCl_3$. Chemical shifts are given in ppm, coupling constants *J* are given in Hertz (Hz). Signal patterns are indicated as s, singlet; d, doublet; dd, double doublet; t, triplet; q, quartet; quin, quintet; sxt, sextet; m, multiplet. Before any physicochemical measurements were conducted, all NTf_2 ionic liquids were heated to 120 °C under vigorous stirring and a vacuum of 0.001 mbar was applied. The ionic liquid was stirred at this temperature for 15 minutes and was allowed to cool to room temperature under vacuum with continued stirring. The ionic liquids were flushed with and stored under argon. Elemental analyses were performed with a Thermo Fisher Scientific FlashSmart elemental analyzer. Viscosities were determined using a Brookfield DV2T viscosimeter with a CPA-52 rotating disc. The viscosimeter was calibrated using Brookfield “CAP2L” viscosity standard with 177 cp at 25 °C. The viscosity accuracy is ±1%. The measurements were carried out from 25 °C to 65 °C in 5 °C steps. Conductivities were measured at 25°C with a Mettler Toledo cond probe InLab 752–6 mm connected to a Mettler Toledo Seven Easy conductivity meter with an uncertainty of ±4 µS/cm. The conductivity meter was calibrated using conductivity standard 1413 µS/cm from Hanna Instruments. Melting points were determined by using a Wagner and Munz PolyTherm A system and are given uncorrected. Linear sweep voltammetry (LSV) was performed in pure ionic liquid using a BioLogic SP-150 potentiostat with a glassy carbon working electrode (diameter 3 mm), a Pt-wire counter electrode and an Ag-wire as a pseudo reference electrode. The measurements were conducted with a sweep rate of 50 mV/s. The electrochemical window was determined with a cut-off current density of 0.1 mA/cm^2. Thermogravimetric analyses were performed using a Netzsch TG209 F1 Libra with Al_2O_3 crucibles. The measurements were conducted under an argon atmosphere with a heating rate of 10 °C/min (25–500 °C). The decomposition temperature was set at 5% mass loss. The TGA sample chamber containing the sample was purged and flushed with argon three times before starting the measurement.

General procedure for the synthesis of 4,5-dimethyl-1-phenylimidazoles

To a solution of butane-2,3-dione monoxime (400 mmol, 1.0 equiv) in 400 ml acetic acid, $BF_3{\cdot}OEt_2$ (440 mmol, 1.1 equiv), formaldehyde (400 mmol, 1.0 equiv) and the corresponding aniline (400 mmol, 1.0 equiv) were added, and the solution was stirred at 50 °C for 4 hours. After 4 hours, iron powder (1.80 mol, 4.5 equiv) was added and the solution was refluxed for 2 hours. Afterwards, the reaction mixture was poured into water and the iron residue was removed via filtration. The filtrate was extracted with chloroform and the organic phase was washed with 10% aqueous potassium carbonate solution until no further gas evolution was observed. The organic layer was washed with distilled water, dried over sodium sulfate and filtered. The chloroform was removed under reduced pressure. The crude product was purified via vacuum distillation.

4,5-Dimethyl-1-phenyl-1*H*-imidazole (1)[1]

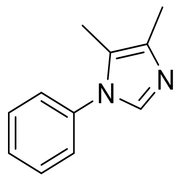

Following the general procedure using 200 mmol of aniline, the product was obtained with a yield of 50% (17.2 g, 100 mmol) as a yellow oil.

^{1}H NMR: (300MHz, $CDCl_3$) δ 7.39 - 7.56 (m, 4 H), 7.22 - 7.29 (m, 2 H), 2.24 (s, 3 H), 2.09 (s, 3 H).

^{13}C NMR: (75 MHz, $CDCl_3$) δ 136.8, 134.9, 134.0, 129.5, 128.2, 125.5, 123.1, 12.6, 9.1.

Melting point: liquid at room temperature.

4,5-Dimethyl-1-(*p*-tolyl)-1*H*-imidazole (2)[2]

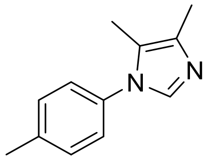

Following the general procedure using 200 mmol of aniline, the product was obtained with a yield of 43% (15.9 g, 85 mmol) as a light yellow oil.

^{1}H NMR: (300MHz, $CDCl_3$) δ 7.47 (s, 1 H), 7.22 - 7.30 (m, 2 H), 7.10 - 7.16 (m, 2 H), 2.40 (s, 3 H), 2.22 (s, 3 H), 2.07 (s, 3 H).

^{13}C NMR: (75 MHz, $CDCl_3$) δ 138.2, 134.9, 134.2, 133.8, 130.0, 125.3, 123.1, 21.0, 12.6, 9.0.

Melting point: liquid at room temperature.

1-(4-Methoxyphenyl)-4,5-dimethyl-1*H*-imidazole (3)[3]

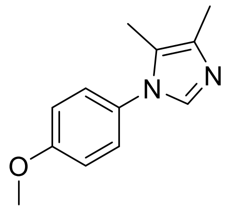

Following the general procedure using 400 mmol of aniline, the product was obtained with a yield of 40% (32 g, 158 mmol) as a light yellow oil that solidified upon standing.

^{1}H NMR: (300MHz, $CDCl_3$) δ 7.47 (s, 1 H), 7.13 - 7.21 (m, 2 H), 6.92 - 7.01 (m, 2 H), 3.85 (s, 3 H), 2.22 (s, 3 H), 2.05 (s, 3 H).

^{13}C NMR: (75 MHz, $CDCl_3$) δ 159.3, 135.0, 133.5, 129.6, 126.9, 123.4, 114.5, 55.5, 12.6, 8.9.

Melting point: 51°C.

1-(4-Bromophenyl)-4,5-dimethyl-1*H*-imidazole (4)

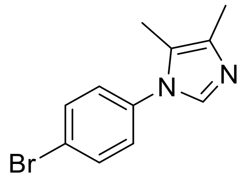

Following the general procedure using 400 mmol of aniline, the product was obtained with a yield of 53% (53.5 g, 213 mmol) as a light yellow oil that solidified upon standing.

^{1}H NMR: (300MHz, $CDCl_3$) δ 7.62 (m, 2 H), 7.56 (s, 1 H), 7.15 (m, 2 H), 2.24 (s, 3 H), 2.09 (s, 3 H).

^{13}C NMR: (75 MHz, $CDCl_3$) δ 135.6, 134.6, 134.1, 132.8, 127.0, 123.0, 122.2, 12.5, 9.0.

Melting point: 64°C.

4,5-Dimethyl-1-(4-(trifluoromethyl)phenyl)-1*H*-imidazole (5)

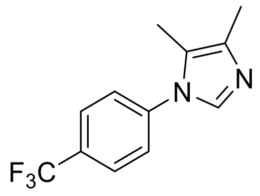

Following the general procedure using 400 mmol of aniline, the product was obtained with a yield of 44% (42.2 g, 176 mmol) as a clear oil that solidified upon standing.

^{1}H NMR: (300MHz, $CDCl_3$) δ 7.77 (m, 2 H), 7.66 (d, *J* = 8.80 Hz, 1 H), 7.42 (m, 2 H), 2.26 (s, 3 H), 2.14 (s, 3 H).

^{13}C NMR: (75 MHz, $CDCl_3$) δ 139.5, 134.6, 130.7, 126.9 (q, *J* = 3.6 Hz), 125.6, 123.0, 121.8, 12.4, 9.2.

^{19}F NMR: (282 MHz, $CDCl_3$) δ -63.2.

Melting point: 74°C.

4,5-Dimethyl-1-(4-(trifluoromethoxy)phenyl)-1*H*-imidazole (6)

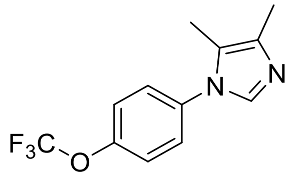

Following the general procedure using 400 mmol of aniline, the product was obtained with a yield of 56% (57.8 g, 225 mmol) as a light yellow oil.

^{1}H NMR: (300MHz, $CDCl_3$) δ 7.56 (s, 1 H), 7.24 - 7.38 (m, 4 H), 2.24 (s, 3 H), 2.10 (s, 3 H).

^{13}C NMR: (75 MHz, $CDCl_3$) δ 148.7, 134.8, 127.0, 125.5, 123.1, 122.0, 118.6, 12.5, 9.0.

^{19}F NMR: (282 MHz, $CDCl_3$) δ -58.6.

Melting point: liquid at room temperature.

4,5-Dimethyl-1-(*o*-tolyl)-1*H*-imidazole (7)[4]

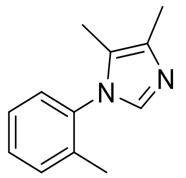

Following the general procedure using 400 mmol of aniline, the product was obtained with a yield of 41% (30.3 g, 163 mmol) as a light yellow oil.

^{1}H NMR: (300MHz, $CDCl_3$) δ 7.23 - 7.38 (m, 4 H), 7.12 (d, *J* = 7.7 Hz, 1 H), 2.22 (s, 3 H), 2.02 (s, 3 H), 1.89 (s, 3 H).

^{13}C NMR: (75 MHz, $CDCl_3$) δ 135.6, 134.8, 133.3, 130.9, 129.1, 127.8, 126.7, 123.4, 17.2, 12.6, 8.4.

Melting point: liquid at room temperature.

1-(2-Methoxyphenyl)-4,5-dimethyl-1*H*-imidazole (8)[1]

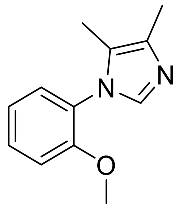

Following the general procedure using 400 mmol of aniline, the product was obtained with a yield of 28% (22.9 g, 113 mmol) as a light yellow oil that solidified upon standing.

^{1}H NMR: (300MHz, $CDCl_3$) δ 7.43 (s, 1 H), 7.37 - 7.42 (m, 1 H), 7.12 - 7.19 (m, 1 H), 7.00 - 7.06 (m, 2 H), 3.78 (s, 3 H), 2.23 (s, 3 H), 1.96 (s, 3 H).

^{13}C NMR: (75 MHz, $CDCl_3$) δ 154.6, 135.4, 132.6, 130.0, 128.4, 125.4, 124.2, 120.7, 112.0, 55.6, 12.6, 8.5.

Melting point: 75°C.

1-(2,4-Difluorophenyl)-4,5-dimethyl-1*H*-imidazole (9)

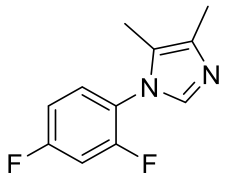

Following the general procedure using 400 mmol of aniline, the product was obtained with a yield of 52% (43.0 g, 207 mmol) as a light yellow oil that solidified upon standing.

^{1}H NMR: (300MHz, $CDCl_3$) δ 7.56 (m, 1 H), 7.21 - 7.30 (m, 1 H), 6.94 - 7.04 (m, 2 H), 2.25 (s, 3 H), 2.01 (s, 3 H).

^{13}C NMR: (75 MHz, $CDCl_3$) δ 164.2 (dd, *J* = 262.9, 10.7 Hz), 159.1 (dd, *J* = 267.0, 12.5 Hz), 135.3, 133.8, 129.6 (dd, *J* = 11.3, 1.2 Hz), 123.9, 112.2 (dd, *J* = 26.2, 4.2 Hz), 105.6 (dd, *J* = 50.1, 23.8 Hz), 12.5, 8.3.

^{19}F NMR: (282 MHz, $CDCl_3$) δ -106.9, -116.6.

Melting point: 45 °C.

S2 Synthesis of imidazolium bromides

General procedure for the synthesis of the imidazolium bromides

In an ACE pressure tube the corresponding aryl-imidazole (34 mmol) is dissolved in 17 mL of THF and 1.1 equiv of the corresponding alkyl bromide is added. The solution is stirred at 75 °C for 72 h. Afterwards the reaction mixture is allowed to cool to room temperature and the solvent was removed in vacuo. The crude product is then washed with diethyl ether and isohexane.

3-Butyl-4,5-dimethyl-1-phenyl-1*H*-imidazol-3-ium bromide (10)

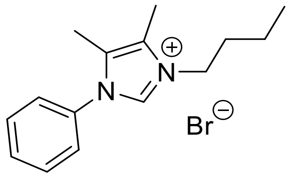

Following the general procedure, 34 mmol of imidazole **1** were alkylated using 1-bromobutane. The product was obtained as a brown solid (9.6 g, 31 mmol, 92%).

^{1}H NMR: (300MHz, $CDCl_3$) δ 10.09 (s, 1 H), 7.37 - 7.56 (m, 5 H), 4.43 (t, *J* = 7.6 Hz, 2 H), 2.30 (s, 3 H), 2.13 (s, 3 H), 1.83 (quin, *J* = 7.6 Hz, 2 H), 1.40 (sxt, *J* = 7.5 Hz, 2 H), 0.92 (t, *J* = 7.3 Hz, 3 H).

^{13}C NMR: (75 MHz, $CDCl_3$) δ 135.6, 133.0, 130.5, 130.1, 126.8, 126.7, 125.7, 47.3, 31.9, 19.4, 13.4, 9.2, 8.6.

Melting point: 55 °C.

4,5-Dimethyl-3-octyl-1-phenyl-1*H*-imidazol-3-ium bromide (11)

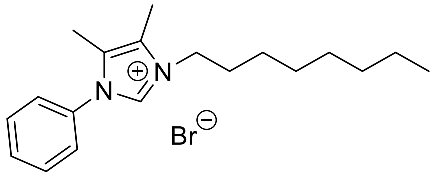

Following the general procedure, 32 mmol of imidazole **1** were alkylated using 1-bromooctane. The product was obtained as a brown solidified melt (11.1 g, 30 mmol, 96%).

^{1}H NMR: (300MHz, $CDCl_3$) δ 10.09 (s, 1 H), 7.43 - 7.58 (m, 5 H), 4.35 - 4.49 (m, 2 H), 2.30 (s, 3 H), 2.14 (s, 3 H), 1.79 - 1.92 (m, 2 H), 1.14 - 1.36 (m, 10 H), 0.75 - 0.86 (m, 3 H).

^{13}C NMR: (75 MHz, $CDCl_3$) δ 135.6, 133.1, 130.5, 130.2, 126.8, 126.7, 125.7, 47.5, 31.6, 30.1, 28.9, 26.2, 22.4, 13.9, 9.2, 8.7.

Melting point: solidified melt.

3-Dodecyl-4,5-dimethyl-1-phenyl-1*H*-imidazol-3-ium bromide (12)

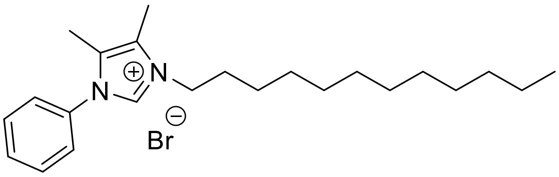

Following the general procedure, 30 mmol of imidazole **1** were alkylated using 1-bromo-dodecane. The product was obtained as a brown solidified melt (11.7 g, 28 mmol, 93%).

^{1}H NMR: (300MHz, $CDCl_3$) δ 10.13 (s, 1 H), 7.45 - 7.60 (m, 5 H), 4.44 (t, *J* = 7.6 Hz, 2 H), 2.31 (s, 3 H), 2.15 (s, 3 H), 1.80 - 1.94 (m, 2 H), 1.13 - 1.31 (m, 18 H), 0.79 - 0.88 (m, 3 H).

^{13}C NMR: (75 MHz, $CDCl_3$) δ 135.8, 133.1, 130.6, 130.2, 126.7, 126.6, 125.7, 47.6, 31.7, 30.2, 29.5, 29.4, 29.4, 29.3, 29.2, 29.0, 26.3, 22.5, 14.0, 9.2, 8.7.

Melting point: solidified melt.

3-Butyl-4,5-dimethyl-1-(*p*-tolyl)-1*H*-imidazol-3-ium bromide (13)

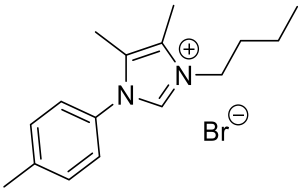

Following the general procedure, 31 mmol of imidazole **2** were alkylated using 1-bromobutane. The product was obtained as a brown oil (9.6 g, 30 mmol, 96%).

^{1}H NMR: (300MHz, $CDCl_3$) δ 10.06 (s, 1 H), 7.21 - 7.38 (m, 4 H), 4.32 - 4.48 (m, 2 H), 2.36 (s,3 H), 2.29 (s, 3 H), 2.11 (s, 3 H), 1.73 - 1.90 (m, 2 H), 1.39 (m, 2 H), 0.84 - 0.96 (m, 3 H).

^{13}C NMR: (75 MHz, $CDCl_3$) δ 141.2, 135.8, 130.9, 130.8, 121.8, 126.8, 125.7, 47.5, 32.3, 21.3, 19.7, 13.7, 9.4, 8.9.

Melting point: liquid at room temperature.

4,5-Dimethyl-3-octyl-1-(*p*-tolyl)-1*H*-imidazol-3-ium bromide (14)

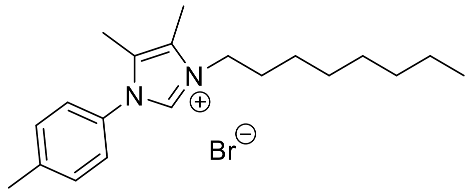

Following the general procedure, 26 mmol of imidazole **2** were alkylated using 1-bromooctane. The product was obtained as a brown solidified melt (9.5 g, 25 mmol, 95%).

^{1}H NMR: (300MHz, $CDCl_3$) δ 10.06 (s, 1 H), 7.31 (m, 4 H), 4.40 - 4.47 (m, 2 H), 2.38 (s, 3 H), 2.30 (s, 3 H), 2.13 (s, 3 H), 1.84 - 1.92 (m, 2 H), 1.22 - 1.40 (m, 10 H), 0.78 - 0.88 (m, 3 H).

^{13}C NMR: (75 MHz, $CDCl_3$) δ 141.0, 135.7, 130.7, 130.5, 126.9, 126.5, 125.5, 47.5, 31.6, 30.2, 29.0, 28.9, 26.2, 22.5, 21.1, 13.9, 9.2, 8.7.

Melting point: solidified melt.

3-Dodecyl-4,5-dimethyl-1-(*p*-tolyl)-1*H*-imidazol-3-ium bromide (15)

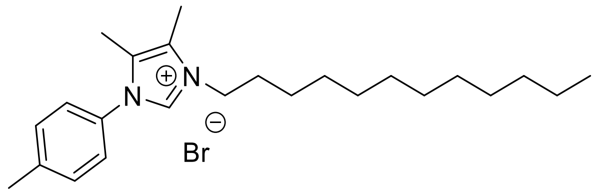

Following the general procedure, 23 mmol of imidazole **2** were alkylated using 1-bromo-dodecane. The product was obtained as a brown solidified melt (9.4 g, 22 mmol, 94%).

^{1}H NMR: (300MHz, $CDCl_3$) δ 10.06 (s, 1 H), 7.27 - 7.36 (m, 4 H), 4.36 - 4.47 (m, 2 H), 2.38 (s, 3 H), 2.30 (s, 3 H), 2.13 (s, 3 H), 1.77 - 1.93 (m, 2 H), 1.21 - 1.38 (m, 18 H), 0.78 - 0.88 (m, 3 H).

^{13}C NMR: (75 MHz, $CDCl_3$) δ 140.9 135.7, 130.7, 130.5, 126.8, 126.5, 125.5, 47.5, 31.7, 30.2, 29.5, 29.5, 29.4, 29.3, 29.2, 29.0, 26.2, 22.5, 21.1, 14.0, 9.2, 8.7.

Melting point: solidified melt.

3-Butyl-1-(4-methoxyphenyl)-4,5-dimethyl-1*H*-imidazol-3-ium bromide (16)

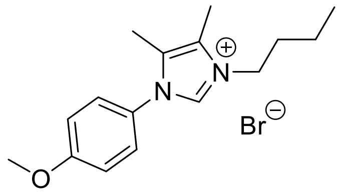

Following the general procedure, 55 mmol of imidazole **3** were alkylated using 1-bromobutane. The product was obtained as a brown solid (17.8 g, 53 mmol, 95%).

^{1}H NMR: (300MHz, $CDCl_3$) δ 10.01 (s, 1 H), 7.38 (m, 2 H), 6.97 (m, 2 H), 4.40 (t, *J* = 7.6 Hz, 2 H), 3.80 (s, 3 H), 2.28 (s, 3 H), 2.10 (s, 3 H), 1.83 (quin, *J* = 7.6 Hz, 2 H), 1.40 (sxt, *J* = 7.5 Hz, 2 H), 0.92 (t, *J* = 7.4 Hz, 3 H).

^{13}C NMR: (75 MHz, $CDCl_3$) δ 160.8, 135.7, 127.1, 127.1, 126.4, 125.6, 115.3, 115.2, 55.6, 47.2, 31.9, 19.5, 13.4, 9.1, 8.6.

Melting point: 58 °C.

1-(4-Methoxyphenyl)-4,5-dimethyl-3-octyl-1*H*-imidazol-3-ium bromide (17)

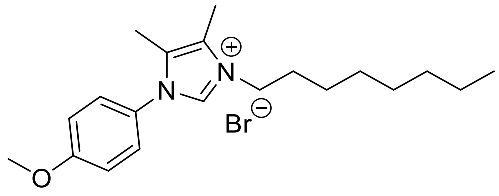

Following the general procedure, 50 mmol of imidazole **3** were alkylated using 1-bromooctane. The product was obtained as a brown solid (19.3 g, 49 mmol, 98%).

^{1}H NMR: (300MHz, $CDCl_3$) δ 10.01 (s, 1 H), 7.36 - 7.44 (m, 2 H), 6.94 - 7.03 (m, 2 H), 4.36 - 4.46 (m, 2 H), 3.81 (s, 3 H), 2.29 (s, 3 H), 2.11 (s, 3 H), 1.78 - 1.92 (m, 2 H), 1.15 - 1.34 (m, 10 H), 0.79 - 0.84 (m, 3 H).

^{13}C NMR: (75 MHz, $CDCl_3$) δ 160.9, 135.8, 127.1, 127.1, 126.4, 125.7,115.4, 115.2, 55.6, 47.5, 31.6, 30.1, 28.9, 26.3, 22.5, 13.9, 9.1, 8.7.

Melting point: 81°C.

3-Dodecyl-1-(4-methoxyphenyl)-4,5-dimethyl-1*H*-imidazol-3-ium bromide (18)

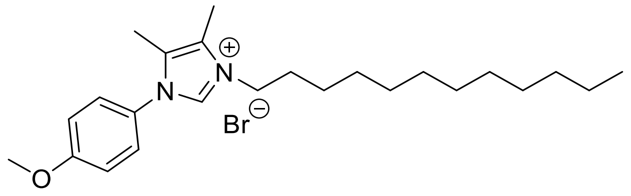

Following the general procedure, 45 mmol of imidazole **3** were alkylated using 1-bromododecane. The product was obtained as a brown solid (19.3 g, 43 mmol, 95%).

^{1}H NMR: (300MHz, $CDCl_3$) δ 10.00 (s, 1 H), 7.34 - 7.42 (m, 2 H), 6.92 - 7.03 (m, 2 H), 4.33 - 4.44 (m, 2 H), 3.79 (s, 3 H), 2.27 (s, 3 H), 2.10 (s, 3 H), 1.75 - 1.92 (m, 2 H), 1.08 - 1.26 (m, 18 H), 0.77 - 0.85 (m, 3 H).

^{13}C NMR: (75 MHz, $CDCl_3$) δ 160.8, 135.6, 127.0, 126.9, 126.3, 125.6, 115.3, 115.1, 55.6, 47.4, 31.7, 30.1, 29.4, 29.3, 29.2, 29.1, 28.9, 26.2, 22.5, 13.9, 9.1, 8.6.

Melting point: 88 °C.

1-(4-Bromophenyl)-3-butyl-4,5-dimethyl-1*H*-imidazol-3-ium bromide (19)

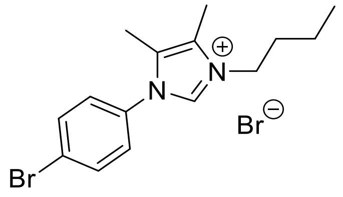

Following the general procedure, 55 mmol of imidazole **4** were alkylated using 1-bromobutane. The product was obtained as a brown solid (19.6 g, 50 mmol, 92%).

^{1}H NMR: (300MHz, $CDCl_3$) δ 10.21 (s, 1 H), 7.62 - 7.69 (m, 2 H), 7.42 - 7.50 (m, 2 H), 4.37 (t, *J* = 7.4 Hz, 2 H), 2.30 (s, 3 H), 2.15 (s, 3 H), 1.85 (quin, *J* = 7.6 Hz, 2 H), 1.40 (sxt, *J* = 7.4 Hz, 2 H), 0.92 (t, *J* = 7.4 Hz, 3 H).

^{13}C NMR: (75 MHz, $CDCl_3$) δ 135.8, 133.3, 132.0, 127.5, 126.9, 126.7, 124.8, 47.4, 31.8, 19.5, 13.4, 9.3, 8.7.

Melting point: 165 °C.

1-(4-Bromophenyl)-4,5-dimethyl-3-octyl-1*H*-imidazol-3-ium bromide (20)

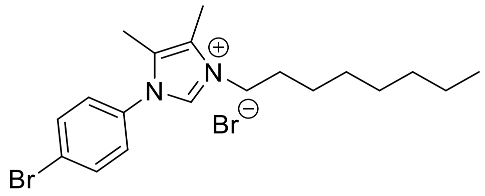

Following the general procedure, 50 mmol of imidazole **4** were alkylated using 1-bromooctane. The product was obtained as a brown solid (20.4 g, 46 mmol, 92%).

^{1}H NMR: (300MHz, $CDCl_3$) δ 10.25 (s, 1 H), 7.64 - 7.71 (m, 2 H), 7.44 - 7.55 (m, 2 H), 4.38 (t, *J* = 7.7 Hz, 2 H), 2.30 (s, 3 H), 2.16 (s, 3 H), 1.88 (quin, *J* = 7.5 Hz, 2 H), 1.17 - 1.31 (m, 10 H), 0.77 - 0.88 (m, 3 H).

^{13}C NMR: (75 MHz, $CDCl_3$) δ 136.0, 133.6, 133.4, 132.1, 127.5, 127.4, 126.9, 126.6, 124.5, 47.7, 31.6, 30.1, 28.9, 26.3, 22.5, 14.0, 9.3, 8.7.

Melting point: 107 °C.

1-(4-Bromophenyl)-3-dodecyl-4,5-dimethyl-1*H*-imidazol-3-ium bromide (21)

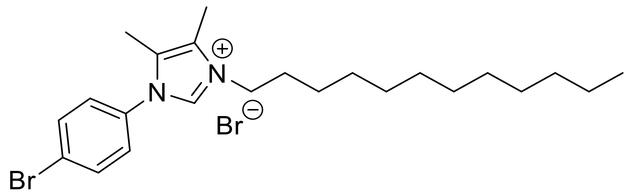

Following the general procedure, 45 mmol of imidazole **4** were alkylated using 1-bromododecane. The product was obtained as a brown solid (21.6 g, 43 mmol, 96%).

^{1}H NMR: (300MHz, $CDCl_3$) δ 10.26 (s, 1 H), 7.66 - 7.73 (m, 2 H), 7.47 - 7.56 (m, 2 H), 4.35 - 4.45 (m, 2 H), 2.32 (s, 3 H), 2.17 (s, 3 H), 1.83 - 1.98 (m, 2 H), 1.17 - 1.32 (m, 18 H), 0.81 - 0.90 (m, 3 H).

^{13}C NMR: (75 MHz, $CDCl_3$) δ 136.1, 133.7, 133.5, 132.1, 127.5, 127.4, 126.9, 126.6, 125.0, 47.8, 31.8, 30.1, 29.5, 29.5, 29.4, 29.3, 29.1, 26.4, 22.6, 14.1, 9.3, 8.7.

Melting point: 84°C.

3-Butyl-4,5-dimethyl-1-(4-(trifluoromethyl)phenyl)-1*H*-imidazol-3-ium bromide (22)

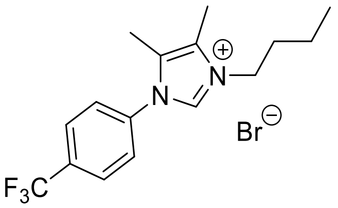

Following the general procedure, 55 mmol of imidazole **5** were alkylated using 1-bromobutane. The product was obtained as a brown solid (17.7 g, 47 mmol, 85%).

^{1}H NMR: (300MHz, $CDCl_3$) δ 10.28 (s, 1 H), 7.76 (s, 4 H), 4.33 (t, *J* = 7.5 Hz, 2 H), 2.29 (s, 3 H), 2.16 (s, 3 H), 1.83 (quin, *J* = 7.6 Hz, 2 H), 1.38 (sxt, *J* = 7.5 Hz, 2 H), 0.88 (t, *J* = 7.3 Hz, 3 H).

^{13}C NMR: (75 MHz, $CDCl_3$) δ 135.9, 135.7, 132.3 (q, *J* = 33.4 Hz), 127.3, 127.2, 126.7, 126.5, 124.8, 47.3, 31.6, 19.4, 13.3, 9.3, 8.6.

^{19}F NMR: (282 MHz, $CDCl_3$) δ -63.0.

Melting point: 95°C.

4,5-Dimethyl-3-octyl-1-(4-(trifluoromethyl)phenyl)-1*H*-imidazol-3-ium bromide (23)

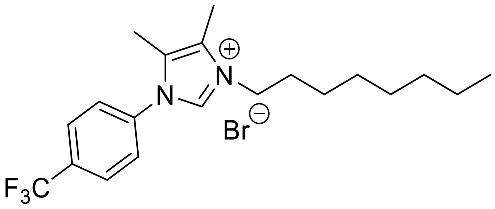

Following the general procedure, 50 mmol of imidazole **5** were alkylated using 1-bromooctane. The product was obtained as a brown solid (20.4 g, 47 mmol, 94%).

^{1}H NMR: (300MHz, $CDCl_3$) δ 10.31 (s, 1 H), 7.78 (s, 4 H), 4.29 - 4.41 (m, 2 H), 2.30 (s, 3 H), 2.18 (s, 3 H), 1.87 (quin, *J* = 7.6 Hz, 2 H), 1.16 - 1.39 (m, 10 H), 0.73 - 0.85 (m, 3 H).

^{13}C NMR: (75 MHz, $CDCl_3$) δ 135.9, 132.3 (q, *J* = 33.4 Hz), 127.4, 127.3, 127.2, 126.7, 126.5, 124.8, 47.7, 31.5, 29.9, 28.8, 26.2, 22.4, 13.8, 9.4, 8.7.

^{19}F NMR: (282 MHz, $CDCl_3$) δ -63.0.

Melting point: 113°C.

3-Dodecyl-4,5-dimethyl-1-(4-(trifluoromethyl)phenyl)-1*H*-imidazol-3-ium bromide (24)

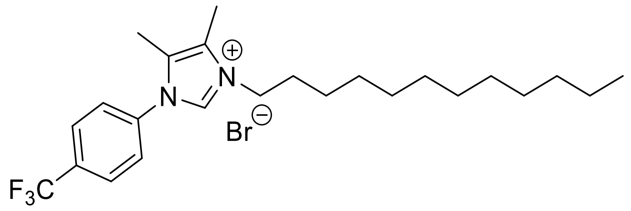

Following the general procedure, 45 mmol of imidazole **5** were alkylated using 1-bromo-dodecane. The product was obtained as a brown solid (21.4 g, 44 mmol, 97%).

^{1}H NMR: (300MHz, $CDCl_3$) δ 10.21 (s, 1 H), 7.79 (s, 4 H), 4.35 (t, *J* = 7.6 Hz, 2 H), 2.31 (s, 3 H), 2.18 (s, 3 H), 1.75 - 1.98 (m, 2 H), 1.16 - 1.40 (m, 18 H), 0.76 - 0.86 (m, 3 H).

^{13}C NMR: (75 MHz, $CDCl_3$) δ 136.0, 135.9, 132.3 (q, *J* = 33.4 Hz), 127.3, 127.2, 126.7, 126.6, 124.9, 47.7, 31.7, 30.0, 29.4, 29.4, 29.3, 29.1, 28.9, 26.3, 22.5, 20.0, 9.4, 8.7.

^{19}F NMR: (282 MHz, $CDCl_3$) δ -63.0.

Melting point: 90°C.

3-Butyl-4,5-dimethyl-1-(4-(trifluoromethoxy)phenyl)-1*H*-imidazol-3-ium bromide (25)

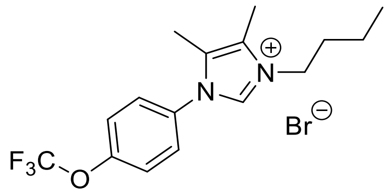

Following the general procedure, 55 mmol of imidazole **6** were alkylated using 1-bromobutane. The product was obtained as a brown solid (21.2 g, 54 mmol, 98%).

^{1}H NMR: (300MHz, $CDCl_3$) δ 10.16 (s, 1 H), 7.64-7.67 (m, 2 H), 7.34-7.37 (m, 2 H), 4.37 (t, *J* = 7.6 Hz, 2 H), 2.30 (s, 3 H), 2.15 (s, 3 H), 1.85 (quin, *J* = 7.6 Hz, 2 H), 1.39 (m, 2 H), 0.84 - 0.98 (m, 3 H).

^{13}C NMR: (75 MHz, $CDCl_3$) δ 150.2, 135.8, 131.3, 127.8, 127.0, 126.8, 122.2, 120.0 (q, *J* = 258.7 Hz), 47.3, 31.8, 19.4, 13.4, 9.3, 8.7.

^{19}F NMR: (282 MHz, $CDCl_3$) δ -57.9.

Melting point: 66°C.

4,5-Dimethyl-3-octyl-1-(4-(trifluoromethoxy)phenyl)-1*H*-imidazol-3-ium bromide (26)

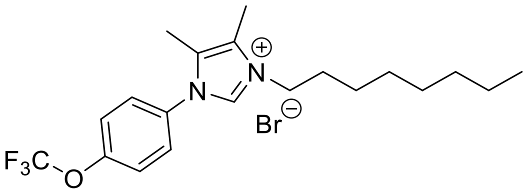

Following the general procedure, 50 mmol of imidazole **6** were alkylated using 1-bromooctane. The product was obtained as a brown solid (22 g, 49 mmol, 98%).

^{1}H NMR: (300MHz, $CDCl_3$) δ 10.17 (s, 1 H), 7.62 - 7.72 (m, 2 H), 7.34 - 7.41 (m, 2 H), 4.37 (t, *J* = 7.4 Hz, 2 H), 2.30 (s, 3 H), 2.16 (s, 3 H), 1.79 - 1.94 (m, 2 H), 1.17 - 1.41 (m, 10 H), 0.73 - 0.86 (m, 3 H).

^{13}C NMR: (75 MHz, $CDCl_3$) δ 150.2, 135.9, 131.3, 127.8, 127.0, 126.8, 122.3, 120.0 (q, *J* = 258.7 Hz), 47.6, 31.5, 30.0, 28.9, 26.2, 22.4, 13.9, 9.3, 8.7.

^{19}F NMR: (282 MHz, $CDCl_3$) δ -57.9.

Melting point: 107°C.

3-Dodecyl-4,5-dimethyl-1-(4-(trifluoromethoxy)phenyl)-1*H*-imidazol-3-ium bromide (27)

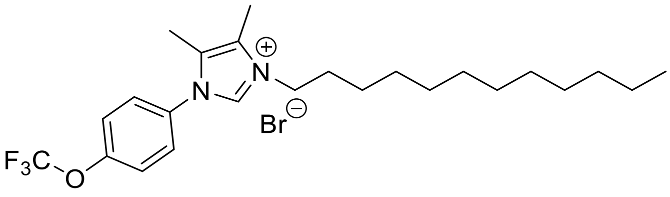

Following the general procedure, 45 mmol of imidazole **6** were alkylated using 1-bromododecane. The product was obtained as a brown solid (18.5 g, 37 mmol, 81%).

^{1}H NMR: (300MHz, $CDCl_3$) δ 10.16 (s, 1 H), 7.63 - 7.68 (m, 2 H), 7.32 - 7.42 (m, 2 H), 4.30 - 4.42 (m, 2 H), 2.30 (s, 3 H), 2.16 (s, 3 H), 1.80 – 1.93 (m, 2 H), 1.19 – 1.31 (m, 18 H), 0.75 - 0.87 (m, 3 H).

^{13}C NMR: (75 MHz, $CDCl_3$) δ 150.2, 135.9, 131.3, 127.8, 127.0, 126.8, 122.3, 120.0 (q, *J* = 258.7 Hz), 47.6, 31.7, 30.0, 29.4, 29.4, 29.3, 29.1, 29.0, 26.3, 22.5, 13.4, 9.3, 8.7.

^{19}F NMR: (282 MHz, $CDCl_3$) δ -57.9.

Melting point: 79°C.

3-Butyl-4,5-dimethyl-1-(*o*-tolyl)-1*H*-imidazol-3-ium bromide (28)

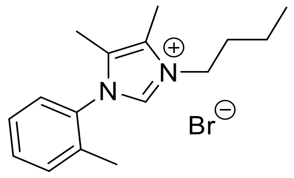

Following the general procedure, 54 mmol of imidazole **7** were alkylated using 1-bromobutane. The product was obtained as a brown solidified melt (15.2 g, 47 mmol, 87%).

^{1}H NMR: (300MHz, $CDCl_3$) δ 10.06 (s, 1 H), 7.27 - 7.49 (m, 4 H), 4.55 (t, *J* = 7.4 Hz, 2 H), 2.34 (s, 3 H), 2.12 (s, 3 H), 1.99 (s, 3 H), 1.86 (m, 2 H), 1.37 - 1.52 (m, 2 H), 0.96 (t, *J* = 7.3 Hz, 3 H).

^{13}C NMR: (75 MHz, $CDCl_3$) δ 136.1, 134.8, 132.0, 131.8, 131.2, 127.7, 127.3, 127.3, 126.4, 47.4, 32.1, 19.5, 17.5, 13.6, 8.8, 8.6.

Melting point: solidified melt.

4,5-Dimethyl-3-octyl-1-(o-tolyl)-1*H*-imidazol-3-ium bromide (29)

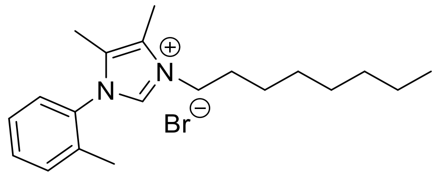

Following the general procedure, 54 mmol of imidazole **7** were alkylated using 1-bromooctane. The product was obtained as a brown solidified melt (18.3 g, 48 mmol, 90%).

^{1}H NMR: (300MHz, $CDCl_3$) δ 10.04 (s, 1 H), 7.27 - 7.49 (m, 4 H), 4.53 (t, *J* = 7.6 Hz, 2 H), 2.33 (s, 3 H), 2.11 (s, 3 H), 1.99 (s, 3 H), 1.87 (quin, *J* = 7.4 Hz, 2 H), 1.17 - 1.43 (m, 10 H), 0.78 - 0.88 (m, 3 H).

^{13}C NMR: (75 MHz, $CDCl_3$) δ 136.0, 134.8, 132.0, 131.8, 131.2, 127.7, 127.3, 126.4, 47.6, 31.6, 30.2, 29.0, 26.2, 22.5, 17.5, 14.0, 8.8, 8.6.

Melting point: solidified melt.

3-Dodecyl-4,5-dimethyl-1-(o-tolyl)-1*H*-imidazol-3-ium bromide (30)

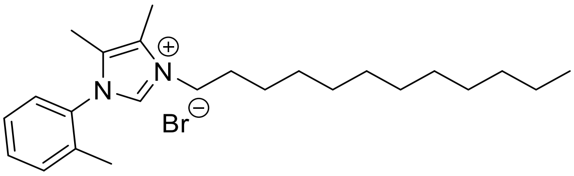

Following the general procedure, 48 mmol of imidazole **7** were alkylated using 1-bromododecane. The product was obtained as a brown solid (18.4 g, 42 mmol, 87%).

^{1}H NMR: (300MHz, $CDCl_3$) δ 10.07 (s, 1 H), 7.27 - 7.50 (m, 4 H), 4.50 - 4.59 (m, 2 H), 2.34 (s, 3 H), 2.12 (s, 3 H), 1.99 (s, 3 H), 1.82 - 1.94 (m, 2 H), 1.20 - 1.33 (m, 18 H), 0.80 - 0.90 (m, 3 H).

^{13}C NMR: (75 MHz, $CDCl_3$) δ 136.2, 134.8, 132.1, 131.8, 131.2, 127.7, 127.4, 127.2, 126.4, 47.6, 31.8, 30.2, 29.5, 29.5, 29.4, 29.3, 29.1, 26.2, 22.6, 17.5, 14.0, 8.8, 8.6.

Melting point: 54°C.

3-Butyl-1-(2-methoxyphenyl)-4,5-dimethyl-1*H*-imidazol-3-ium bromide (31)

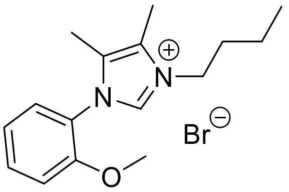

Following the general procedure, 50 mmol of imidazole **8** were alkylated using 1-bromobutane. The product was obtained as a brown solid (12.5 g, 37 mmol, 74%).

^{1}H NMR: (300MHz, $CDCl_3$) δ 9.77 (s, 1 H), 7.29 - 7.49 (m, 2 H), 6.95 - 7.04 (m, 2 H), 4.43 (t, *J* = 7.4 Hz, 2 H), 3.75 (s, 3 H), 2.27 (s, 3 H), 1.95 (s, 3 H), 1.71 - 1.87 (m, 2 H), 1.30 - 1.47 (m, 2 H), 0.88 (t, *J* = 7.4 Hz, 3 H).

^{13}C NMR: (75 MHz, $CDCl_3$) δ 153.5, 135.8, 132.3, 128.1, 128.0, 125.7, 121.3, 121.2, 112.2, 55.8, 47.0, 31.8, 19.2, 13.4, 8.6, 8.5.

Melting Point: 133°C.

1-(2-Methoxyphenyl)-4,5-dimethyl-3-octyl-1*H*-imidazol-3-ium bromide (32)

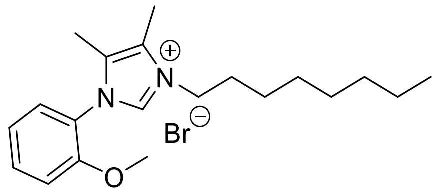

Following the general procedure, 35 mmol of imidazole **8** were alkylated using 1-bromooctane. The product was obtained as a brown solidified melt (13.7 g, 34 mmol, 99%).

^{1}H NMR: (300MHz, $CDCl_3$) δ 9.85 (s, 1 H), 7.37 - 7.52 (m, 2 H), 7.01 - 7.11 (m, 2 H), 4.47 (t, *J* = 7.6 Hz, 2 H), 3.80 (s, 3 H), 2.30 (s, 3 H), 1.99 (s, 3 H), 1.77 - 1.92 (m, 2 H), 1.11 - 1.36 (m, 10 H), 0.79 - 0.84 (m, 3 H).

^{13}C NMR: (75 MHz, $CDCl_3$) δ 153.7, 136.2, 132.4, 128.3, 128.1, 125.7, 121.5, 121.4, 112.3, 55.9, 47.5, 31.6, 30.1, 29.0, 26.1, 22.4, 13.9, 8.6, 8.6.

Melting point: solidified melt.

3-Dodecyl-1-(2-methoxyphenyl)-4,5-dimethyl-1*H*-imidazol-3-ium bromide (33)

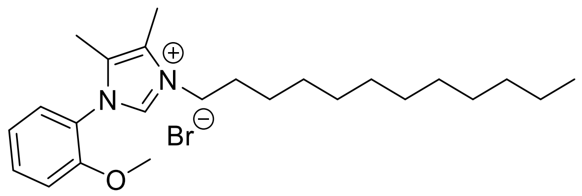

Following the general procedure, 25 mmol of imidazole **8** were alkylated using 1-bromo-dodecane. The product was obtained as a brown solidified melt (9.7 g, 22 mmol, 86%).

^{1}H NMR: (300MHz, $CDCl_3$) δ 9.89 (s, 1 H), 7.40 - 7.55 (m, 2 H), 7.02 - 7.15 (m, 2 H), 4.49 (t, *J* = 7.6 Hz, 2 H), 3.82 (s, 3 H), 2.31 (s, 3 H), 2.01 (s, 3 H), 1.81 - 1.94 (m, 2 H), 1.18 - 1.37 (m, 18 H), 0.81 - 0.87 (m, 3 H).

^{13}C NMR: (75 MHz, $CDCl_3$) δ 153.7, 136.4, 132.5, 128.4, 128.1, 125.7, 121.6, 121.5, 112.3, 56.0, 47.5, 31.8, 30.2, 29.5, 29.5, 29.4, 29.2, 29.1, 26.2, 22.6, 14.0, 8.7, 8.6.

Melting point: solidified melt.

3-Butyl-1-(2,4-difluorophenyl)-4,5-dimethyl-1*H*-imidazol-3-ium bromide (34)

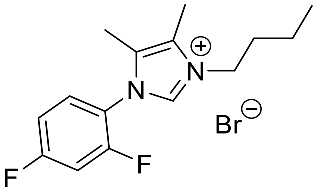

Following the general procedure, 50 mmol of imidazole **9** were alkylated using 1-bromobutane. The product was obtained as a brown solidified melt (17 g, 49 mmol, 98%).

^{1}H NMR: (300MHz, $CDCl_3$) δ 10.15 (s, 1 H), 7.79 - 7.92 (m, 1 H), 6.95 - 7.13 (m, 2 H), 4.37 (t, *J* = 7.6 Hz, 2 H), 2.30 (s, 3 H), 2.05 (s, 3 H), 1.85 (quin, *J* = 7.6 Hz, 2 H), 1.31 - 1.46 (m, 2 H), 0.91 (t, *J* = 7.3 Hz, 3 H).

^{13}C NMR: (75 MHz, $CDCl_3$) δ 165.6 (dd, *J* = 267.0, 10.7 Hz), 158.3 (dd, *J* = 268.8, 13.1 Hz), 136.6, 130.7 (d, *J* = 10.7 Hz), 127.9, 126.6, 117.2, 113.4 (dd, *J* = 26.8, 3.6 Hz), 105.8 (dd, *J* = 49.5, 22.7 Hz), 47.5, 31.6, 19.4, 13.3, 8.6, 8.5.

^{19}F NMR: (282 MHz, $CDCl_3$) δ -102.8, -116.4.

Melting point: solidified melt.

1-(2,4-Difluorophenyl)-4,5-dimethyl-3-octyl-1*H*-imidazol-3-ium bromide (35)

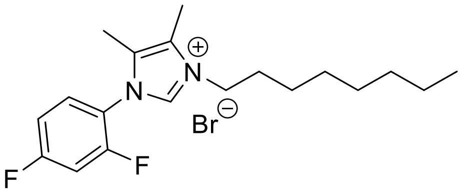

Following the general procedure, 43 mmol of imidazole **9** were alkylated using 1-bromooctane. The product was obtained as a brown solid (14.4 g, 36 mmol, 84%).

^{1}H NMR: (300MHz, $CDCl_3$) δ 10.15 (s, 1 H), 7.90 - 8.01 (m, 1 H), 6.99 - 7.15 (m, 2 H), 4.40 (t, *J* = 7.6 Hz, 2 H), 2.32 (s, 3 H), 2.09 (s, 3 H), 1.84 - 1.97 (m, 2 H), 1.14 - 1.36 (m, 10 H), 0.78 - 0.87 (m, 3 H).

^{13}C NMR: (75 MHz, $CDCl_3$) δ 165.7 (dd, *J* = 267.0, 10.7 Hz), 158.4 (dd, *J* = 268.8, 13.1 Hz), 136.8, 130.9 (d, *J* = 10.1 Hz), 127.9, 126.5, 117.2, 113.6 (dd, *J* = 26.8, 3.6 Hz), 105.8 (dd, *J* = 50.1, 22.7 Hz), 47.9, 31.6, 29.9, 28.9, 26.2, 22.5, 19.9, 8.7, 8.6.

^{19}F NMR: (282 MHz, $CDCl_3$) δ -102.7, -116.5.

Melting point: 57 °C.

1-(2,4-Difluorophenyl)-3-dodecyl-4,5-dimethyl-1*H*-imidazol-3-ium bromide (36)

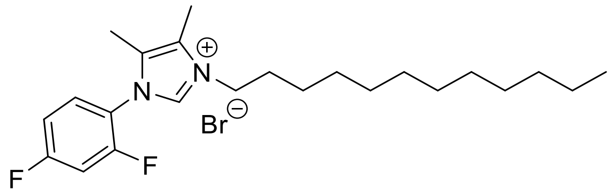

Following the general procedure, 38 mmol of imidazole **9** were alkylated using 1-bromododecane. The product was obtained as a brown solid (12.7 g, 28 mmol, 73%).

^{1}H NMR: (300MHz, $CDCl_3$) δ 10.16 (s, 1 H), 7.89 - 8.01 (m, 1 H), 6.98 - 7.15 (m, 2 H), 4.39 (t, *J* = 7.4 Hz, 2 H), 2.32 (s, 3 H), 2.09 (s, 3 H), 1.83 - 1.96 (m, 2 H), 1.18 - 1.33 (m, 18 H), 0.79 - 0.86 (m, 3 H).

^{13}C NMR: (75 MHz, $CDCl_3$) δ 165.7 (dd, *J* = 267.0, 10.7 Hz), 158.4 (dd, *J* = 268.8, 13.1 Hz), 136.8, 130.9 (d, *J* = 10.1 Hz), 127.9, 126.5, 117.2, 113.6 (dd, *J* = 26.8, 3.6 Hz), 105.8 (dd, *J* = 49.5, 22.7 Hz), 47.9, 31.8, 29.9, 29.5, 29.4, 29.3, 29.2, 29.0, 26.2, 22.5, 14.0, 8.7,8.6 (d, *J* = 3 Hz).

^{19}F NMR: (282 MHz, $CDCl_3$) δ -102.7, -116.5.

Melting point: 69 °C.

S3 Synthesis of NTf_2 ionic liquids

General procedure for the anion exchange

In a round bottom flask, the corresponding bromide salt (1 equiv) was dissolved in a small amount of methanol. $LiNTf_2$ (1.1 equiv 70% aq. solution), additional water and DCM were added and the reaction mixture was stirred at room temperature for 24 h. The organic phase was separated and the water was extracted with DCM. The combined organic phases were washed three times with water and dried over $MgSO_4$. The solvent was removed under reduced pressure to yield the corresponding ionic liquid.

3-Butyl-4,5-dimethyl-1-phenyl-1*H*-imidazol-3-ium bis(trifluoromethylsulfonyl)amide (37)

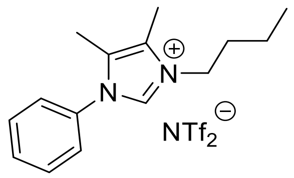

Following the general procedure, 16 mmol of bromide salt **10** were used for the anion exchange. The product was obtained as a dark green oil (7 g, 14 mmol, 85%).

^{1}H NMR: (300MHz, $CDCl_3$) δ 8.57 (s, 1 H), 7.52 - 7.62 (m, 3 H), 7.38 - 7.45 (m, 2 H), 4.10 - 4.21 (m, 2 H), 2.34 (s, 3 H), 2.16 (s, 3 H), 1.78 - 1.93 (m, 2 H), 1.43 (sxt, *J* = 7.5 Hz, 2 H), 0.99 (t, *J* = 7.4 Hz, 3 H).

^{13}C NMR: (75 MHz, $CDCl_3$) δ 133.6, 133.0, 130.1, 130.3, 128.0, 127.4, 125.9 ,119.9 (q, *J* = 321.5 Hz), 47.5, 31.5, 19.6, 13.3, 9.0, 8.5.

^{19}F NMR: (282 MHz, $CDCl_3$) δ -79.6.

Melting point: liquid at room temperature.

Elemental analysis:

Calculated: C: 40.08%, H: 4.15%, N: 8.25%, S: 12.59%.
Found: C: 40.41%, H: 4.29%, N: 7.82%, S: 12.43%.

4,5-Dimethyl-3-octyl-1-phenyl-1*H*-imidazol-3-ium bis(trifluoromethylsulfonyl)amide (38)

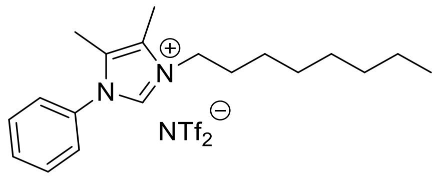

Following the general procedure, 14 mmol of bromide salt **11** were used for the anion exchange. The product was obtained as a dark green oil (7 g, 12 mmol, 89%).

^{1}H NMR: (300MHz, $CDCl_3$) δ 8.56 (s, 1 H), 7.55 - 7.63 (m, 3 H), 7.38 - 7.47 (m, 2 H), 4.15 (t, *J* = 7.7 Hz, 2 H), 2.34 (s, 3 H), 2.16 (s, 3 H), 1.80 - 1.93 (m, 2 H), 1.22 - 1.41 (m, 10 H), 0.83 - 0.92 (m, 3 H).

^{13}C NMR: (75 MHz, $CDCl_3$) δ 133.6, 133.0, 131.0, 130.3, 128.0, 127.3, 126.0,119.9 (q, *J* = 321.5 Hz), 47.7, 31.6, 29.6, 29.0, 28.9, 26.3, 22.5, 14.0, 9.0, 8.6.

^{19}F NMR: (282 MHz, $CDCl_3$) δ -79.6.

Melting point: liquid at room temperature.

Elemental analysis:

Calculated: C: 44.60%, H: 5.17%, N: 7.43%, S: 11.34%.
Found: C: 44.54%, H: 5.42%, N: 7.41%, S: 11.20%.

3-Dodecyl-4,5-dimethyl-1-phenyl-1*H*-imidazol-3-ium bis(trifluoromethylsulfonyl)amide (39)

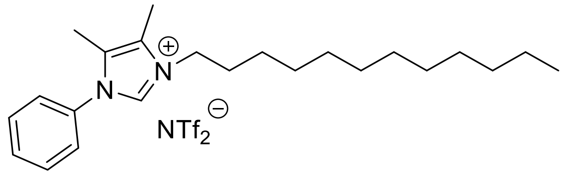

Following the general procedure, 12 mmol of bromide salt **12** were used for the anion exchange. The product was obtained as a light green oil (6.6 g, 11 mmol, 88%).

^{1}H NMR: (300MHz, $CDCl_3$) δ 8.56 (s, 1 H) 7.54 - 7.62 (m, 3 H) 7.38 - 7.47 (m, 2 H) 4.15 (t, *J* = 7.7 Hz, 2 H) 2.34 (s, 3 H) 2.16 (s, 3 H) 1.81 - 1.93 (m, 2 H) 1.18 - 1.35 (m, 18 H) 0.81 - 0.93 (m, 3 H).

^{13}C NMR: (75 MHz, $CDCl_3$) δ 133.7, 133.0, 131.0, 130.3, 128.0, 127.3, 126.0,119.9 (q, *J* = 321.5 Hz), 47.8, 31.9, 29.6, 29.6, 29.5, 29.3, 29.3, 29.0, 26.3, 22.7, 14.1, 9.1, 8.6.

^{19}F NMR: (282 MHz, $CDCl_3$) δ -79.6.

Melting point: liquid at room temperature.

Elemental analysis:

Calculated: C: 48.30%, H: 6.00%, N: 6.76%, S: 10.31%.
Found: C: 48.11%, H: 6.36%, N: 6.94%, S: 10.22%.

3-Butyl-4,5-dimethyl-1-(*p*-tolyl)-1*H*-imidazol-3-ium bis(trifluoromethylsulfonyl)amide (40)

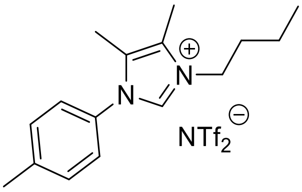

Following the general procedure, 16 mmol of bromide salt **13** were used for the anion exchange. The product was obtained as a dark brown oil (7.5 g, 14 mmol, 89%).

^{1}H NMR: (300MHz, $CDCl_3$) δ 8.51 (s, 1 H), 7.33 - 7.38 (m, 2 H), 7.27 (d, *J* = 7.7 Hz, 2 H), 4.07 - 4.20 (m, 2 H), 2.44 (s, 3 H), 2.33 (s, 3 H), 2.14 (s, 3 H), 1.76 - 1.91 (m, 2 H), 1.43 (sxt, *J* = 7.4 Hz, 2 H), 0.98 (t, *J* = 7.3 Hz, 3 H).

^{13}C NMR: (75 MHz, $CDCl_3$) δ 141.4, 133.5, 130.8, 130.4, 128.1, 127.2, 125.6, 119.9 (q, *J* = 321.5 Hz), 47.4, 31.5, 21.2, 20.0, 13.3, 9.0, 8.5.

^{19}F NMR: (282 MHz, $CDCl_3$) δ -79.6.

Melting point: liquid at room temperature.

Elemental analysis:

Calculated: C: 41.30%, H: 4.43%, N: 8.03%, S: 12.25%.
Found: C: 41.63%, H: 4.62%, N: 7.68%, S: 11.99%.

4,5-Dimethyl-3-octyl-1-(*p*-tolyl)-1*H*-imidazol-3-ium bis(trifluoromethylsulfonyl)amide (41)

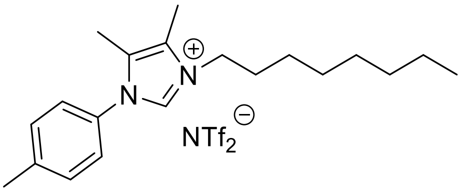

Following the general procedure, 13 mmol of bromide salt **14** were used for the anion exchange. The product was obtained as a dark brown oil (7.2 g, 12 mmol, 94%).

^{1}H NMR: (300MHz, $CDCl_3$) δ 8.50 (s, 1 H), 7.33 - 7.39 (m, 2 H), 7.27 (d, *J* = 8.8 Hz, 2 H), 4.13 (t, *J* = 7.0 Hz, 2 H), 2.44 (s, 3 H), 2.33 (s, 3 H), 2.14 (s, 3 H), 1.81 – 1.92 (m, 2 H), 1.22 - 1.37 (m, 10 H), 0.84 - 0.90 (m, 3 H).

^{13}C NMR: (75 MHz, $CDCl_3$) δ 141.4, 133.6, 130.1, 130.4, 128.1, 127.2, 125.7, 119.9 (q, *J* = 321.9 Hz), 47.7, 29.6, 29.0, 28.9, 26.3, 22.5, 21.2, 14.0, 9.0, 8.6.

^{19}F NMR: (282 MHz, $CDCl_3$) δ -79.6.

Melting point: liquid at room temperature.

Elemental analysis:

Calculated: C: 45.59%, H: 5.39%, N: 7.25%, S: 11.06%.
Found: C: 45.89%, H: 5.66%, N: 6.97%, S: 10.96%.

3-Dodecyl-4,5-dimethyl-1-(*p*-tolyl)-1*H*-imidazol-3-ium bis(trifluoromethylsulfonyl)amide (42)

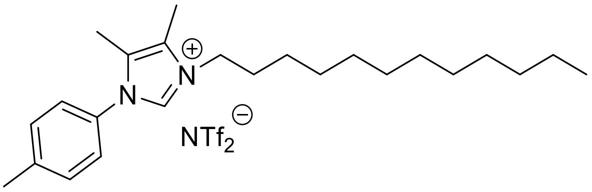

Following the general procedure, 12 mmol of bromide salt **15** were used for the anion exchange. The product was obtained as a dark brown oil (6.7 g, 11 mmol, 92%).

^{1}H NMR: (300MHz, $CDCl_3$) δ 8.51 (s, 1 H), 7.33 - 7.39 (m, 2 H), 7.28 (d, *J* = 9.4 Hz, 2 H), 4.14 (t, *J* = 7.7 Hz, 2 H), 2.44 (s, 3 H), 2.33 (s, 3 H), 2.14 (s, 3 H), 1.79 - 1.93 (m, 2 H), 1.18 - 1.35 (m, 18 H), 0.82 - 0.91 (m, 3 H).

^{13}C NMR: (75 MHz, $CDCl_3$) δ 141.4, 133.6, 130.8, 130.4, 128.0, 127.2, 125.6, 119.9 (q, *J* = 321.9 Hz), 47.7, 31.9, 29.6, 29.6, 29.5, 29.3, 29.3, 28.9, 26.3, 22.6, 21.2, 14.1, 9.0, 8.6.

^{19}F NMR: (282 MHz, $CDCl_3$) δ -79.6.

Melting point: liquid at room temperature.

Elemental analysis:

Calculated: C: 49.12%, H: 6.18%, N: 6.61%, S: 10.09%.
Found: C: 49.34%, H: 6.35%, N: 6.21%, S: 10.02%.

3-Butyl-1-(4-methoxyphenyl)-4,5-dimethyl-1*H*-imidazol-3-ium bis(trifluoromethylsulfonyl)amide (43)

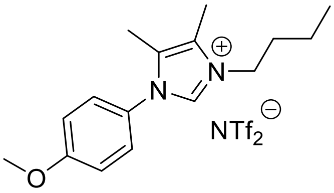

Following the general procedure, 18 mmol of bromide salt **16** were used for the anion exchange. The product was obtained as a dark brown oil (8.9 g, 16 mmol, 93%).

^{1}H NMR: (300MHz, $CDCl_3$) δ 8.47 (s, 1 H), 7.30 – 7.33 (m, 2 H), 7.01 – 7.04 (m, 2 H), 4.12 (t, *J* = 7.7 Hz, 2 H), 3.86 (s, 3 H), 2.32 (s, 3 H), 2.12 (s, 3 H), 1.84 (quin, *J* = 7.6 Hz, 2 H), 1.42 (sxt, *J* = 7.4 Hz, 2 H), 0.98 (t, *J* = 7.3 Hz, 3 H).

^{13}C NMR: (75 MHz, $CDCl_3$) δ 161.2, 133.6, 128.3, 127.3, 127.1, 125.5, 119.9 (q, *J* = 321.9 Hz), 115.3, 55.7, 47.4, 31.4, 19.5, 13.3, 8.9, 8.5.

^{19}F NMR: (282 MHz, $CDCl_3$) δ -79.6.

Melting point: liquid at room temperature.

Elemental analysis:

Calculated: C: 40.07%, H: 4.30%, N: 7.79%, S: 11.88%.
Found: C: 39.95%, H: 4.68%, N: 7.50%, S: 11.74%.

1-(4-Methoxyphenyl)-4,5-dimethyl-3-octyl-1*H*-imidazol-3-ium bis(trifluoromethylsulfonyl)amide (44)

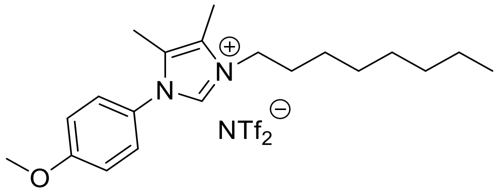

Following the general procedure, 13 mmol of bromide salt **17** were used for the anion exchange. The product was obtained as a dark brown oil (6.3 g, 11 mmol, 80%).

^{1}H NMR: (300MHz, $CDCl_3$) δ 8.48 (s, 1 H), 7.29 - 7.36 (m, 2 H), 6.99 - 7.07 (m, 2 H), 4.13 (t, *J* = 7.4 Hz, 2 H), 3.86 (s, 3 H), 2.32 (s, 3 H), 2.13 (s, 3 H), 1.81 - 1.91 (m, 2 H), 1.24 - 1.38 (m, 10 H), 0.83 - 0.90 (m, 3 H).

^{13}C NMR: (75 MHz, $CDCl_3$) δ 161.2, 133.6, 128.3, 127.3, 127.1, 125.5, 119.9 (q, *J* = 321.3 Hz), 115.3, 55.7, 47.6, 31.6, 29.6, 29.0, 28.9, 26.3, 22.5, 14.0, 8.9, 8.5.

^{19}F NMR: (282 MHz, $CDCl_3$) δ -79.6.

Melting point: liquid at room temperature.

Elemental analysis:

Calculated: C: 44.36%, H: 5.25%, N: 7.06%, S: 10.77%.
Found: C: 44.27%, H: 5.54%, N: 7.03%, S: 10.69%.

3-Dodecyl-1-(4-methoxyphenyl)-4,5-dimethyl-1*H*-imidazol-3-ium bis(trifluoromethylsulfonyl)amide (45)

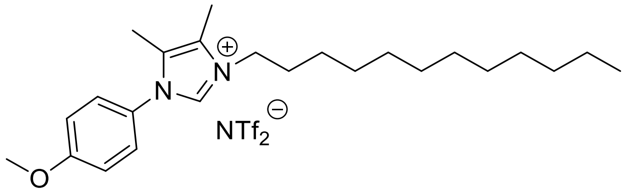

Following the general procedure, 11 mmol of bromide salt **18** were used for the anion exchange. The product was obtained as a dark orange oil (6.5 g, 10 mmol, 89%).

^{1}H NMR: (300MHz, $CDCl_3$) δ 8.44 (s, 1 H), 7.28 - 7.35 (m, 2 H), 6.99 - 7.06 (m, 2 H), 4.05 - 4.16 (m, 2 H), 3.85 (s, 3 H), 2.30 (s, 3 H), 2.11 (s, 3 H), 1.84 (quin, *J* = 7.5 Hz, 2 H), 1.16 - 1.44 (m, 18 H), 0.82 - 0.90 (m, 3 H).

^{13}C NMR: (75 MHz, $CDCl_3$) δ 161.2, 133.8, 128.3, 127.3, 126.1, 125.5, 119.9 (q, *J* = 321.3 Hz), 115.2, 55.7, 47.5, 31.8, 29.5, 29.4, 29.3, 29.3, 28.9, 26.3, 22.6, 14.0, 8.9, 8.5.

^{19}F NMR: (282 MHz, $CDCl_3$) δ -79.6.

Melting point: liquid at room temperature.

Elemental analysis:

Calculated: C: 47.92%, H: 6.03%, N: 6.45%, S: 9.84%.
Found: C: 48.09%, H: 6.18%, N: 6.30%, S: 9.90%.

1-(4-Bromophenyl)-3-butyl-4,5-dimethyl-1*H*-imidazol-3-ium bis(trifluoromethylsulfonyl)amide (46)

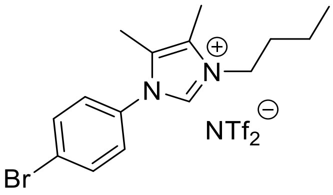

Following the general procedure, 13 mmol of bromide salt **19** were used for the anion exchange. The product was obtained as a dark brown oil (7.2 g, 12 mmol, 95%).

^{1}H NMR: (300MHz, $CDCl_3$) δ 8.55 (s, 1 H), 7.66 - 7.75 (m, 2 H), 7.29 - 7.39 (m, 2 H), 4.13 (t, *J* = 7.7 Hz, 2 H), 2.32 (s, 3 H), 2.15 (s, 3 H), 1.78 - 1.91 (m, 2 H), 1.43 (sxt, *J* = 7.4 Hz, 2 H), 0.98 (t, *J* = 7.3 Hz, 3 H).

^{13}C NMR: (75 MHz, $CDCl_3$) δ 133.7, 133.5, 132.0, 128.0, 127.7, 127.6, 125.3, 119.9 (q, *J* = 321.3 Hz), 47.6, 31.4, 19.6, 13.3, 9.0, 8.6.

^{19}F NMR: (282 MHz, $CDCl_3$) δ -79.6.

Melting point: liquid at room temperature.

Elemental analysis:

Calculated: C: 34.70%, H: 3.43%, N: 7.14%, S: 10.90%.
Found: C: 34.56%, H: 3.43%, N: 7.52%, S: 10.75%.

1-(4-Bromophenyl)-4,5-dimethyl-3-octyl-1*H*-imidazol-3-ium bis(trifluoromethylsulfonyl)amide (47)

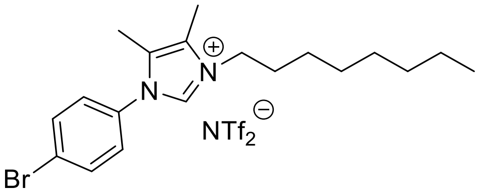

Following the general procedure, 11 mmol of bromide salt **20** were used for the anion exchange. The product was obtained as a dark brown oil (7.0 g, 10 mmol, 96%).

^{1}H NMR: (300MHz, $CDCl_3$) δ 8.56 (s, 1 H), 7.65 - 7.72 (m, 2 H), 7.29 - 7.36 (m, 2 H), 4.04 - 4.14 (m, 2 H), 2.33 (s, 3 H), 2.15 (s, 3 H), 1.78 - 1.91 (m, 2 H), 1.18 - 1.44 (m, 10 H), 0.81 - 0.90 (m, 3 H).

^{13}C NMR: (75 MHz, $CDCl_3$) δ 133.9, 133.7, 133.5, 132.0, 128.0, 127.7, 125.4, 119.9 (q, *J* = 321.3 Hz), 48.0, 31.9, 29.6, 29.3, 28.9, 26.3, 22.6, 14.0, 9.2, 8.7.

^{19}F NMR: (282 MHz, $CDCl_3$) δ -79.6.

Melting point: liquid at room temperature.

Elemental analysis:

Calculated: C: 39.14%, H: 4.38%, N: 6.52%, S: 9.95%.
Found: C: 38.73%, H: 4.47%, N: 6.93%, S: 9.78%.

1-(4-Bromophenyl)-3-dodecyl-4,5-dimethyl-1*H*-imidazol-3-ium bis(trifluoromethylsulfonyl)amide (48)

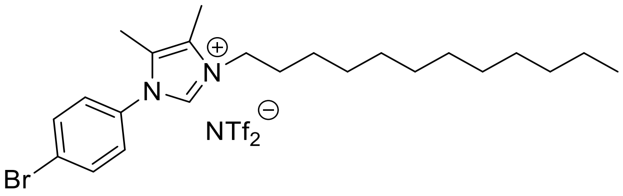

Following the general procedure, 10 mmol of bromide salt **21** were used for the anion exchange. The product was obtained as an orange solid. (6.9 g, 10 mmol, 99%).

^{1}H NMR: (300MHz, $CDCl_3$) δ 8.55 (s, 1 H), 7.65 - 7.73 (m, 2 H), 7.29 - 7.37 (m, 2 H), 4.04 - 4.18 (m, 2 H), 2.33 (s, 3 H), 2.15 (s, 3 H), 1.79 - 1.91 (m, 2 H), 1.20 - 1.37 (m, 18 H), 0.82 - 0.92 (m, 3 H).

^{13}C NMR: (75 MHz, $CDCl_3$) δ 133.8, 133.7, 133.5, 132.0, 128.0, 127.7, 125.4, 119.9 (q, *J* = 321.3 Hz), 41.8, 31.8, 29.5, 29.5, 29.4, 29.3, 29.3, 28.9, 26.3, 22.6, 14.1, 8.9, 8.5.

^{19}F NMR: (282 MHz, $CDCl_3$) δ -79.6.

Melting point: 27 °C.

Elemental analysis:

Calculated: C: 42.86%, H: 5.18%, N: 6.00%, S: 9.15%.
Found: C: 43.22%, H: 5.36%, N: 5.63%, S: 9.16%.

3-Butyl-4,5-dimethyl-1-(4-(trifluoromethyl)phenyl)-1*H*-imidazol-3-ium bis(trifluoromethylsulfonyl)amide (49)

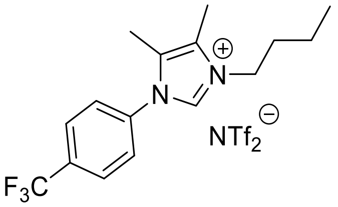

Following the general procedure, 13mmol of bromide salt **22** were used for the anion exchange. The product was obtained as an orange oil. (6.7 g, 12 mmol, 87%).

^{1}H NMR: (300MHz, CDCl3) δ 8.62 (s, 1 H), 7.81 - 7.89 (m, 2 H), 7.59 - 7.66 (m, 2 H), 4.07 - 4.23 (m, 2 H), 2.34 (s, 3 H), 2.17 (s, 3 H), 1.79 - 1.92 (m, 2 H), 1.43 (sxt, J = 7.5 Hz, 2 H), 0.98 (t, J = 7.3 Hz, 3 H).

^{13}C NMR: (75 MHz, CDCl3) δ 135.9, 133.8, 133.3, 132.8, 127.9, 127.5, 127.4, 126.8, 119.9 (q, J = 321.3 Hz), 47.7, 31.3, 19.5, 13.3, 9.0, 8.5.

^{19}F NMR: (282 MHz, CDCl3) δ -63.7, -79.7.

Melting point: liquid at room temperature.

Elemental analysis:

Calculated: C: 37.44%, H: 3.49%, N: 7.28%, S: 11.10%.
Found: C: 37.57%, H: 3.71%, N: 7.00%, S: 11.09%.

4,5-Dimethyl-3-octyl-1-(4-(trifluoromethyl)phenyl)-1*H*-imidazol-3-ium bis(trifluoromethylsulfonyl)amide (50)

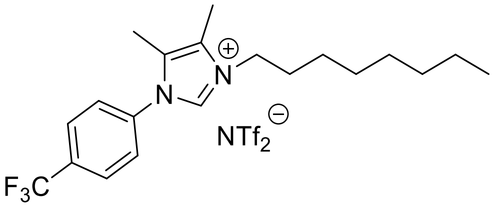

Following the general procedure, 12 mmol of bromide salt **23** were used for the anion exchange. The product was obtained as an orange oil. (7.2 g, 11 mmol, 98%).

^{1}H NMR: (300MHz, $CDCl_3$) δ 8.62 (s, 1 H), 7.85 (d, *J* = 8.5 Hz, 2 H), 7.62 (d, *J* = 8.3 Hz, 2 H), 4.09 - 4.20 (m, 2 H), 2.34 (s, 3 H), 2.17 (s, 3 H), 1.81 - 1.95 (m, 2 H), 1.18 - 1.47 (m, 10 H), 0.82 - 0.93 (m, 3 H).

^{13}C NMR: (75 MHz, $CDCl_3$) δ 135.9, 133.8, 133.3, 132.9, 127.9, 127.5, 127.4, 126.8, 119.9 (q, *J* = 321.3 Hz), 47.9, 31.6, 29.5, 28.9, 28.8, 26.3, 22.5, 14.0, 9.1, 8.5.

^{19}F NMR: (282 MHz, $CDCl_3$) δ -63.7, -79.7.

Melting point: liquid at room temperature.

Elemental analysis:
Calculated: C: 41.71%, H: 4.45%, N: 6.63%, S: 10.12%.
Found: C: 41.91%, H: 4.60%, N: 6.29%, S: 10.06%.

3-Dodecyl-4,5-dimethyl-1-(4-(trifluoromethyl)phenyl)-1*H*-imidazol-3-ium bis(trifluoromethylsulfonyl)amide (51)

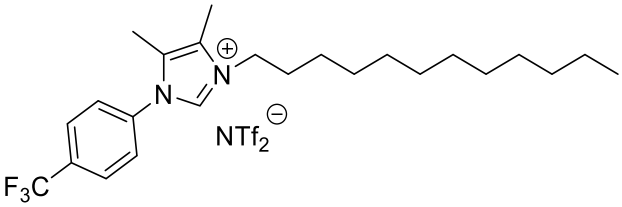

Following the general procedure, 10 mmol of bromide salt **24** were used for the anion exchange. The product was obtained as an orange solid. (6.8 g, 9 mmol, 95%).

^{1}H NMR: (300MHz, $CDCl_3$) δ 8.59 (s, 1 H), 7.84 (d, *J* = 8.3 Hz, 2 H), 7.62 (d, *J* = 8.8 Hz, 2 H), 4.07 - 4.17 (m, 2 H), 2.33 (s, 3 H), 2.17 (s, 3 H), 1.80 - 1.94 (m, 2 H), 1.21 - 1.43 (m, 18 H), 0.81 - 0.93 (m, 3 H).

^{13}C NMR: (75 MHz, $CDCl_3$) δ 135.9, 133.6, 133.3, 132.9, 127.9, 127.5, 127.4, 126.8, 119.9 (q, *J* = 321.9 Hz), 47.9, 31.9, 29.5, 29.4, 29.3, 29.3, 28.9, 26.3, 22.6, 14.0, 9.0, 8.5.

^{19}F NMR: (282 MHz, $CDCl_3$) δ -63.7, -79.7.

Melting point: 40 °C.

Elemental analysis:
Calculated: C: 45.28%, H: 5.26%, N: 6.09%, S: 9.30%.
Found: C: 45.37%, H: 5.32%, N: 5.66%, S: 9.34%.

3-Butyl-4,5-dimethyl-1-(4-(trifluoromethoxy)phenyl)-1*H*-imidazol-3-ium bis(trifluoromethylsulfonyl)amide (52)

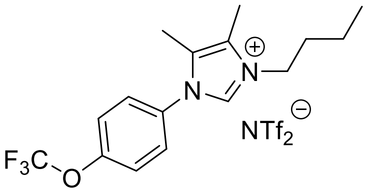

Following the general procedure, 13 mmol of bromide salt **25** were used for the anion exchange. The product was obtained as a light-yellow oil. (7.4 g, 12 mmol, 97%).

^{1}H NMR: (300MHz, $CDCl_3$) δ 8.57 (s, 1 H), 7.37 - 7.57 (m, 4 H), 4.13 (t, *J* = 7.7 Hz, 2 H), 2.33 (s, 3 H), 2.15 (s, 3 H), 1.85 (quin, *J* = 7.6 Hz, 2 H), 1.43 (sxt, *J* = 7.4 Hz, 2 H), 0.98 (t, *J* = 7.3 Hz, 3 H).

^{13}C NMR: (75 MHz, $CDCl_3$) δ 150.7, 133.8, 131.2, 128.1, 128.0, 127.6, 122.4, 118.5, 119.9 (q, *J* = 321.9 Hz), 47.6, 31.3, 19.6, 13.3, 9.0, 8.5.

^{19}F NMR: (282 MHz, $CDCl_3$) δ -58.6, -79.7.

Melting point: liquid at room temperature.

Elemental analysis:

Calculated: C: 36.43%, H: 3.40%, N: 7.08%, S: 10.80%.
Found: C: 36.46%, H: 3.62%, N: 6.84%, S: 10.84%.

4,5-Dimethyl-3-octyl-1-(4-(trifluoromethoxy)phenyl)-1*H*-imidazol-3-ium bis(trifluoromethylsulfonyl)amide (53)

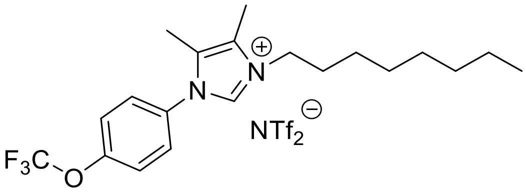

Following the general procedure, 11 mmol of bromide salt **26** were used for the anion exchange. The product was obtained as a light-yellow oil. (6.8 g, 10 mmol, 93%).

^{1}H NMR: (300MHz, $CDCl_3$) δ 8.56 (s, 1 H), 7.37 - 7.57 (m, 4 H), 4.04 - 4.21 (m, 2 H), 2.33 (s, 3 H), 2.16 (s, 3 H), 1.78 - 1.97 (m, 2 H), 1.21 - 1.44 (m, 10 H), 0.81 - 0.94 (m, 3 H).

^{13}C NMR: (75 MHz, $CDCl_3$) δ 150.7, 133.7, 131.2, 128.1, 128.0, 127.6, 122.4, 118.5, 119.9 (q, *J* = 321.9 Hz), 47.8, 31.6, 29.5, 28.9, 28.8, 26.3, 22.5, 14.0, 9.0, 8.5.

^{19}F NMR: (282 MHz, $CDCl_3$) δ -58.6, -79.7.

Melting point: liquid at room temperature.

Elemental analysis:

Calculated: C: 40.68%, H: 4.34%, N: 6.47%, S: 9.87%.
Found: C: 40.44%, H: 4.74%, N: 6.38%, S: 9.91%.

3-Dodecyl-4,5-dimethyl-1-(4-(trifluoromethoxy)phenyl)-1*H*-imidazol-3-ium bis(trifluoromethylsulfonyl)amide (54)

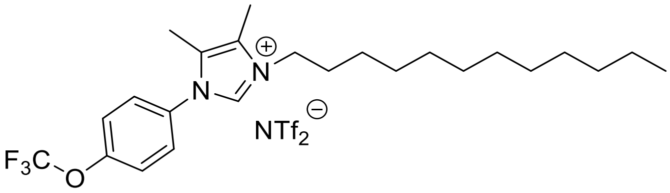

Following the general procedure, 10 mmol of bromide salt **27** were used for the anion exchange. The product was obtained as a light-yellow solid. (6.9 g, 10 mmol, 98%).

^{1}H NMR: (300MHz, $CDCl_3$) δ 8.56 (s, 1 H), 7.38 - 7.55 (m, 4 H), 4.07 - 4.16 (m, 2 H), 2.33 (s, 3 H), 2.15 (s, 3 H), 1.80 - 1.93 (m, 2 H), 1.21 - 1.44 (m, 18 H), 0.83 - 0.91 (m, 3 H).

^{13}C NMR: (75 MHz, $CDCl_3$) δ 150.7, 133.7, 131.2, 128.1, 128.0, 127.6, 122.4, 118.5, 119.9 (q, *J* = 321.9 Hz), 47.8, 31.9, 29.6, 29.5, 29.4, 29.3, 28.9, 26.3, 22.6, 14.0, 8.9, 8.5.

^{19}F NMR: (282 MHz, $CDCl_3$) δ -58.6, -79.7.

Melting point: 38 °C.

Elemental analysis:

Calculated: C: 44.25%, H: 5.14%, N: 5.95%, S: 9.09%.
Found: C: 44.62%, H: 5.14%, N: 5.54%, S: 8.95%.

3-Butyl-4,5-dimethyl-1-(o-tolyl)-1*H*-imidazol-3-ium bis(trifluoromethylsulfonyl)amide (55)

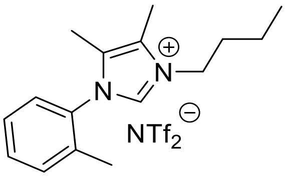

Following the general procedure, 15 mmol of bromide salt **28** were used for the anion exchange. The product was obtained as a light-red oil. (6.3 g, 12 mmol, 80%).

^{1}H NMR: (600MHz, $CDCl_3$) δ 8.50 (s, 1 H), 7.49 - 7.54 (m, 1 H), 7.37 - 7.44 (m, 2 H), 7.30 (d, *J* = 8.1 Hz, 1 H), 4.19 (t, *J* = 7.7 Hz, 2 H), 2.38 (s, 3 H), 2.09 (s, 3 H), 2.03 (s, 3 H), 1.87 (quin, *J* = 7.6 Hz, 2 H), 1.44 (sxt, *J* = 7.5 Hz, 2 H), 1.00 (t, *J* = 7.5 Hz, 3 H).

^{13}C NMR: (151 MHz, $CDCl_3$) δ 134.7, 133.5, 131.9, 131.6, 131.3, 128.3, 127.6, 127.3, 127.2, 119.7 (q, *J* = 321.4 Hz), 47.4, 31.3, 19.4, 16.8, 13.2, 8.4, 8.4.

^{19}F NMR: (565 MHz, $CDCl_3$) δ -79.1.

Melting point: liquid at room temperature.

Elemental analysis:

Calculated: C: 41.30%, H: 4.43%, N: 8.03%, S: 12.25%.
Found: C: 41.65%, H: 4.47%, N: 7.61%, S: 12.16%.

4,5-Dimethyl-3-octyl-1-(*o*-tolyl)-1*H*-imidazol-3-ium bis(trifluoromethylsulfonyl)amide (56)

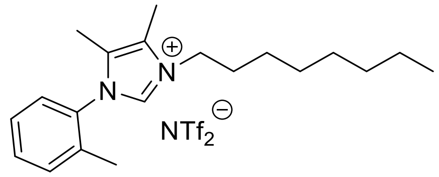

Following the general procedure, 14 mmol of bromide salt **29** were used for the anion exchange. The product was obtained as a light-red oil. (5.4 g, 9 mmol, 67%).

^{1}H NMR: (600MHz, $CDCl_3$) δ 8.48 (s, 1 H), 7.47 - 7.51 (m, 1 H), 7.35 - 7.42 (m, 2 H), 7.26 - 7.29 (m, 1 H), 4.16 (t, *J* = 7.7 Hz, 2 H), 2.35 (s, 3 H), 2.07 (s, 3 H), 2.01 (s, 3 H), 1.86 (quin, *J* = 7.4 Hz, 2 H), 1.22 - 1.41 (m, 10 H), 0.87 (t, *J* = 7.0 Hz, 3 H).

^{13}C NMR: (151 MHz, $CDCl_3$) δ 134.7, 133.5, 131.9, 131.6, 131.3, 128.4, 127.6, 127.3, 127.2, 119.7 (q, *J* = 321.4 Hz), 47.6, 31.5, 29.4, 28.9, 28.7, 26.1, 22.4, 16.8, 13.9, 8.4, 8.4.

^{19}F NMR: (565 MHz, $CDCl_3$) δ -79.1.

Melting point: liquid at room temperature.

Elemental analysis:

Calculated: C: 45.59%, H: 5.39%, N: 7.25%, S: 11.06%.
Found: C: 45.88%, H: 5.09%, N: 6.83%, S: 11.00%.

3-Dodecyl-4,5-dimethyl-1-(*o*-tolyl)-1*H*-imidazol-3-ium bis(trifluoromethylsulfonyl)amide (57)

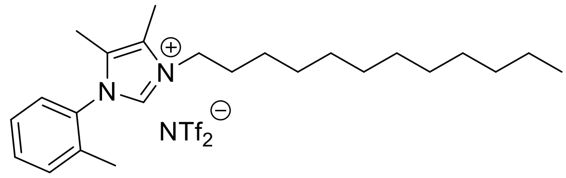

Following the general procedure, 13 mmol of bromide salt **30** were used for the anion exchange. The product was obtained as a light-red oil. (6.4 g, 10 mmol, 77%).

^{1}H NMR: (600MHz, $CDCl_3$) δ 8.51 (s, 1 H), 7.50 - 7.54 (m, 1 H), 7.38 - 7.45 (m, 2 H), 7.29 - 7.33 (m, 1 H), 4.19 (t, *J* = 7.5 Hz, 2 H), 2.38 (s, 3 H), 2.10 (s, 3 H), 2.04 (s, 3 H), 1.89 (quin, *J* = 7.3 Hz, 2 H), 1.24 - 1.43 (m, 18 H), 0.90 (t, *J* = 7.2 Hz, 3 H).

^{13}C NMR: (151 MHz, $CDCl_3$) δ 134.7, 133.6, 131.9, 131.6, 131.3, 128.4, 127.7, 127.3, 119.7 (q, *J* = 321.4 Hz), 47.6, 31.8, 29.5, 29.4, 29.3, 29.2, 28.8, 26.1, 22.6, 16.8, 14.0, 8.5, 8.4.

^{19}F NMR: (565 MHz, $CDCl_3$) δ -79.0.

Melting point: liquid at room temperature.

Elemental analysis:

Calculated: C: 49.12%, H: 6.18%, N: 6.61%, S: 10.09%.
Found: C: 49.35%, H: 5.97%, N: 6.33%, S: 10.08%.

3-Butyl-1-(2-methoxyphenyl)-4,5-dimethyl-1*H*-imidazol-3-ium bis(trifluoromethylsulfonyl)amide (58)

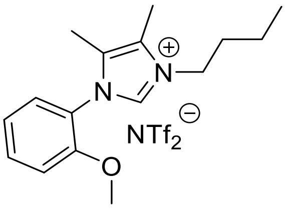

Following the general procedure, 15 mmol of bromide salt **31** were used for the anion exchange. The product was obtained as a brown oil. (7.5 g, 14 mmol, 93%).

^{1}H NMR: (300MHz, $CDCl_3$) δ 8.42 (s, 1 H), 7.50 - 7.59 (m, 1 H), 7.30 - 7.35 (m, 1 H), 7.04 - 7.14 (m, 2 H), 4.09 - 4.20 (m, 2 H), 3.81 (s, 3 H), 2.32 (s, 3 H), 2.03 (s, 3 H), 1.76 - 1.91 (m, 2 H), 1.40 (m, 2 H), 0.92 - 1.03 (m, 3 H).

^{13}C NMR: (75 MHz, $CDCl_3$) δ 153.8, 134.2, 132.7, 129.0, 128.0, 126.5, 121.9, 121.2, 119.7 (q, *J* = 321.4 Hz), 112.3, 55.9, 47.3, 31.4, 19.4, 13.3, 8.5, 8.4.

^{19}F NMR: (282 MHz, $CDCl_3$) δ -79.0.

Melting point: liquid at room temperature.

Elemental analysis:

Calculated: C: 40.07%, H: 4.30%, N: 7.79%, S: 11.88%.
Found: C: 39.81%, H: 4.58%, N: 7.95%, S: 11.95%.

1-(2-Methoxyphenyl)-4,5-dimethyl-3-octyl-1*H*-imidazol-3-ium bis(trifluoromethylsulfonyl)amide (59)

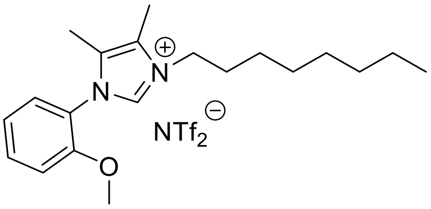

Following the general procedure, 15 mmol of bromide salt **32** were used for the anion exchange. The product was obtained as a brown oil. (6.4 g, 11 mmol, 72%).

^{1}H NMR: (300MHz, $CDCl_3$) δ 8.43 (s, 1 H), 7.49 - 7.58 (m, 1 H), 7.30 - 7.36 (m, 1 H), 7.06 - 7.14 (m, 2 H), 4.09 - 4.19 (m, 2 H), 3.82 (s, 3 H), 2.32 (s, 3 H), 2.03 (s, 3 H), 1.85 (quin, *J* = 7.4 Hz, 2 H), 1.21 - 1.41 (m, 10 H), 0.81 - 0.90 (m, 3 H).

^{13}C NMR: (75 MHz, $CDCl_3$) δ 153.8, 134.3, 132.7, 129.0, 128.0, 126.4, 121.3, 121.3, 119.7 (q, *J* = 321.4 Hz), 112.3, 55.9, 47.5, 31.6, 29.5, 28.9, 28.8, 26.1, 22.5, 13.9, 8.5, 8.4.

^{19}F NMR: (282 MHz, $CDCl_3$) δ -79.0.

Melting point: liquid at room temperature.

Elemental analysis:
Calculated: C: 44.36%, H: 5.25%, N: 7.06%, S: 10.77%.
Found: C: 44.68%, H: 5.23%, N: 6.71%, S: 10.84%.

3-Dodecyl-1-(2-methoxyphenyl)-4,5-dimethyl-1*H*-imidazol-3-ium bis(trifluoromethylsulfonyl)amide (60)

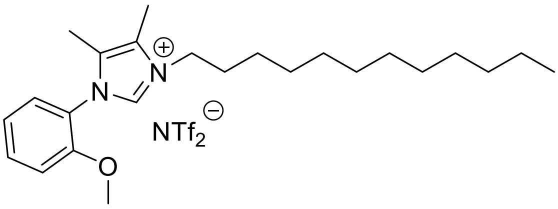

Following the general procedure, 12 mmol of bromide salt **33** were used for the anion exchange. The product was obtained as a brown oil. (6.1 g, 9 mmol, 77%).

^{1}H NMR: (300MHz, $CDCl_3$) δ 8.42 (s, 1 H), 7.50 - 7.57 (m, 1 H), 7.30 - 7.36 (m, 1 H), 7.05 - 7.13 (m, 2 H), 4.09 - 4.18 (m, 2 H), 3.82 (s, 3 H), 2.32 (s, 3 H), 2.03 (s, 3 H), 1.78 - 1.91 (m, 2 H), 1.14 - 1.43 (m, 18 H), 0.81 - 0.91 (m, 3 H).

^{13}C NMR: (75 MHz, $CDCl_3$) δ 153.8, 134.2, 132.7, 129.0, 128.0, 126.4, 121.3, 121.3, 119.7 (q, *J* = 321.4 Hz), 112.3, 55.9, 47.5, 31.8, 29.5, 29.4, 29.3, 29.2, 28.9, 26.1, 22.6, 14.0, 8.5, 8.4.

^{19}F NMR: (282 MHz, $CDCl_3$) δ -79.0.

Melting point: liquid at room temperature.

Elemental analysis:

Calculated: C: 47.92%, H: 6.03%, N: 6.45%, S: 9.84%.
Found: C: 47.71%, H: 6.02%, N: 6.10%, S: 9.92%.

3-Butyl-1-(2,4-difluorophenyl)-4,5-dimethyl-1*H*-imidazol-3-ium bis(trifluoromethylsulfonyl)amide (61)

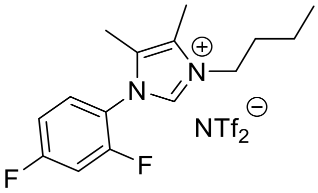

Following the general procedure, 18 mmol of bromide salt **34** were used for the anion exchange. The product was obtained as a brown oil. (7.8 g, 14 mmol, 79%).

^{1}H NMR: (300MHz, $CDCl_3$) δ 8.65 (s, 1 H), 7.57 - 7.72 (m, 1 H), 7.03 - 7.19 (m, 2 H), 4.10 - 4.24 (m, 2 H), 2.35 (s, 3 H), 2.11 (s, 3 H), 1.86 (quin, *J* = 7.6 Hz, 2 H), 1.32 - 1.52 (m, 2 H), 0.92 - 1.06 (m, 3 H).

^{13}C NMR: (75 MHz, $CDCl_3$) δ 164.2 (dd, *J* = 256.3, 10.7 Hz), 156.9 (dd, 256.3, 12.5 Hz), 134.8, 130.5, 130.4, 128.9, 127.4, 119.9 (q, *J* = 321.3 Hz), 113.1 (dd, *J* = 22.7, 3.6 Hz), 105.6 (dd, *J* = 26.8, 4.2 Hz), 47.7, 31.3, 19.5, 13.3, 8.6, 8.5.

^{19}F NMR: (282 MHz, $CDCl_3$) δ -79.0, -102.4, -117.0.

Melting point: liquid at room temperature.

Elemental analysis:
Calculated: C: 37.43%, H: 3.51%, N: 7.70%, S: 11.76%.
Found: C: 37.80%, H: 3.65%, N: 7.98%, S: 11.45%.

1-(2,4-Difluorophenyl)-4,5-dimethyl-3-octyl-1*H*-imidazol-3-ium bis(trifluoromethylsulfonyl)amide (62)

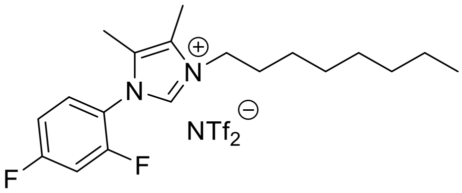

Following the general procedure, 15 mmol of bromide salt **35** were used for the anion exchange. The product was obtained as a brown oil. (8.7 g, 14 mmol, 96%).

^{1}H NMR: (300MHz, $CDCl_3$) δ 8.64 (s, 1 H), 7.59 - 7.72 (m, 1 H), 7.03 - 7.19 (m, 2 H), 4.16 (t, *J* = 7.6 Hz, 2 H), 2.35 (s, 3 H), 2.11 (s, 3 H), 1.82 - 1.94 (m, 2 H), 1.25 - 1.38 (m, 10 H), 0.80 - 0.92 (m, 3 H).

^{13}C NMR: (75 MHz, $CDCl_3$) δ 164.2 (dd, *J* = 256.3, 10.7 Hz), 156.9 (dd, 256.3, 12.5 Hz), 134.8, 130.5, 130.4, 128.9, 127.4, 119.9 (q, *J* = 321.3 Hz), 113.1 (dd, *J* = 22.7, 3.6 Hz), 105.6 (dd, *J* = 26.8, 4.2 Hz), 48.0, 31.6, 29.4, 28.9, 28.8, 26.2, 22.5, 14.0, 8.6, 8.5.

^{19}F NMR: (282 MHz, $CDCl_3$) δ -79.0, -102.4, -117.0.

Melting point: liquid at room temperature.

Elemental analysis:
Calculated: C: 41.93%, H: 4.52%, N: 6.99%, S: 10.66%.
Found: C: 41.69%, H: 4.60%, N: 6.60%, S: 10.52%.

1-(2,4-Difluorophenyl)-3-dodecyl-4,5-dimethyl-1*H*-imidazol-3-ium bis(trifluoromethylsulfonyl)amide (63)

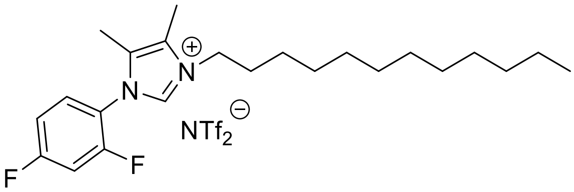

Following the general procedure, 13 mmol of bromide salt **36** were used for the anion exchange. The product was obtained as a brown oil. (7.1 g, 11 mmol, 83%).

^{1}H NMR: (300MHz, $CDCl_3$) δ 8.64 (s, 1 H), 7.60 - 7.73 (m, 1 H), 7.02 - 7.20 (m, 2 H), 4.16 (t, *J* = 7.6 Hz, 2 H), 2.35 (s, 3 H), 2.12 (s, 3 H), 1.81 - 1.95 (m, 2 H), 1.23 - 1.37 (m, 18 H), 0.83 - 0.93 (m, 3 H).

^{13}C NMR: (75 MHz, $CDCl_3$) δ 164.2 (dd, *J* = 256.3, 10.7 Hz), 156.9 (dd, 256.3, 12.5 Hz), 134.8, 130.5, 130.4, 128.9, 127.4, 119.9 (q, *J* = 321.3 Hz), 113.1 (dd, *J* = 22.7, 3.6 Hz), 105.6 (dd, *J* = 26.8, 4.2 Hz), 48.0, 31.9, 29.6, 29.5, 29.5, 29.4, 29.3, 28.9, 26.3, 22.7, 14.1, 8.6, 8.5.

^{19}F NMR: (282 MHz, $CDCl_3$) δ -79.0, -102.3, -117.1.

Melting point: liquid at room temperature.

Elemental analysis:
Calculated: C: 45.66%, H: 5.36%, N: 6.39%, S: 9.75%.
Found: C: 45.75%, H: 5.55%, N: 6.02%, S: 9.82%.

S4 Electrochemical measurements

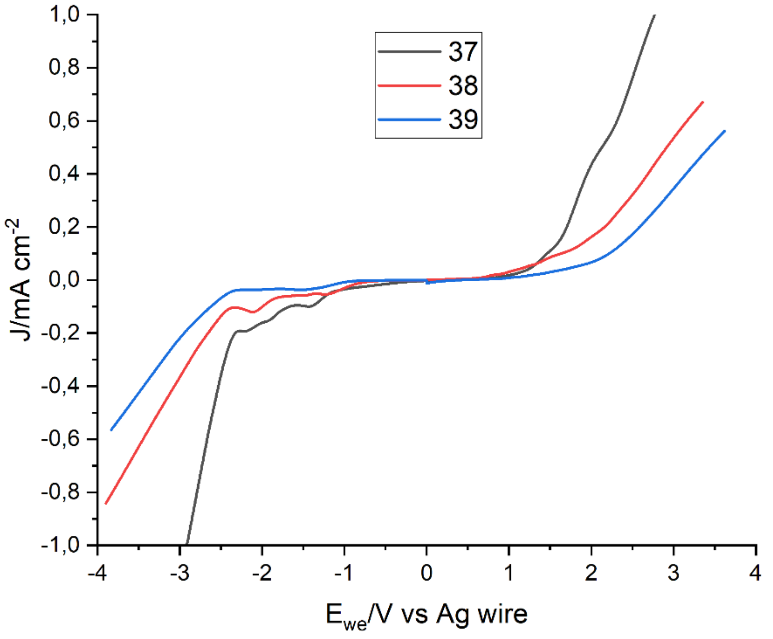

Figure S1. LSV of compounds **37–39**.

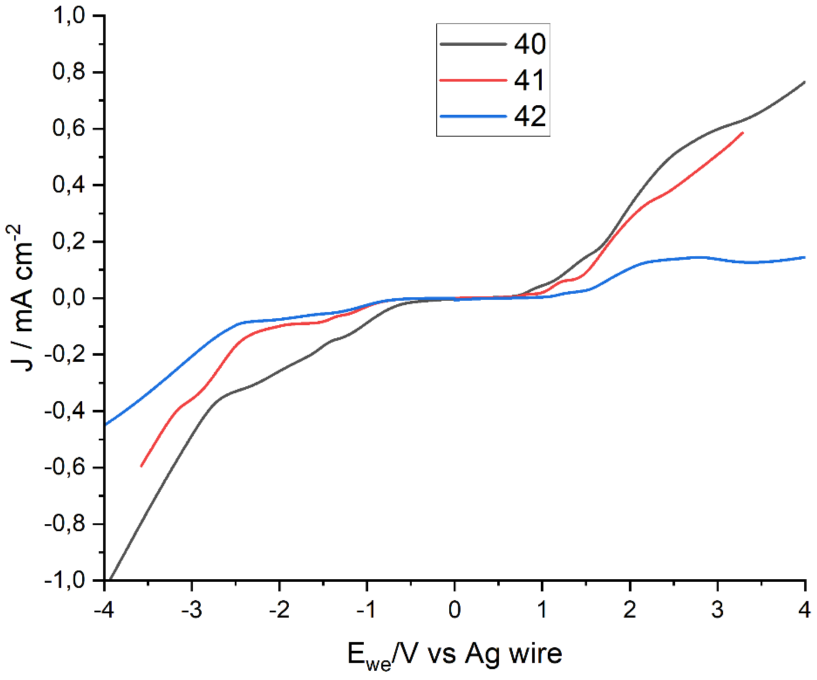

Figure S2. LSV of compounds **40–42**.

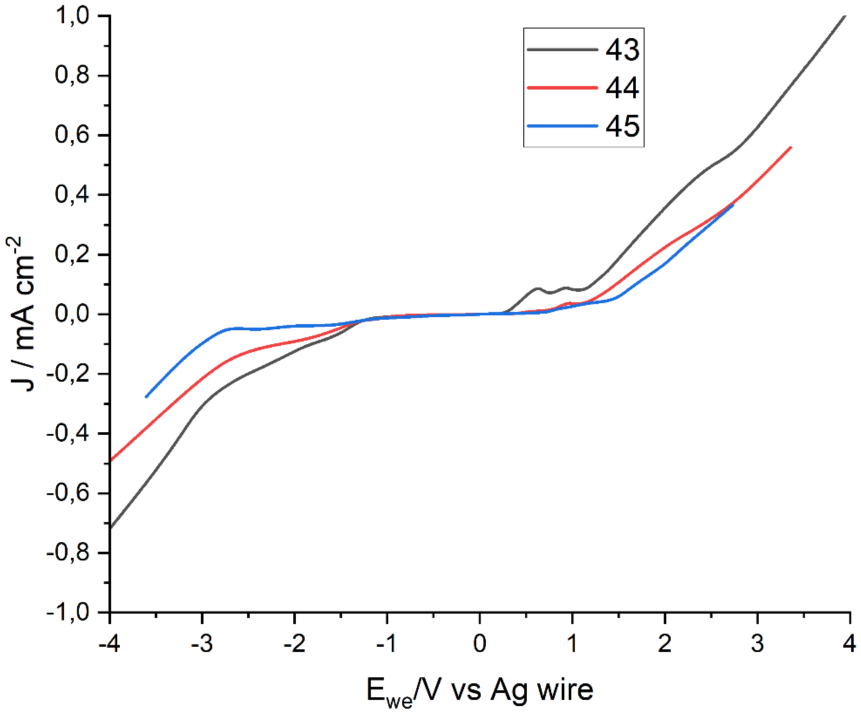

Figure S3. LSV of compounds **43–45**.

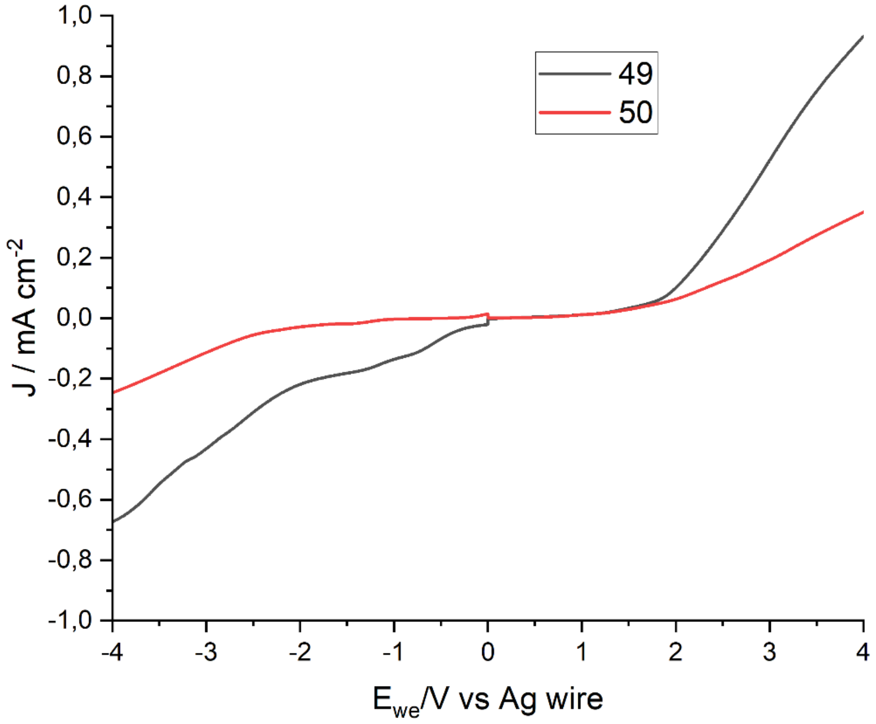

Figure S4. LSV of compounds **49** and **50**.

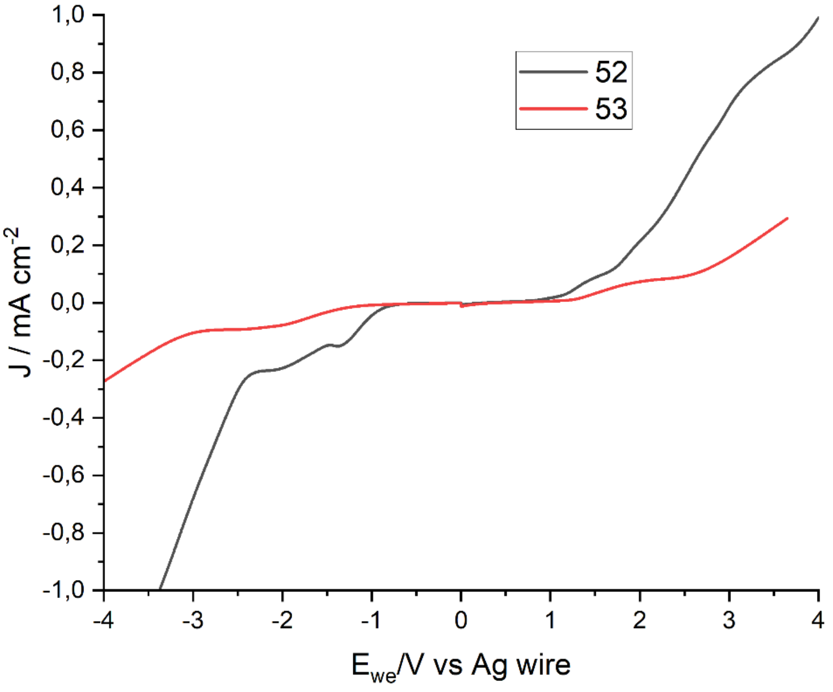

Figure S5. LSV of compounds **52** and **53**.

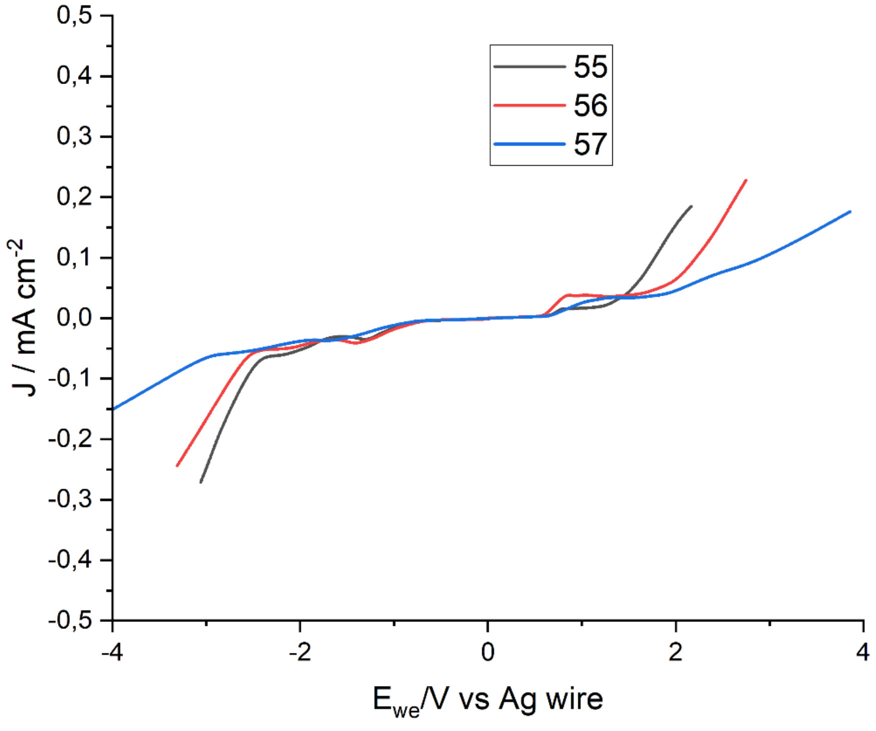

Figure S6. LSV of compounds **55–57**.

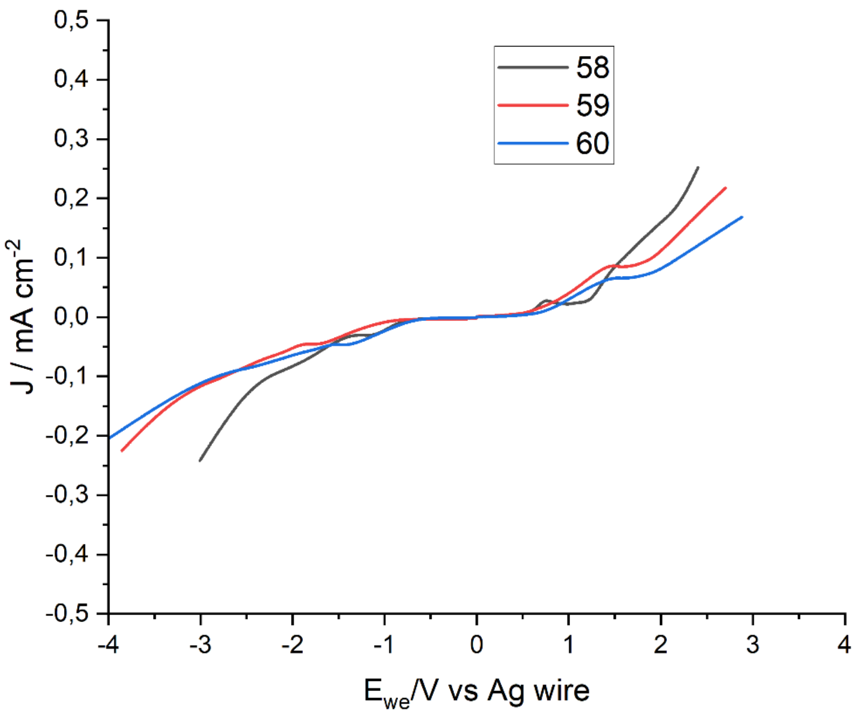

Figure S7. LSV of compounds **58–60**.

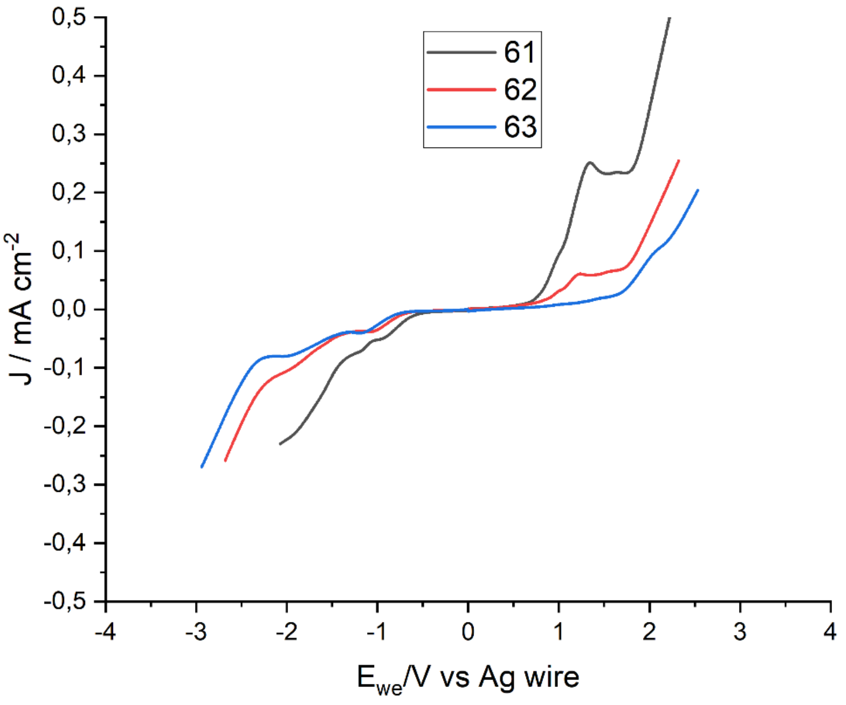

Figure S8. LSV of compounds **61–63**.

S5 Viscosity measurements

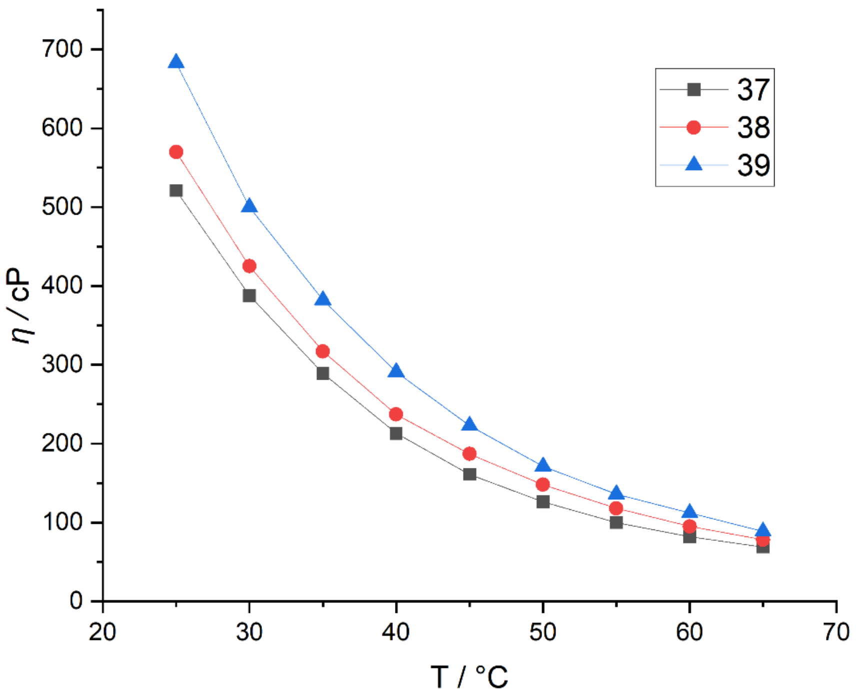

Figure S9. Viscosity measurements of compounds **37–39**.

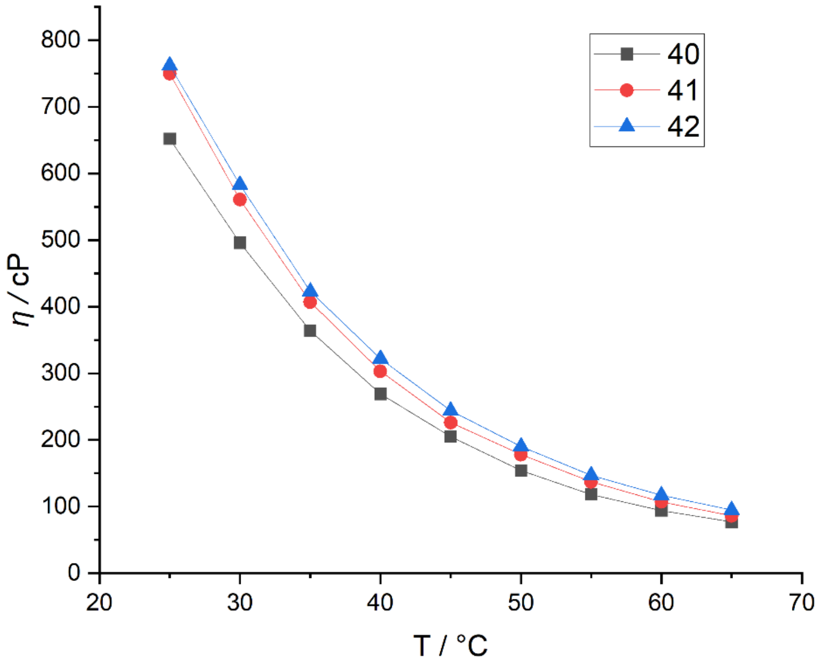

Figure S10. Viscosity measurements of compounds **40–42**.

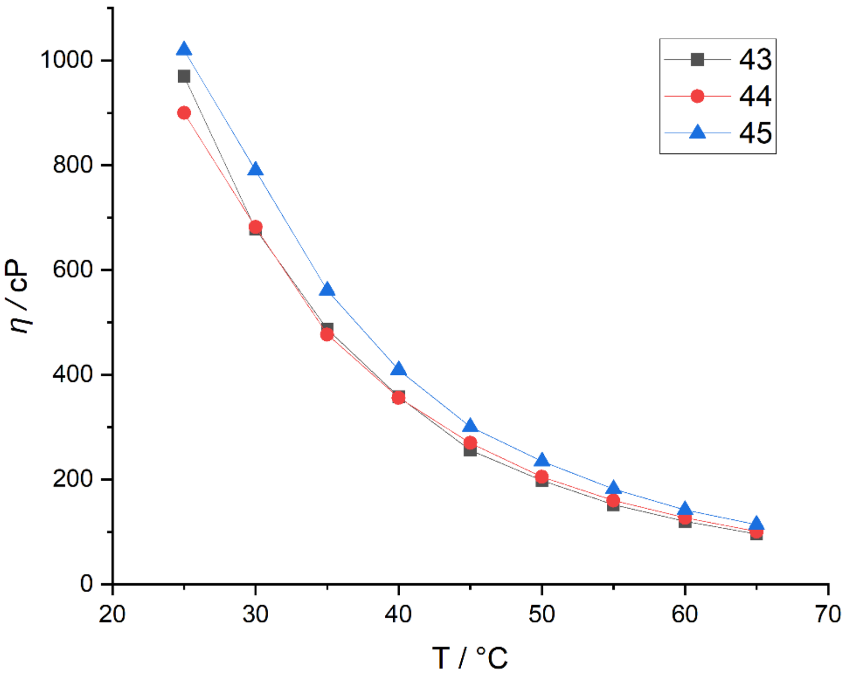

Figure S11. Viscosity measurements of compounds **43–45**.

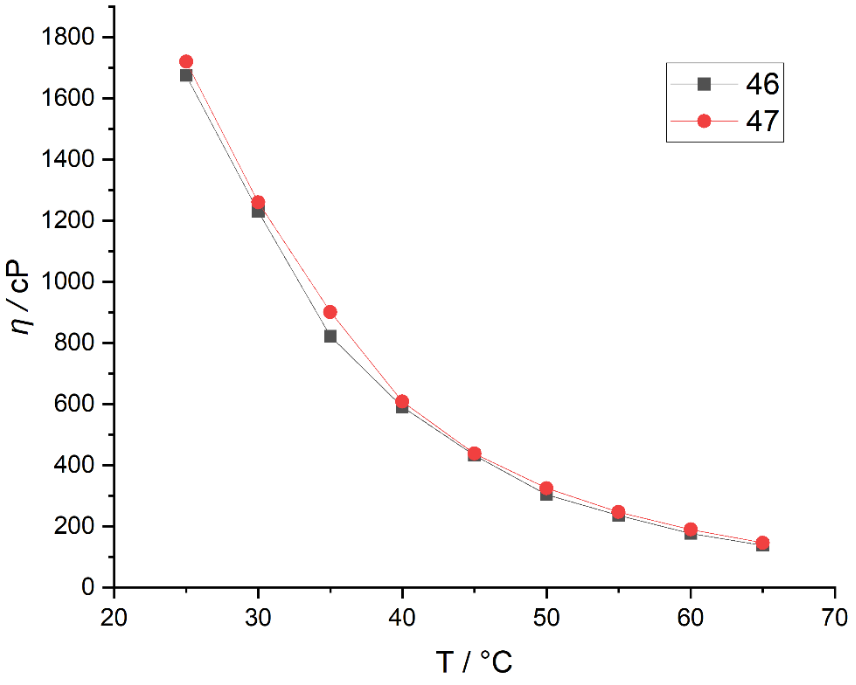

Figure S12. Viscosity measurements of compounds **46** and **47**.

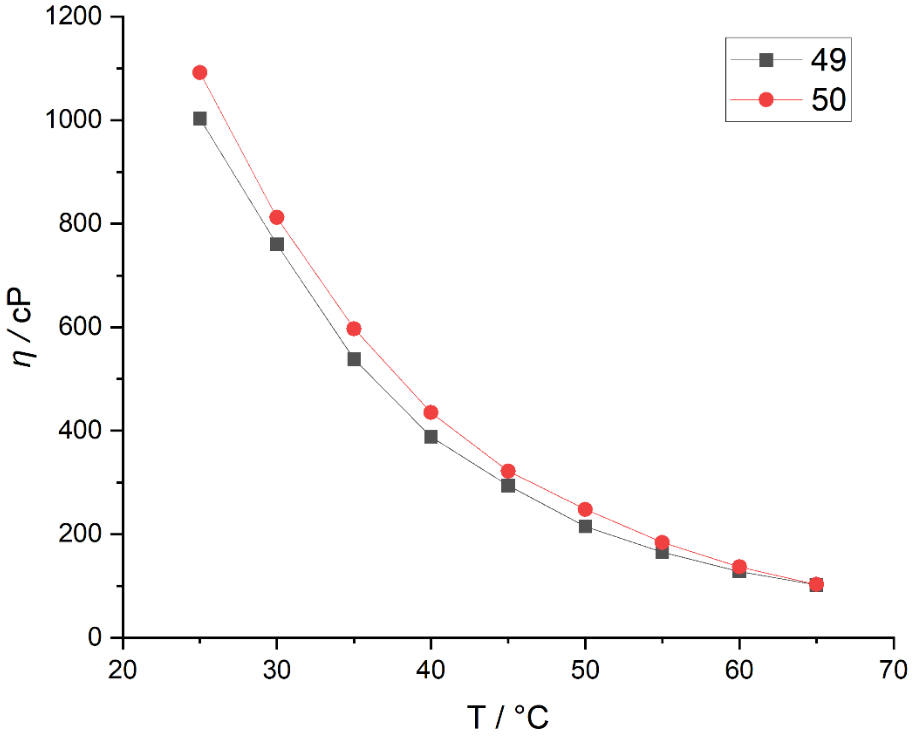

Figure S13. Viscosity measurements of compounds **49** and **50**.

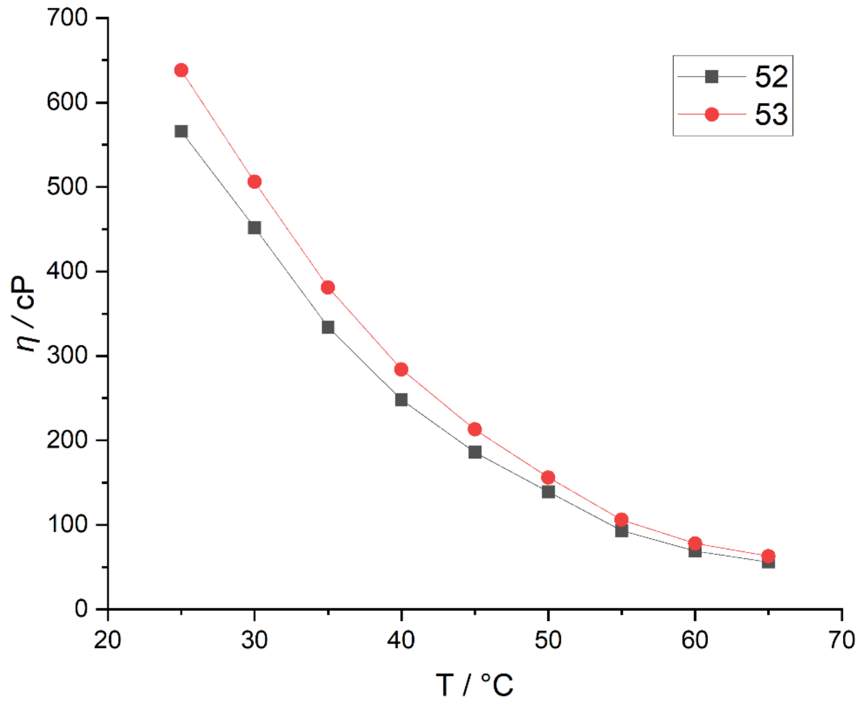

Figure S14. Viscosity measurements of compounds **52** and **53**.

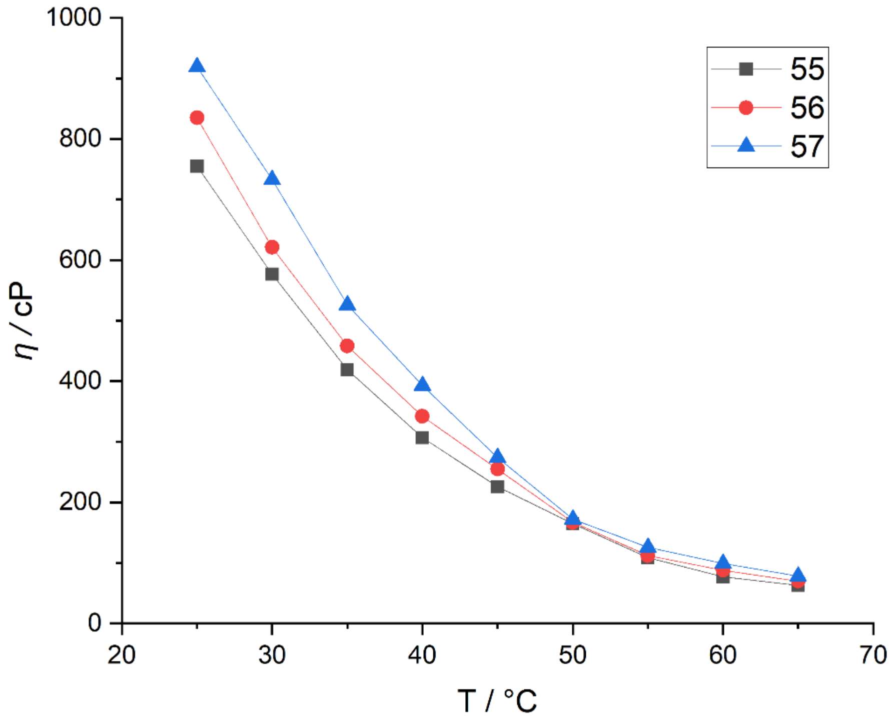

Figure S15. Viscosity measurements of compounds **55–57**.

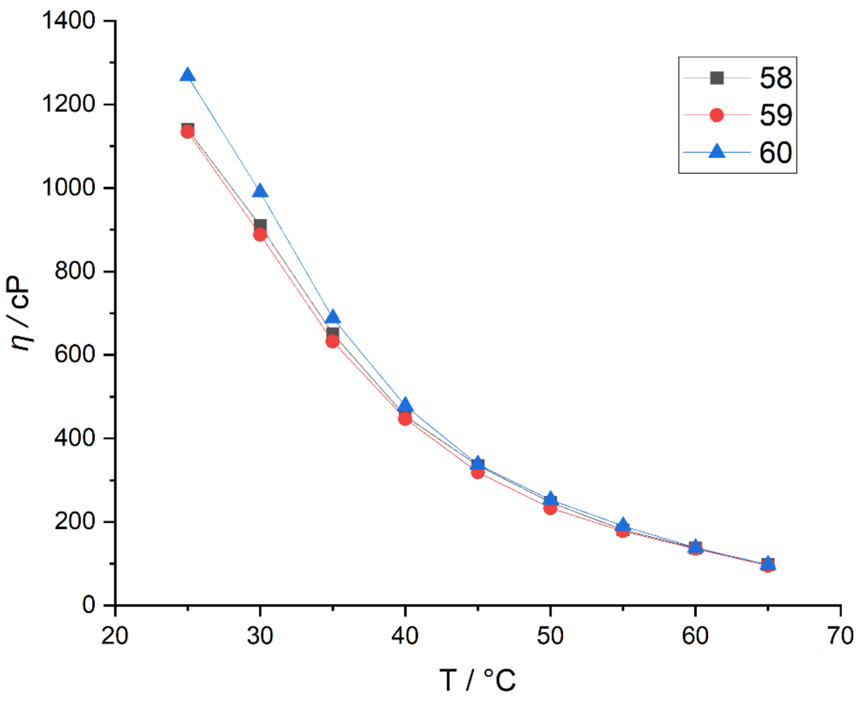

Figure S16. Viscosity measurements of compounds **58–60**.

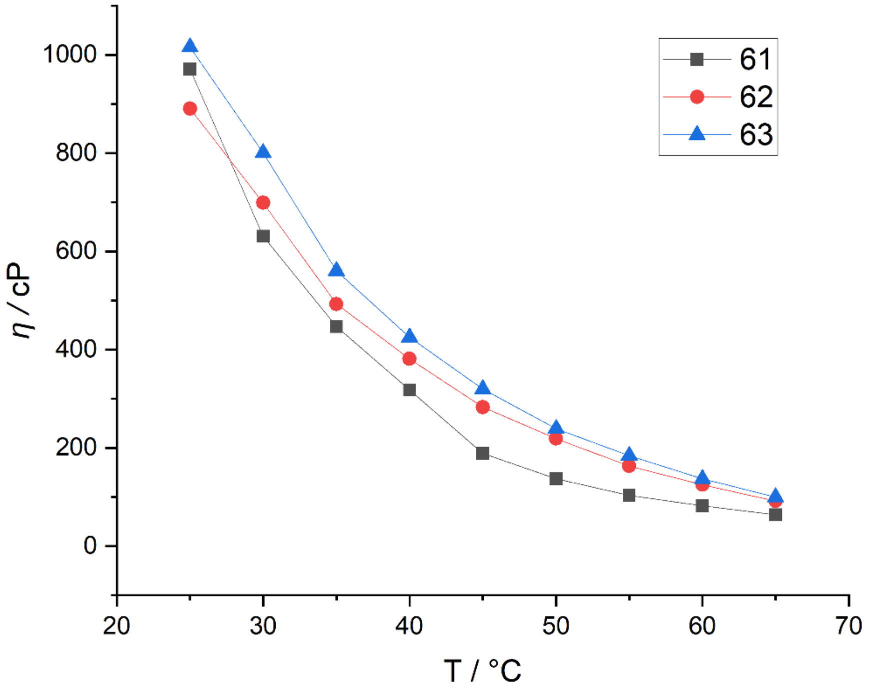

Figure S17. Viscosity measurements of compounds **61–63**.

S6 Thermogravimetric analysis

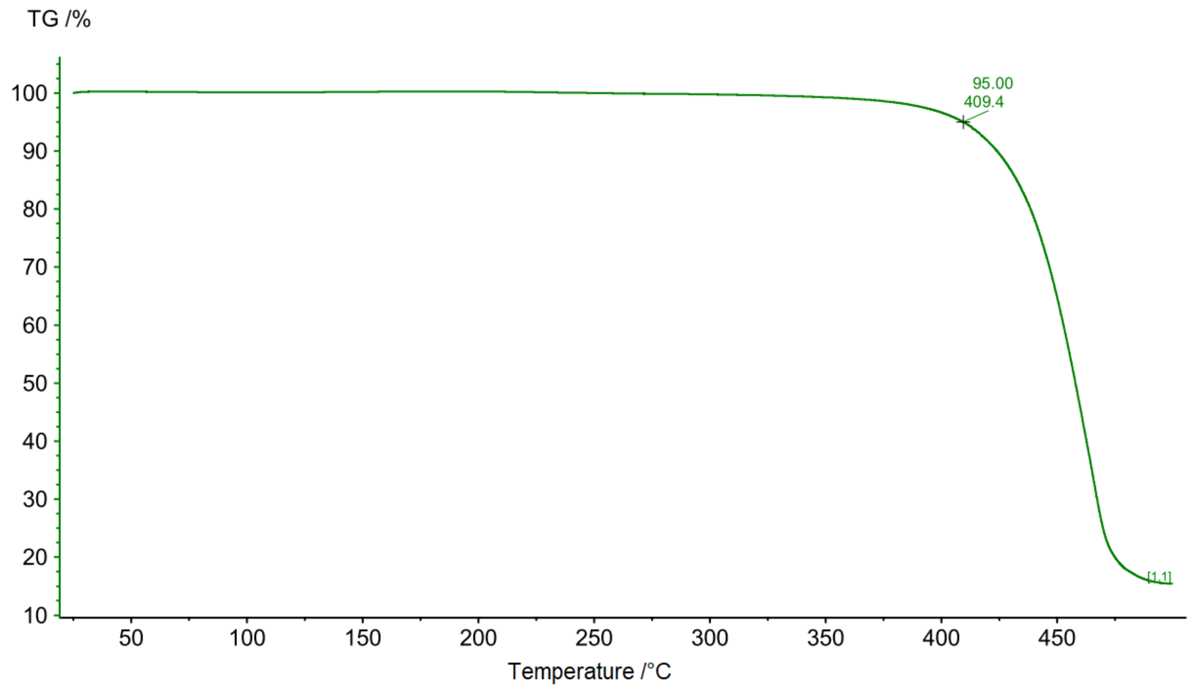

Figure S18. Thermogravimetric analysis of compound **37**.

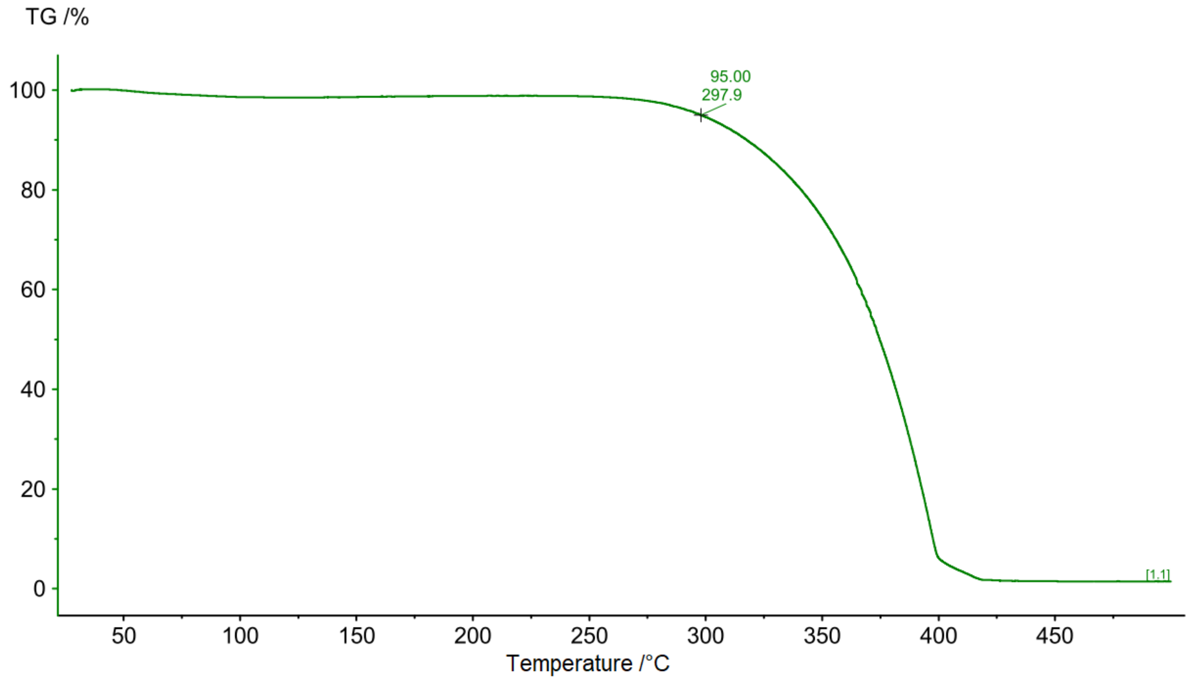

Figure S19. Thermogravimetric analysis of compound **38**.

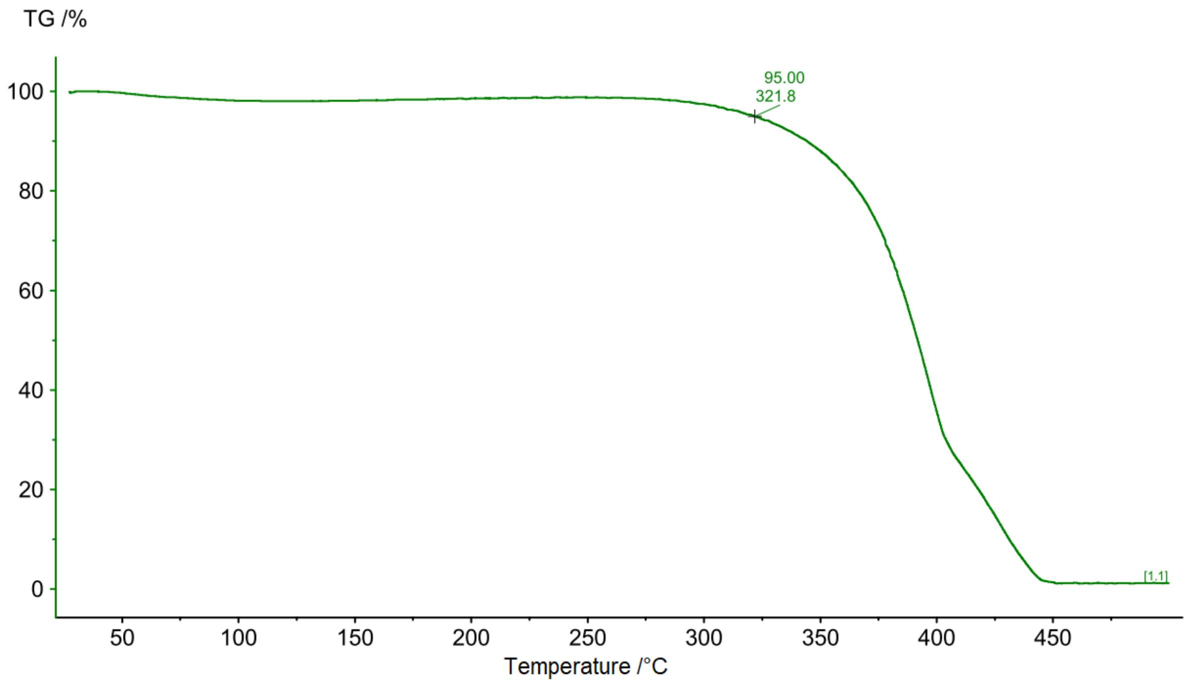

Figure S20. Thermogravimetric analysis of compound **39**.

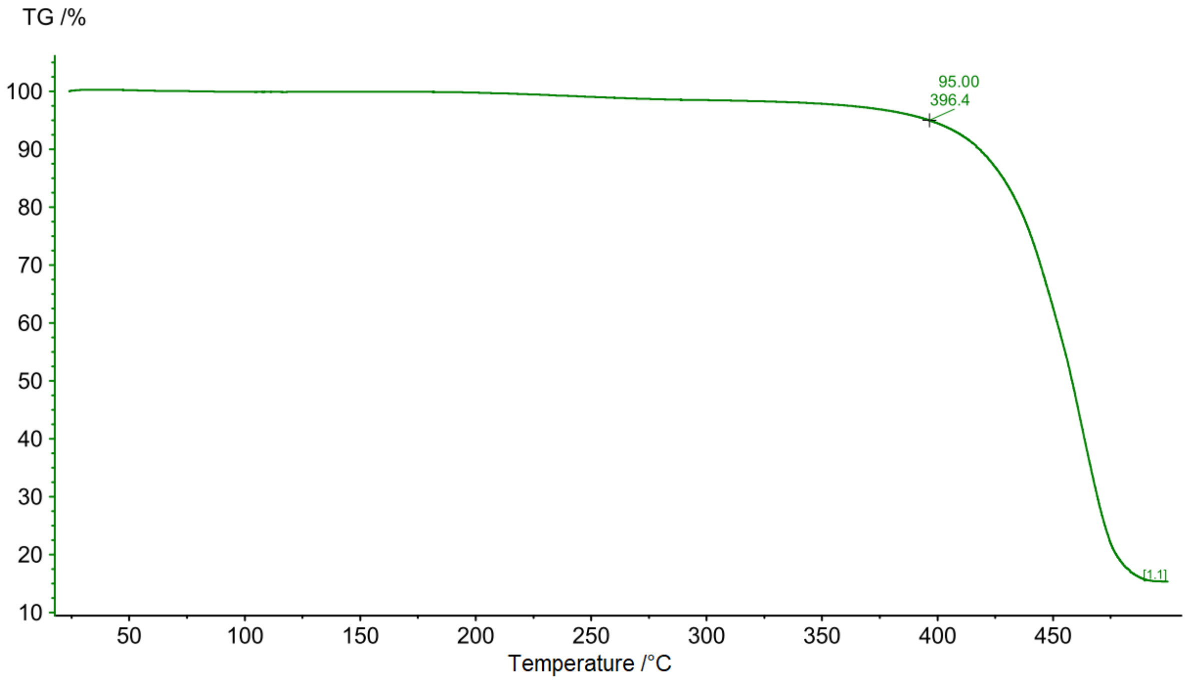

Figure S21. Thermogravimetric analysis of compound **40**.

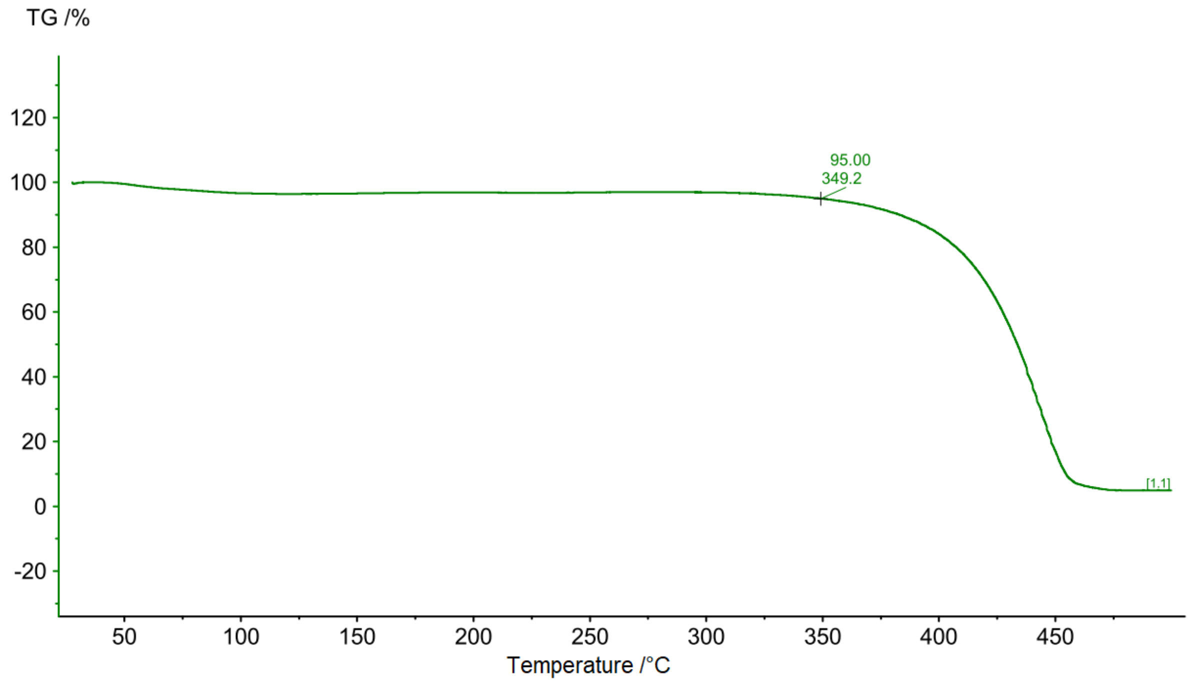

Figure S22. Thermogravimetric analysis of compound **41**.

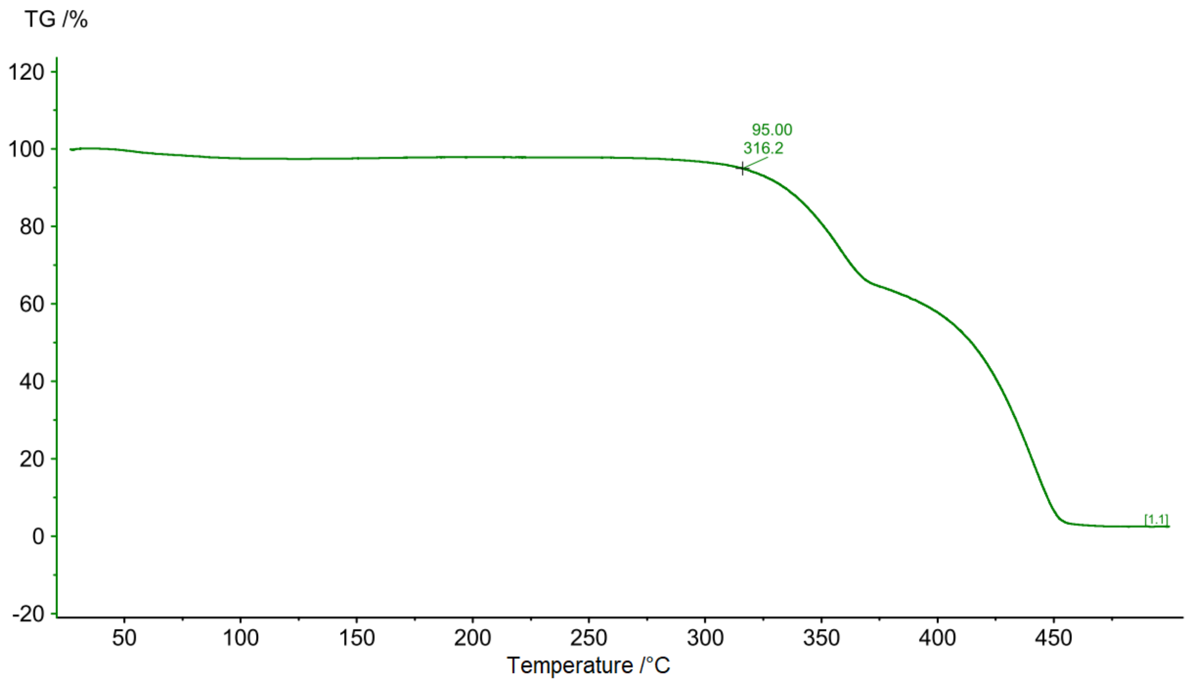

Figure S23. Thermogravimetric analysis of compound **42**.

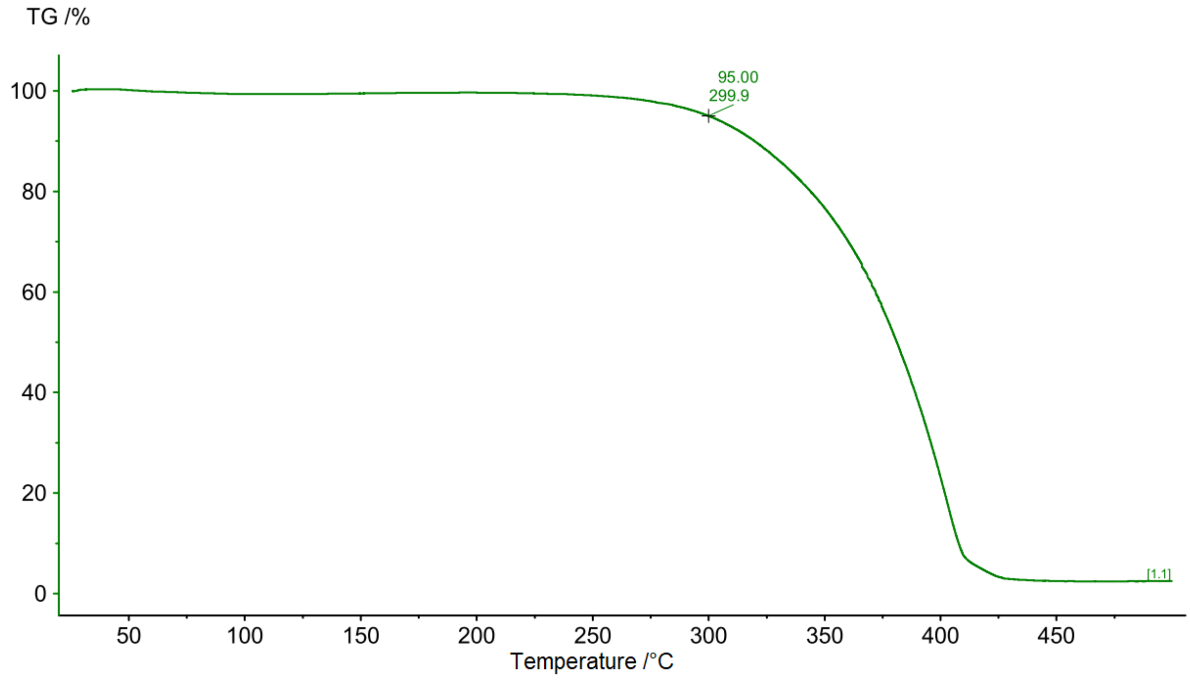

Figure S24. Thermogravimetric analysis of compound **43**.

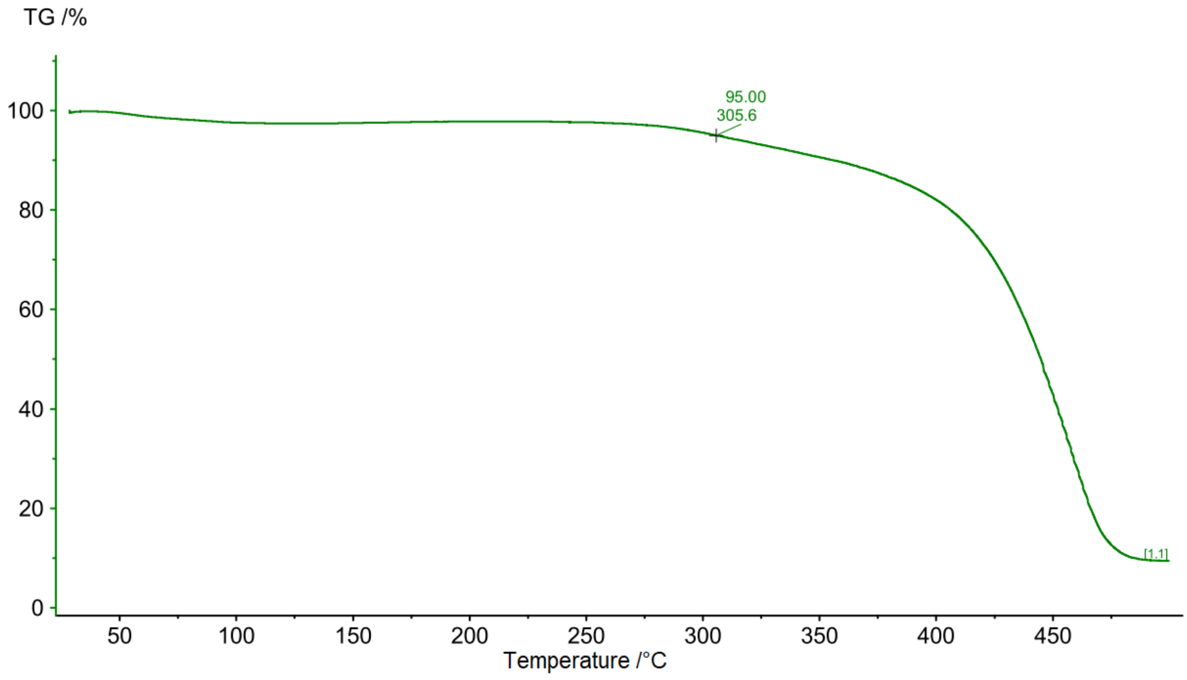

Figure S25. Thermogravimetric analysis of compound **44**.

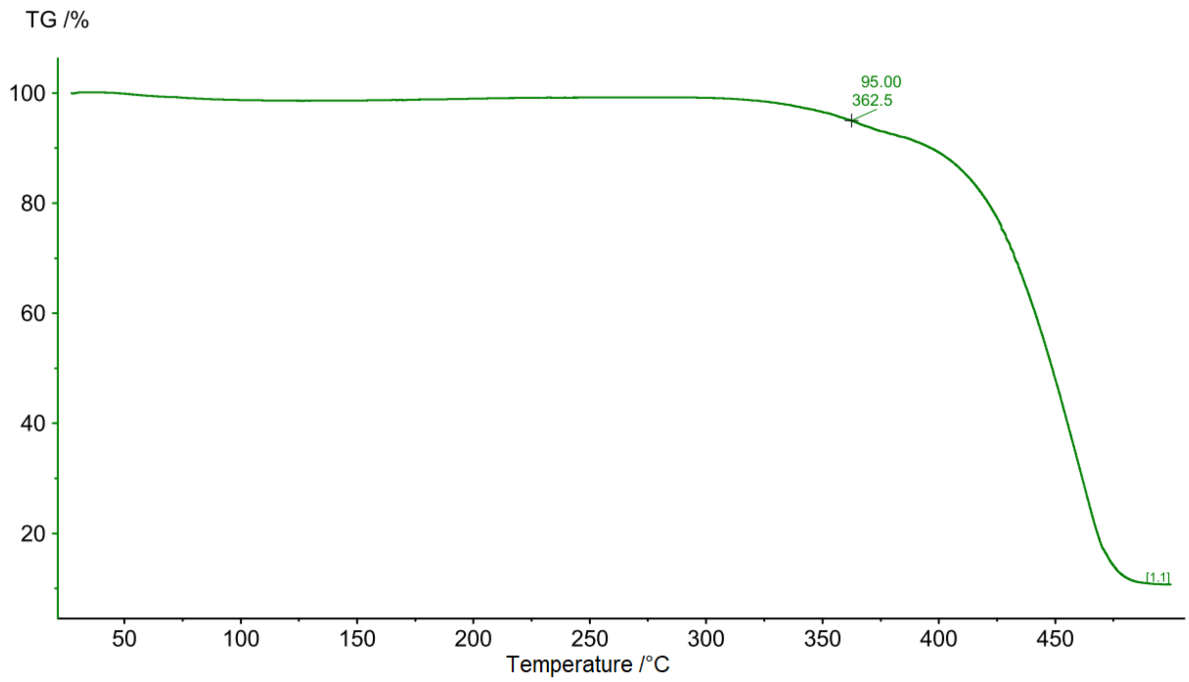

Figure S26. Thermogravimetric analysis of compound **45**.

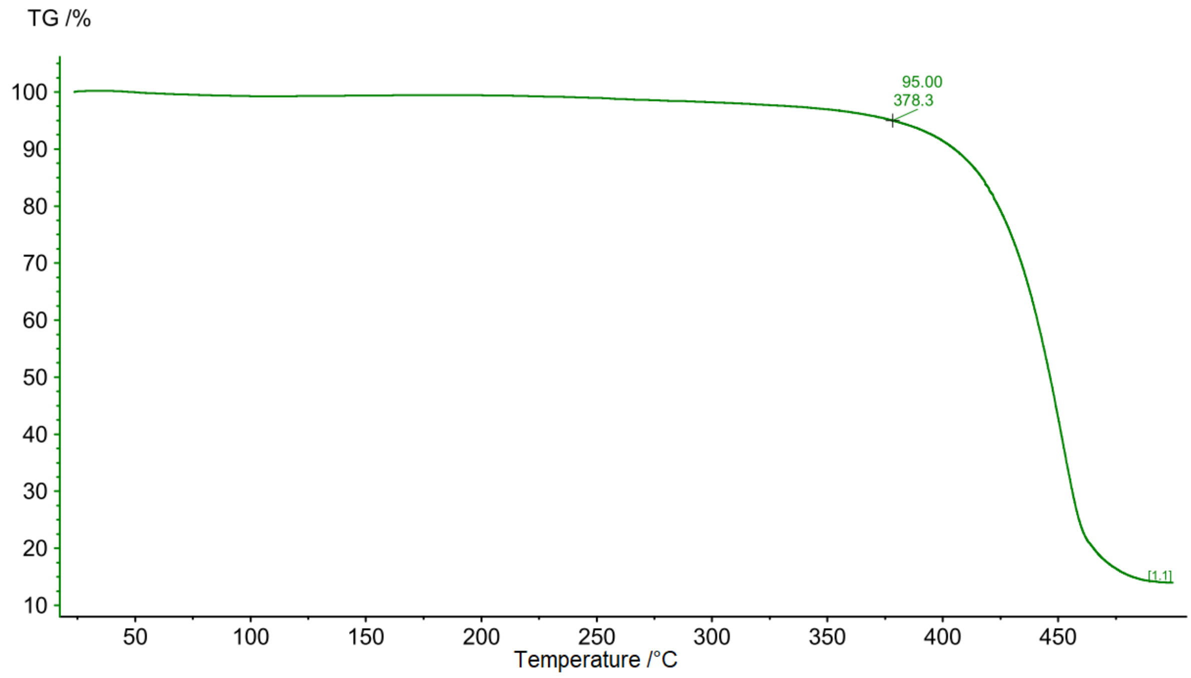

Figure S27. Thermogravimetric analysis of compound **46**.

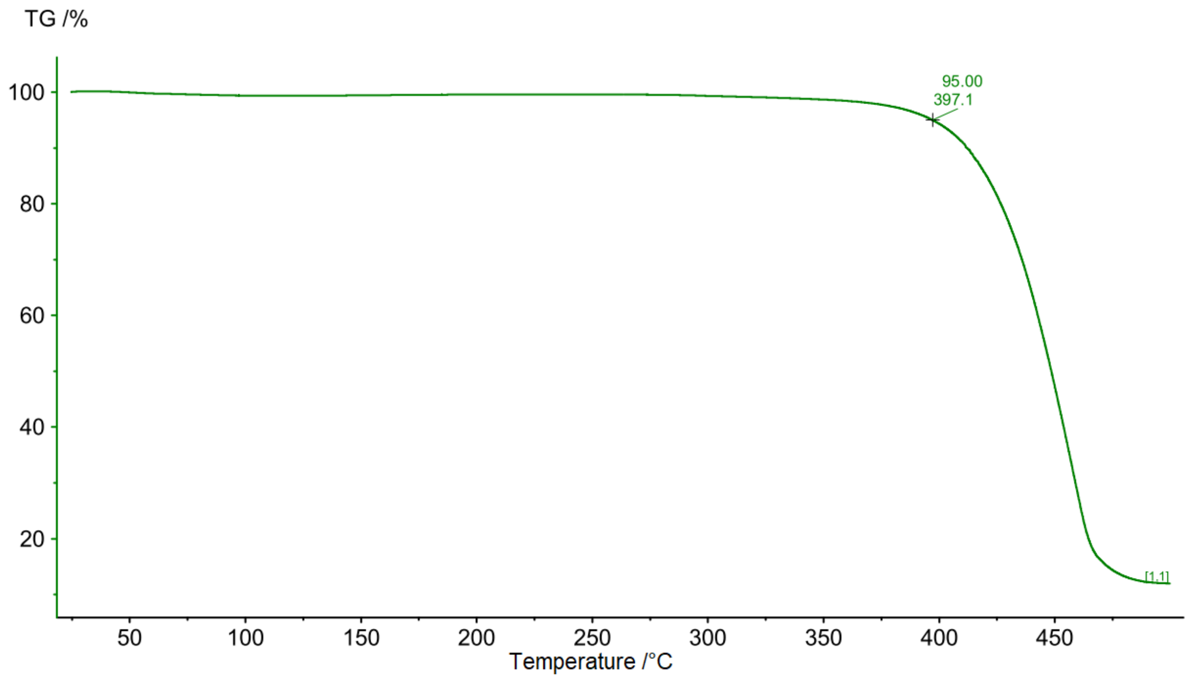

Figure S28. Thermogravimetric analysis of compound **47**.

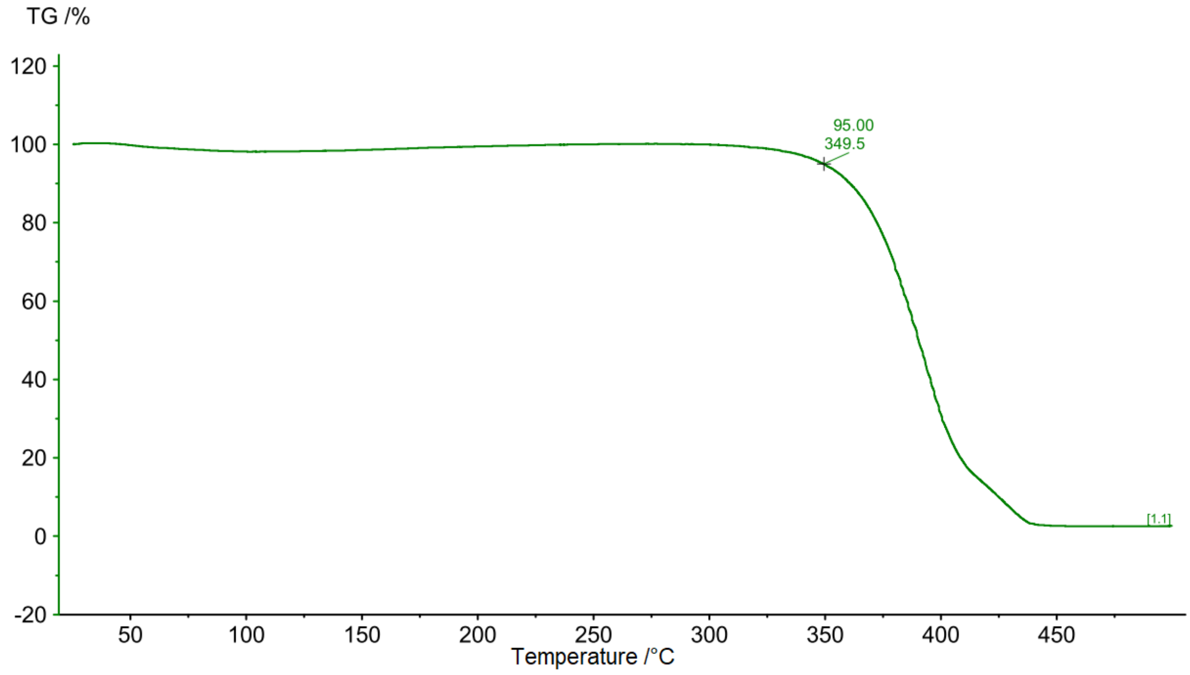

Figure S29. Thermogravimetric analysis of compound **48**.

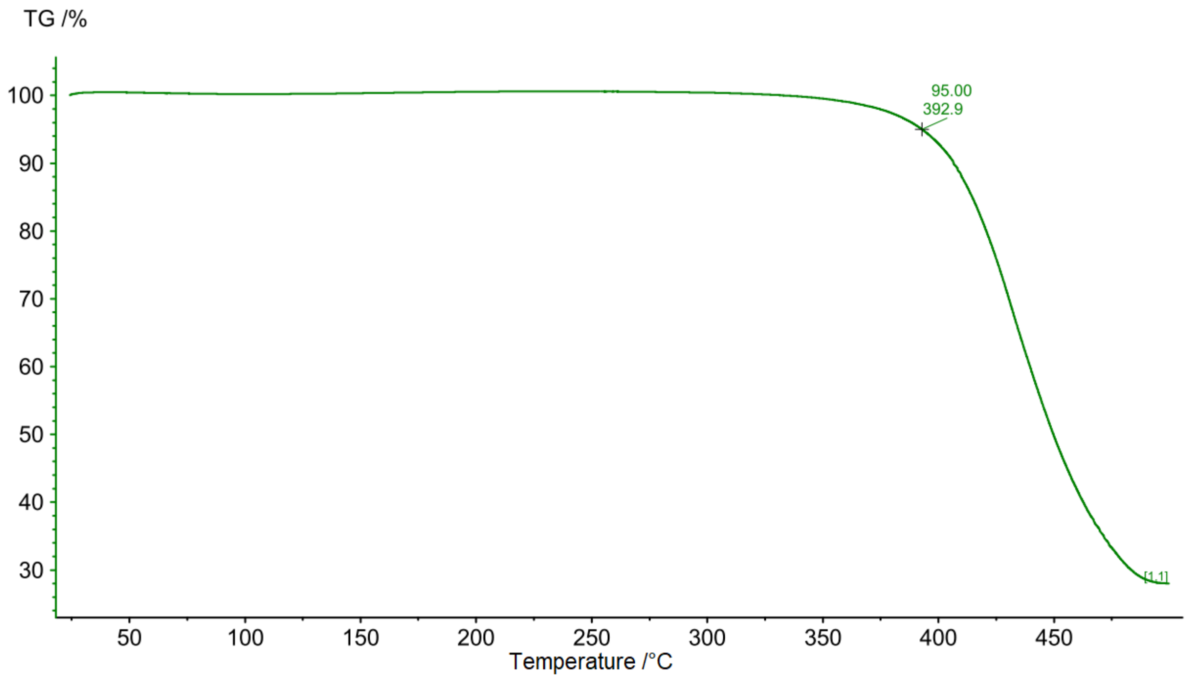

Figure S30. Thermogravimetric analysis of compound **49**.

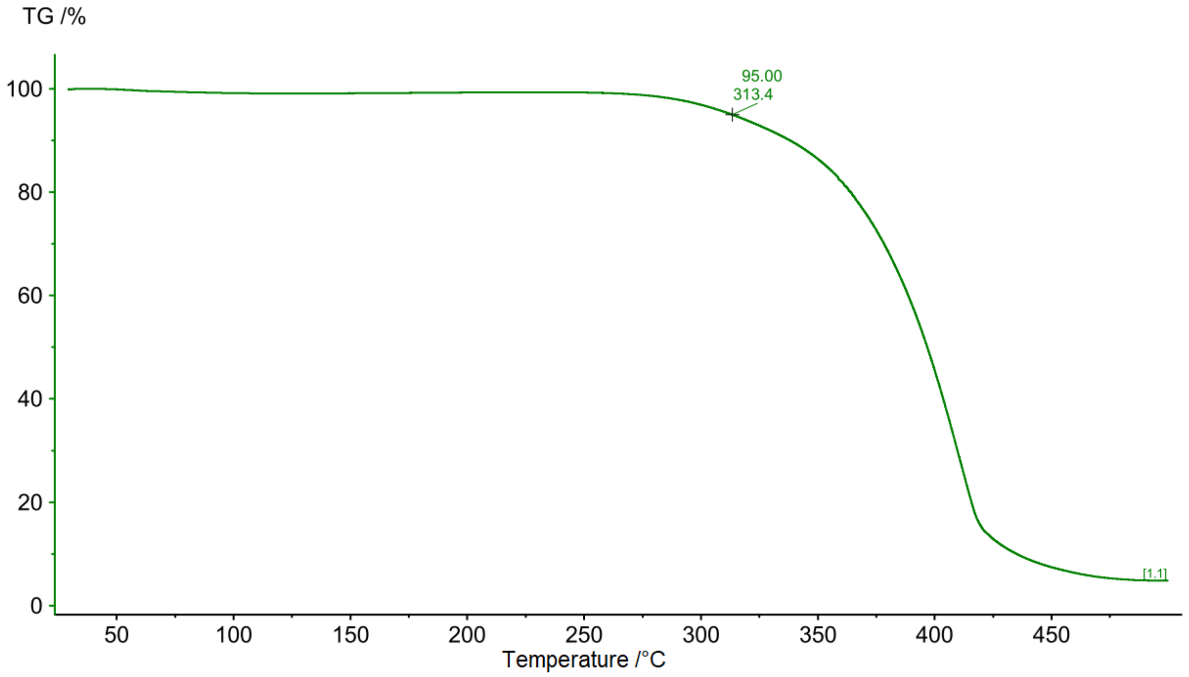

Figure S31. Thermogravimetric analysis of compound **50**.

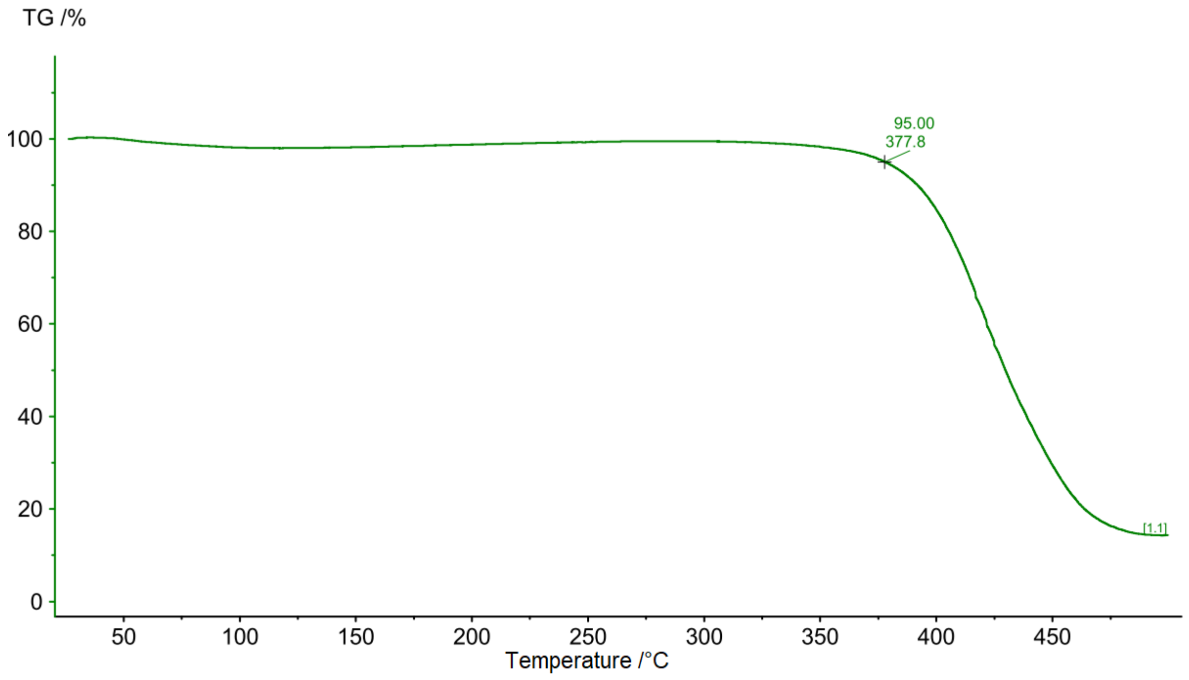

Figure S32. Thermogravimetric analysis of compound **51**.

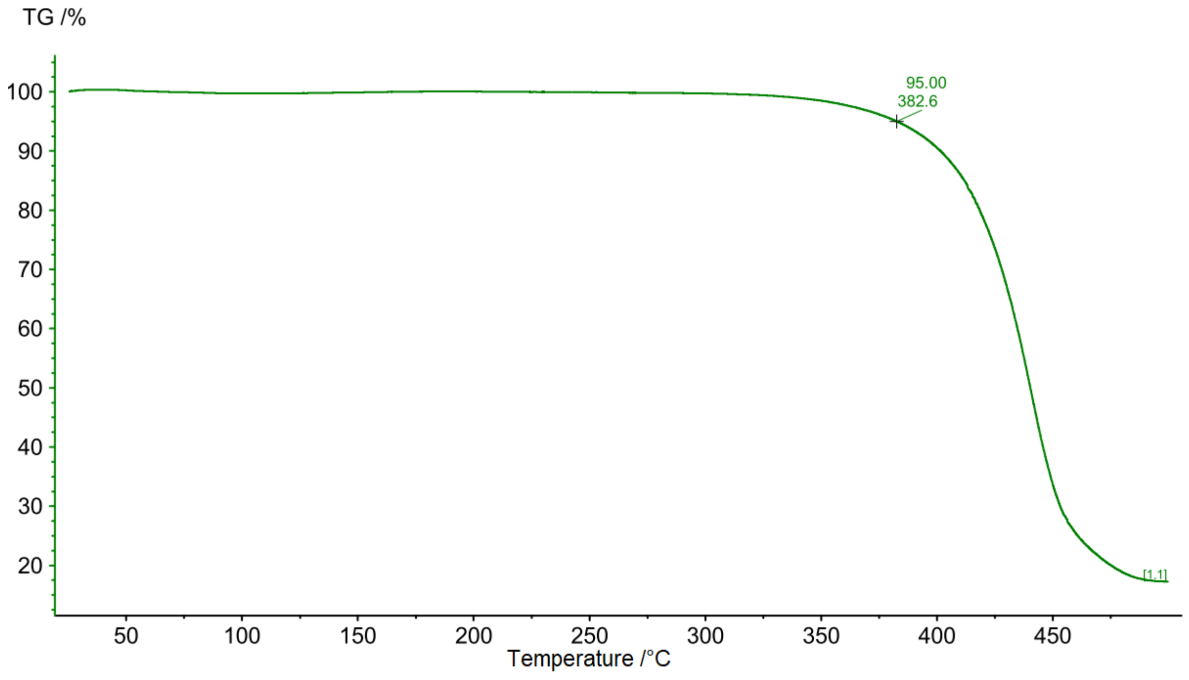

Figure S33. Thermogravimetric analysis of compound **52**.

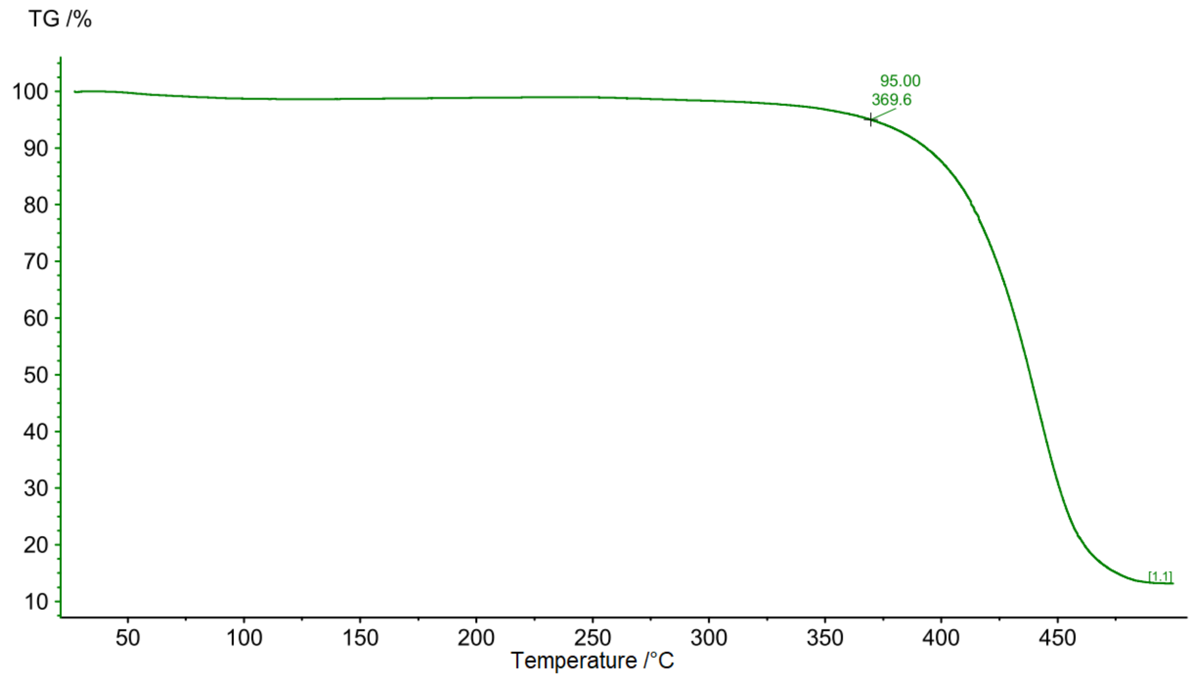

Figure S34. Thermogravimetric analysis of compound **53**.

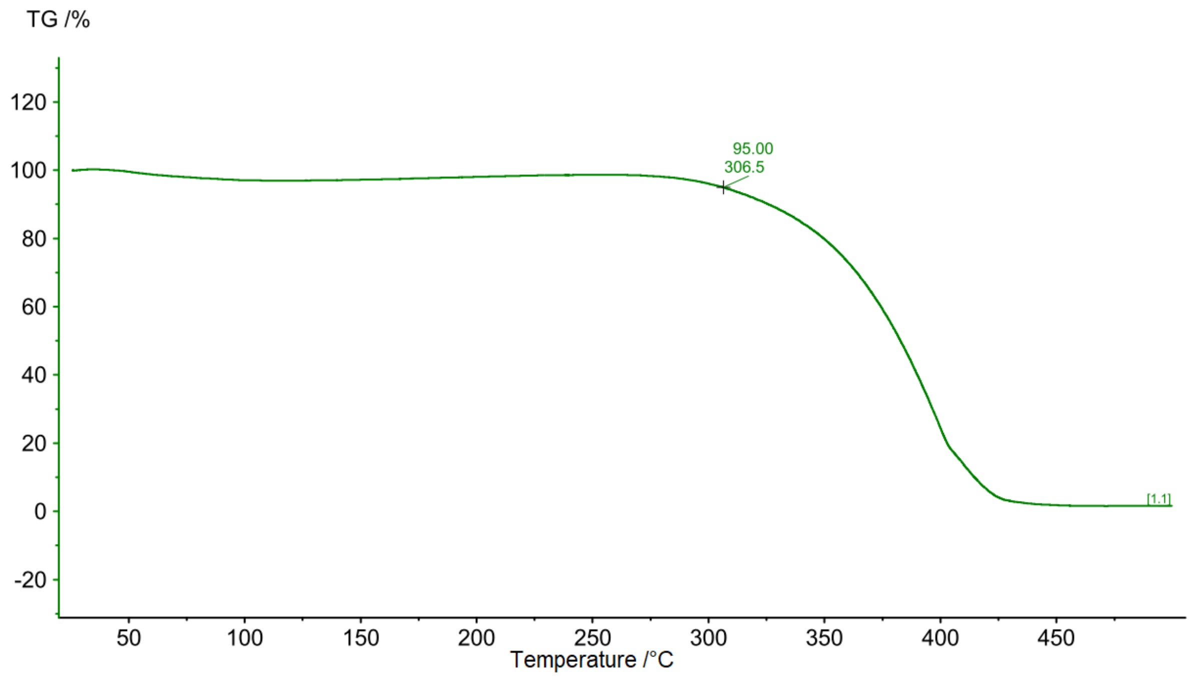

Figure S35. Thermogravimetric analysis of compound **54**.

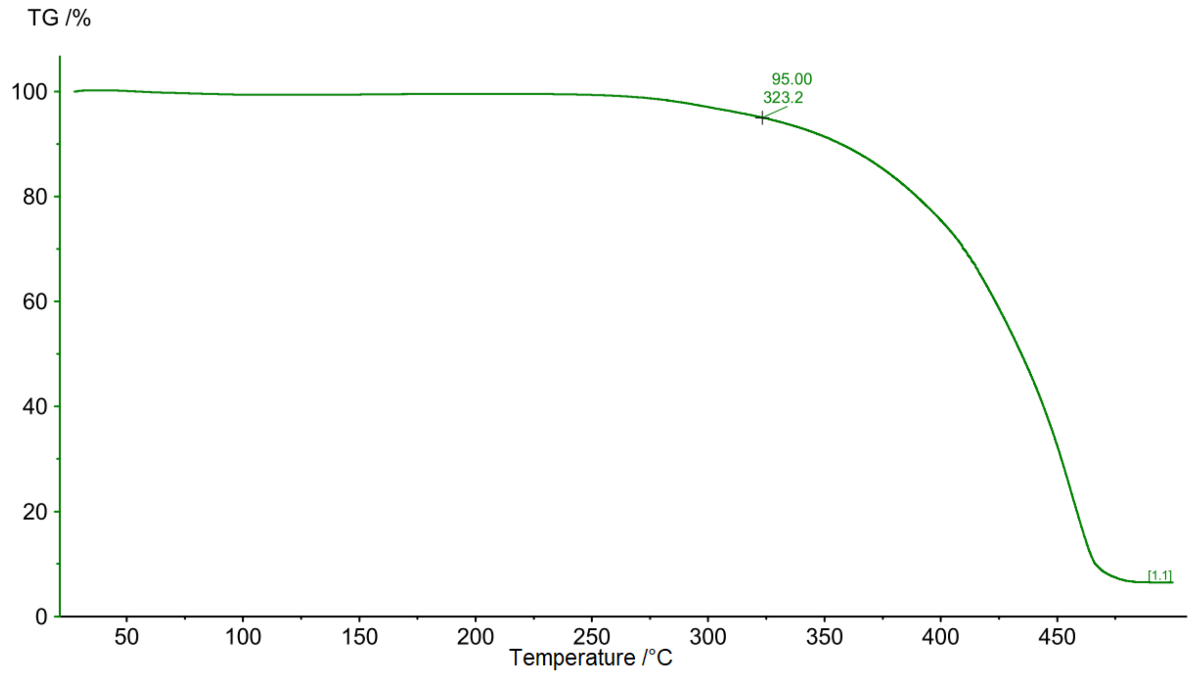

Figure S36. Thermogravimetric analysis of compound **55**.

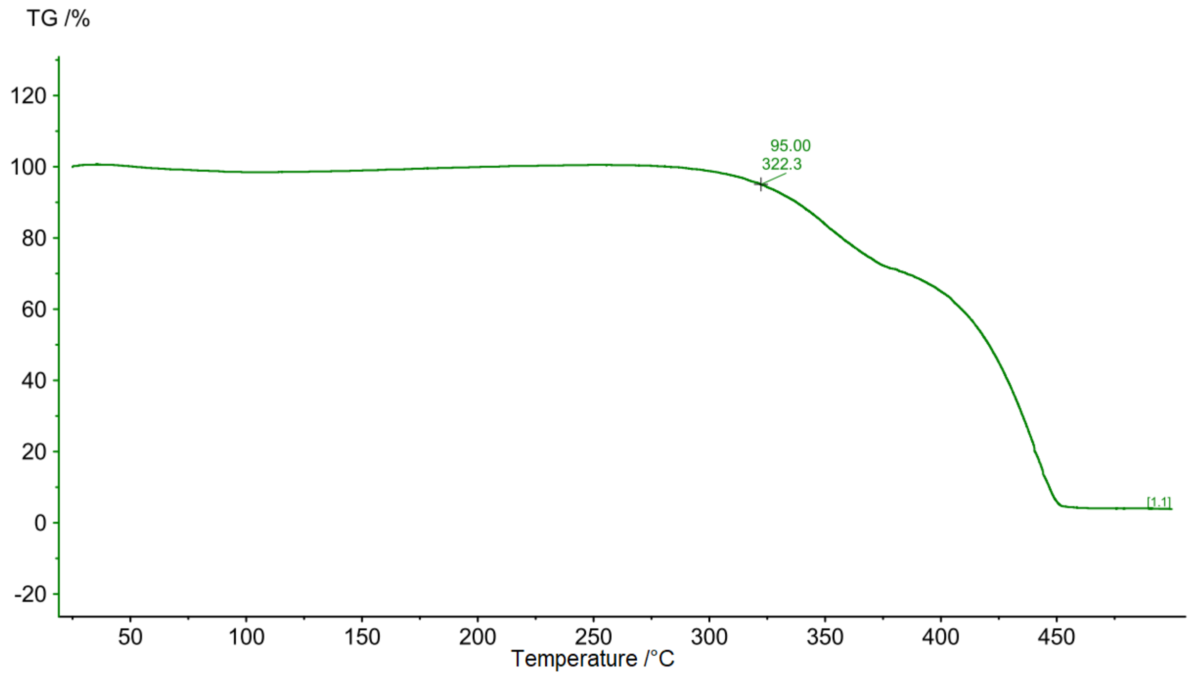

Figure S37. Thermogravimetric analysis of compound **56**.

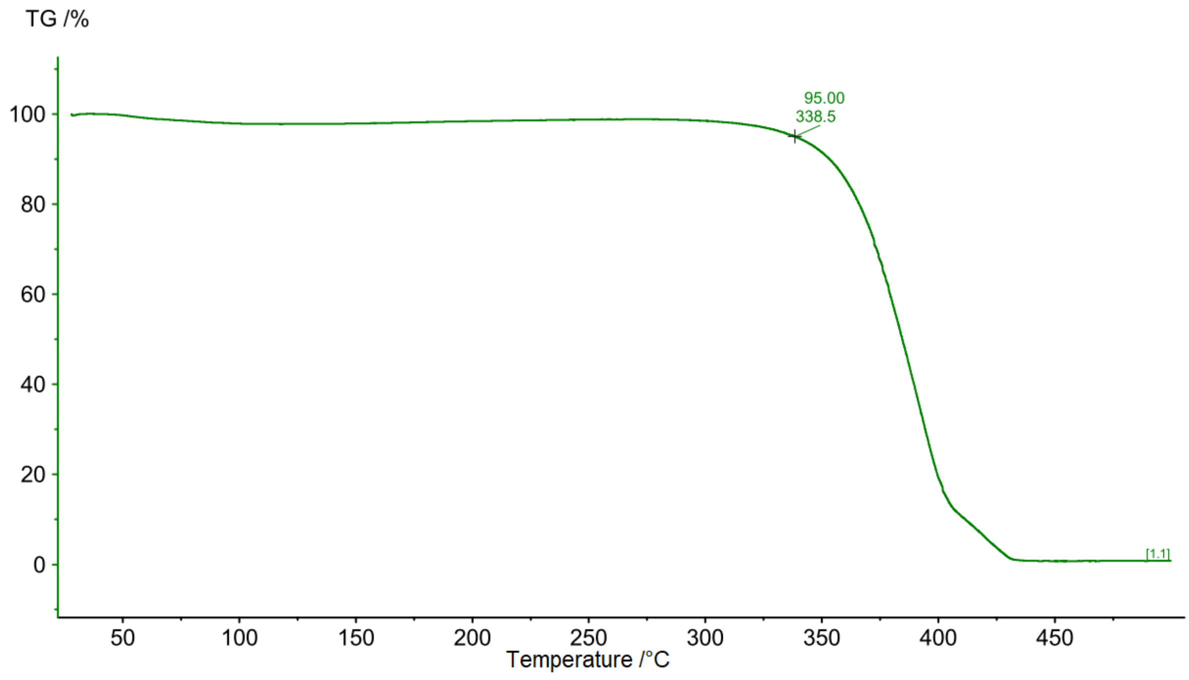

Figure S38. Thermogravimetric analysis of compound **57**.

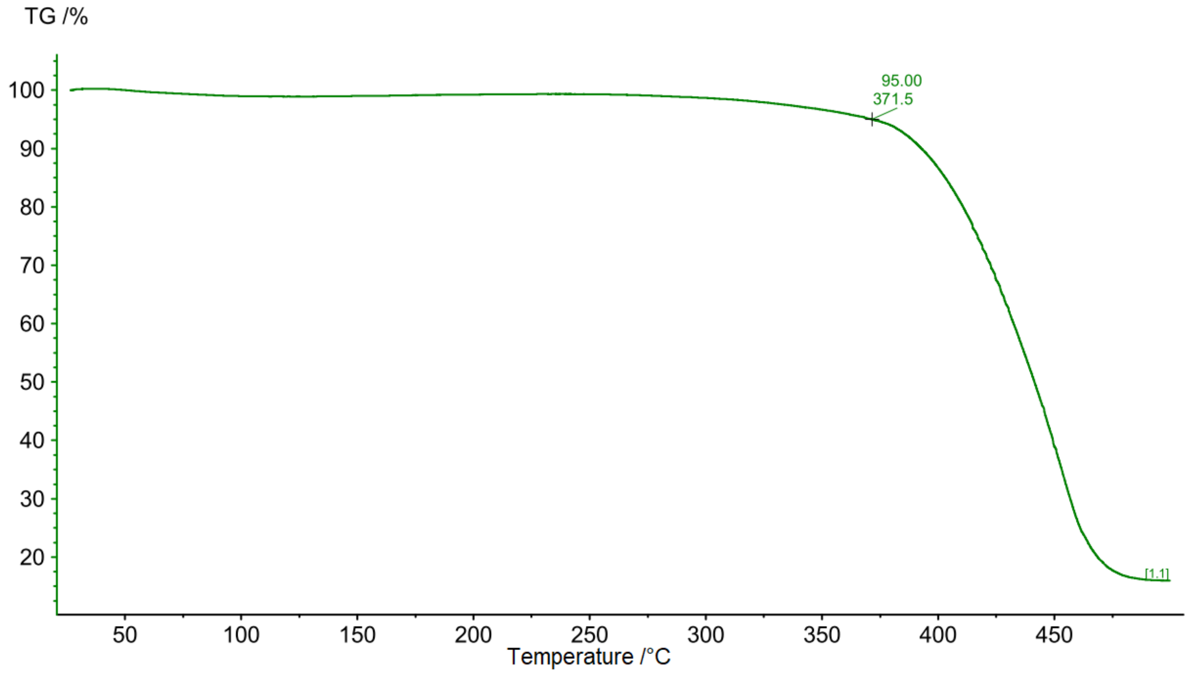

Figure S39. Thermogravimetric analysis of compound **58**.

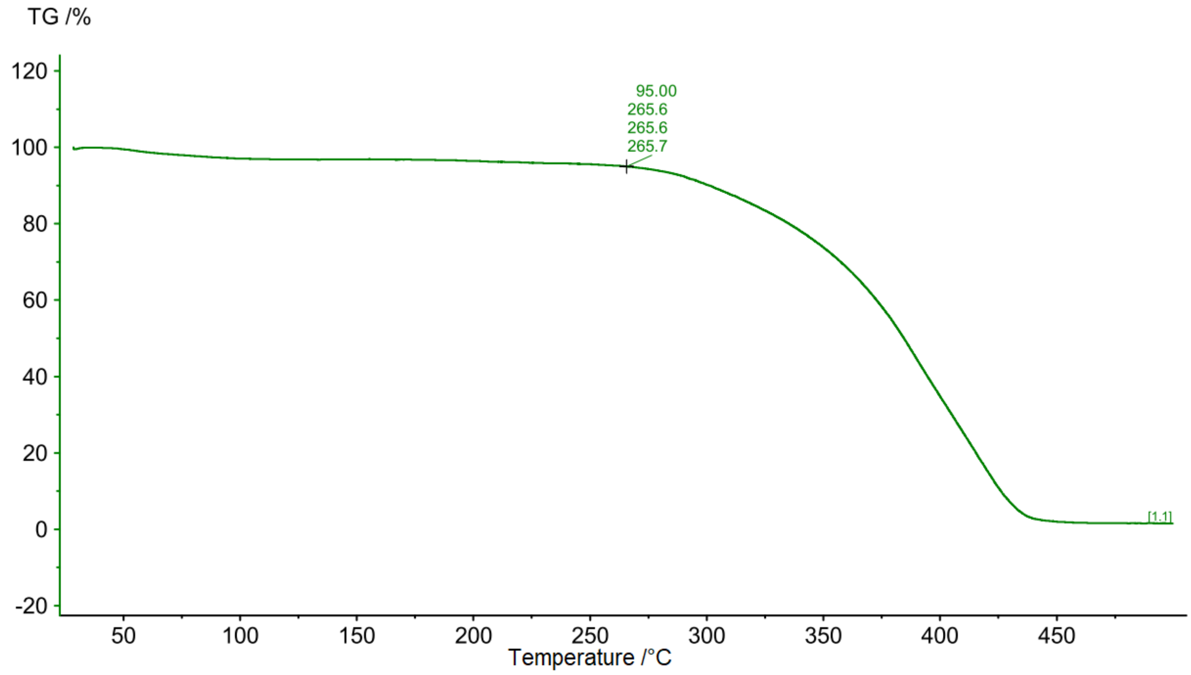

Figure S40. Thermogravimetric analysis of compound **59**.

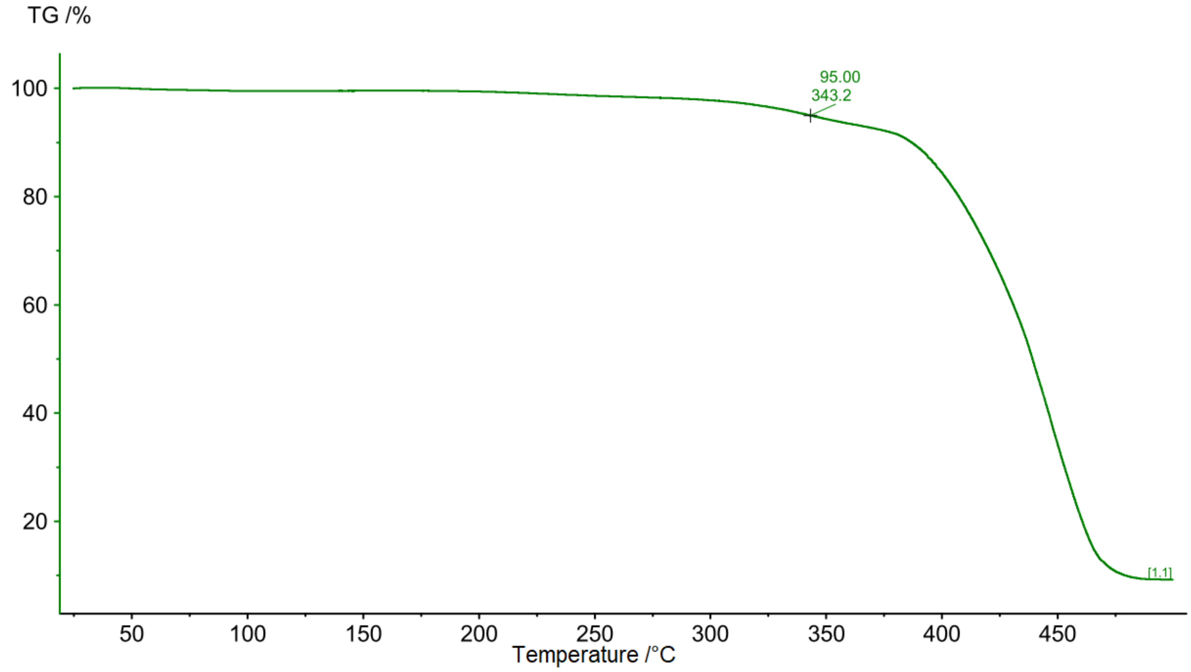

Figure S41. Thermogravimetric analysis of compound **60**.

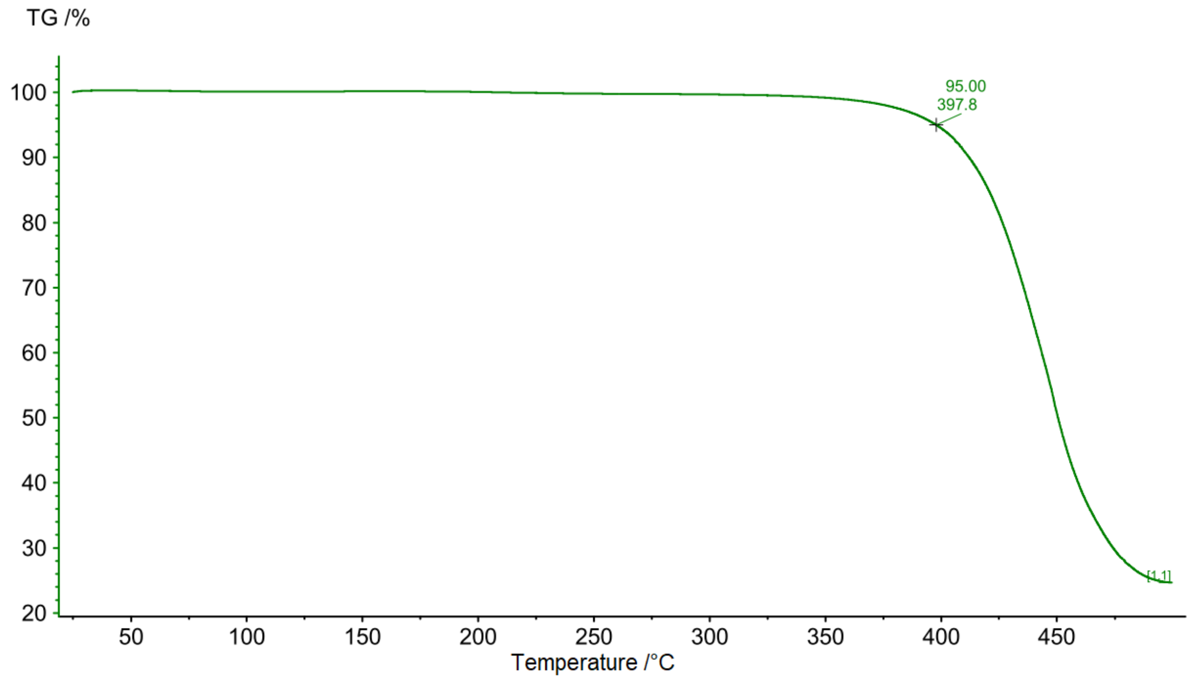

Figure S42. Thermogravimetric analysis of compound **61**.

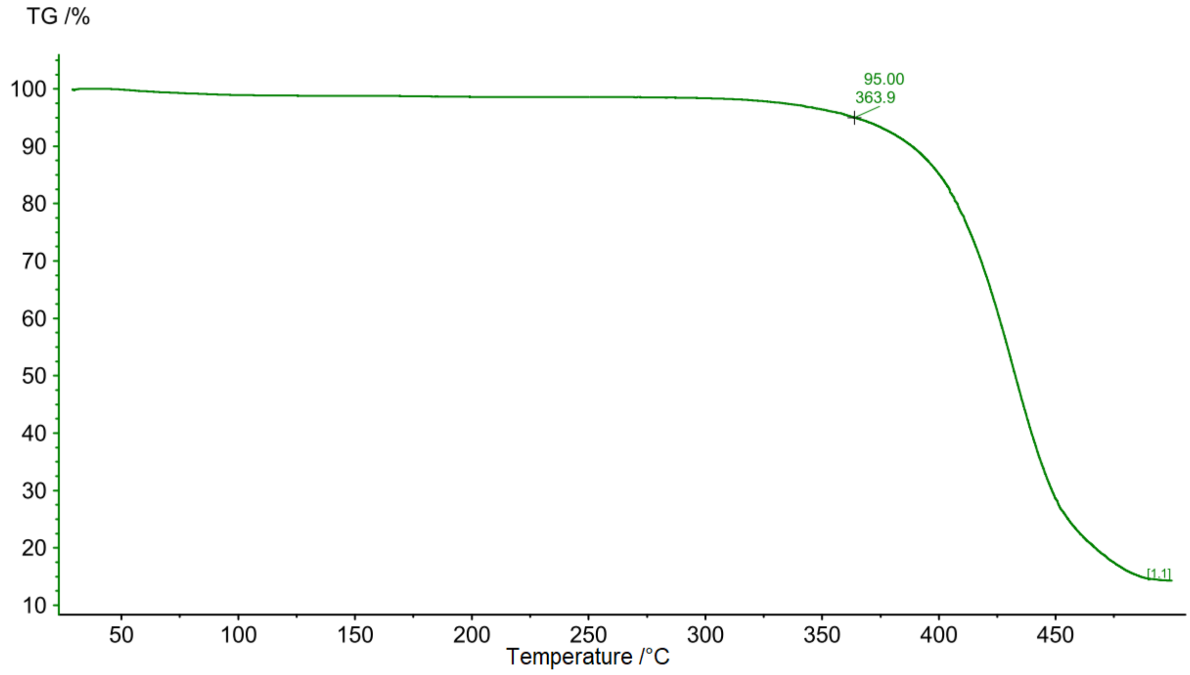

Figure S43. Thermogravimetric analysis of compound **62**.

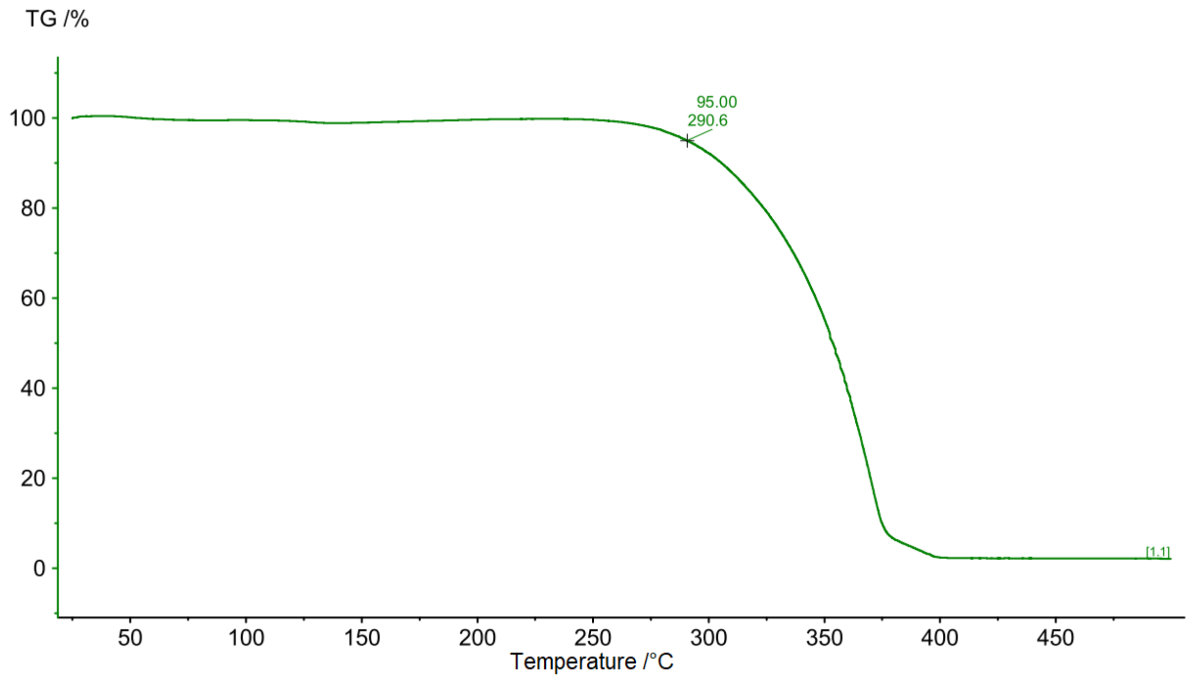

Figure S44. Thermogravimetric analysis of compound **63**.

S7 NMR-Spectra

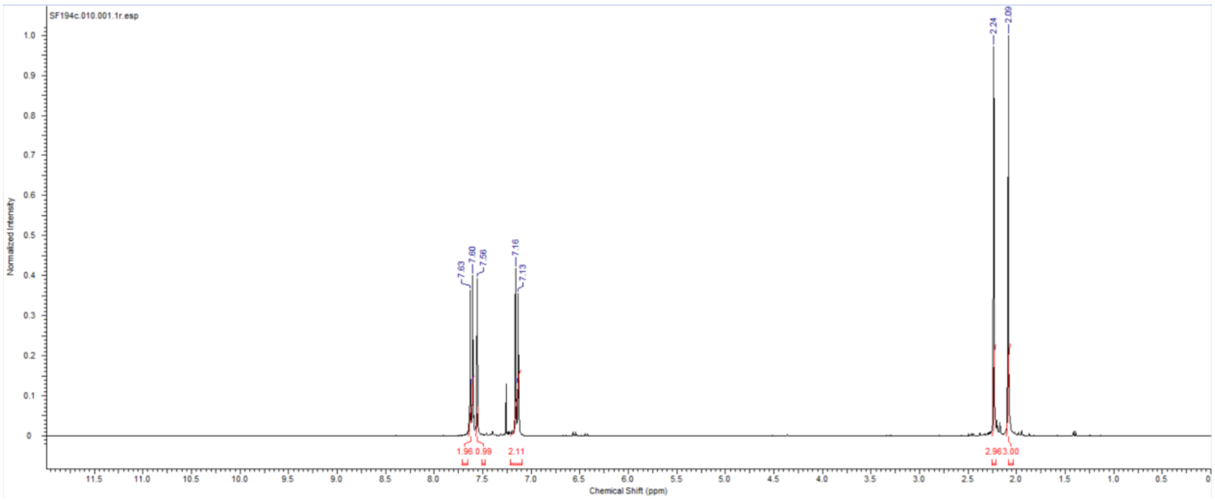

Figure S45. ^{1}H NMR of compound **4**.

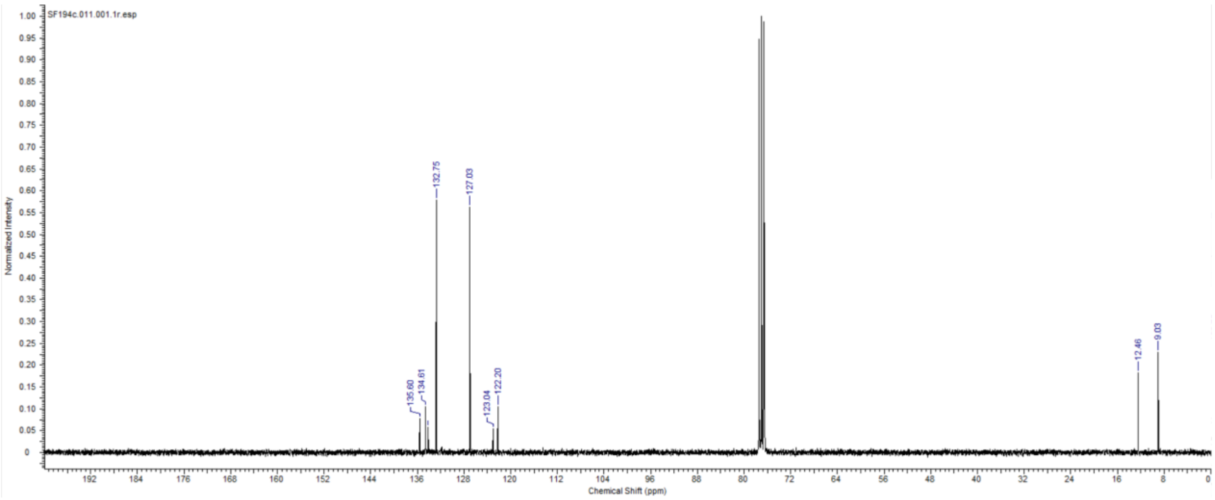

Figure S46. ^{13}C NMR of compound **4**.

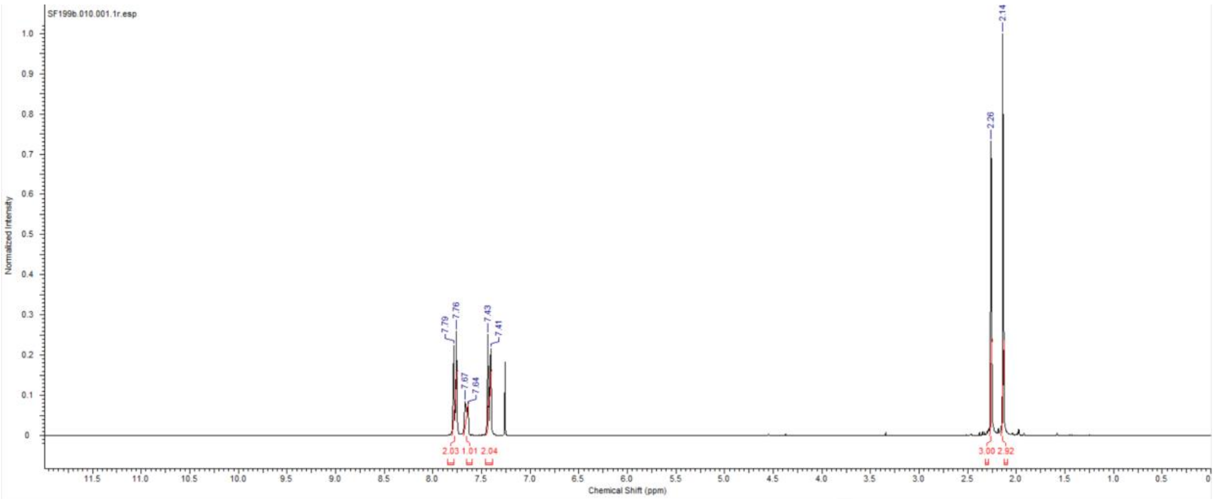

Figure S47. ^{1}H NMR of compound **5**.

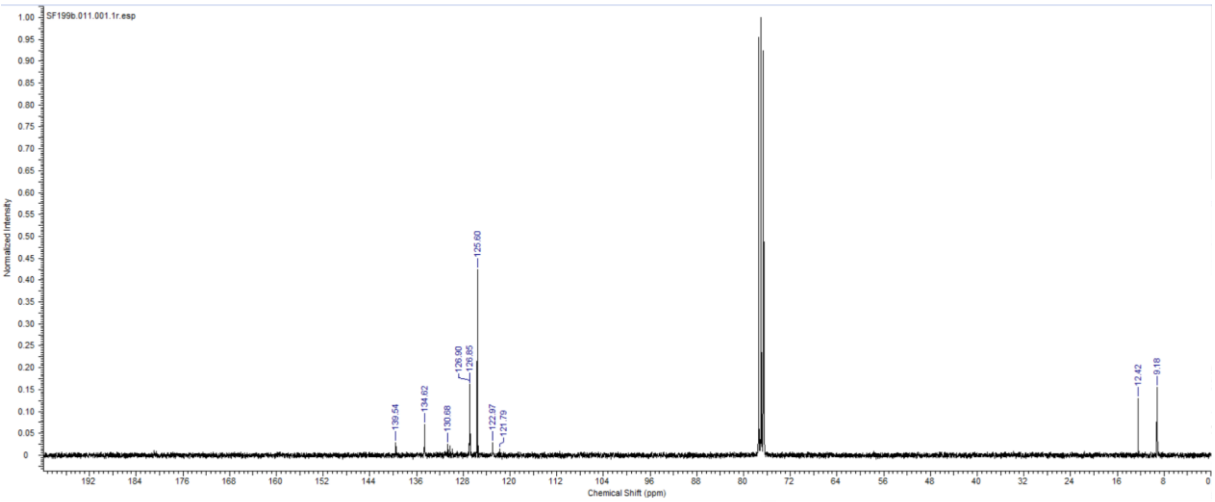

Figure S48. ^{13}C NMR of compound **5**.

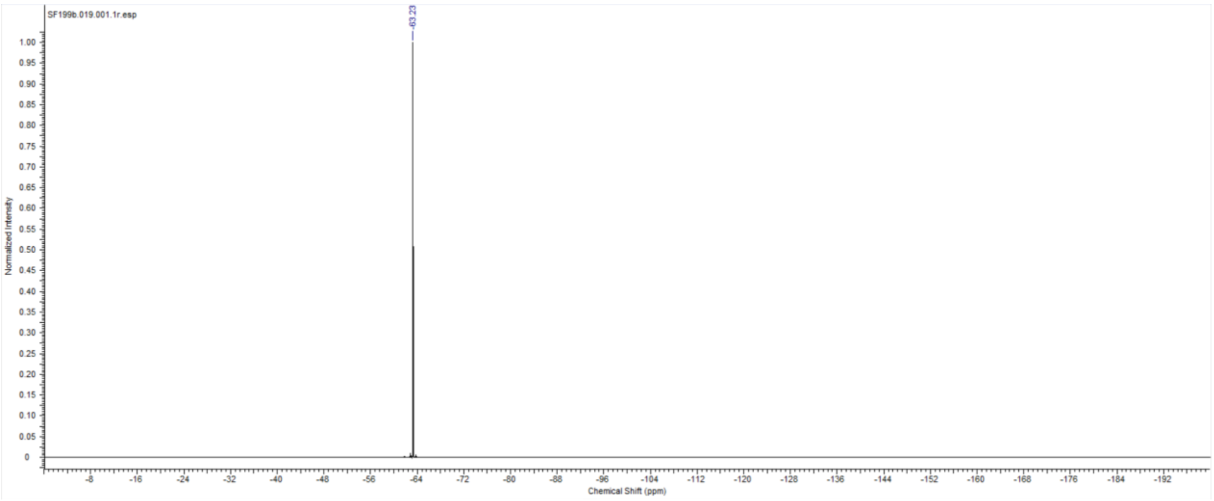

Figure S49. ^{19}F NMR of compound **5**.

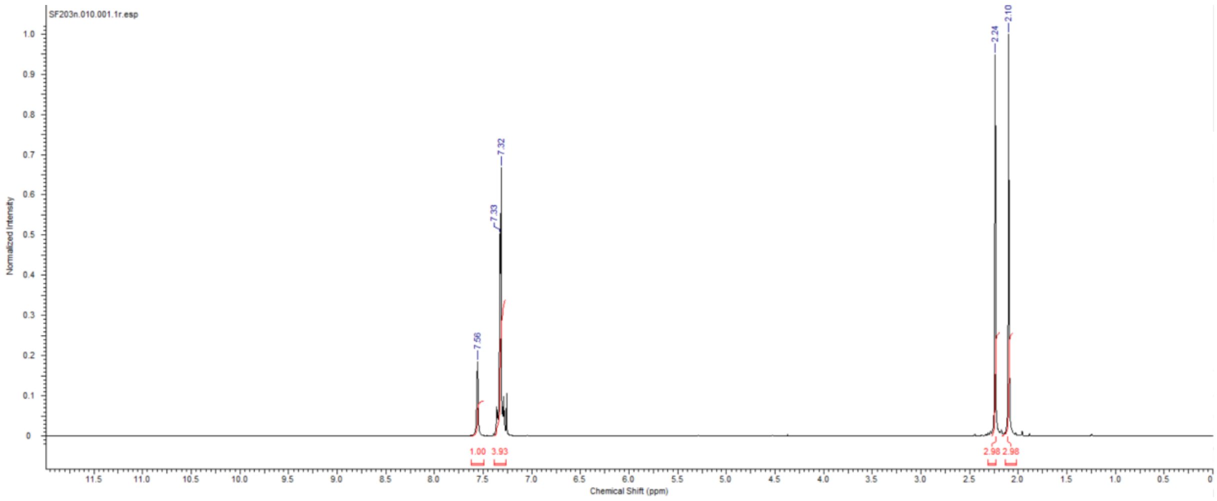

Figure S50. ^{1}H NMR of compound **6**.

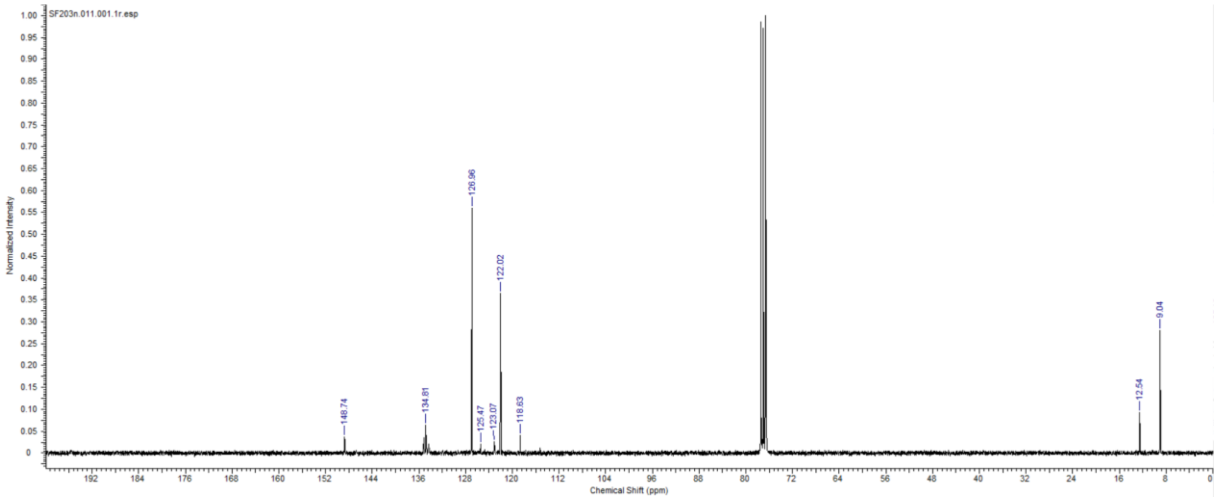

Figure S51. ^{13}C NMR of compound **6**.

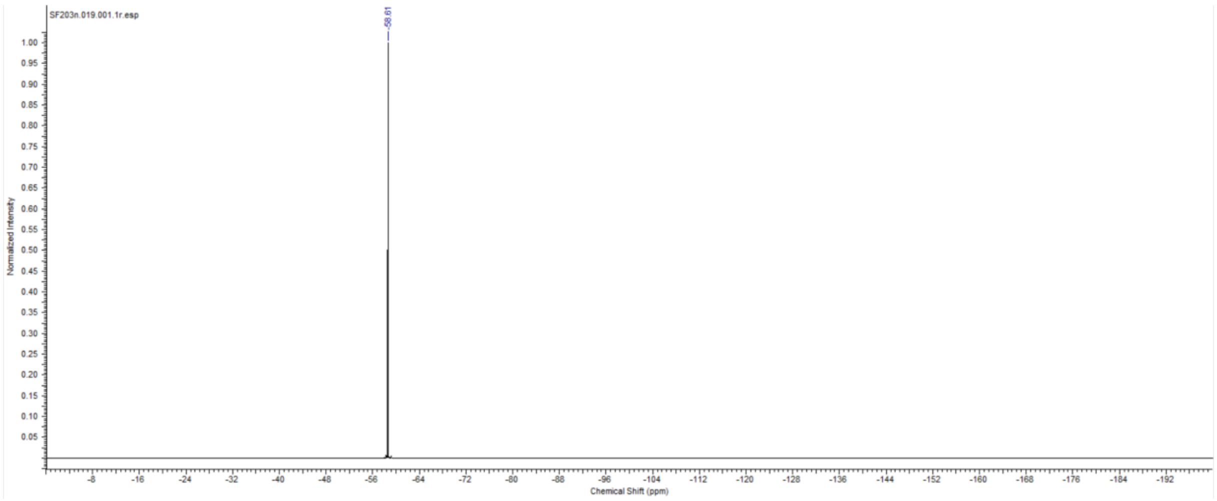

Figure S52. ^{19}F NMR of compound **6**.

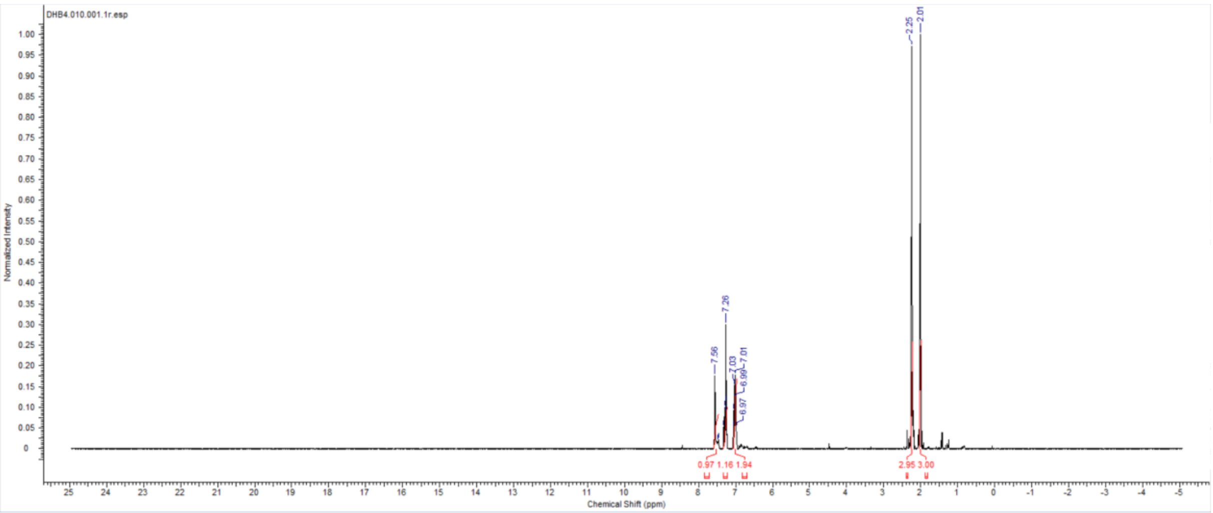

Figure S53. ^{1}H NMR of compound **9**.

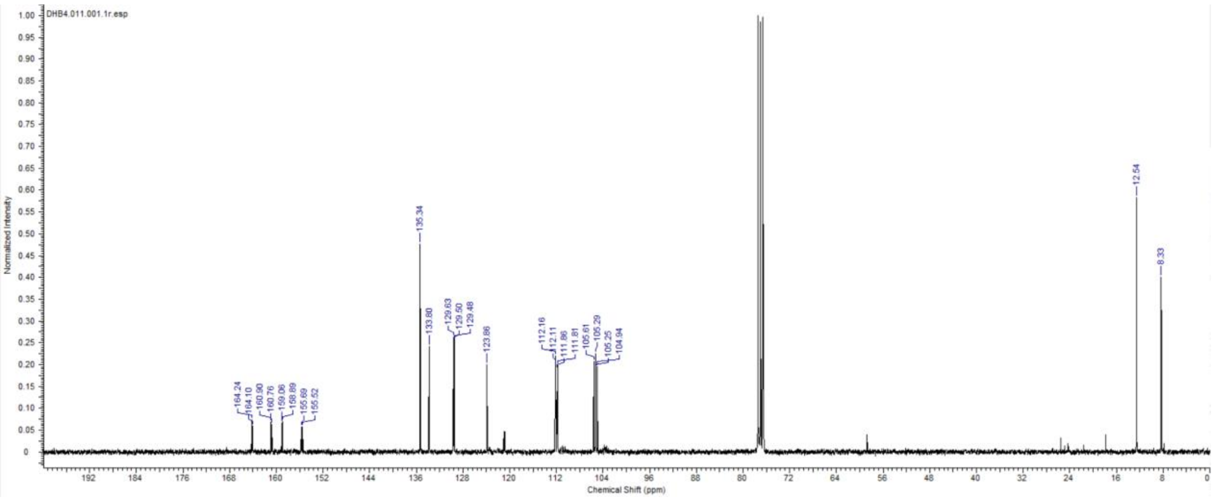

Figure S54. ^{13}C NMR of compound **9**.

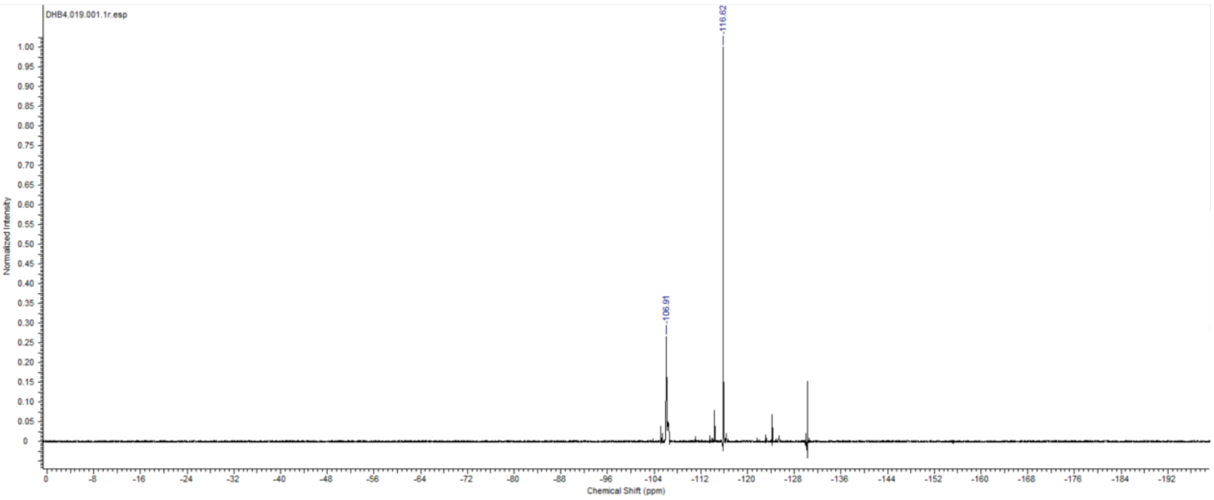

Figure S55. ^{19}F NMR of compound **9**.

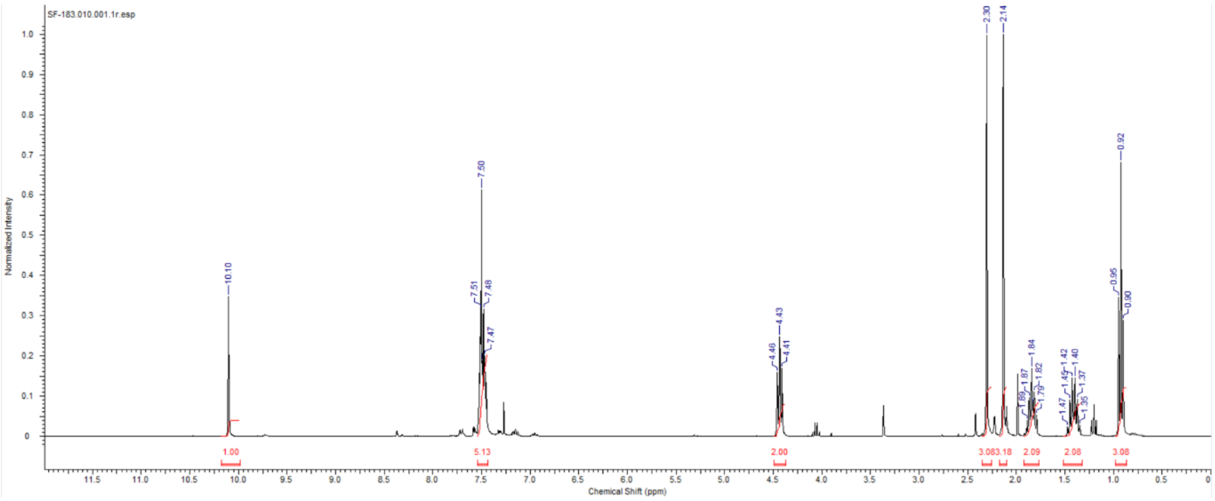

Figure S56. ^{1}H NMR of compound **10**.

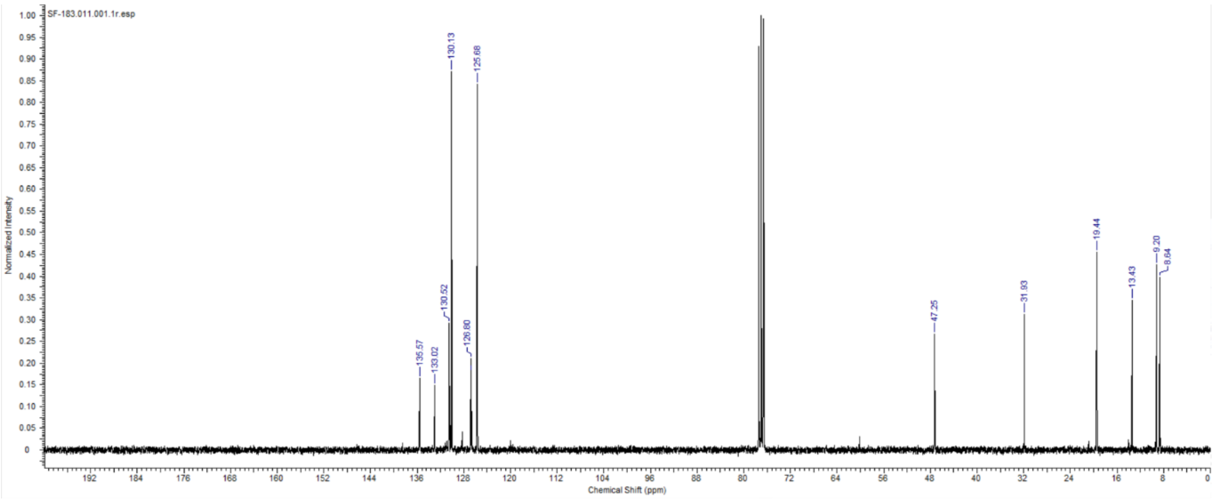

Figure S57. ^{13}C NMR of compound **10**.

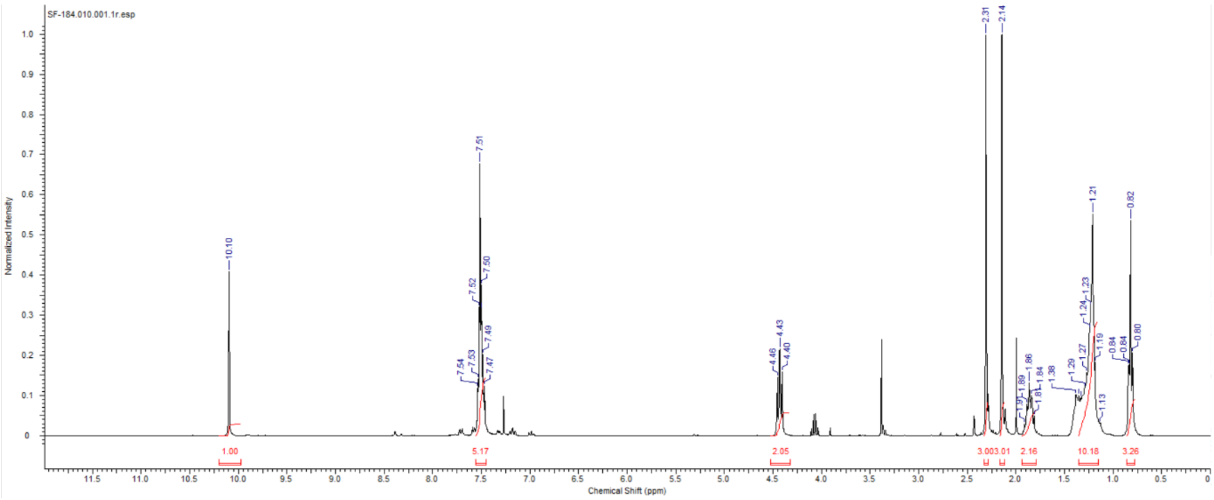

Figure S58. ^{1}H NMR of compound **11**.

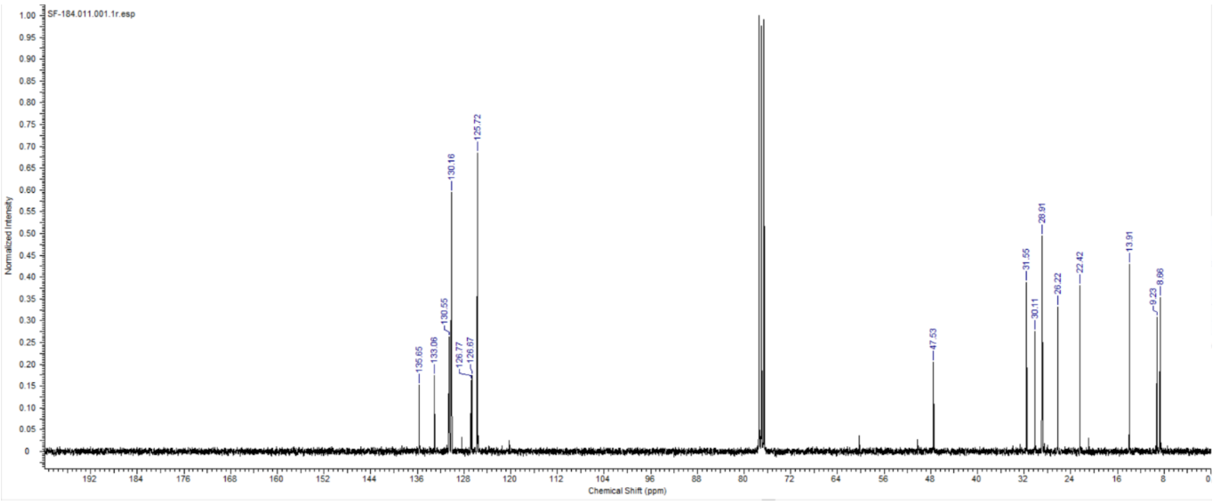

Figure S59. ^{13}C NMR of compound **11**.

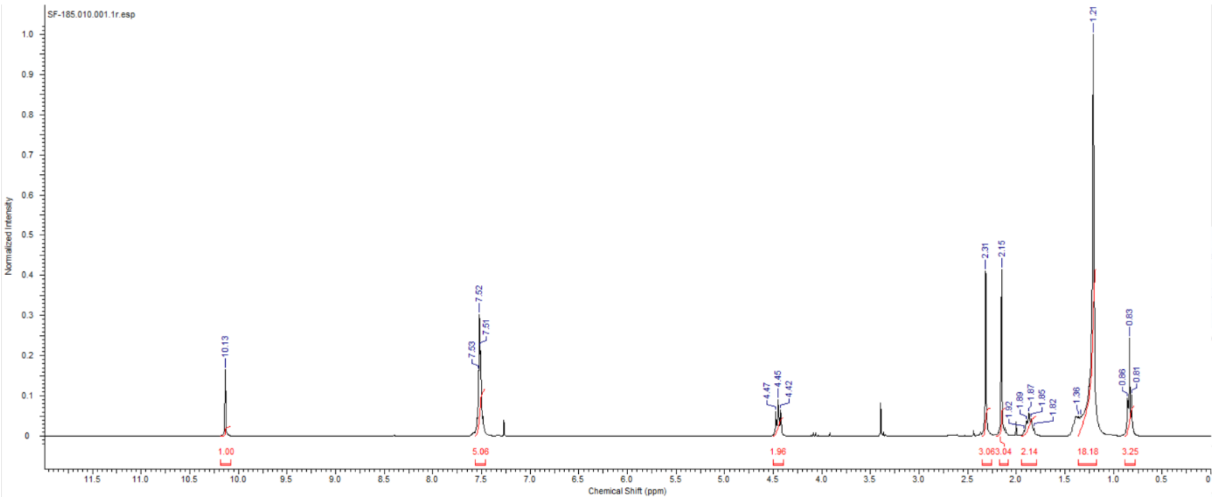

Figure S60. ^{1}H NMR of compound **12**.

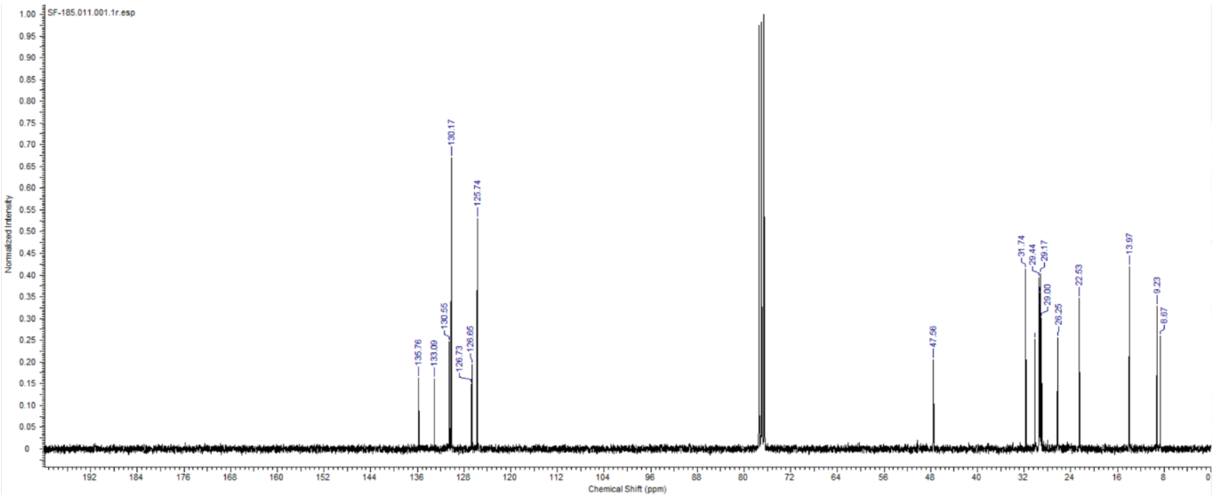

Figure S61. ^{13}C NMR of compound **12**.

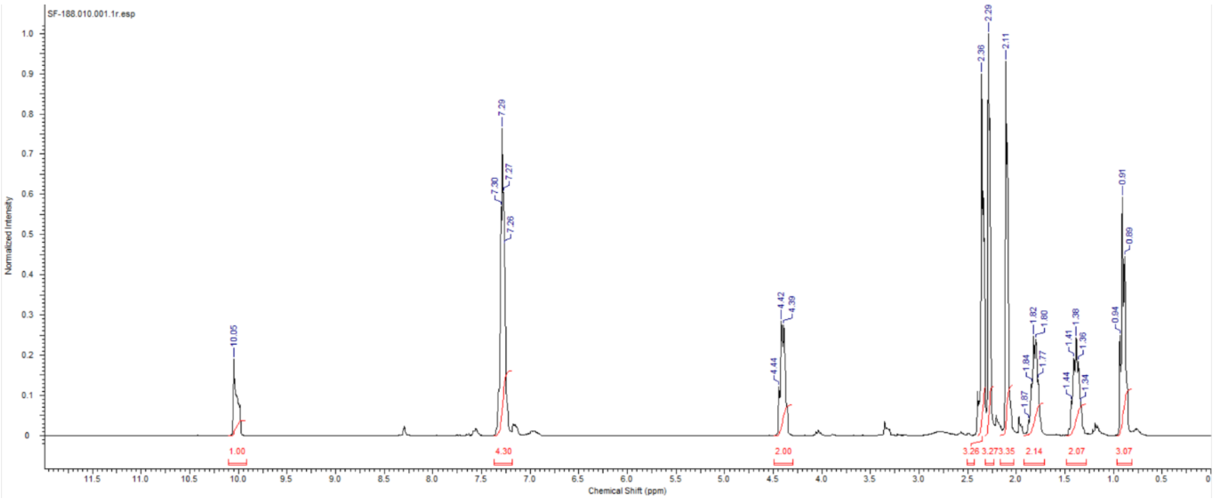

Figure S62. ^{1}H NMR of compound **13**.

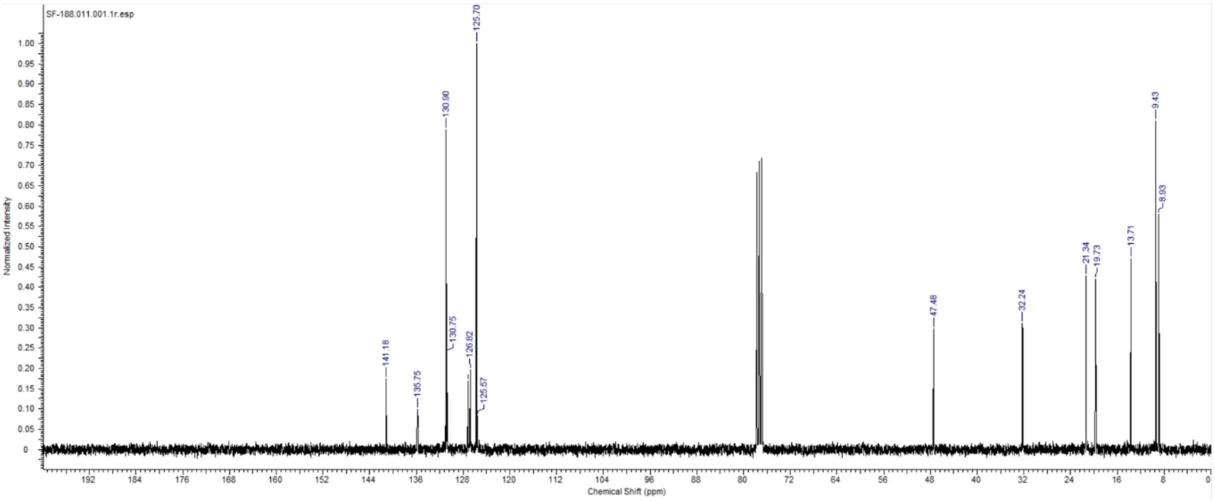

Figure S63. ^{13}C NMR of compound **13**.

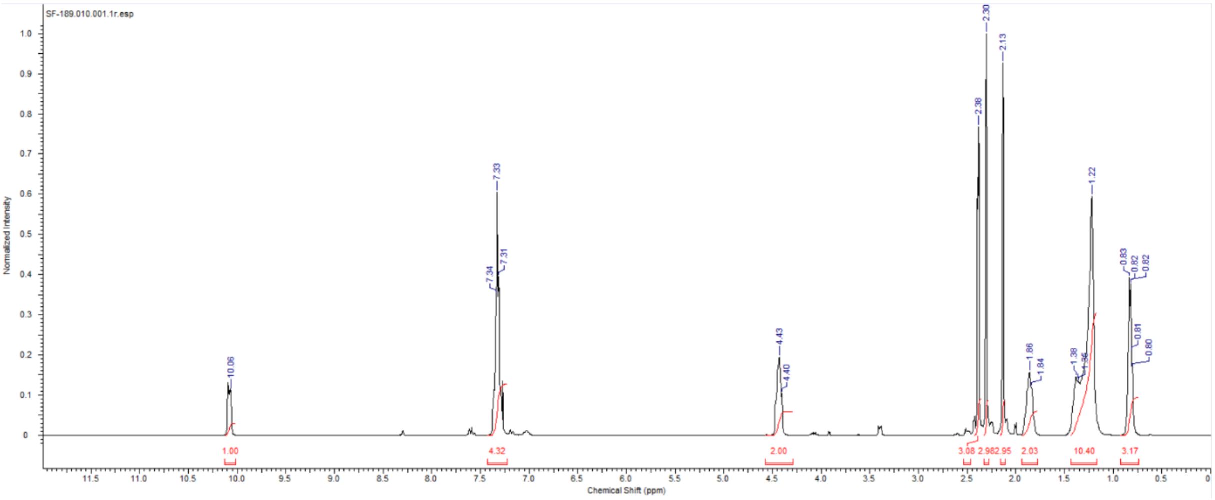

Figure S64. ^{1}H NMR of compound **14**.

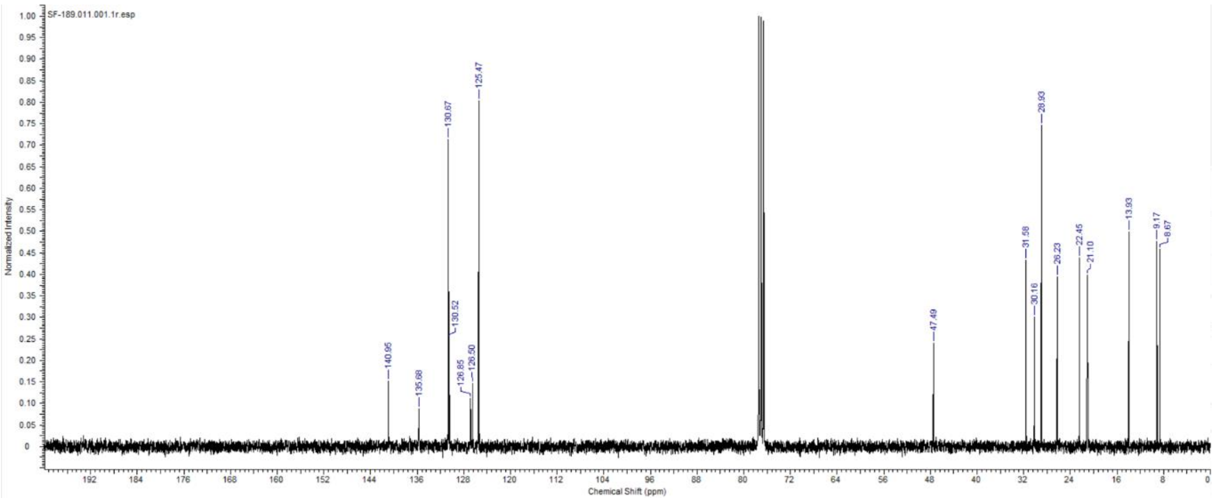

Figure S65. ^{13}C NMR of compound **14**.

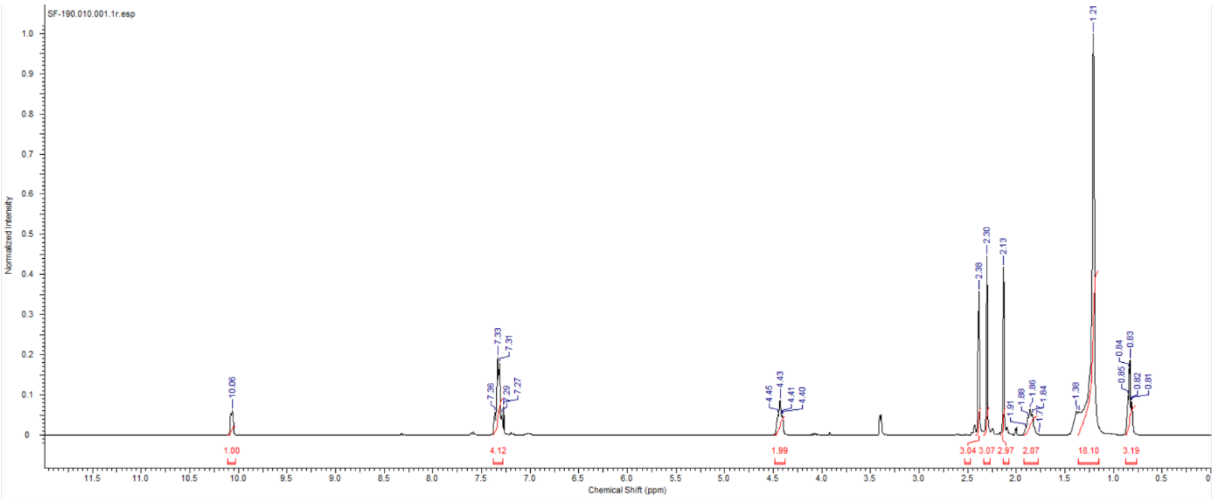

Figure S66. ^{1}H NMR of compound **15**.

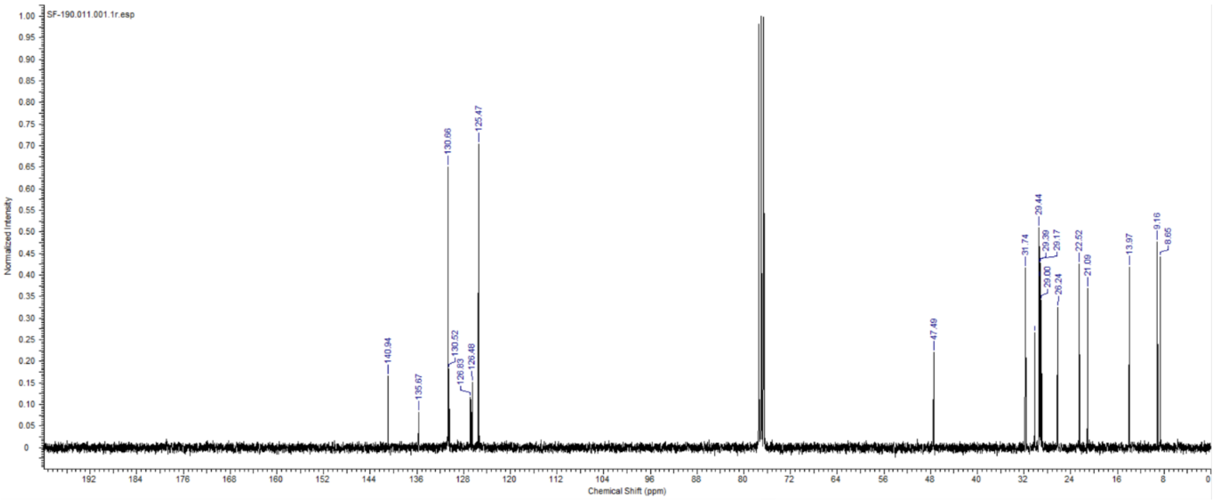

Figure S67. ^{13}C NMR of compound **15**.

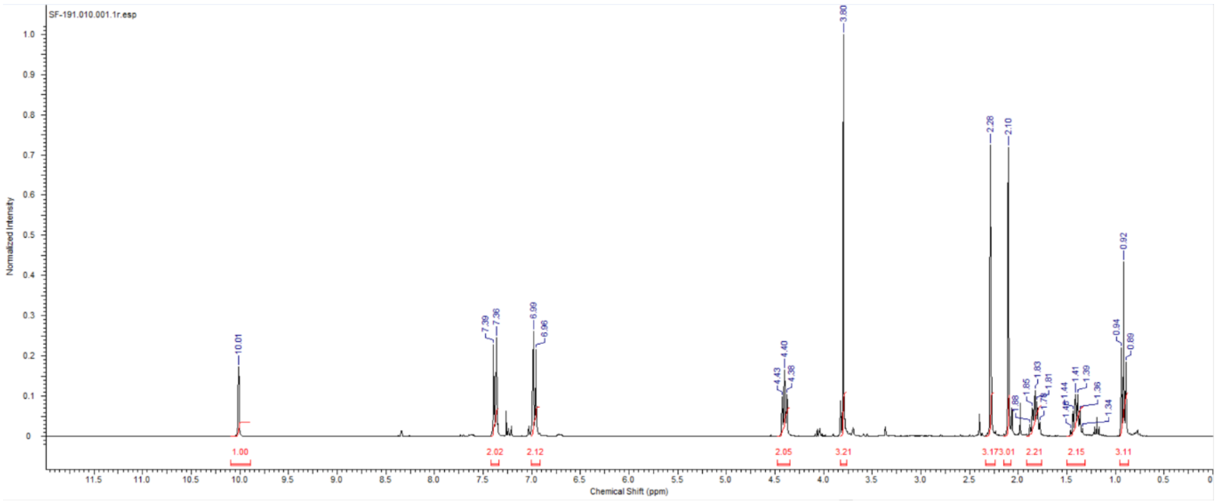

Figure S68. ^{1}H NMR of compound **16**.

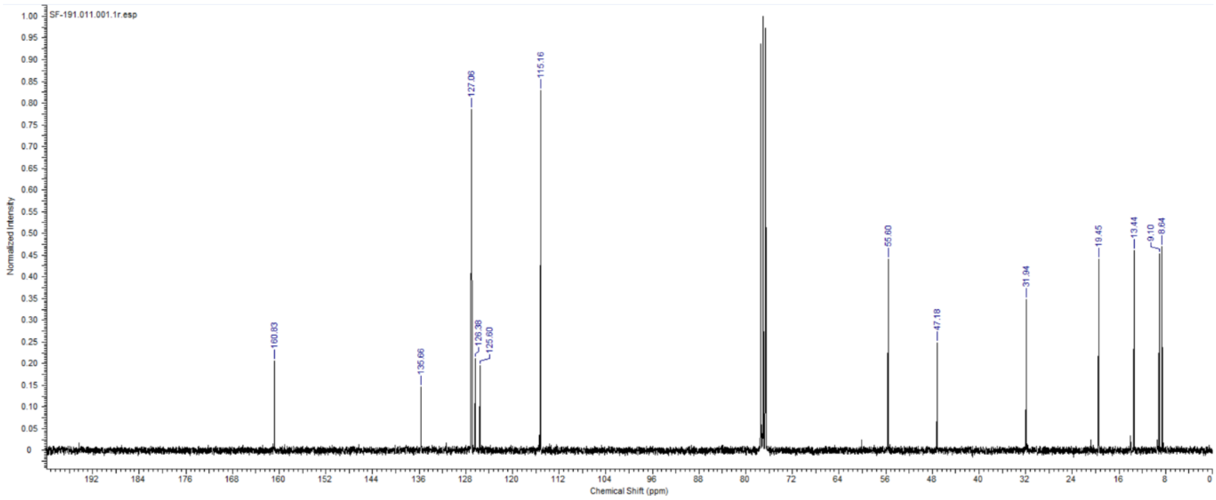

Figure S69. ^{13}C NMR of compound **16**.

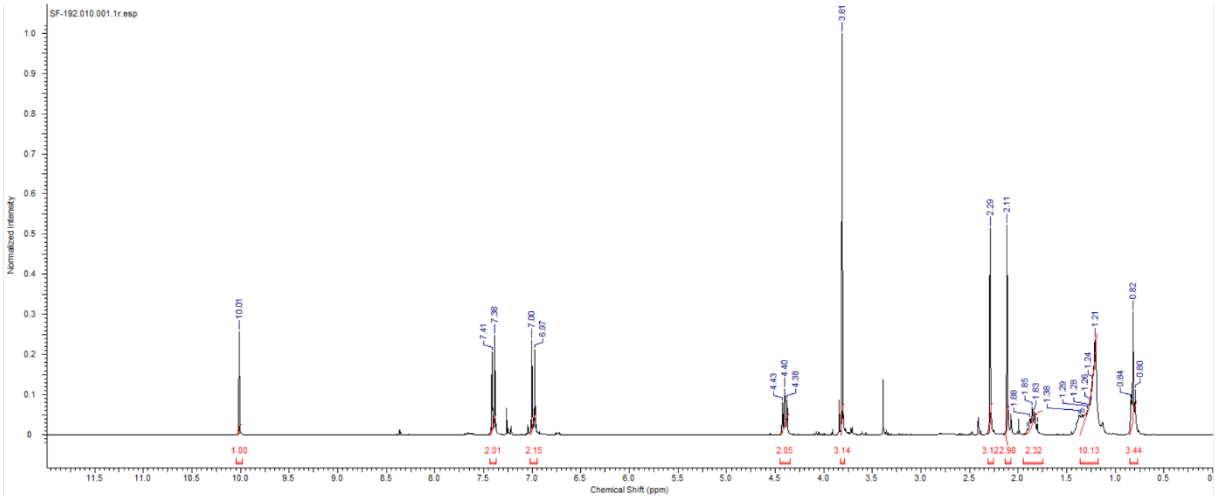

Figure S70. ^{1}H NMR of compound **17**.

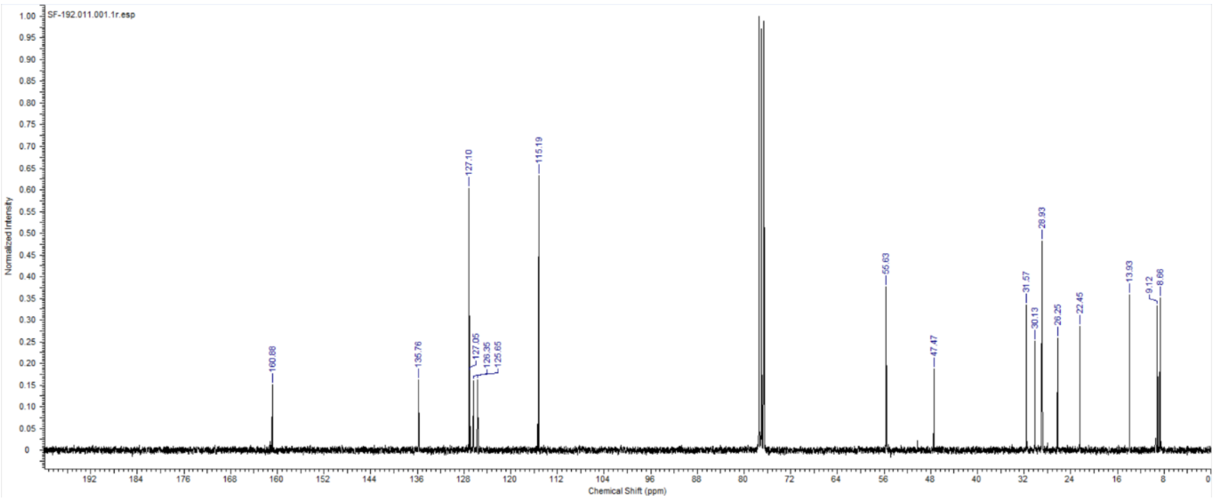

Figure S71. ^{13}C NMR of compound **17**.

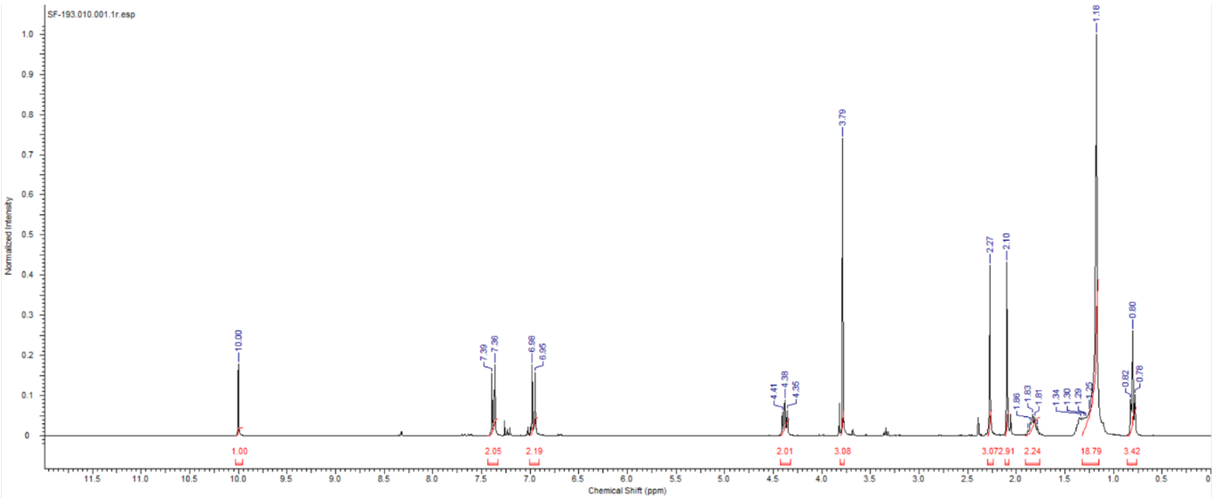

Figure S72. ^{1}H NMR of compound **18**.

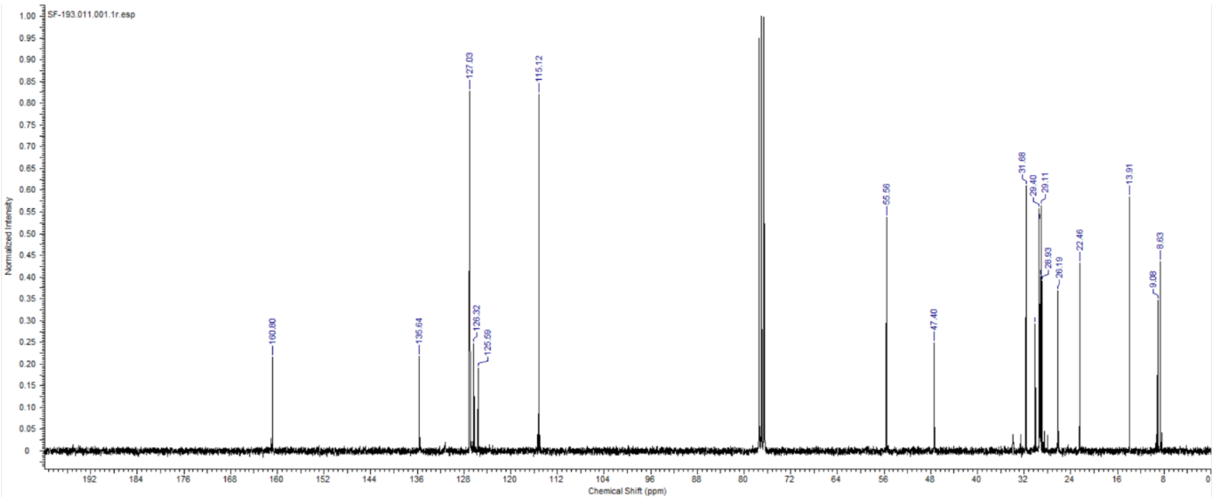

Figure S73. ^{13}C NMR of compound **18**.

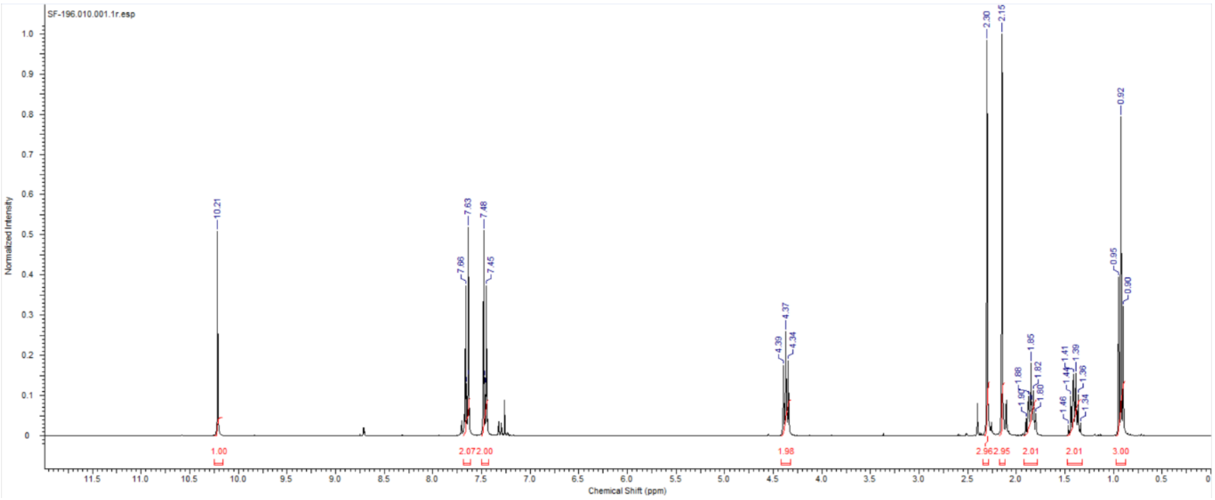

Figure S74. ^{1}H NMR of compound **19**.

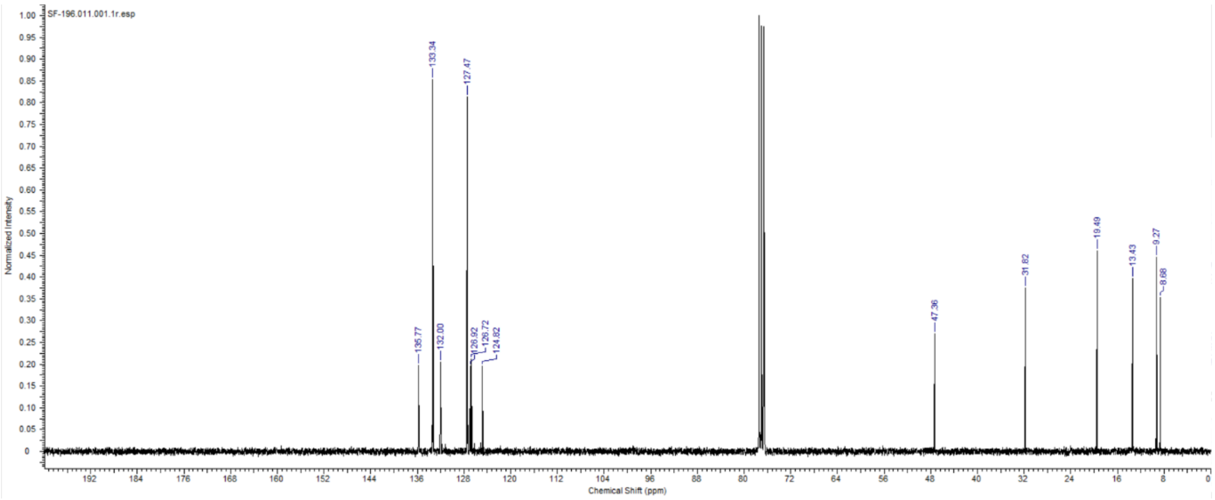

Figure S75. ^{13}C NMR of compound **19**.

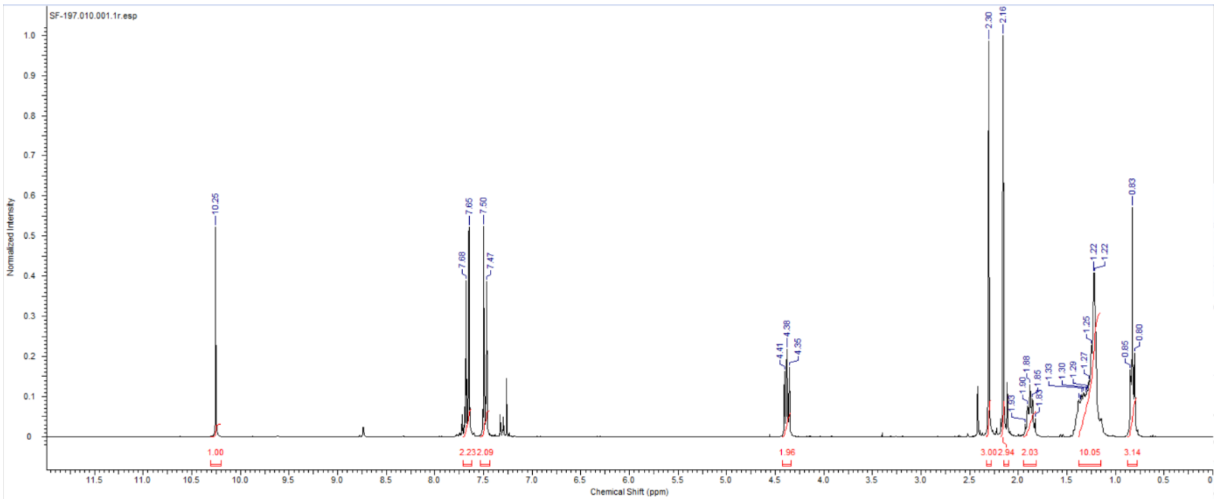

Figure S76. ^{1}H NMR of compound **20**.

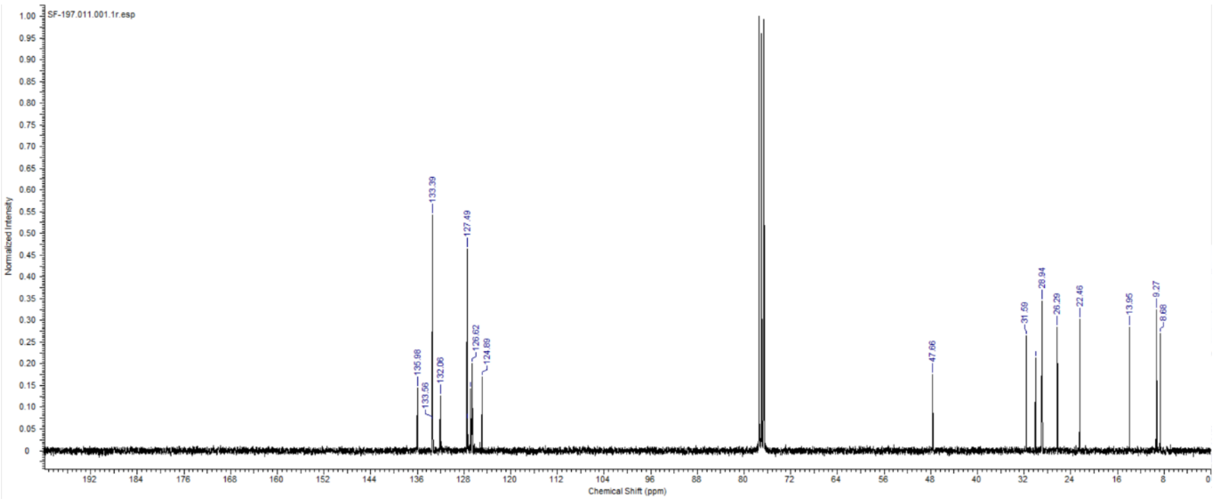

Figure S77. ^{13}C NMR of compound **20**.

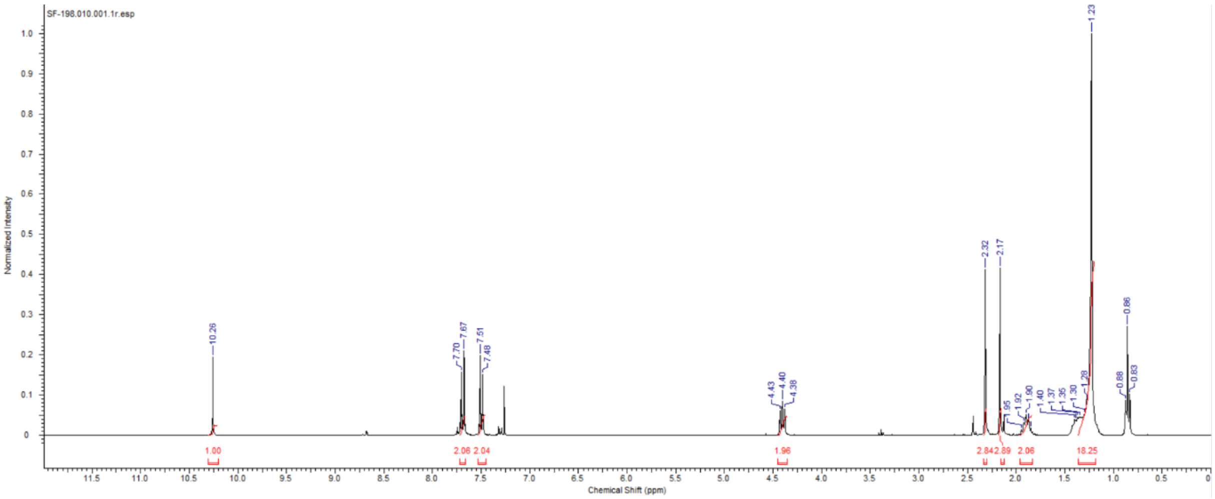

Figure S78. ^{1}H NMR of compound **21**.

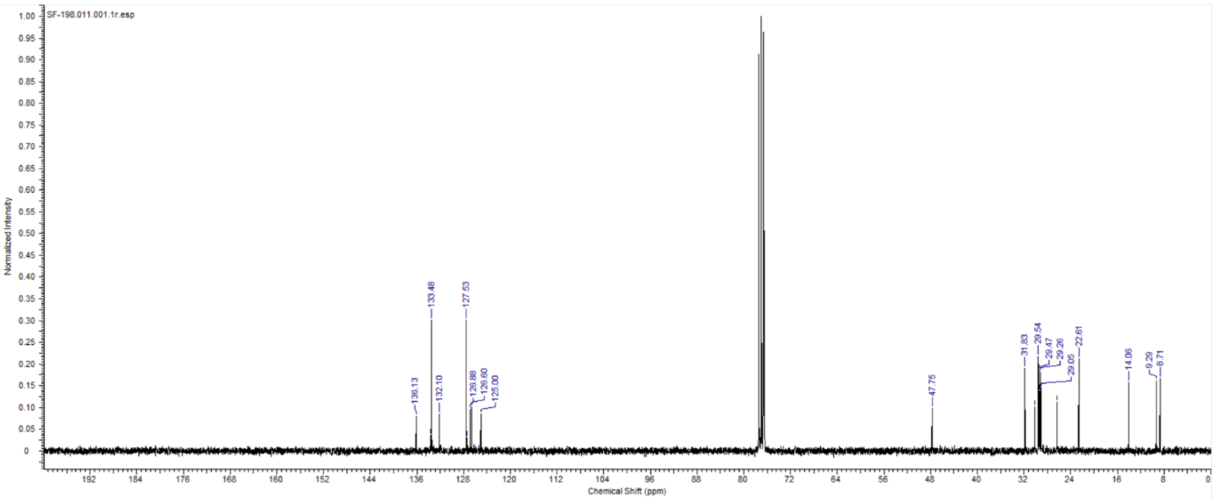

Figure S79. ^{13}C NMR of compound **21**.

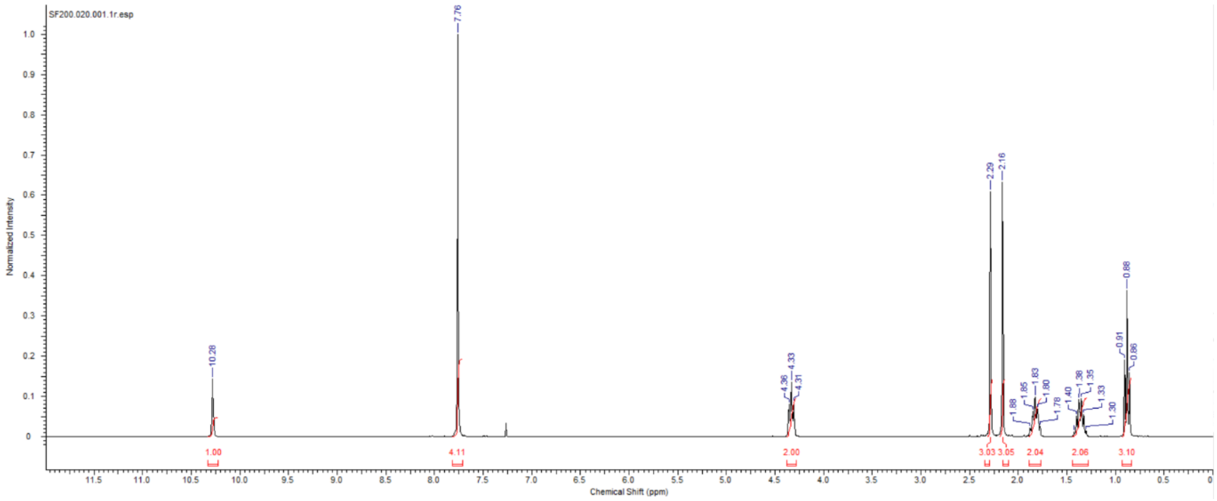

Figure S80. ^{1}H NMR of compound **22**.

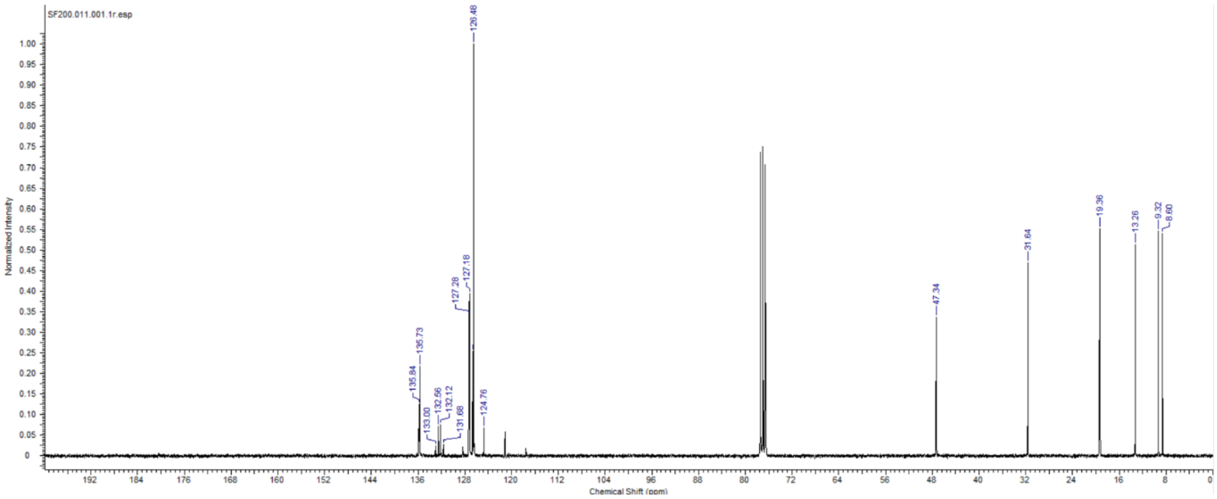

Figure S81. ^{13}C NMR of compound **22**.

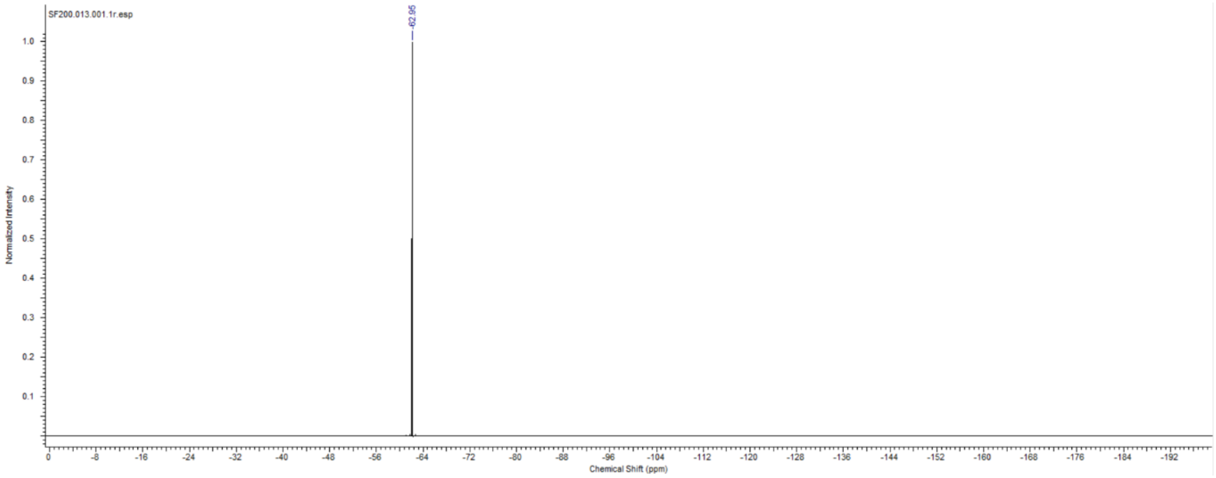

Figure S82. ^{19}F NMR of compound **22**.

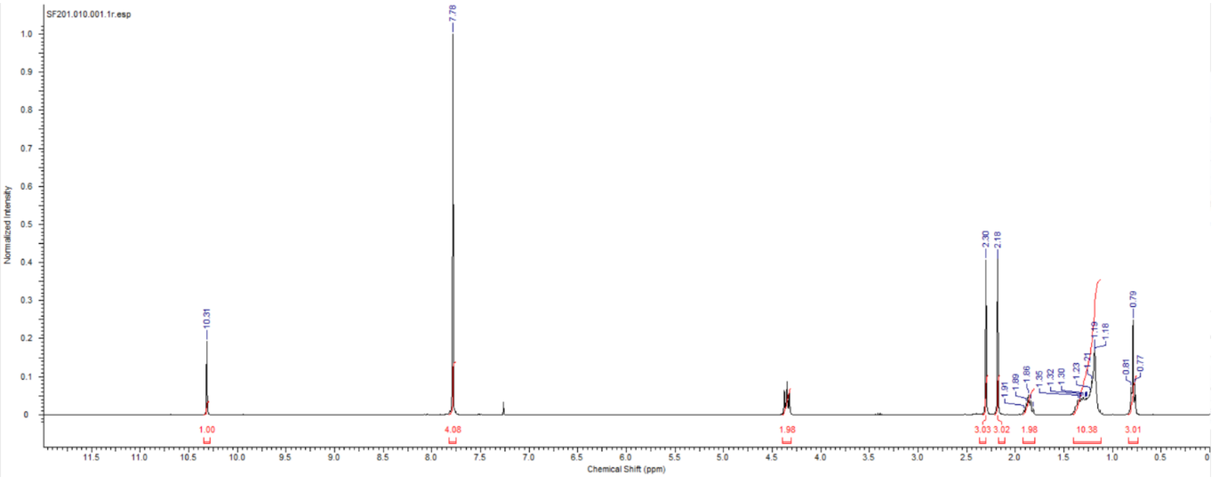

Figure S83. ^{1}H NMR of compound **23**.

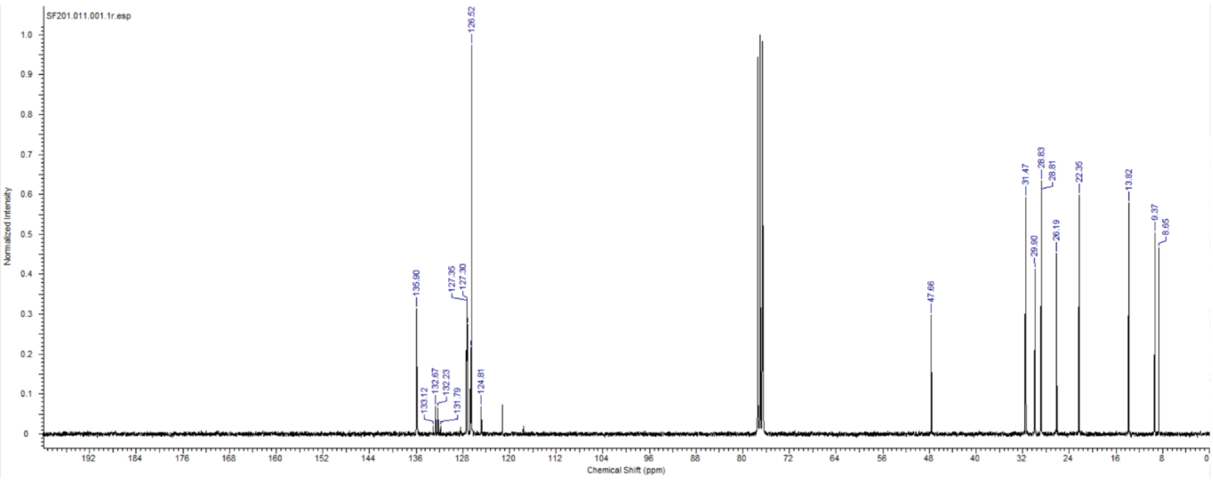

Figure S84. ^{13}C NMR of compound **23**.

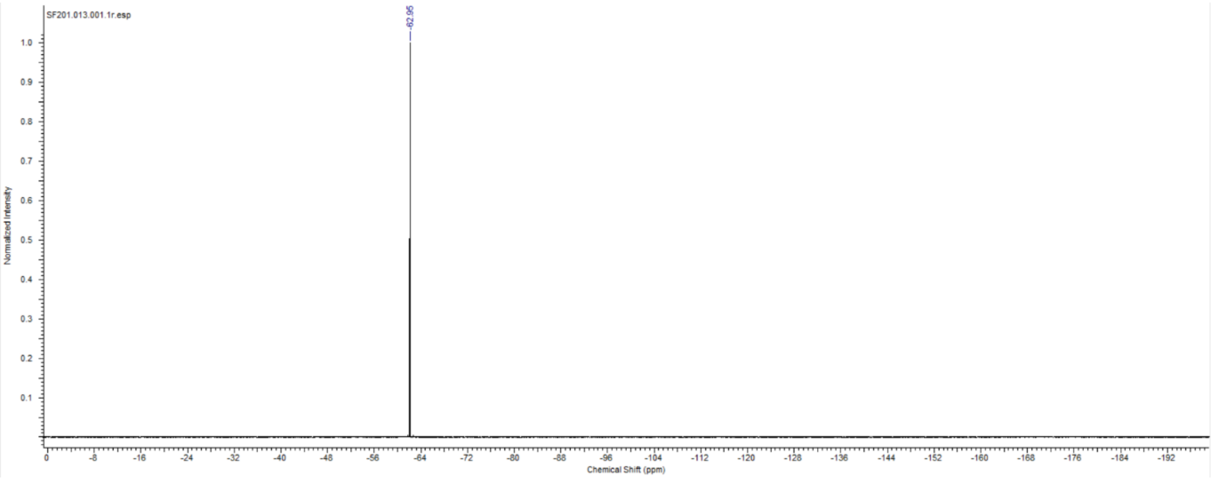

Figure S85. ^{19}F NMR of compound **23**.

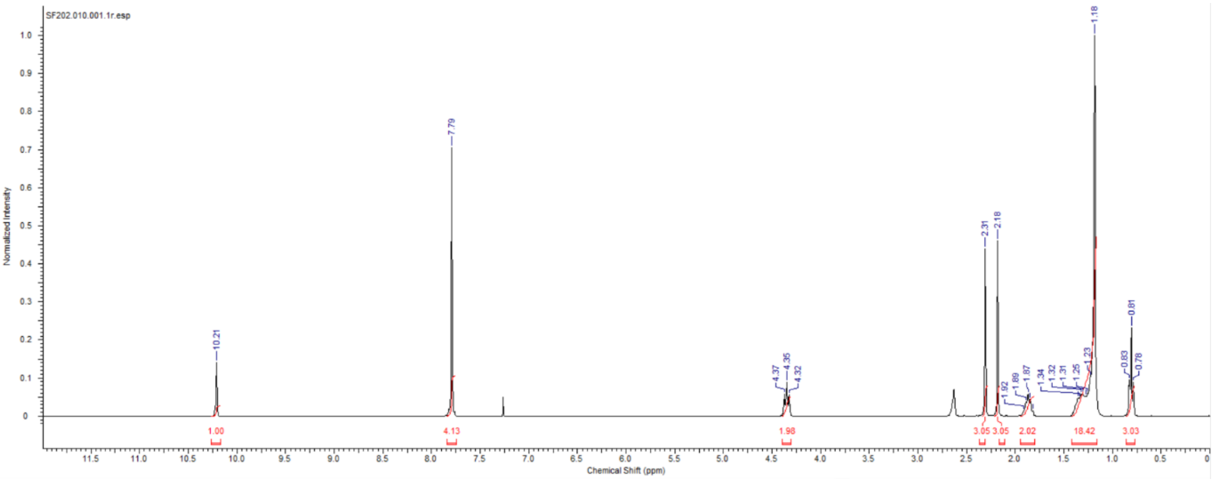

Figure S86. ^{1}H NMR of compound **24**.

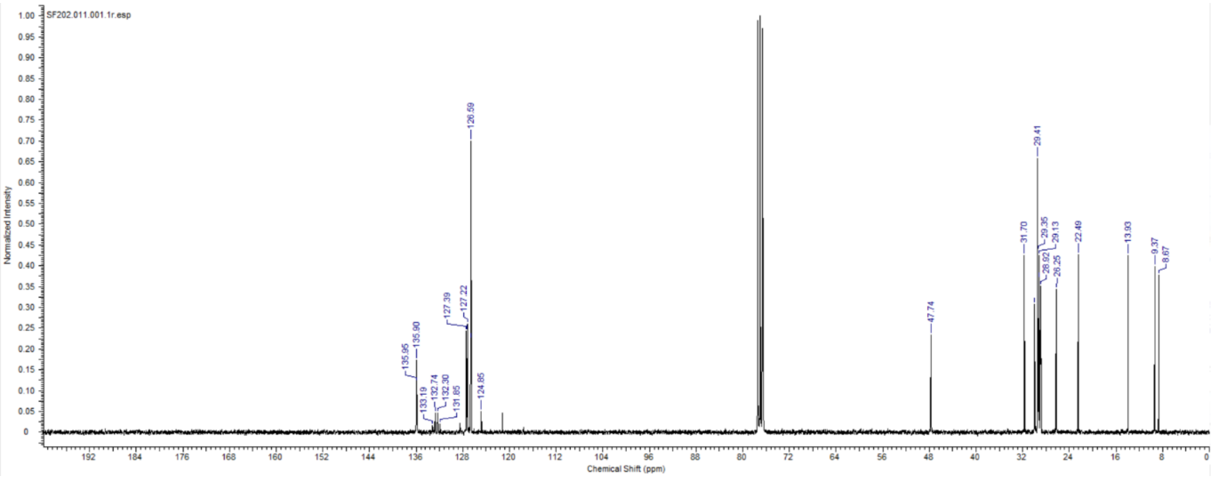

Figure S87. ^{13}C NMR of compound **24**.

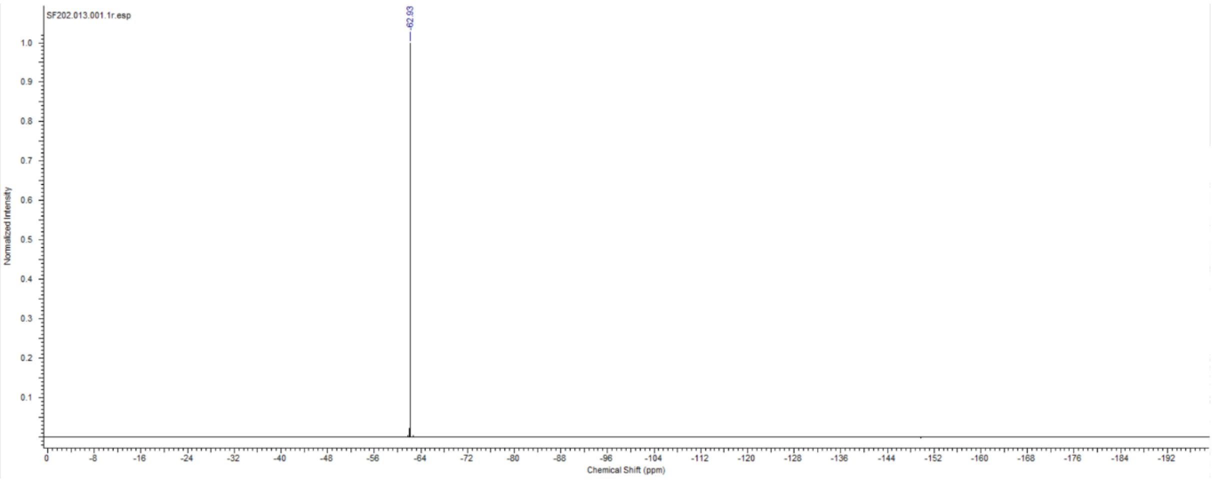

Figure S88. ^{19}F NMR of compound **24**.

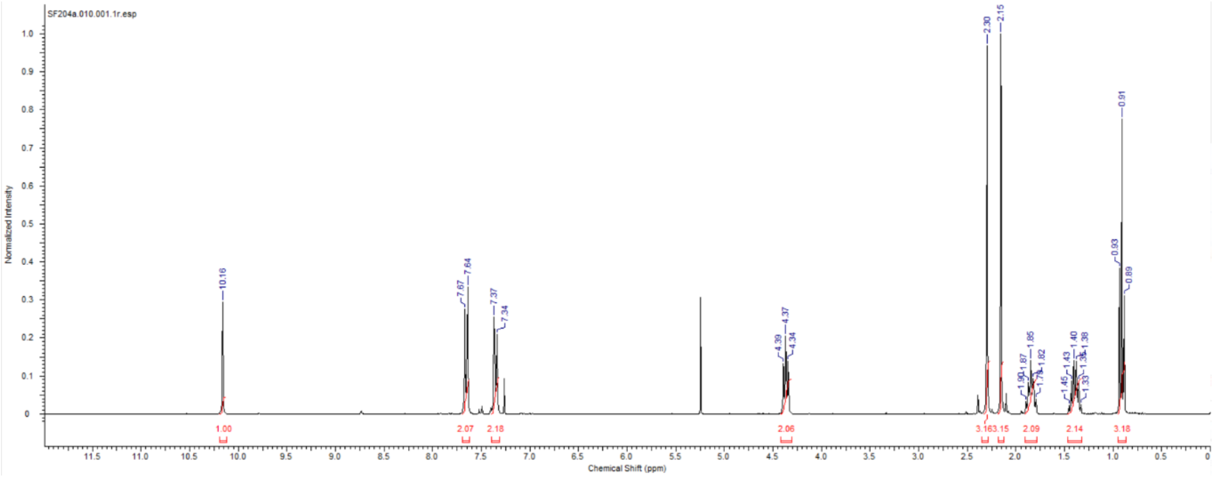

Figure S89. ^{1}H NMR of compound **25**.

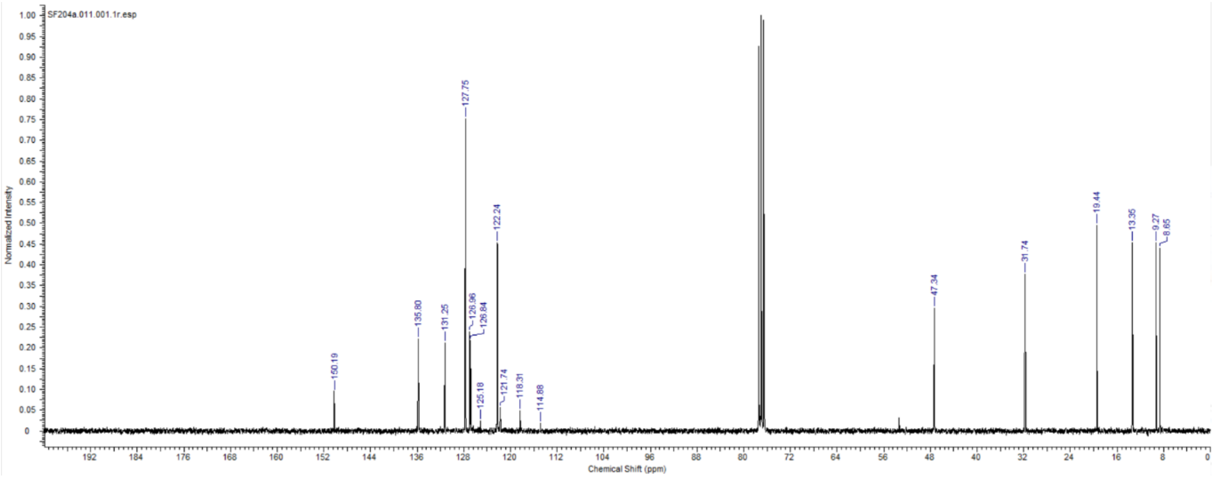

Figure S90. ^{13}C NMR of compound **25**.

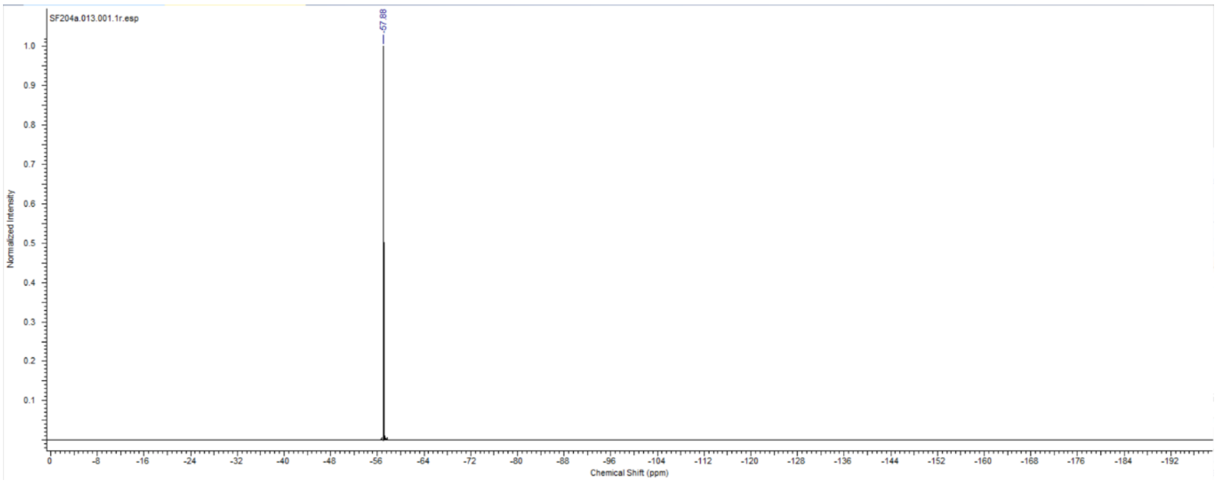

Figure S91. ^{19}F NMR of compound **25**.

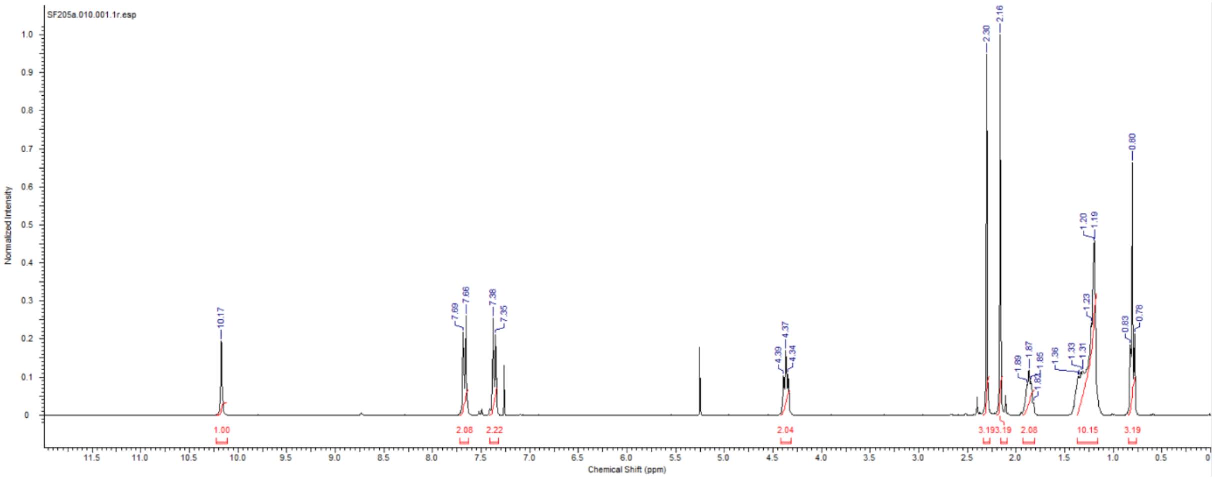

Figure S92. ^{1}H NMR of compound **26**.

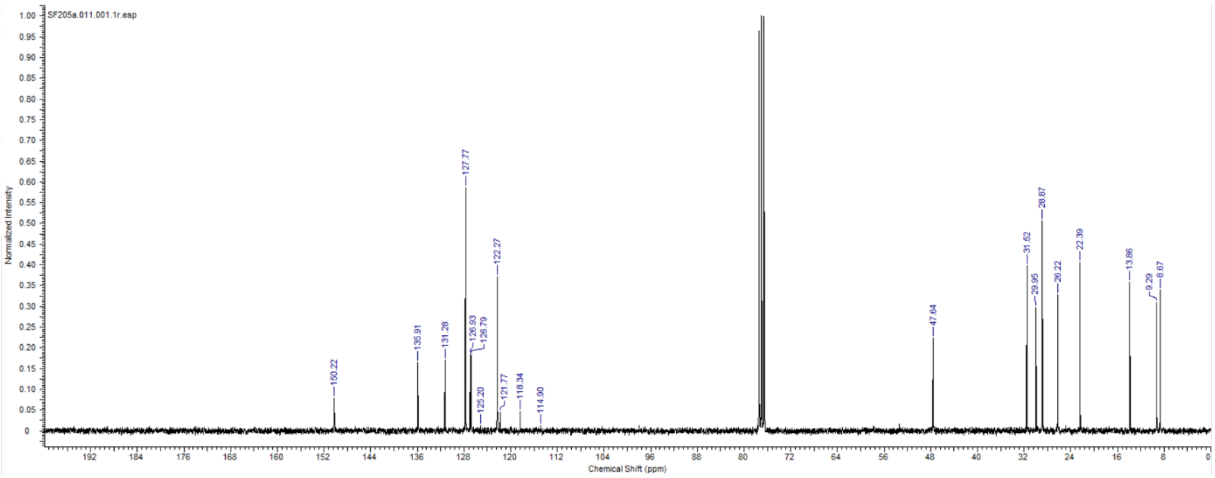

Figure S93. ^{13}C NMR of compound **26**.

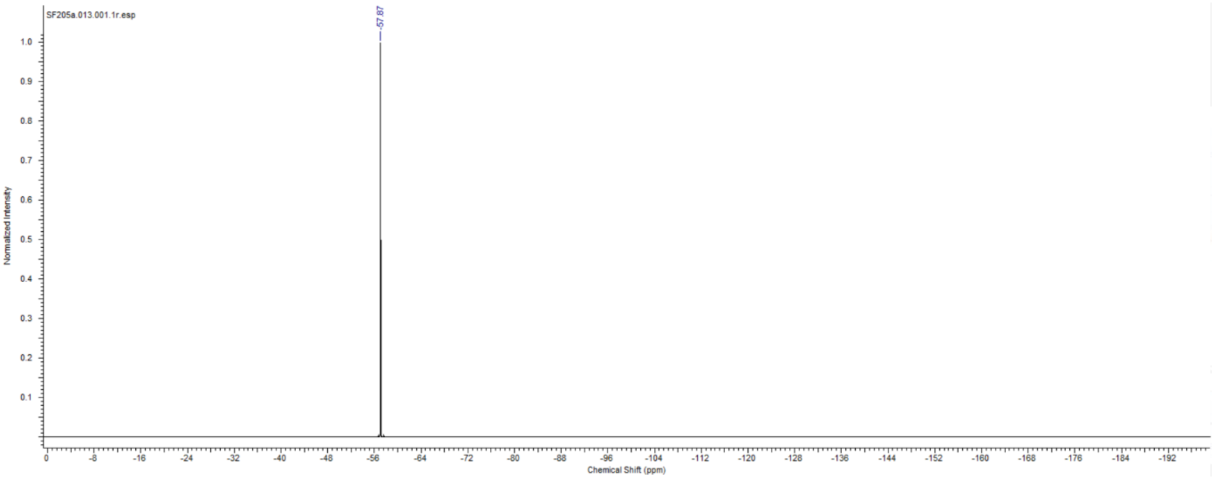

Figure S94. ^{19}F NMR of compound **26**.

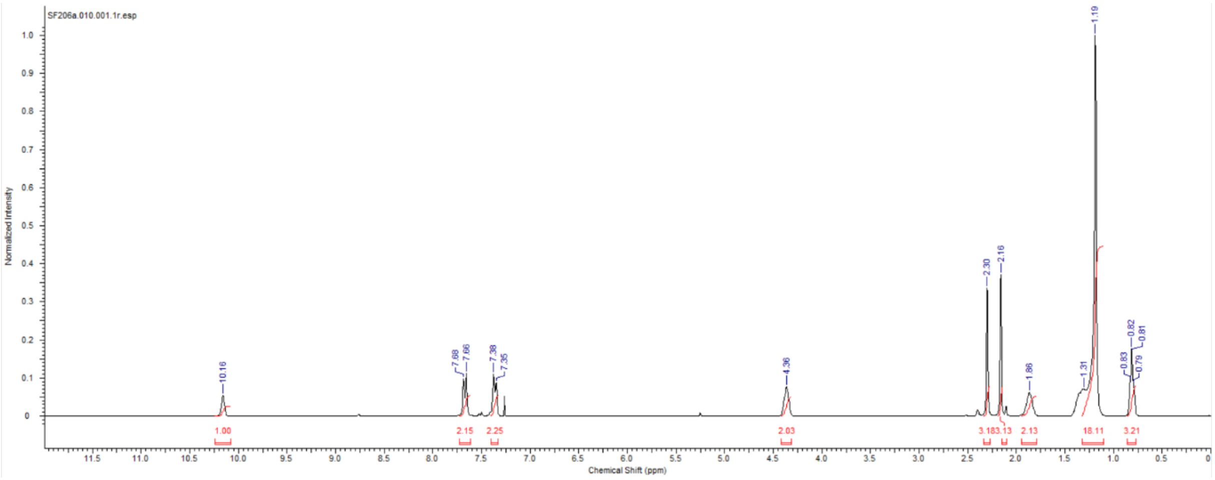

Figure S95. ^{1}H NMR of compound **27**.

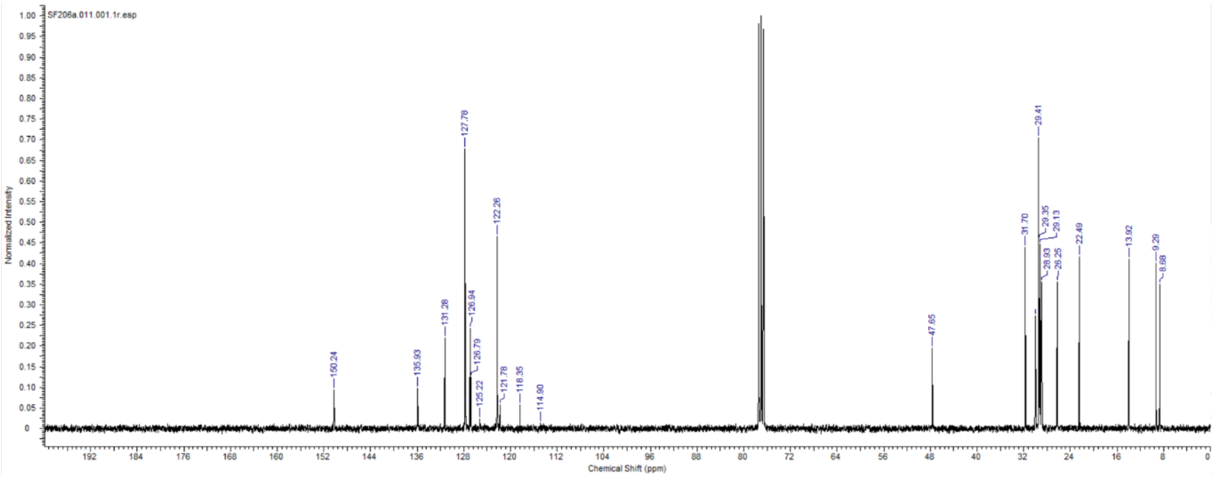

Figure S96. ^{13}C NMR of compound **27**.

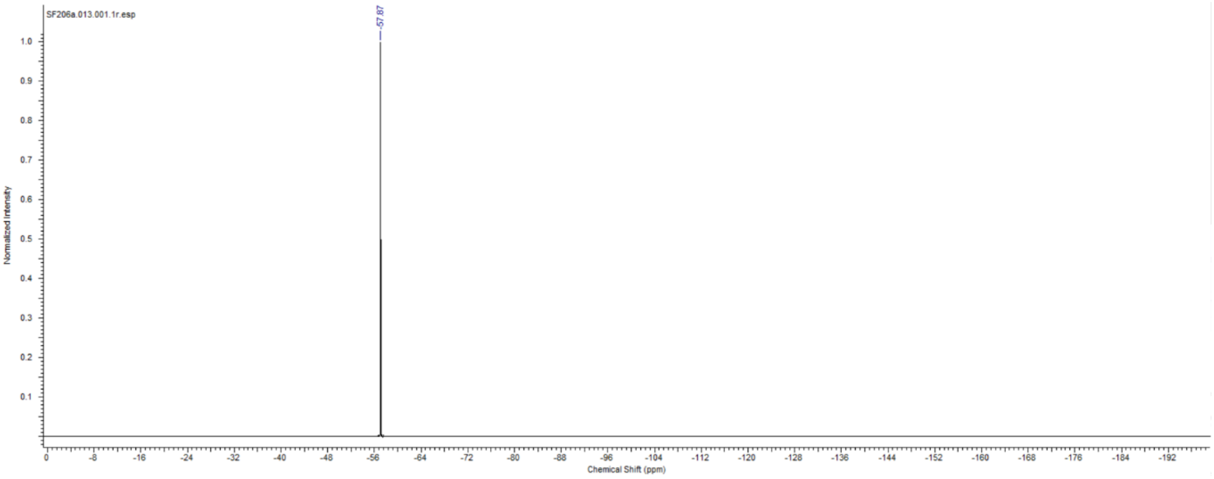

Figure S97. ^{19}F NMR of compound **27**.

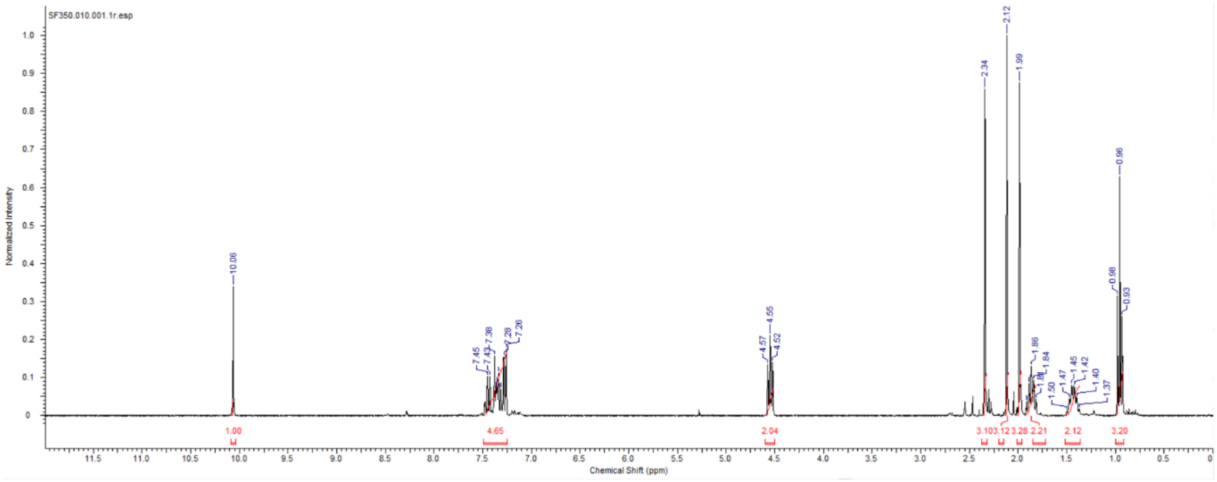

Figure S98. ^{1}H NMR of compound **28.**

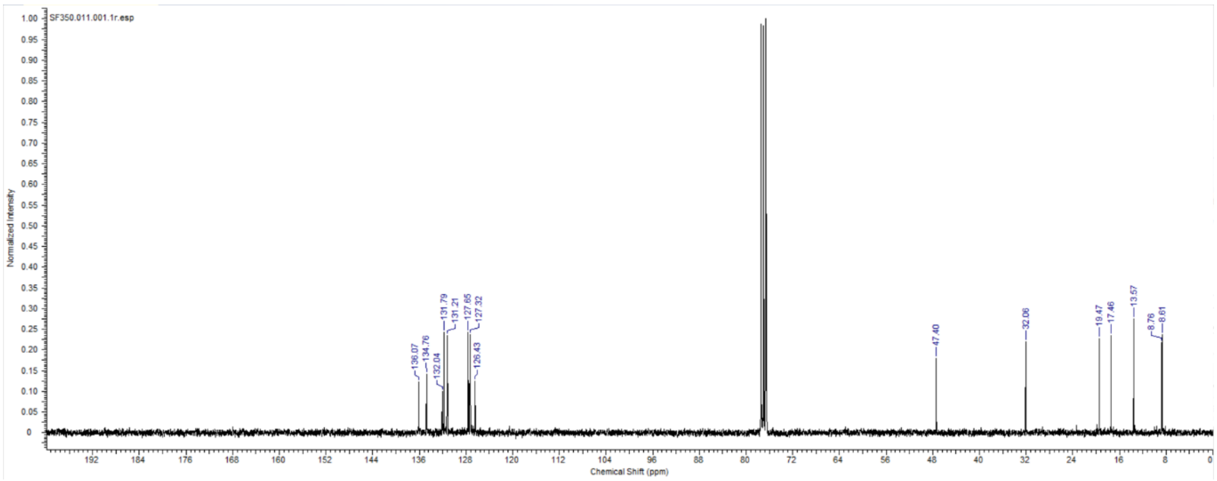

Figure S99. ^{13}C NMR of compound **28**.

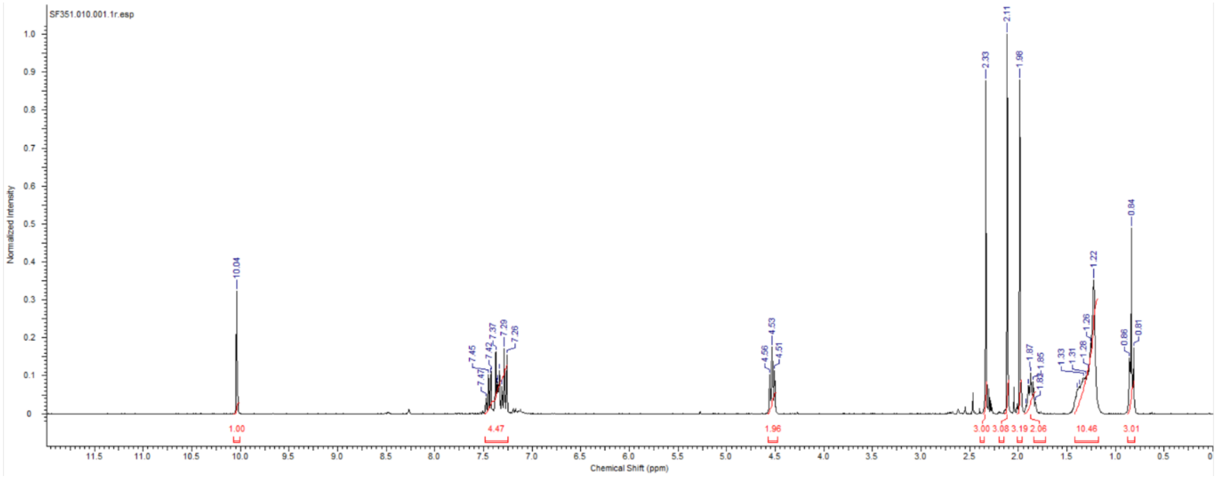

Figure S100. ^{1}H NMR of compound **29**.

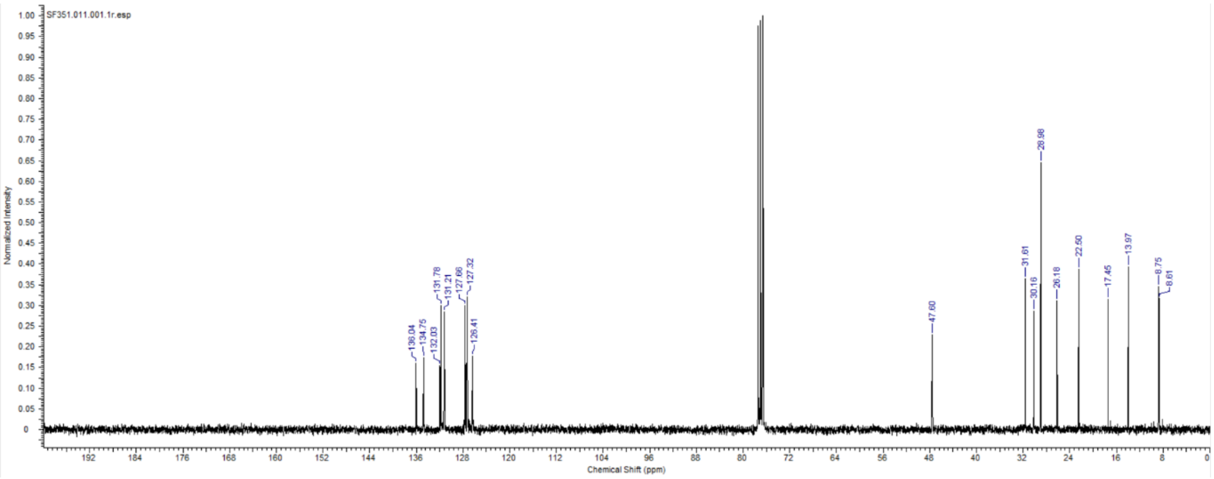

Figure S101. ^{13}C NMR of compound **29**.

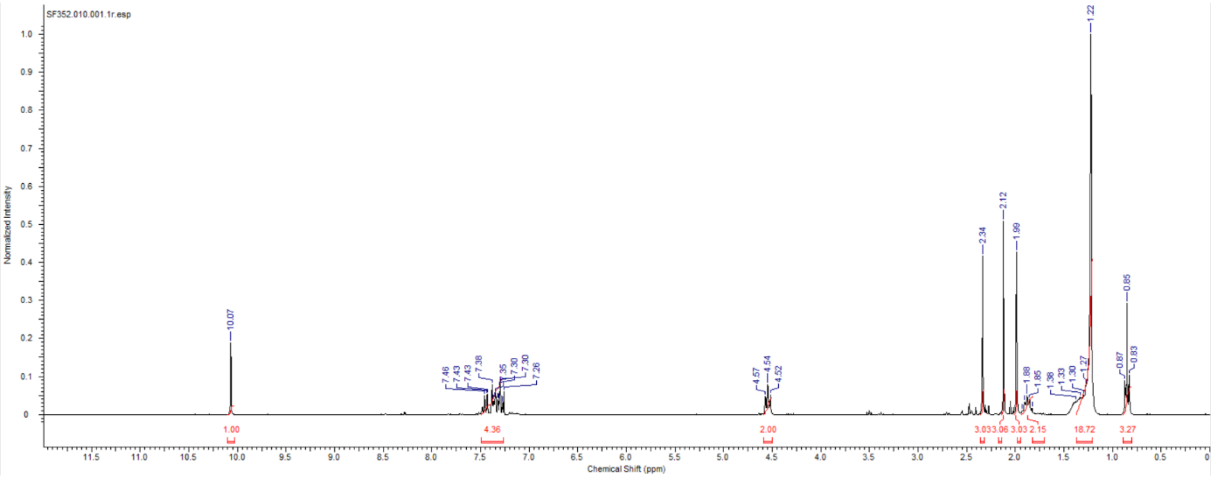

Figure S102. ^{1}H NMR of compound **30**.

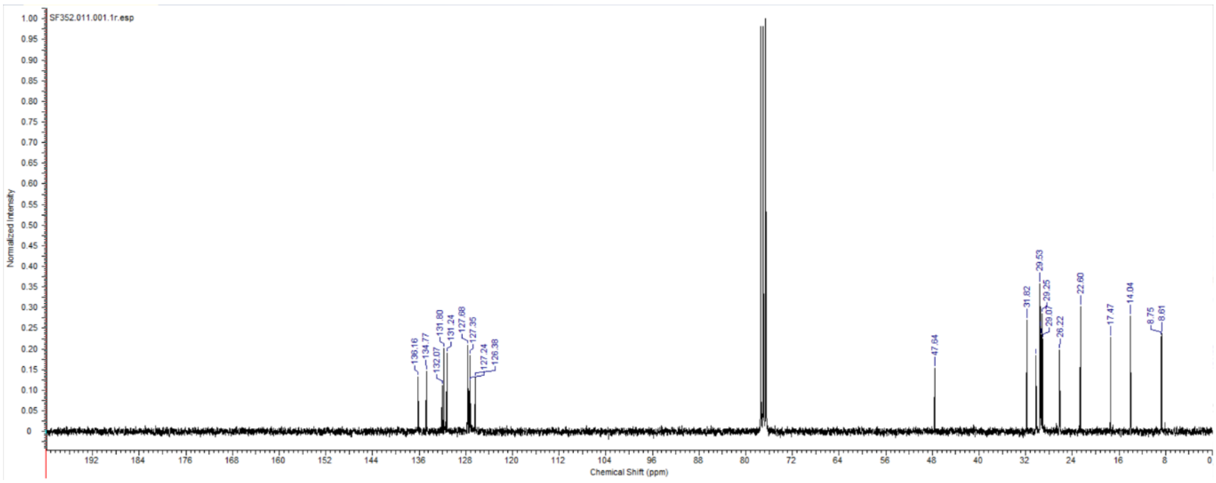

Figure S103. ^{13}C NMR of compound **30**.

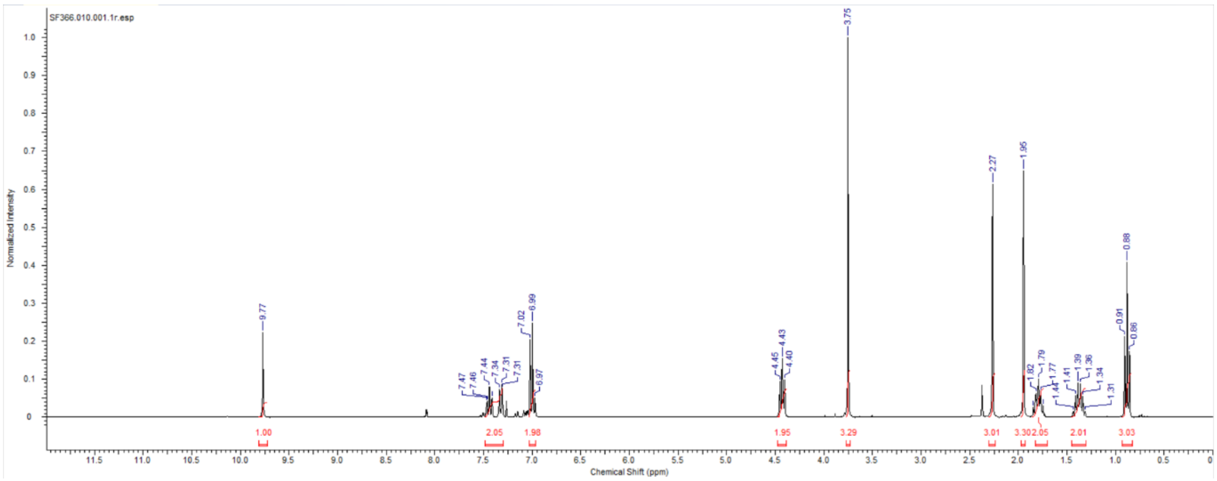

Figure S104. ^{1}H NMR of compound **31**.

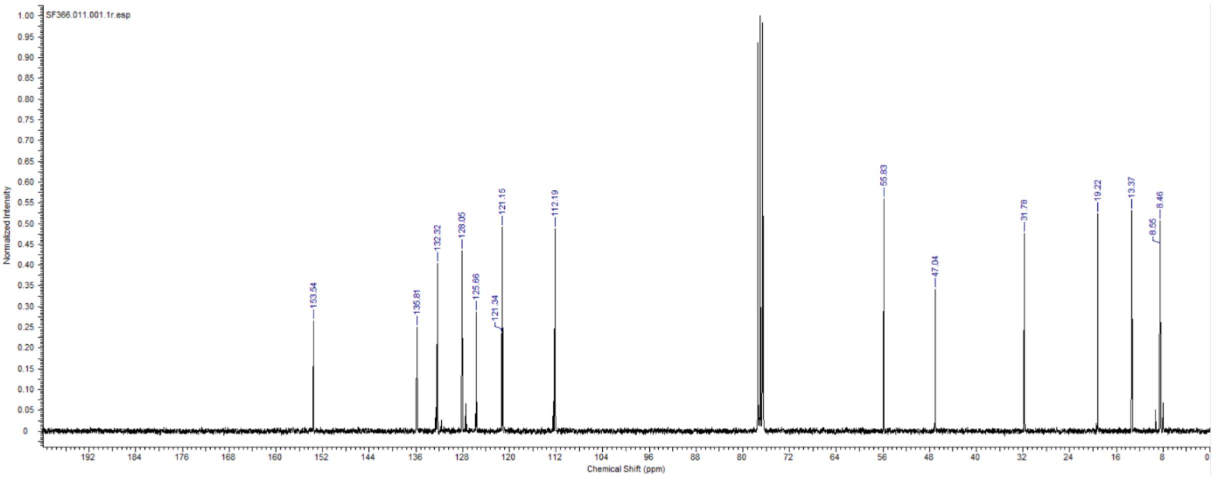

Figure S105. ^{13}C NMR of compound **31**.

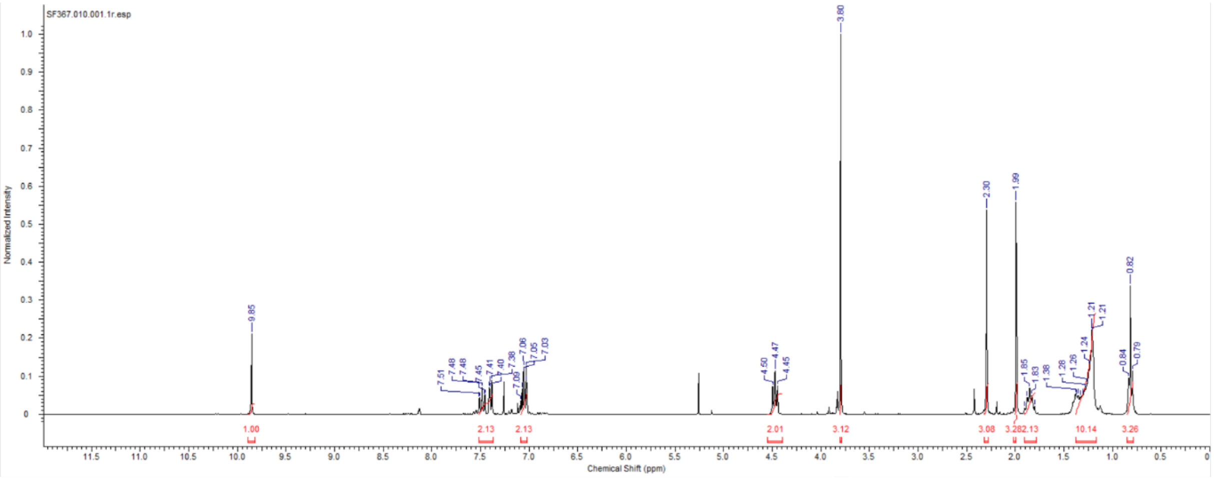

Figure S106. ^{1}H NMR of compound **32**.

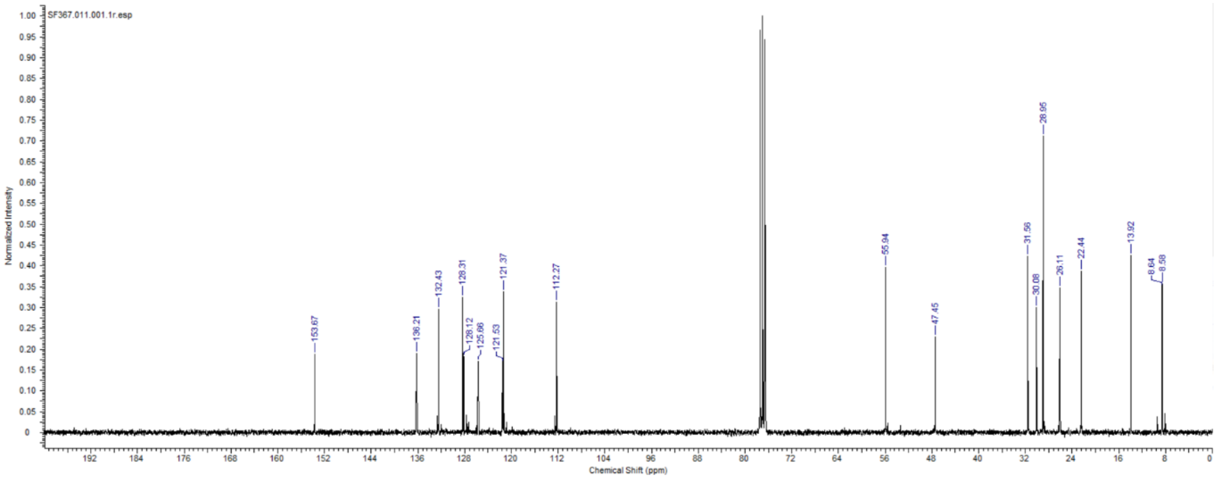

Figure S107. ^{13}C NMR of compound **32**.

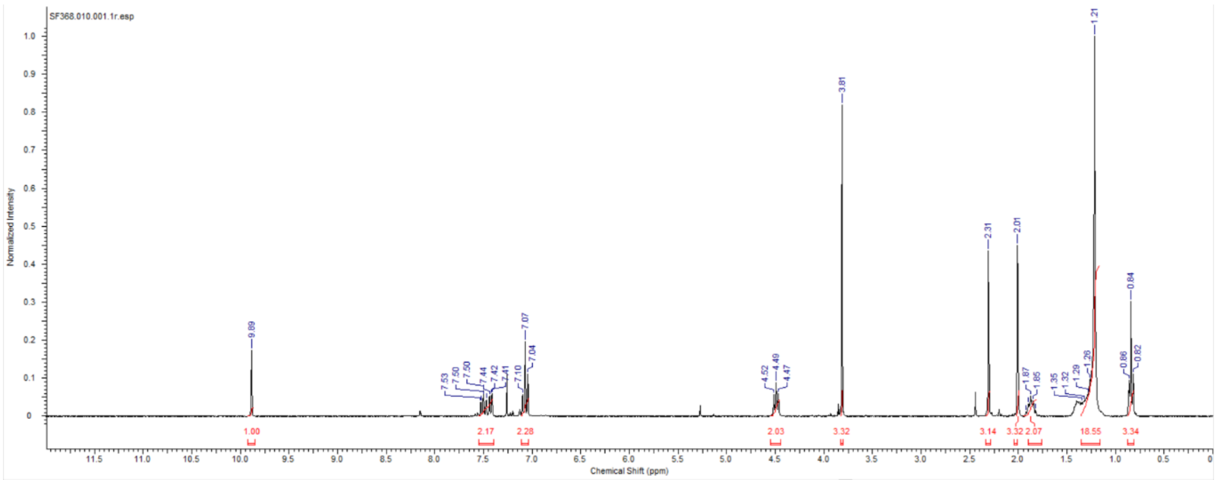

Figure S108. ^{1}H NMR of compound **33**.

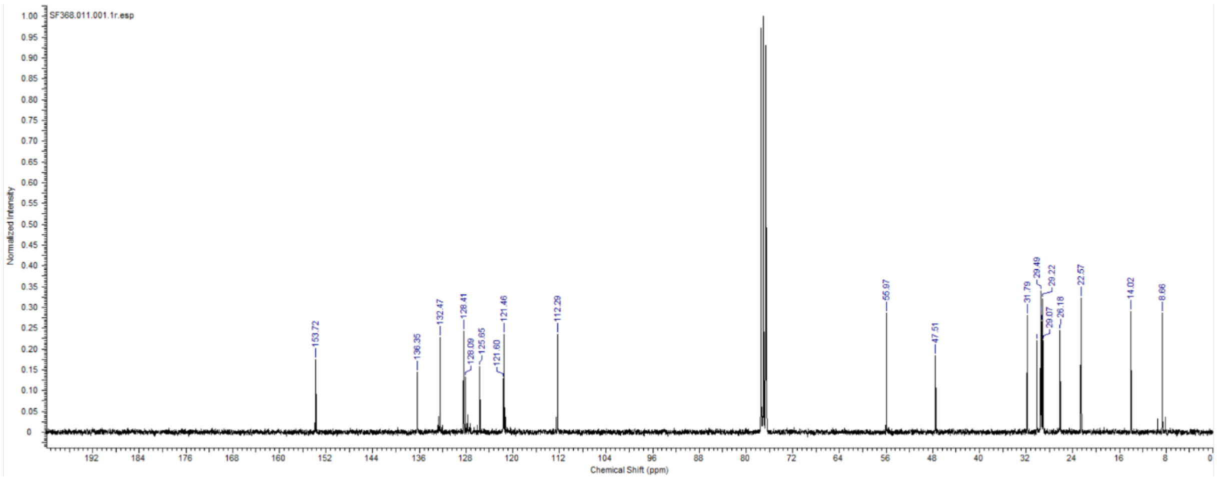

Figure S109. ^{13}C NMR of compound **33**.

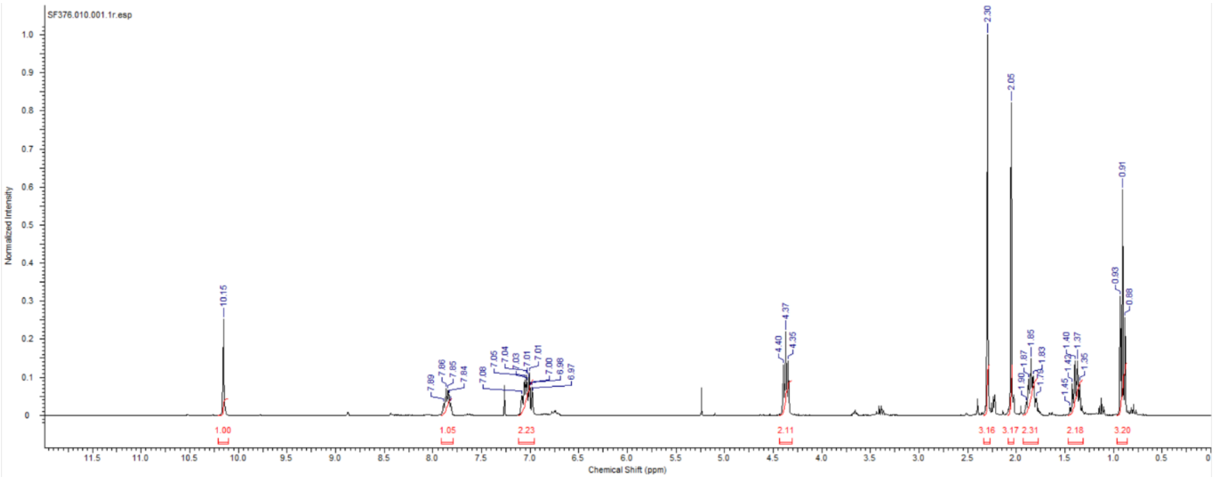

Figure S110. ^{1}H NMR of compound **34**.

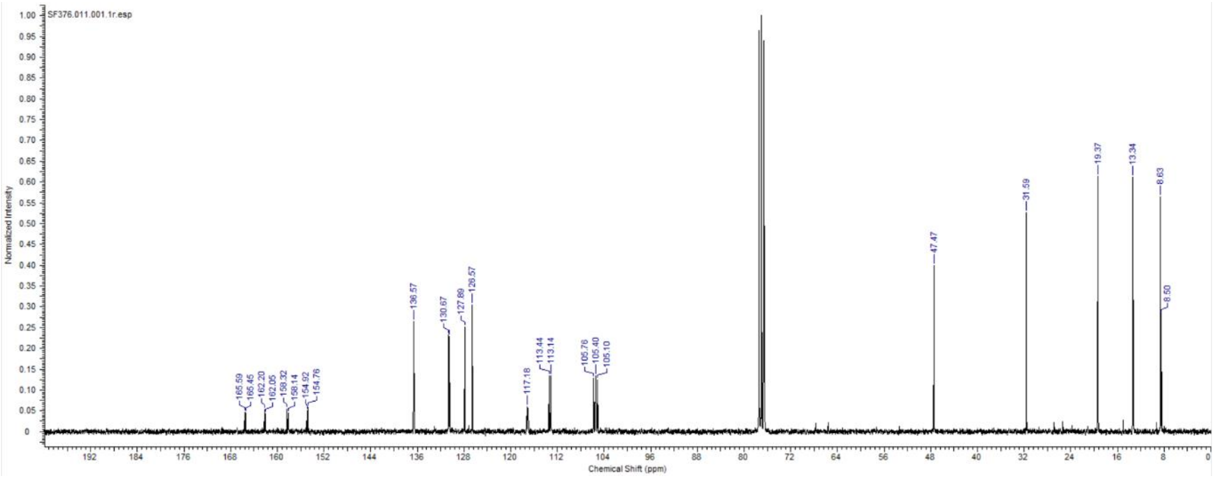

Figure S111. ^{13}C NMR of compound **34**.

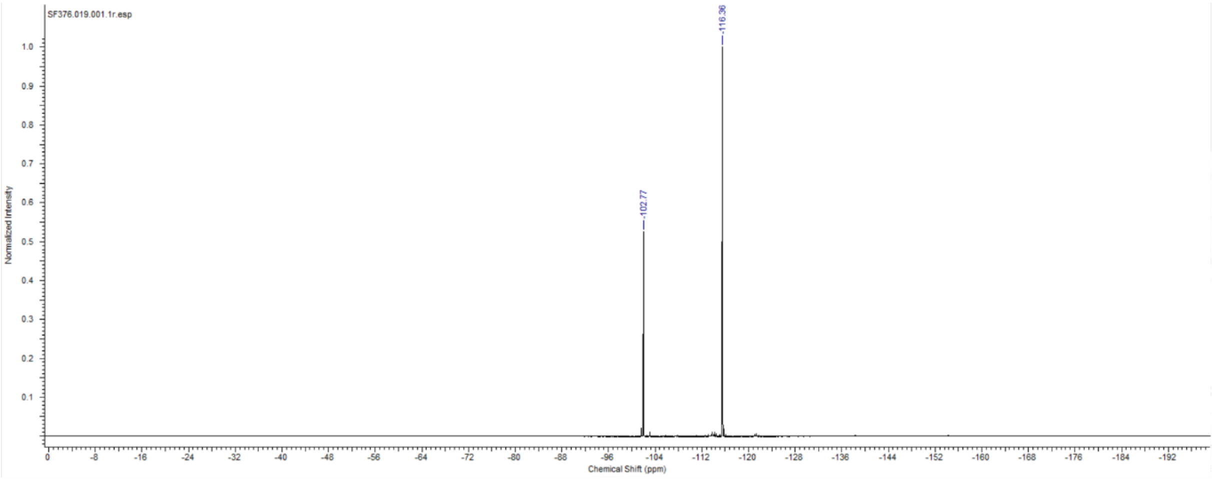

Figure S112. ^{19}F NMR of compound **34**.

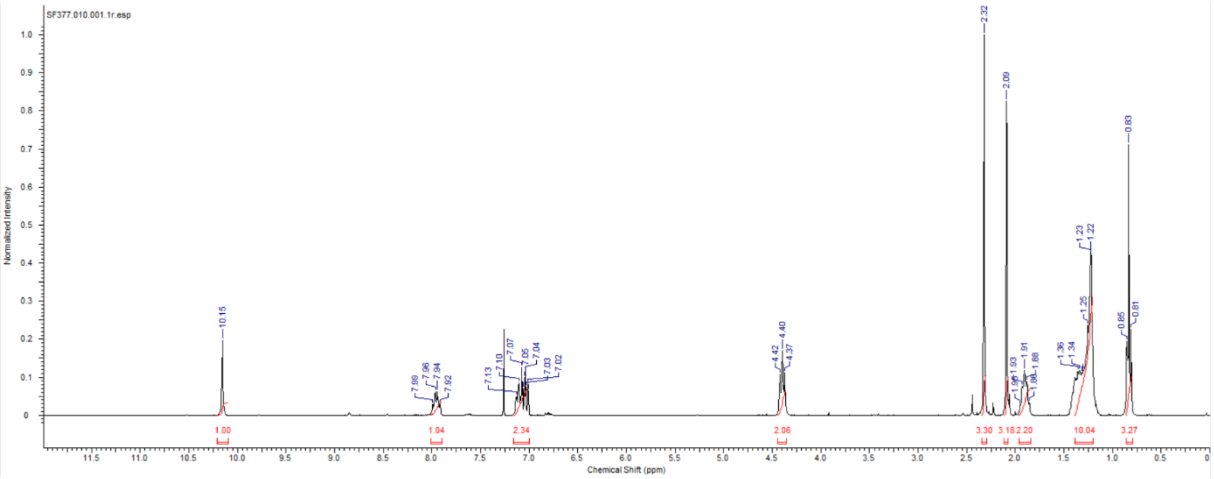

Figure S113. ^{1}H NMR of compound **35**.

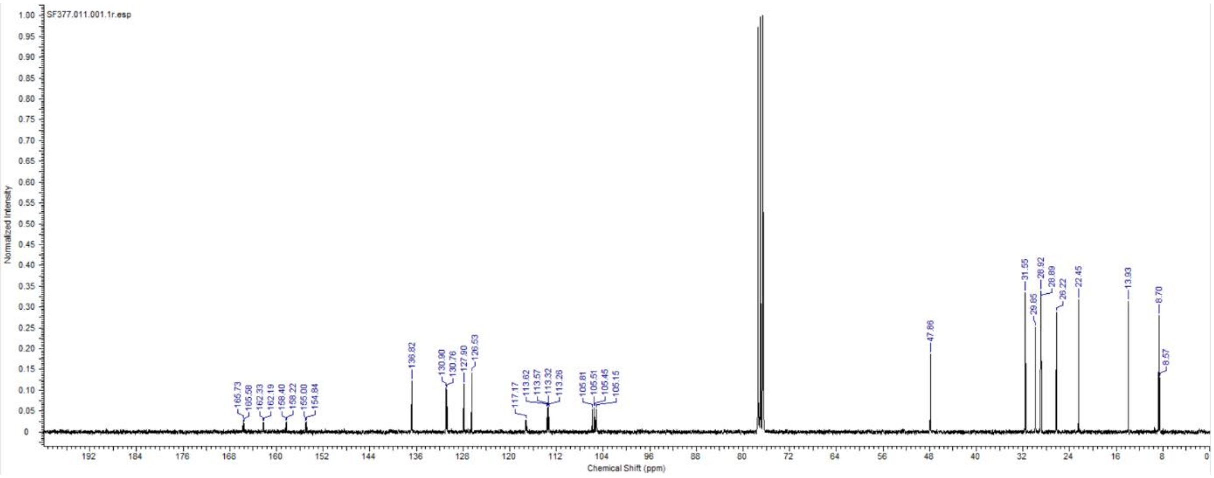

Figure S114. ^{13}C NMR of compound **35**.

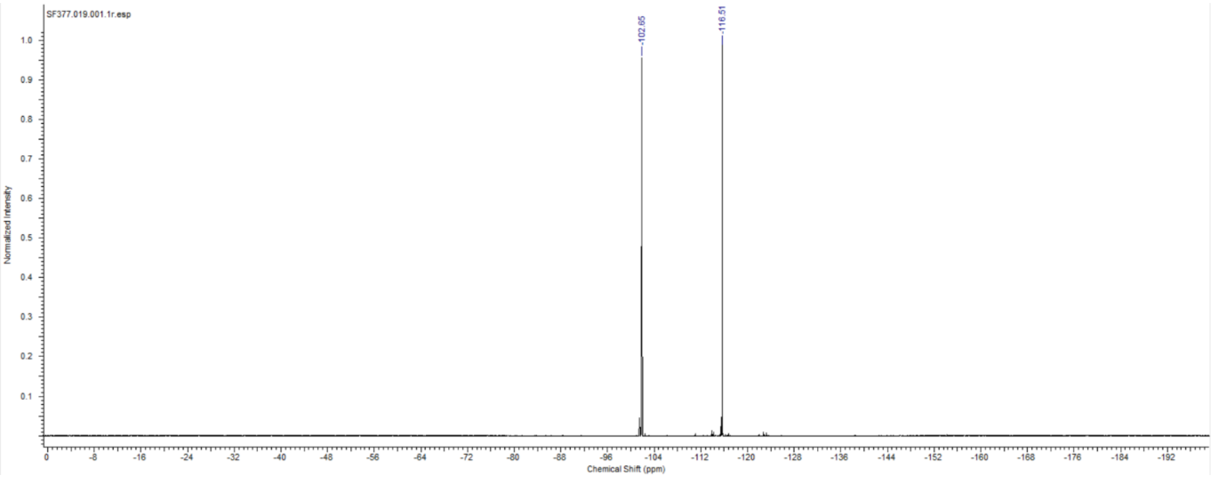

Figure S115. ^{19}F NMR of compound **35**.

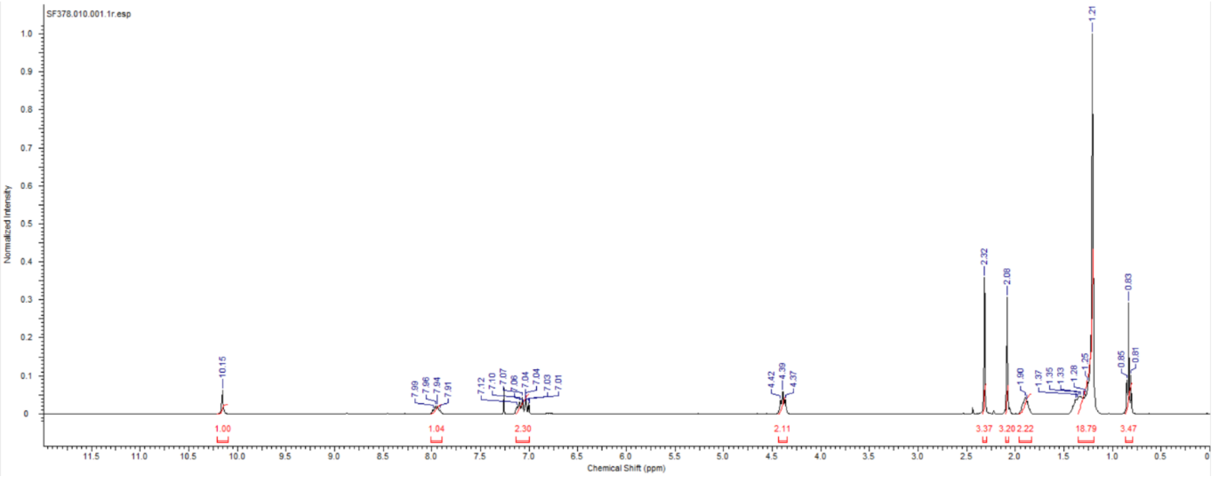

Figure S116. ^{1}H NMR of compound **36**.

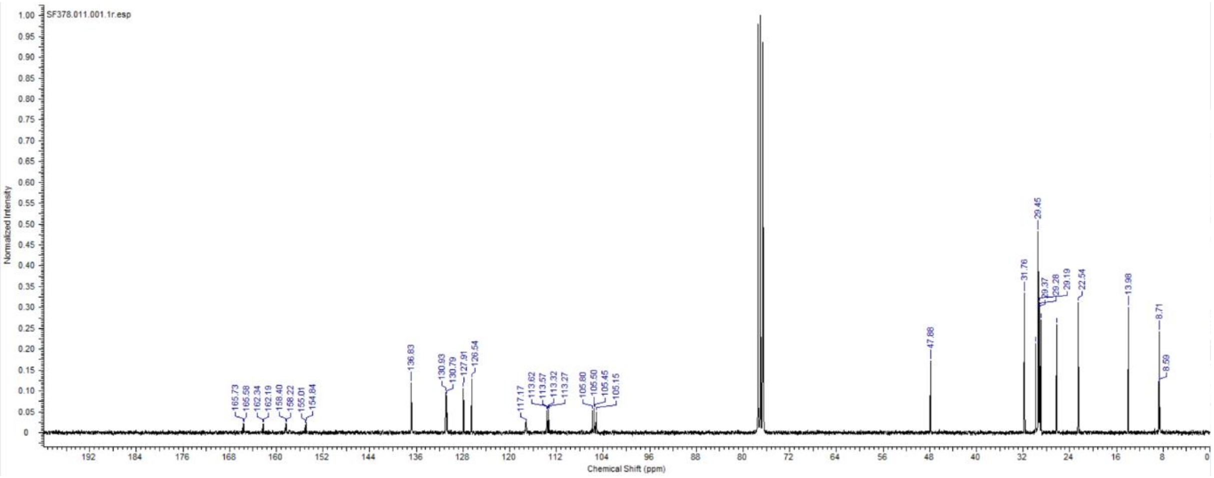

Figure S117. ^{13}C NMR of compound **36**.

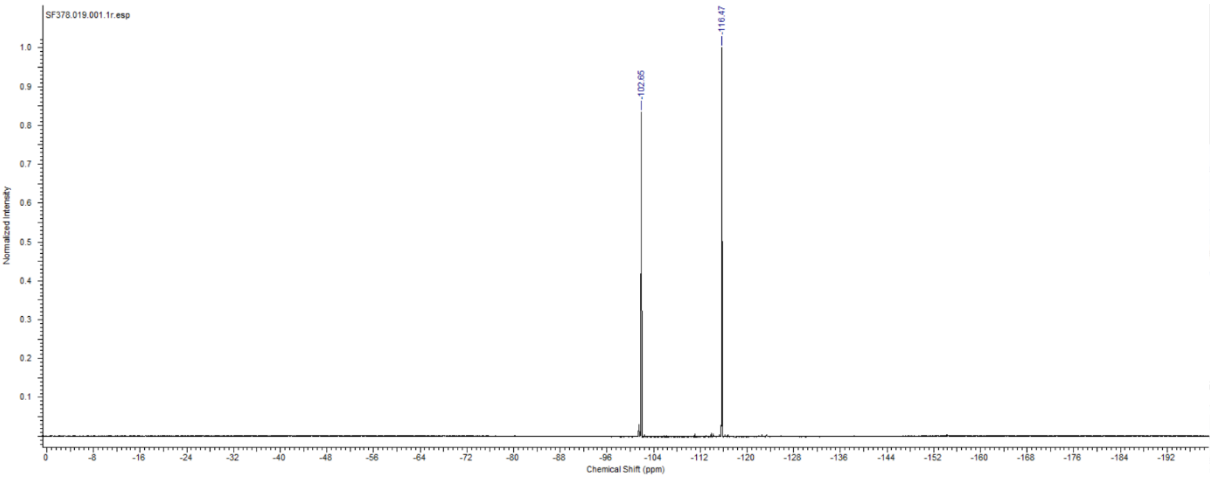

Figure S118. ^{19}F NMR of compound **36**.

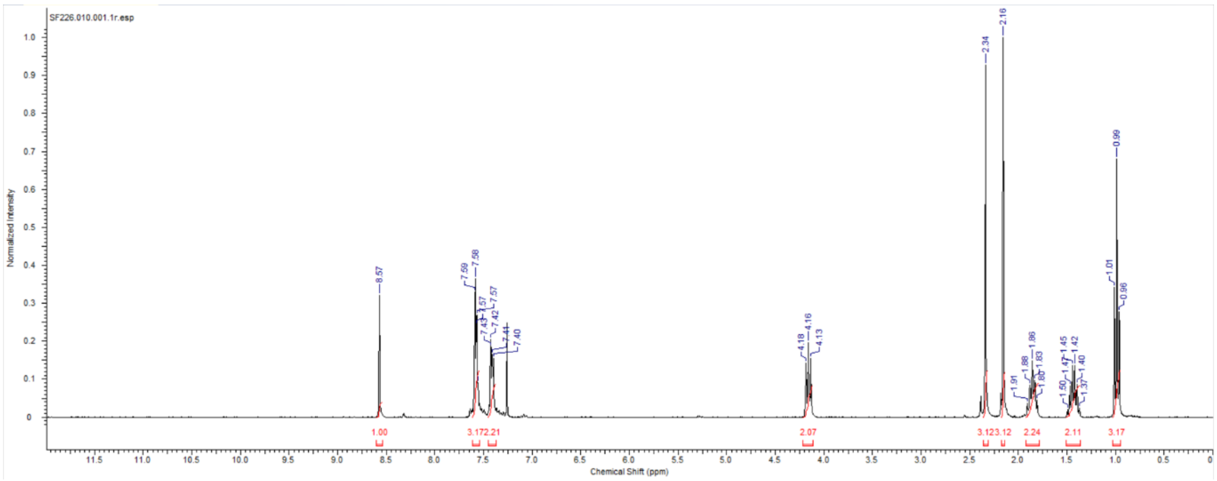

Figure S119. ^{1}H NMR of compound **37**.

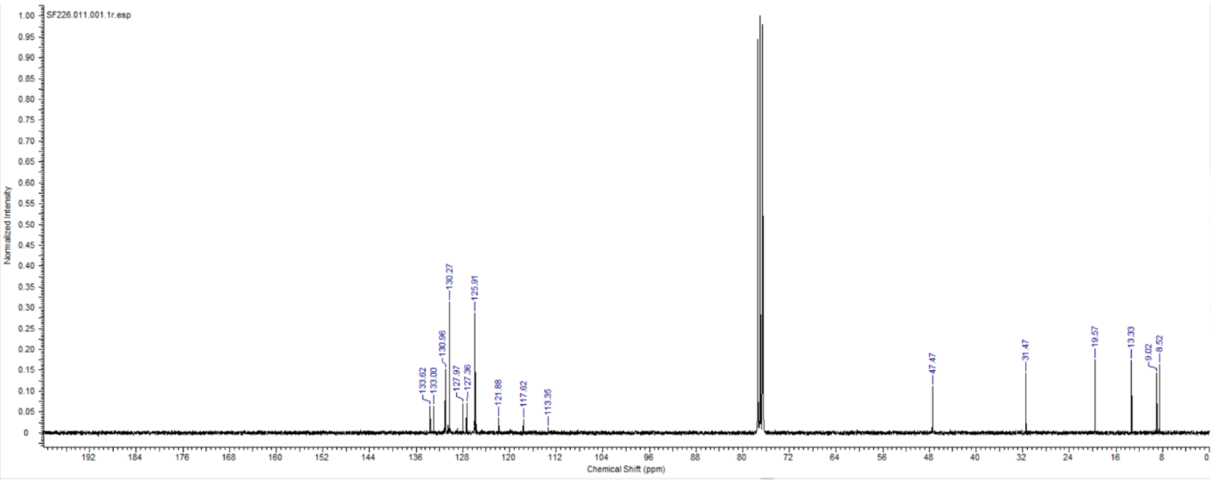

Figure S120. ^{13}C NMR of compound **37**.

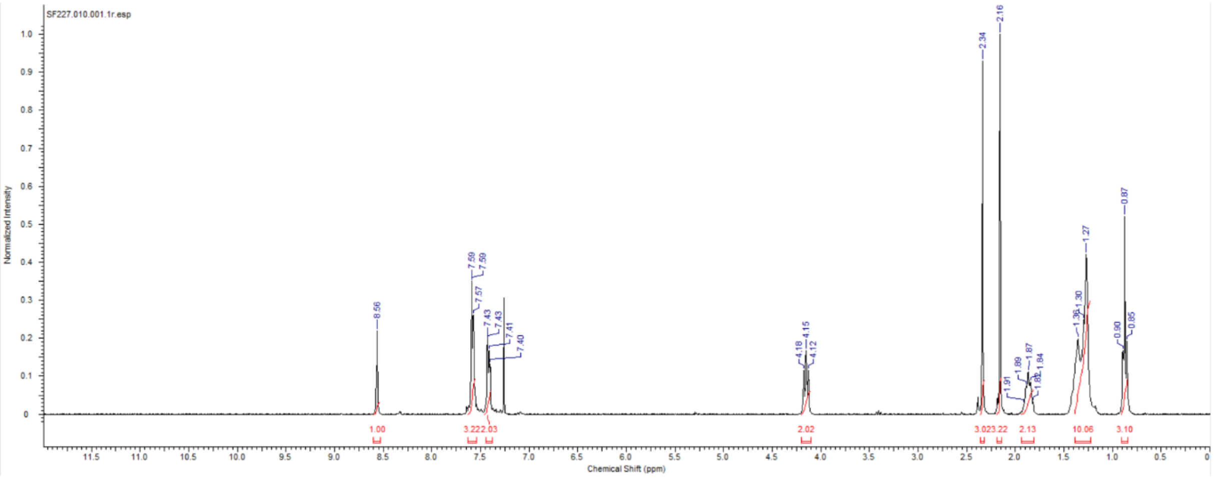

Figure S121. ^{1}H NMR of compound **38**.

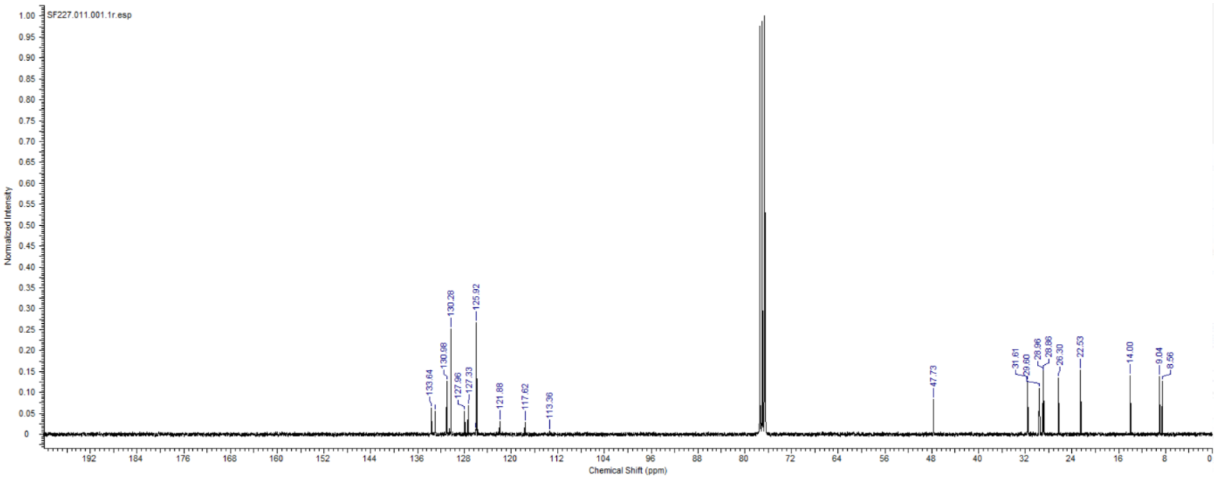

Figure S122. ^{13}C NMR of compound **38**.

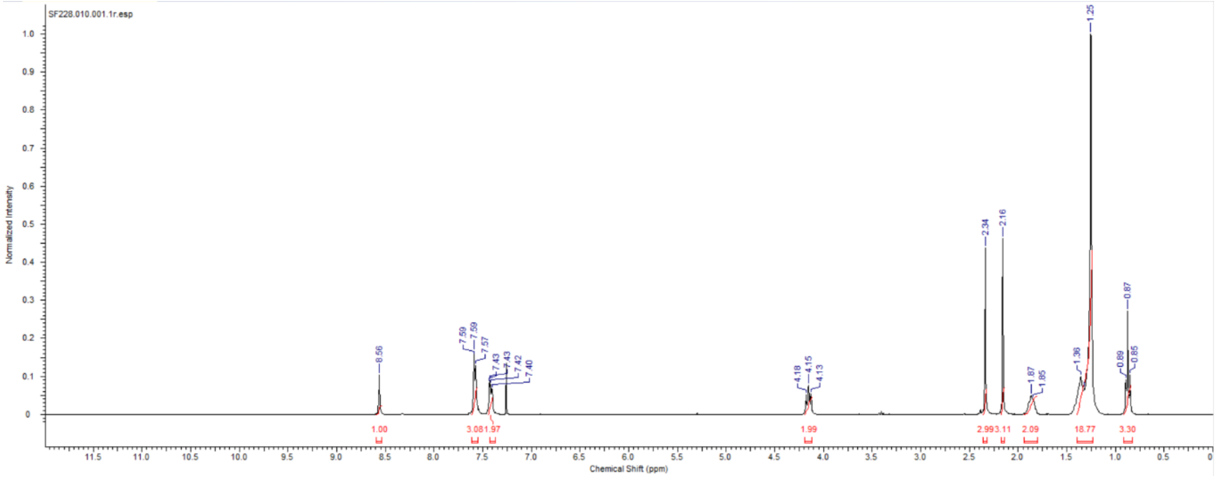

Figure S121. ^{1}H NMR of compound **39**.

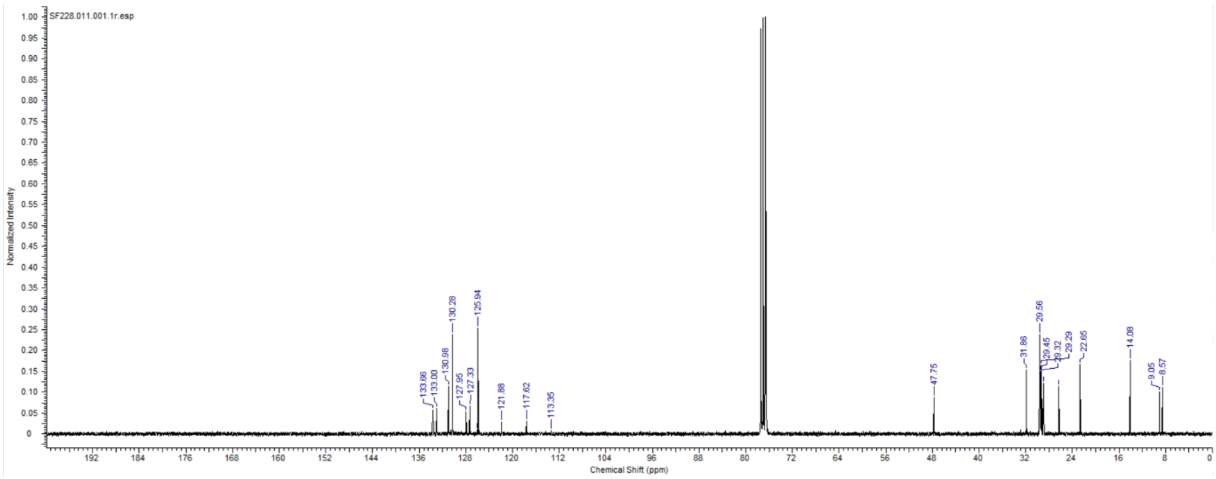

Figure S122. ^{13}C NMR of compound **39**.

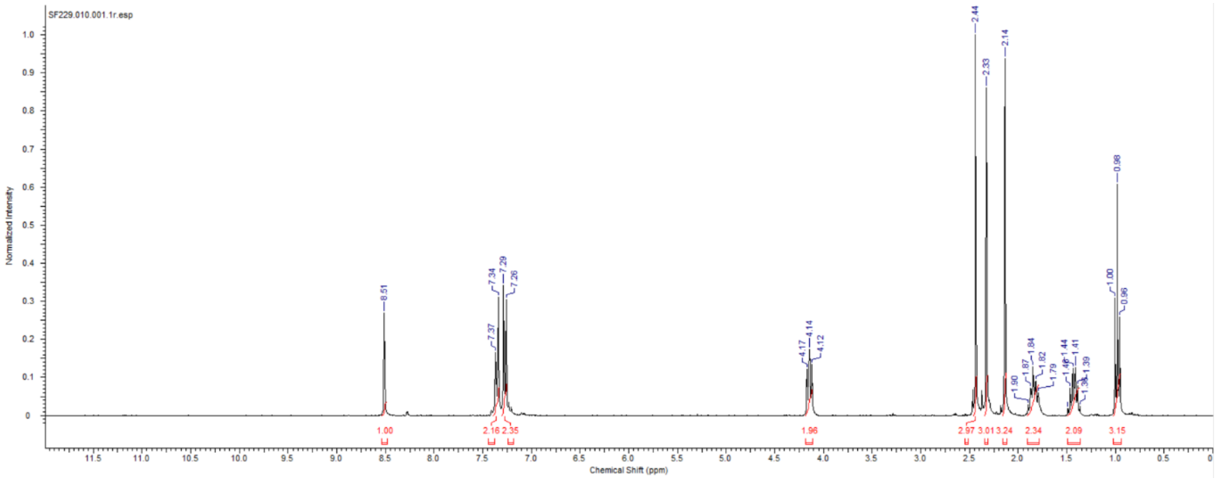

Figure S123. ^{1}H NMR of compound **40**.

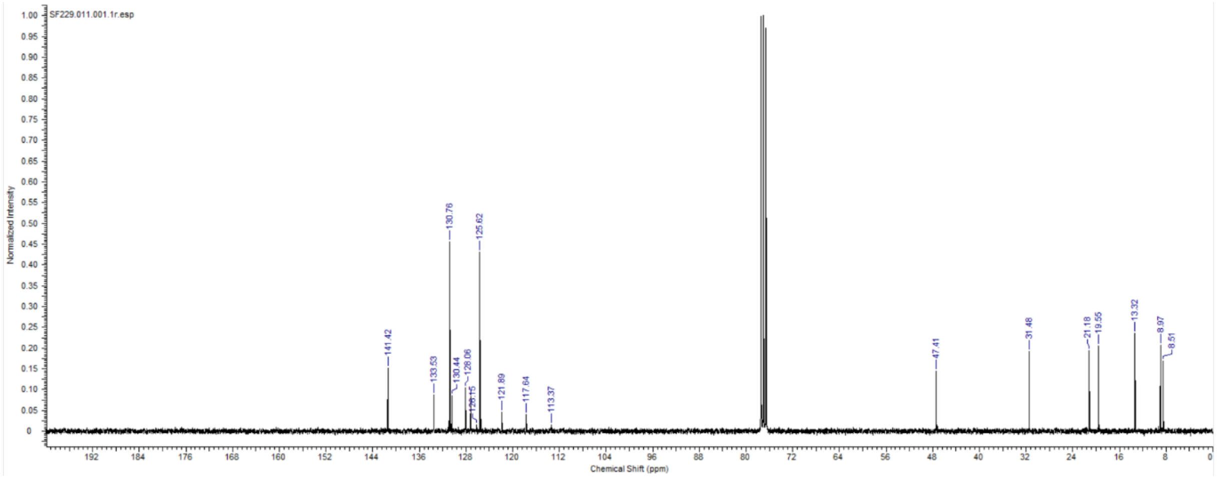

Figure S124. ^{13}C NMR of compound **40**.

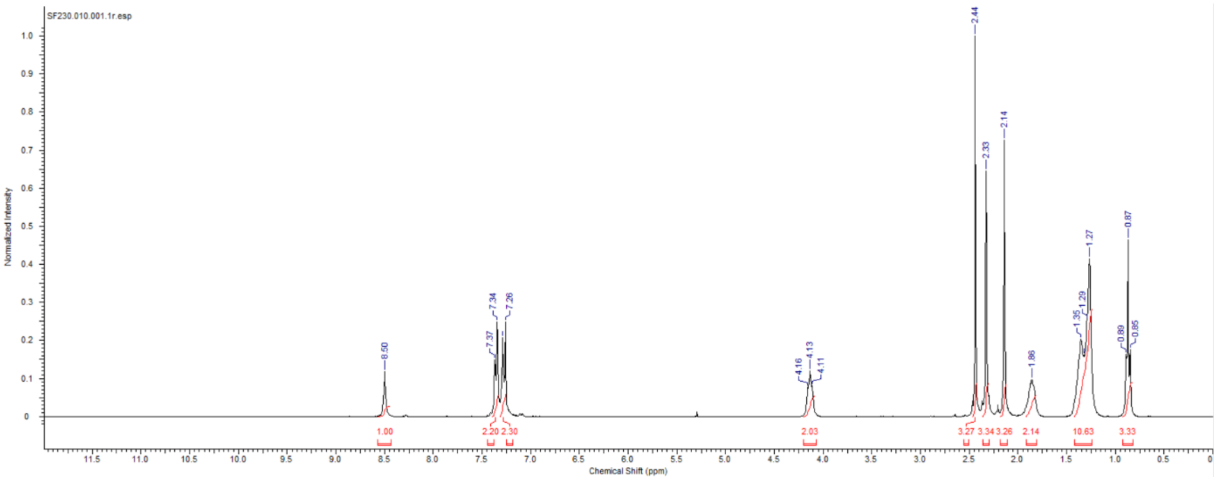

Figure S125. ^{1}H NMR of compound **41**.

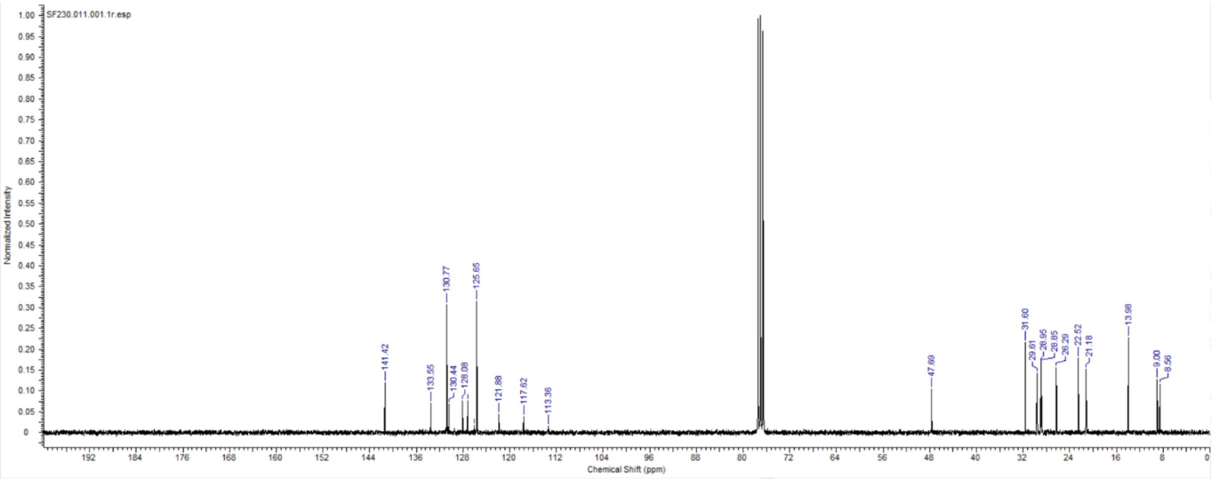

Figure S126. ^{13}C NMR of compound **41**.

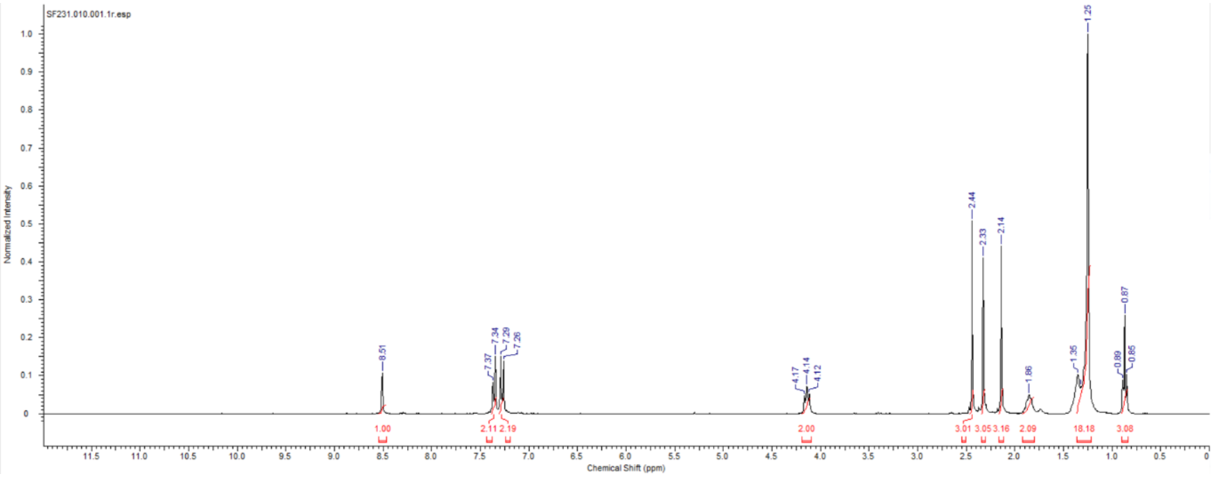

Figure S127. ^{1}H NMR of compound **42**.

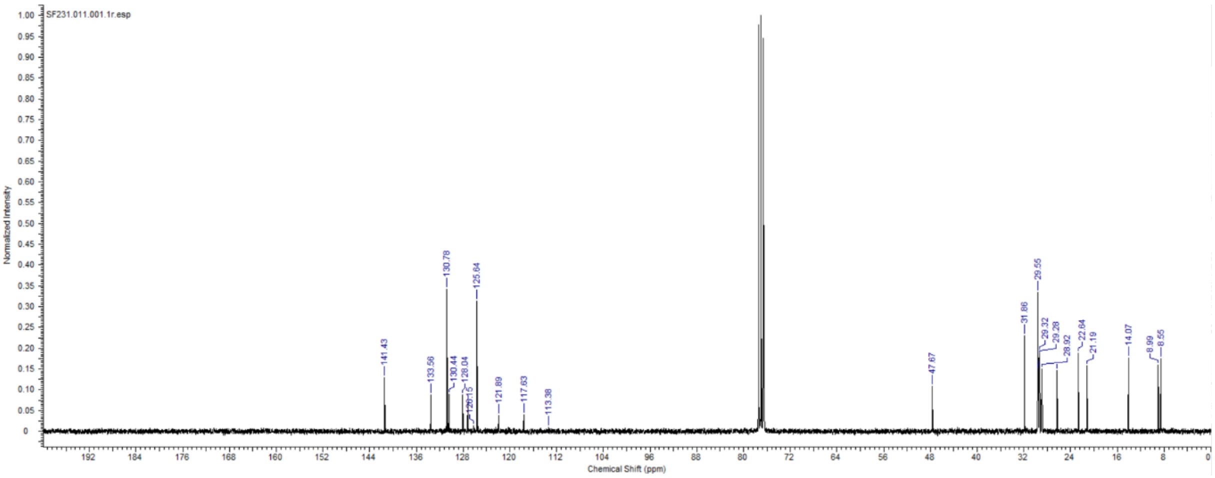

Figure S128. ^{13}C NMR of compound **42**.

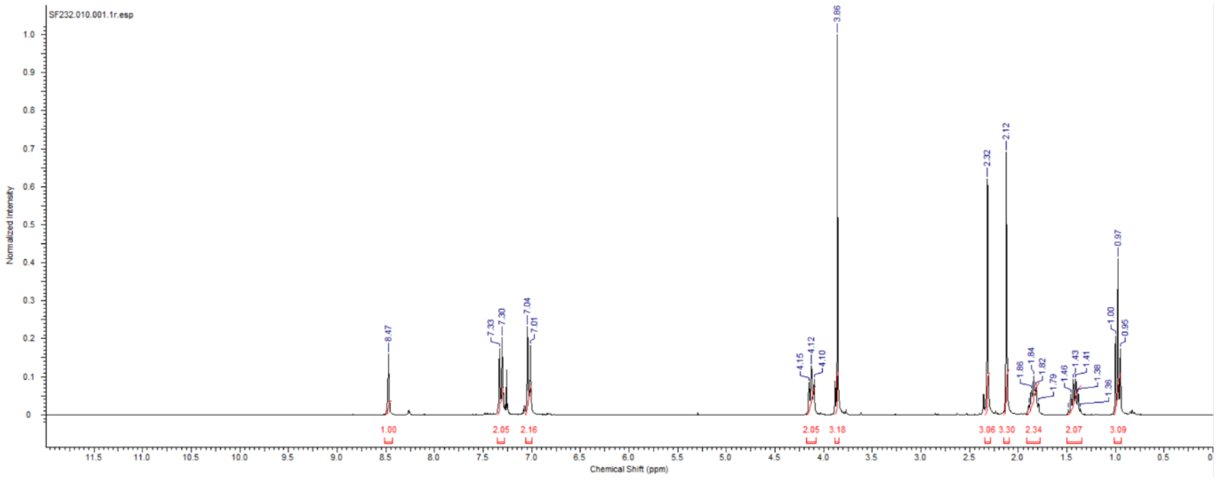

Figure S129. ^{1}H NMR of compound **43**.

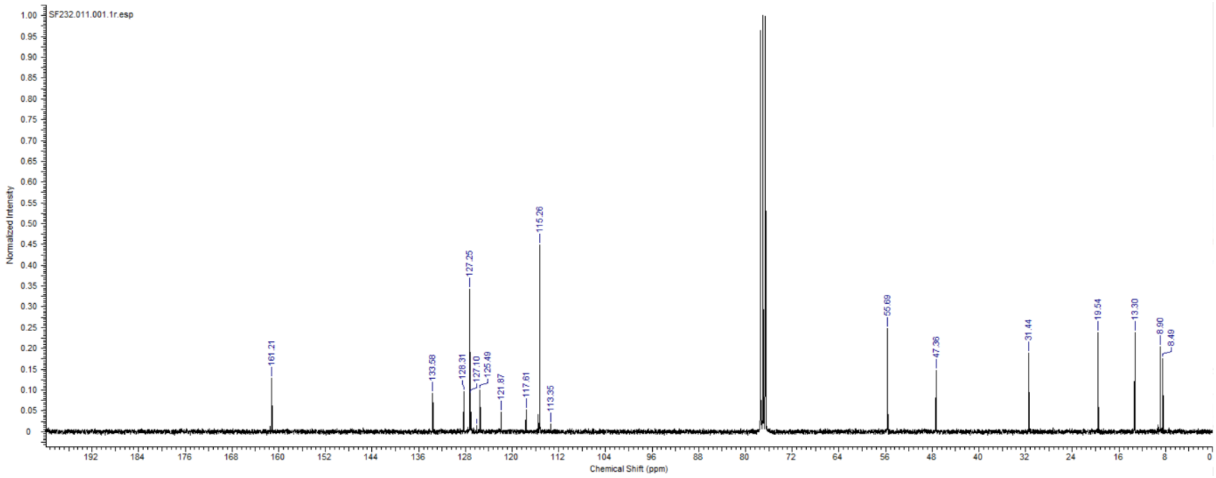

Figure S130. ^{13}C NMR of compound **43**.

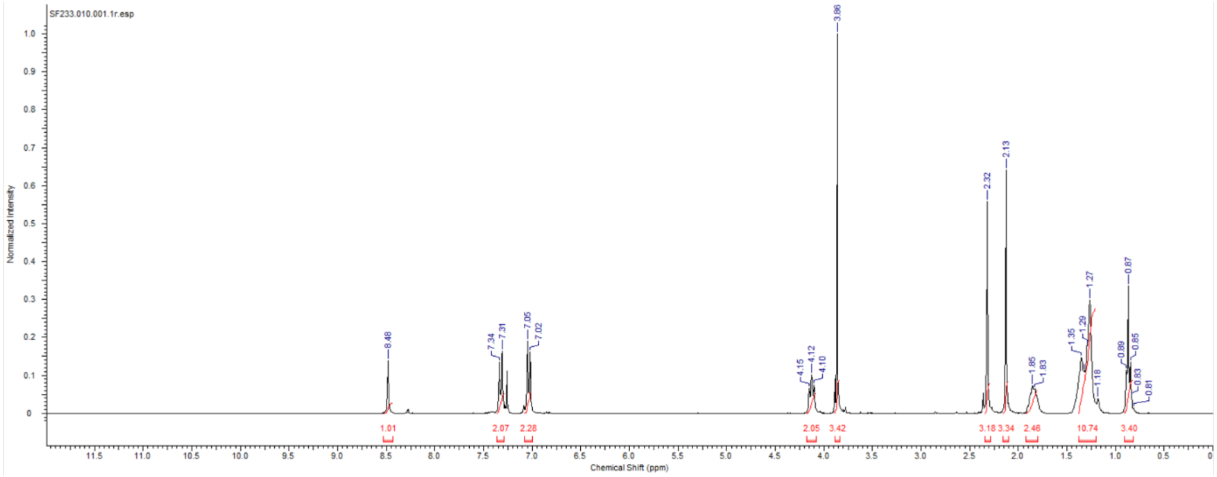

Figure S131. ^{1}H NMR of compound **44**.

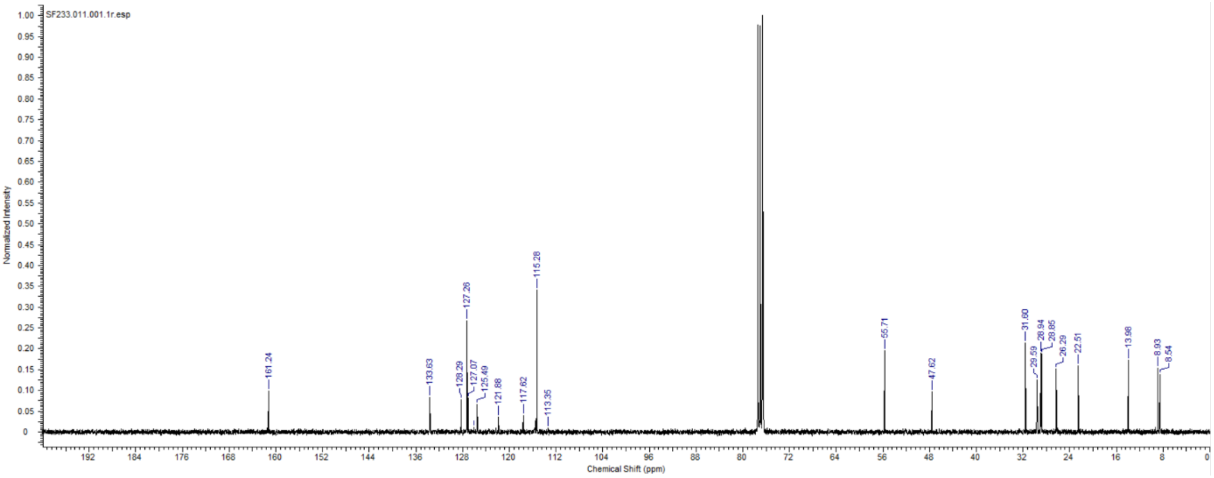

Figure S132. ^{13}C NMR of compound **44**.

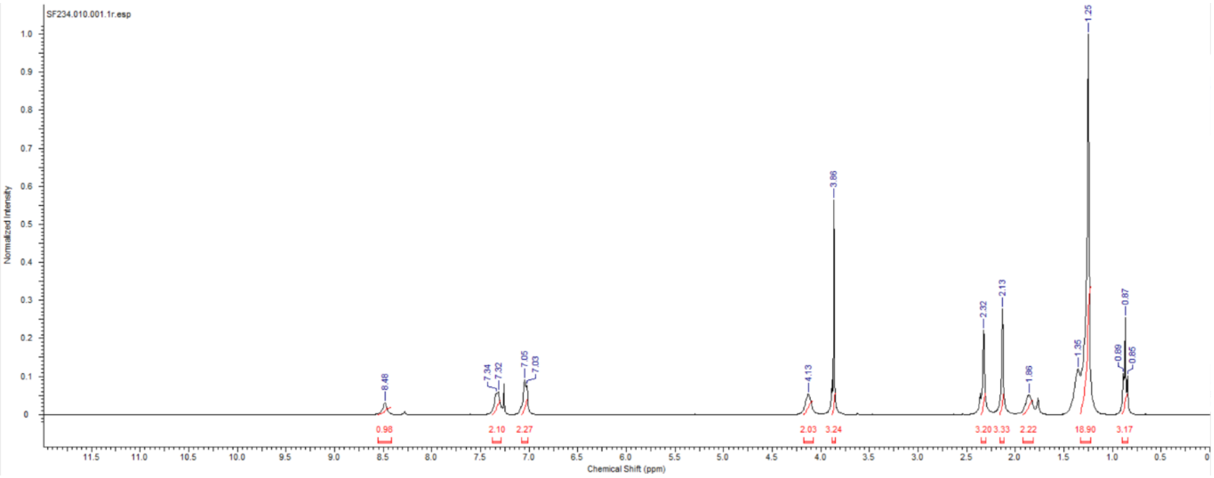

Figure S133. ^{1}H NMR of compound **45**.

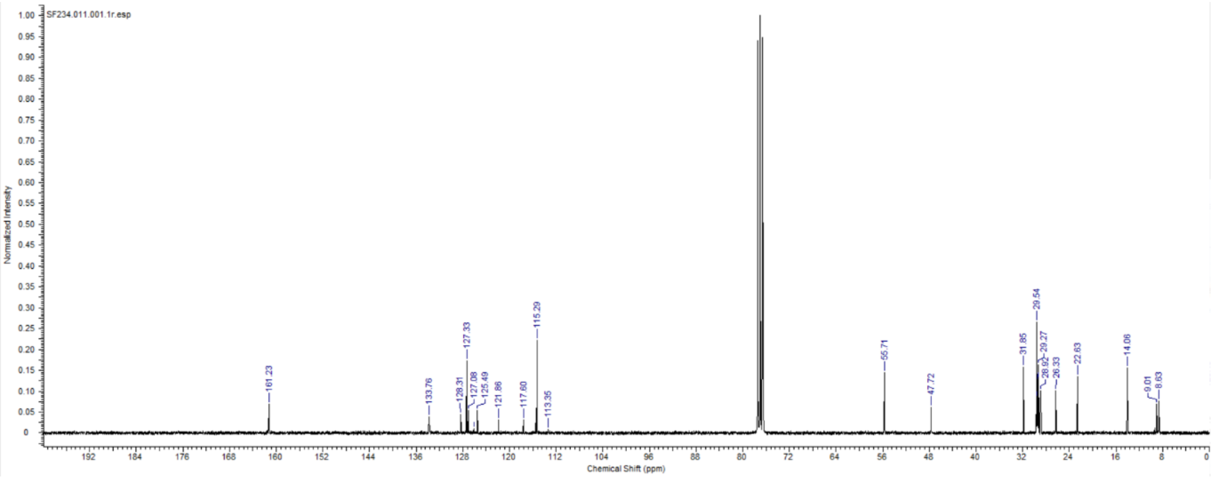

Figure S134. ^{13}C NMR of compound **45**.

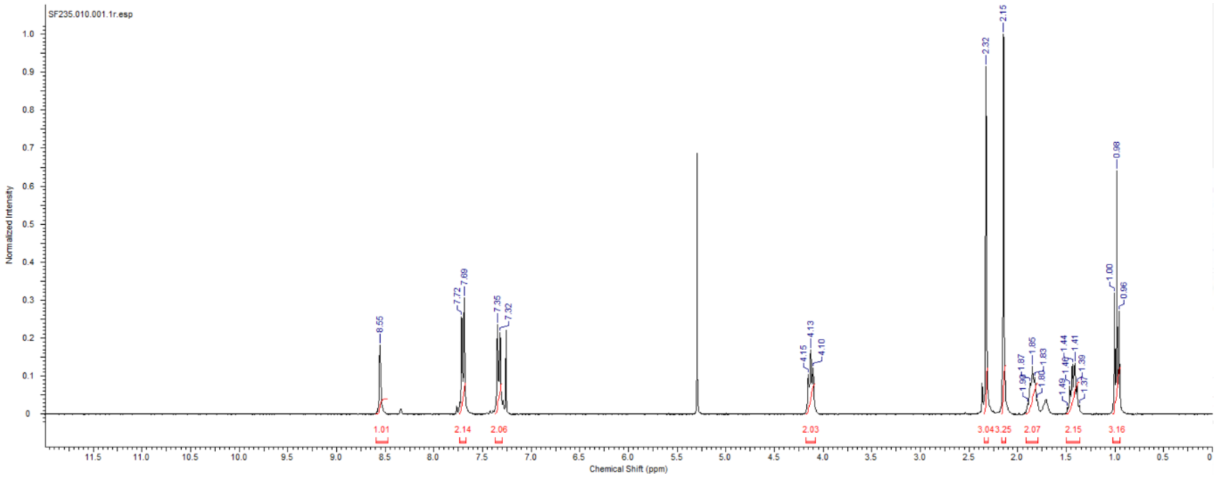

Figure S135. ^{1}H NMR of compound **46**.

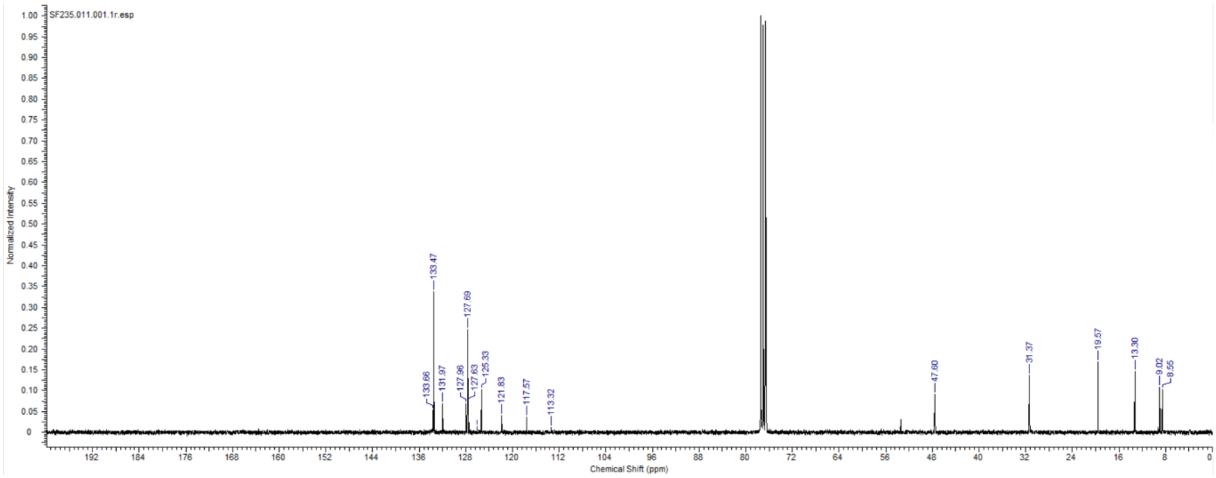

Figure S136. ^{13}C NMR of compound **46**.

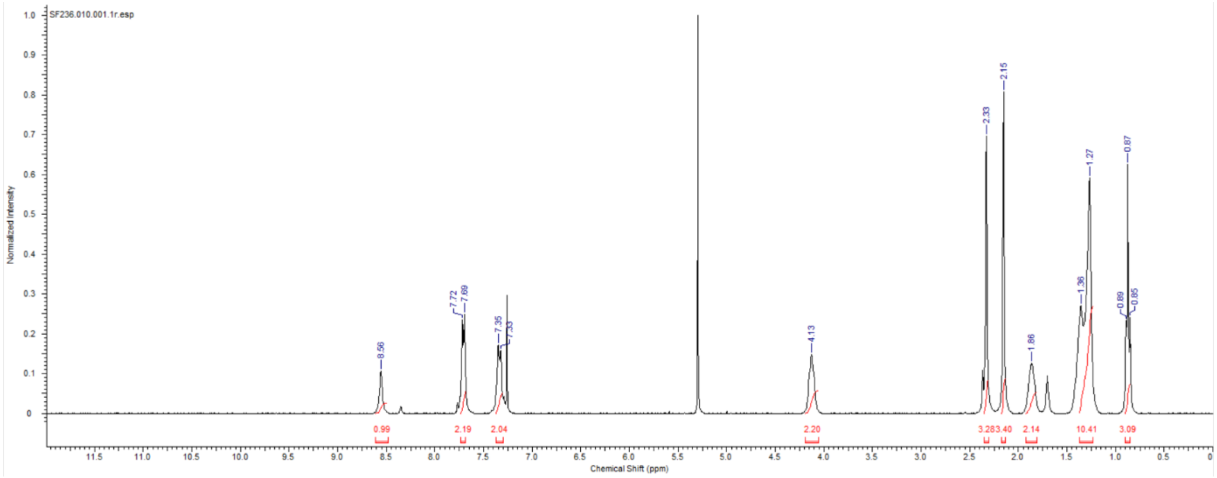

Figure S137. ^{1}H NMR of compound **47**.

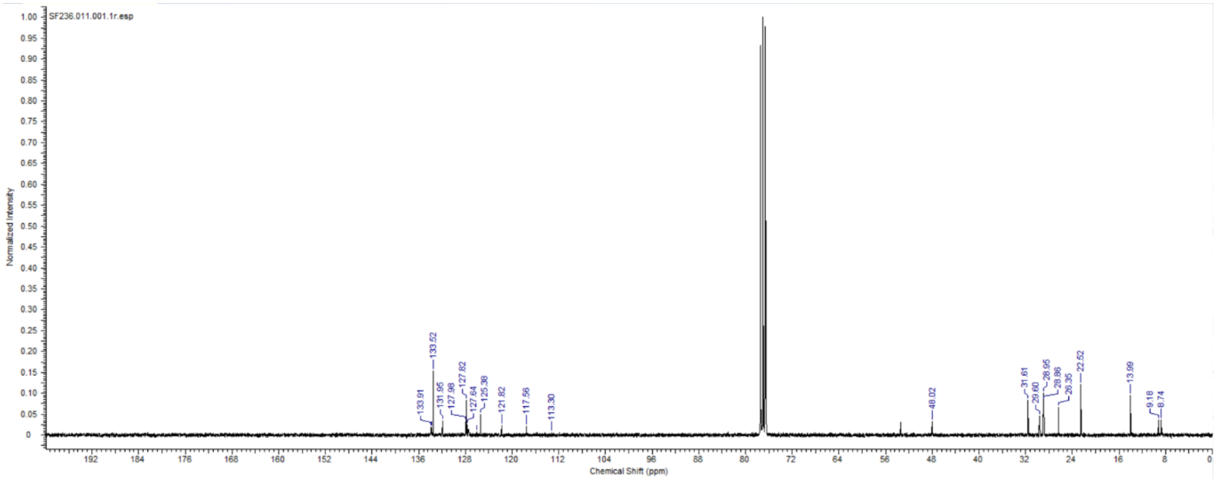

Figure S138. ^{13}C NMR of compound **47**.

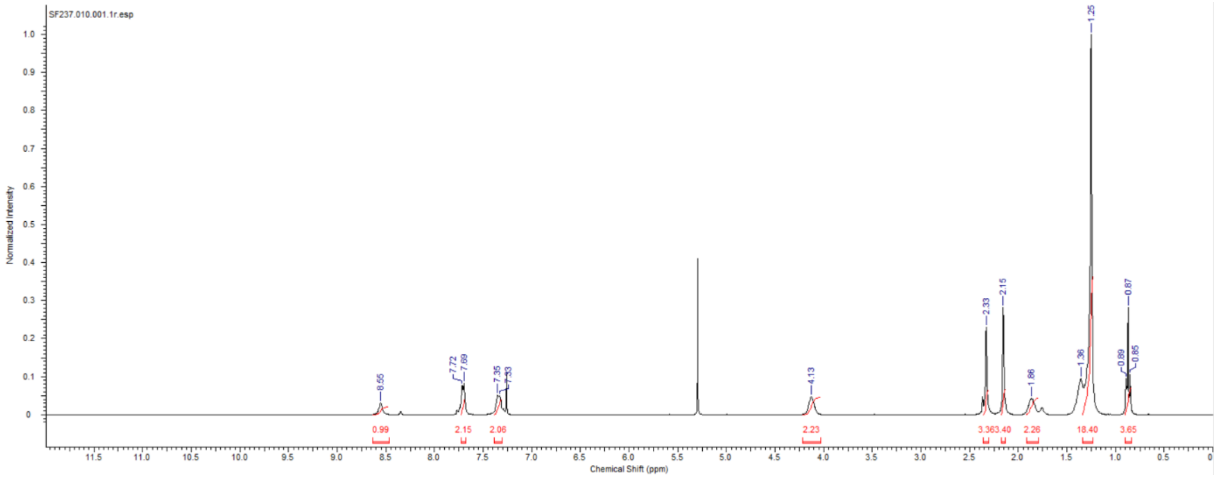

Figure S139. ^{1}H NMR of compound **48**.

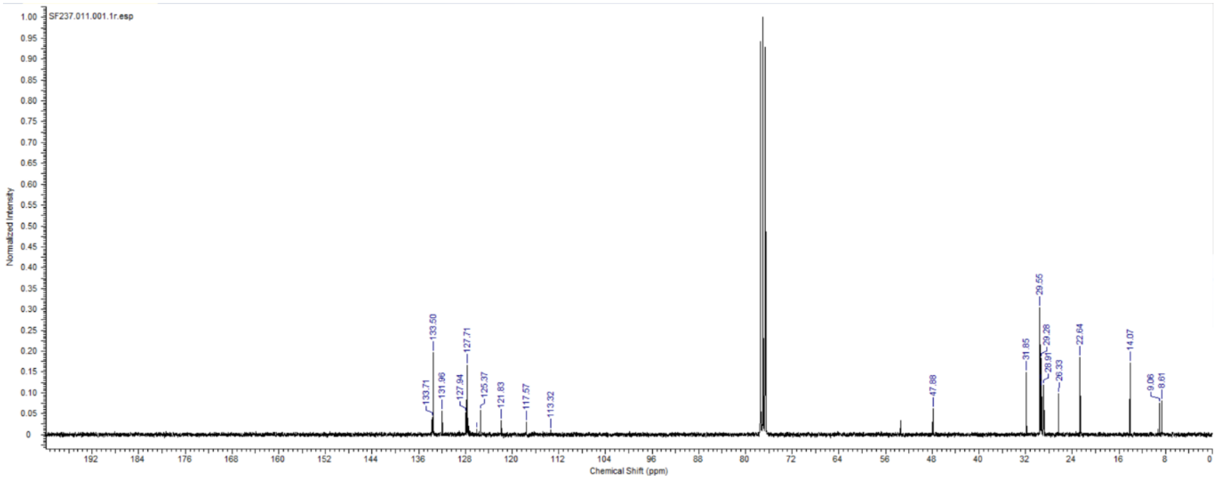

Figure S140. ^{13}C NMR of compound **48**.

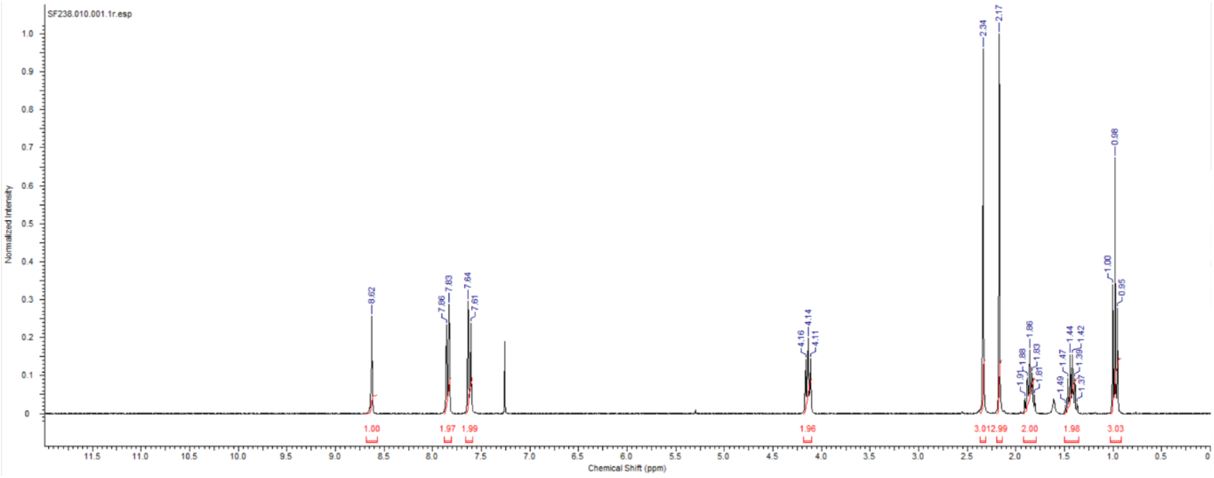

Figure S141. ^{1}H NMR of compound **49**.

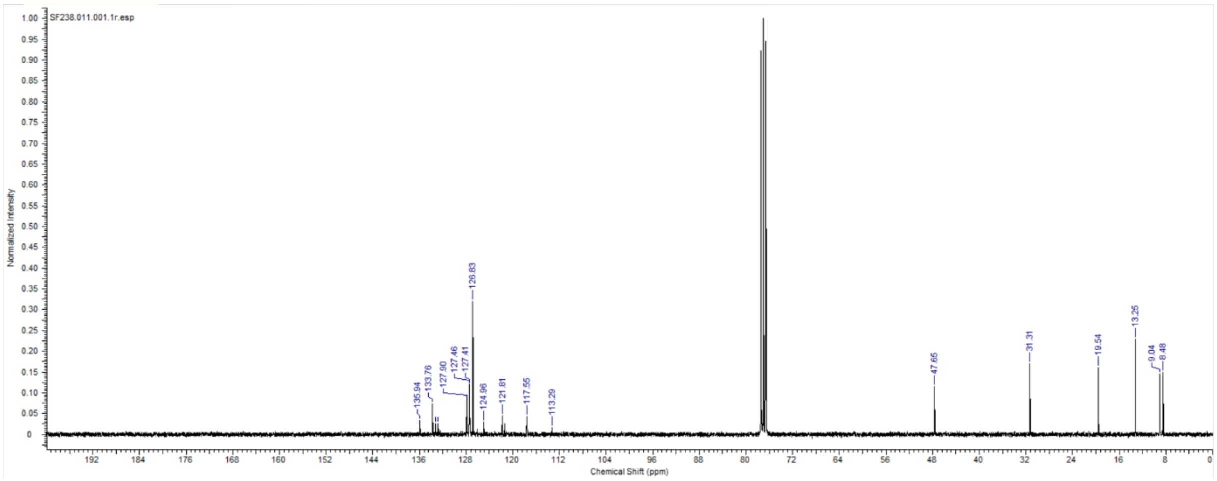

Figure S142. ^{13}C NMR of compound **49**.

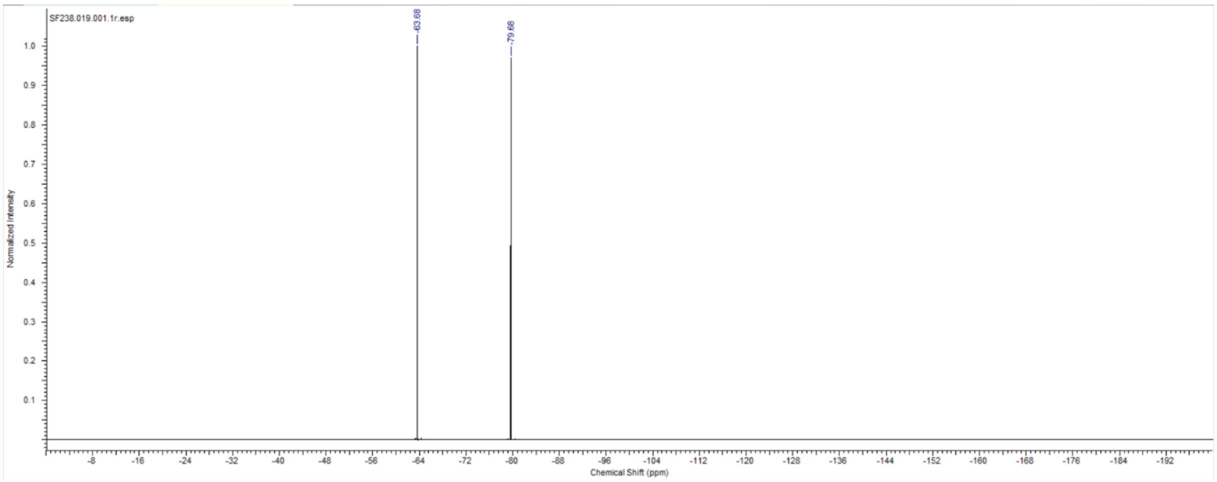

Figure S143. ^{19}F NMR of compound **49**.

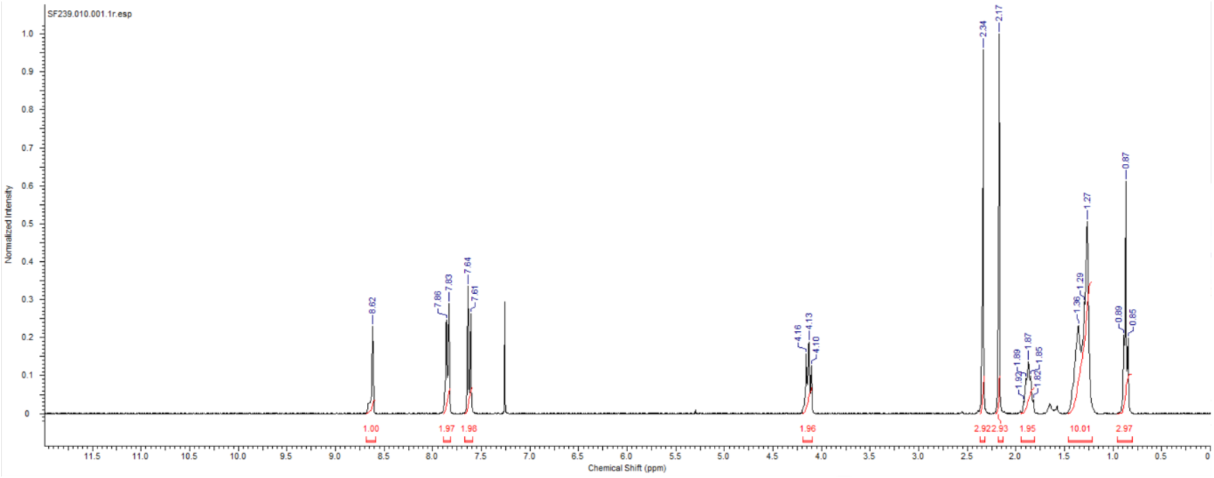

Figure S144. ^{1}H NMR of compound **50**.

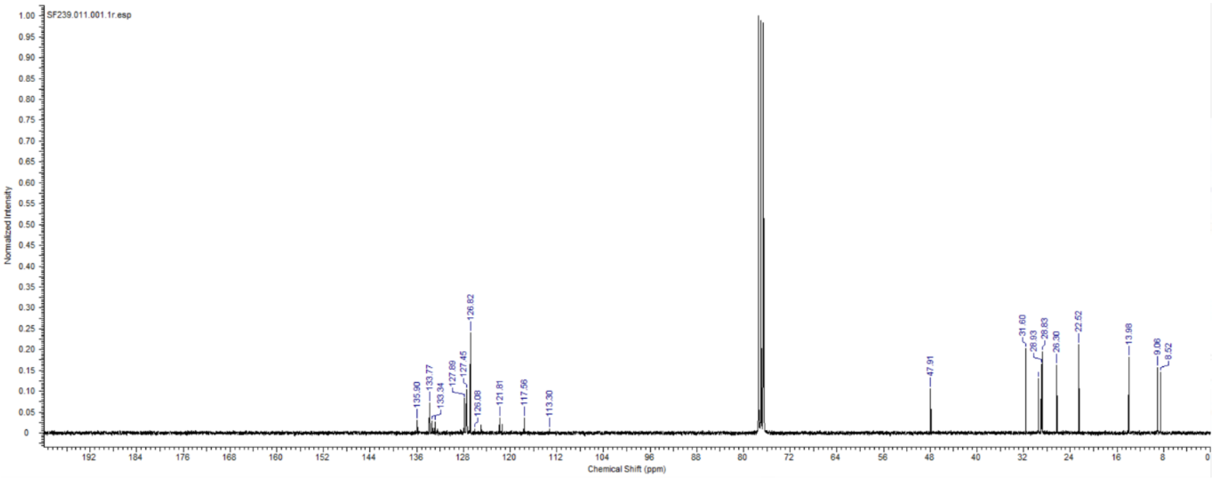

Figure S145. ^{13}C NMR of compound **50**.

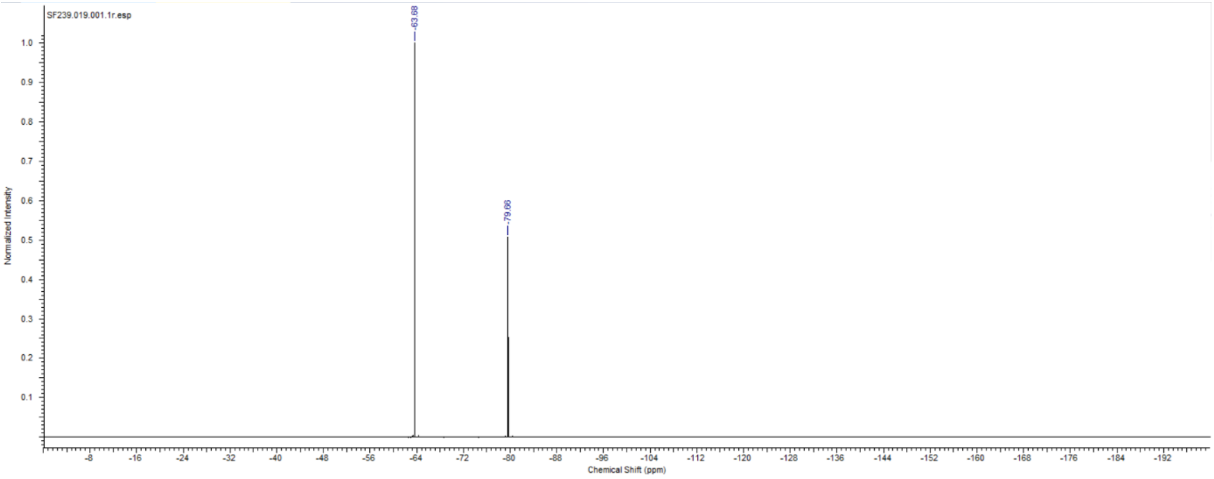

Figure S146. ^{19}F NMR of compound **50**.

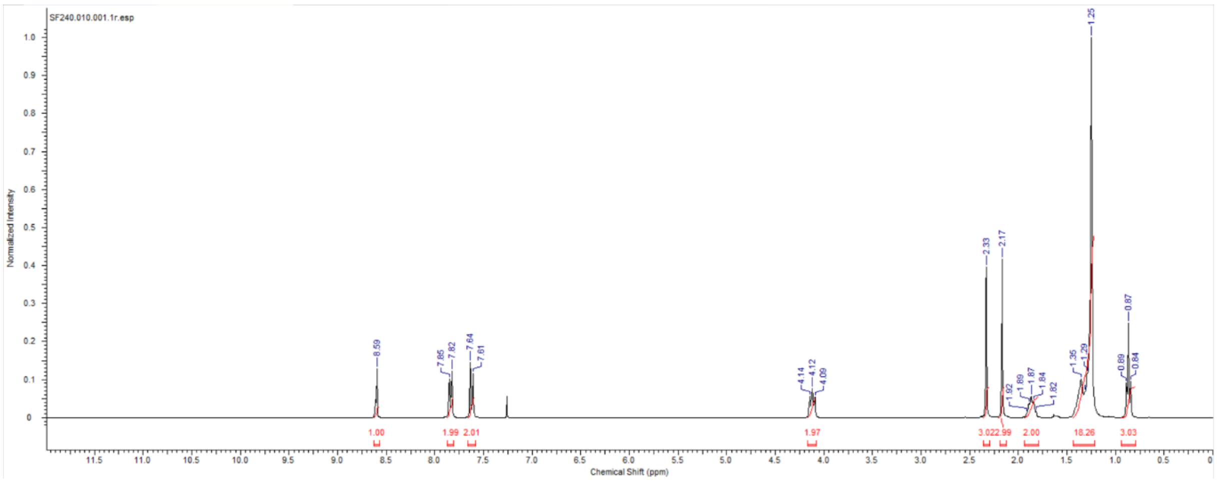

Figure S147. ^{1}H NMR of compound **51**.

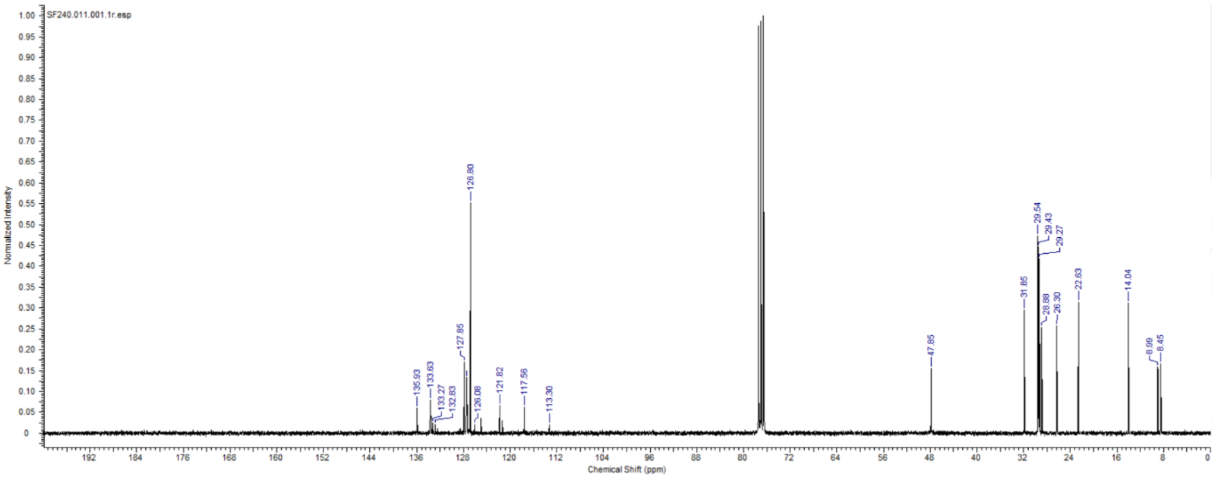

Figure S148. ^{13}C NMR of compound **51**.

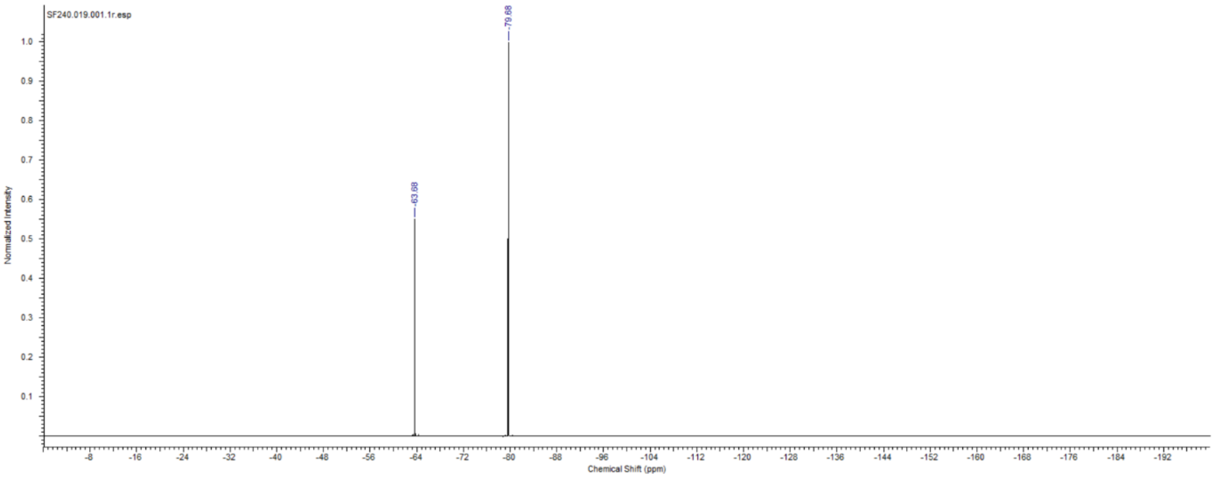

Figure S149. ^{19}F NMR of compound **51**.

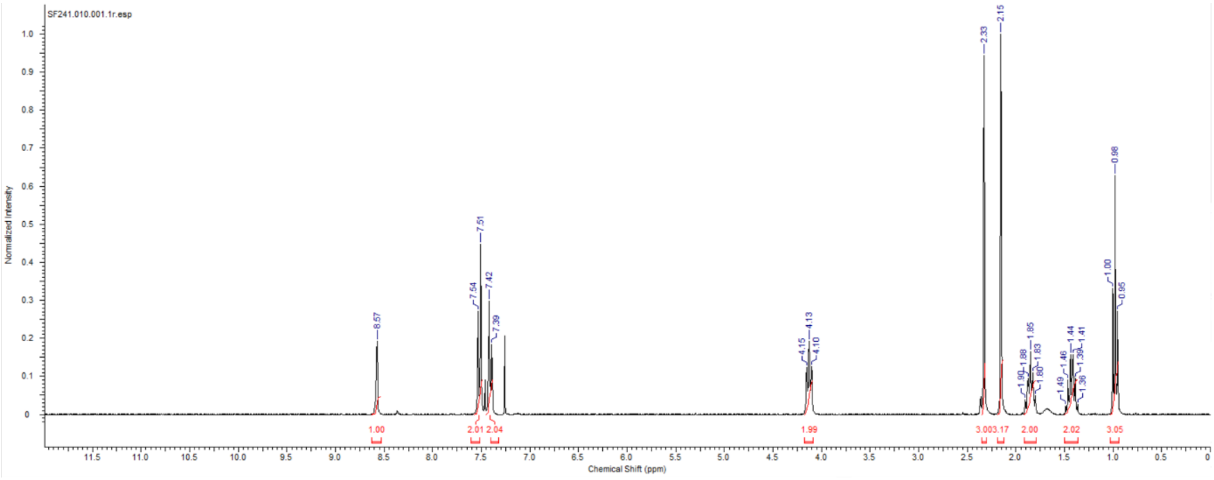

Figure S150. ^{1}H NMR of compound **52**.

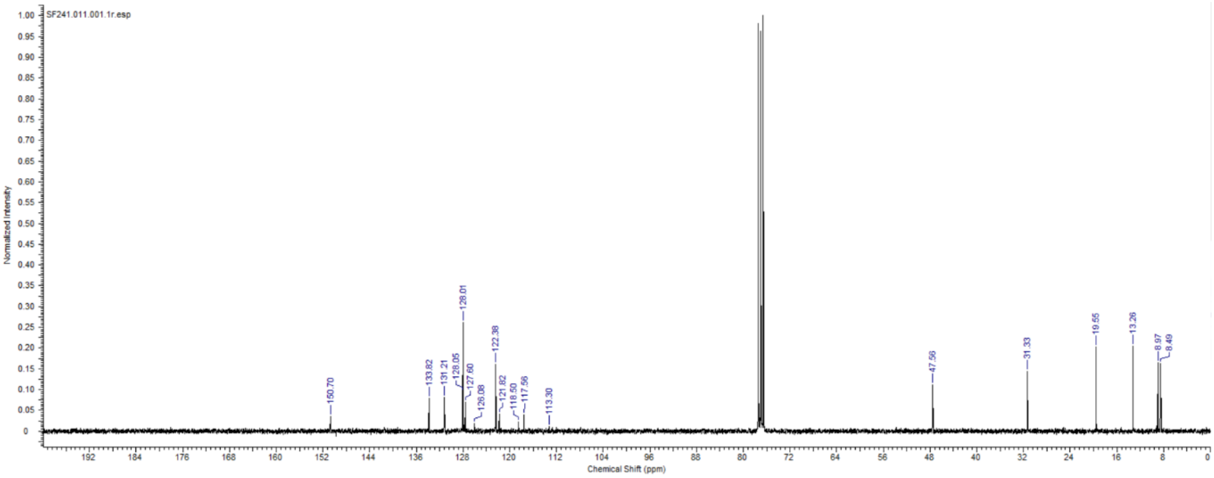

Figure S151. ^{13}C NMR of compound **52**.

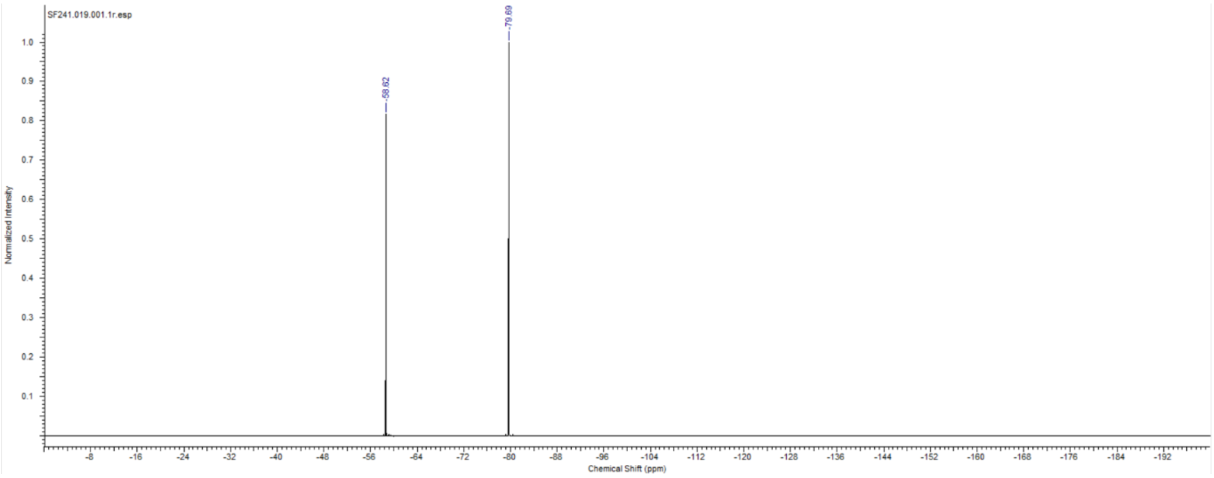

Figure S152. ^{19}F NMR of compound **52**.

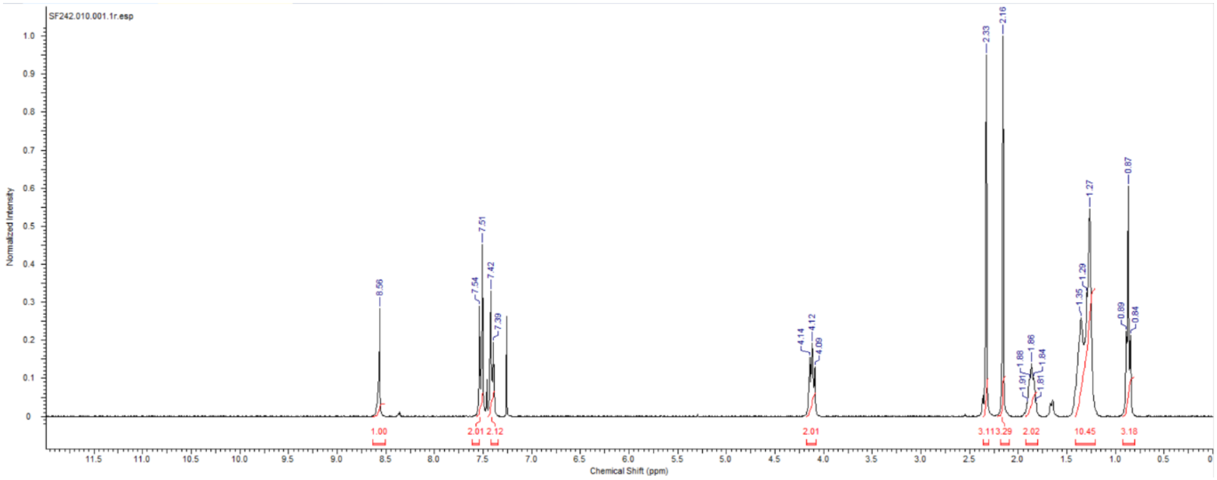

Figure S153. ^{1}H NMR of compound **53**.

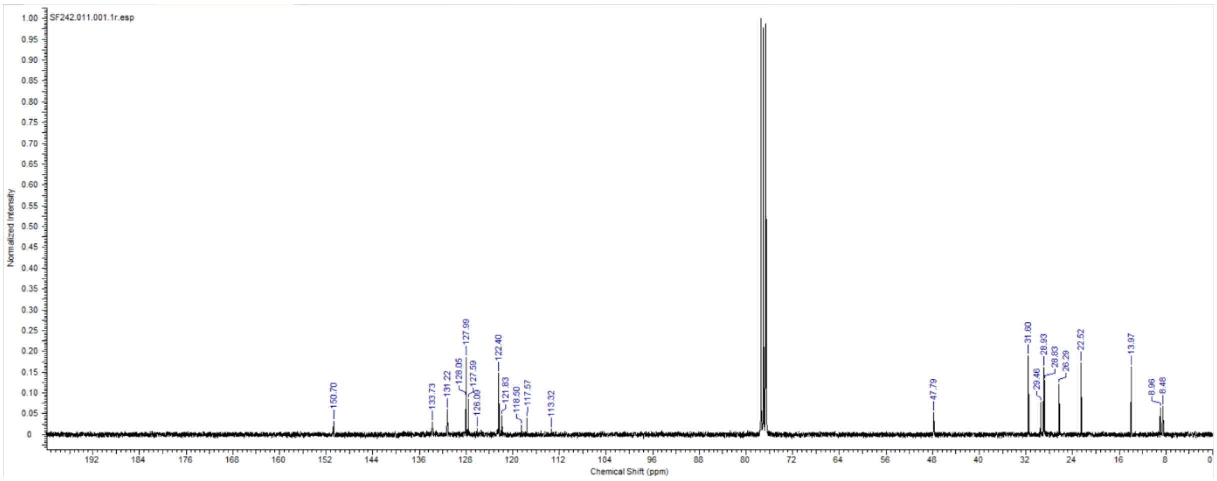

Figure S154. ^{13}C NMR of compound **53**.

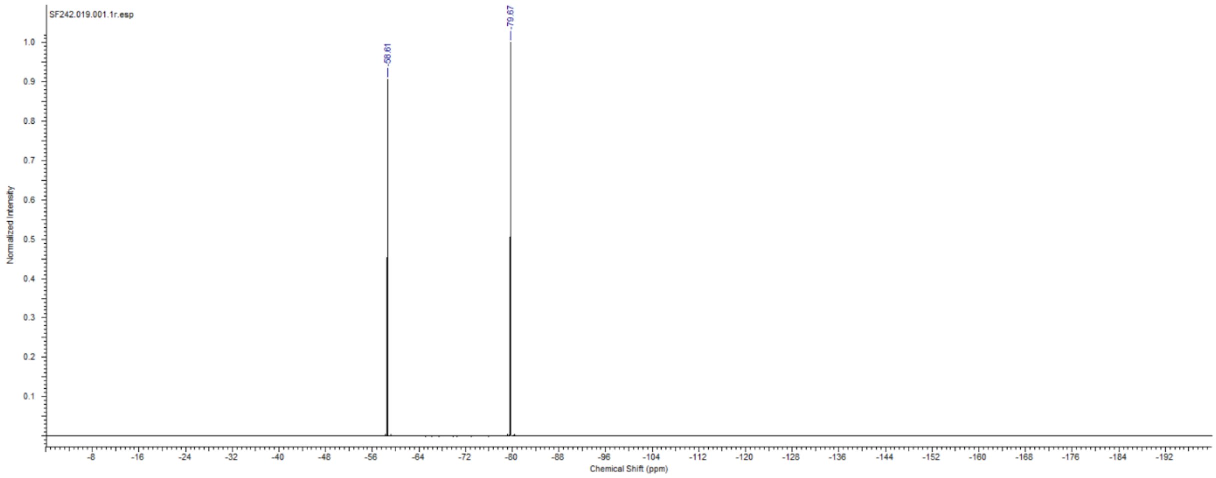

Figure S155. ^{19}F NMR of compound **53**.

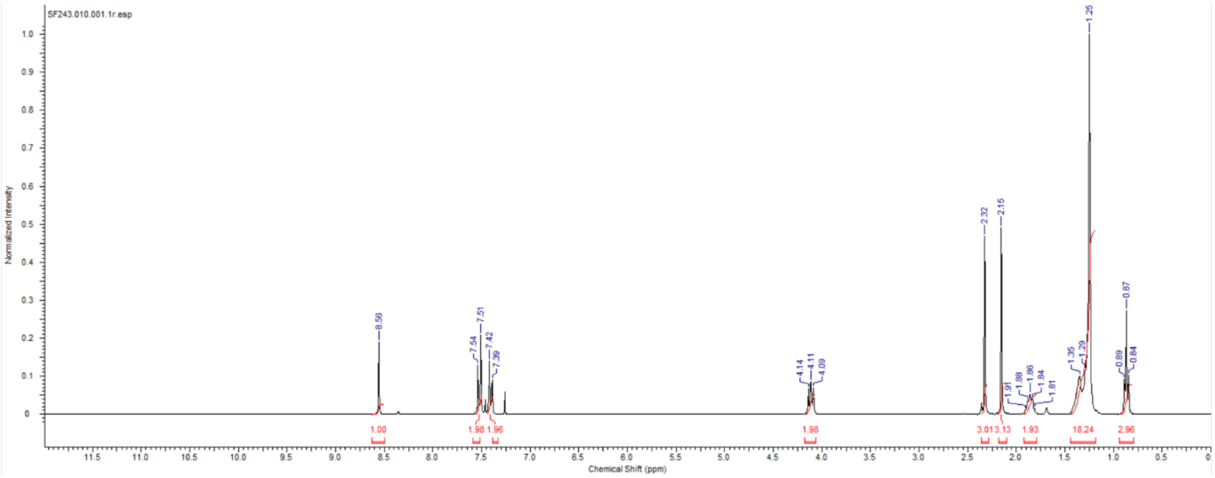

Figure S156. ^{1}H NMR of compound **54**.

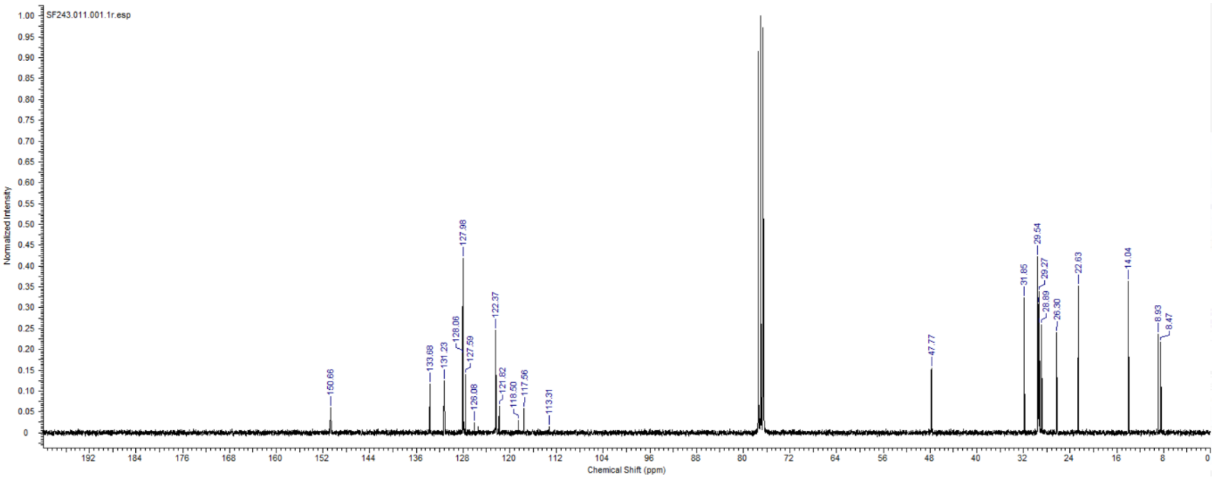

Figure S157. ^{13}C NMR of compound **54**.

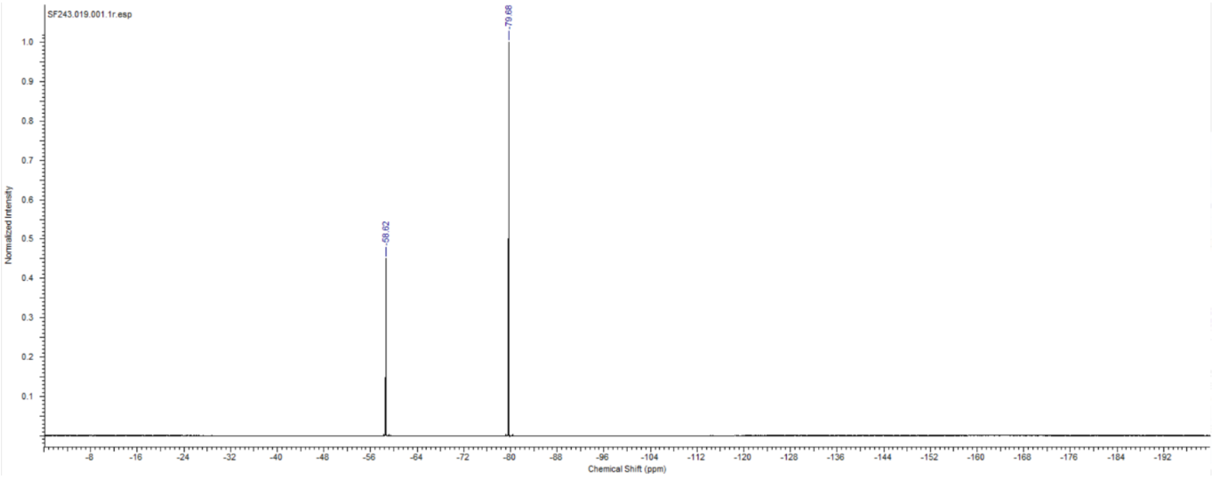

Figure S158. ^{19}F NMR of compound **54**.

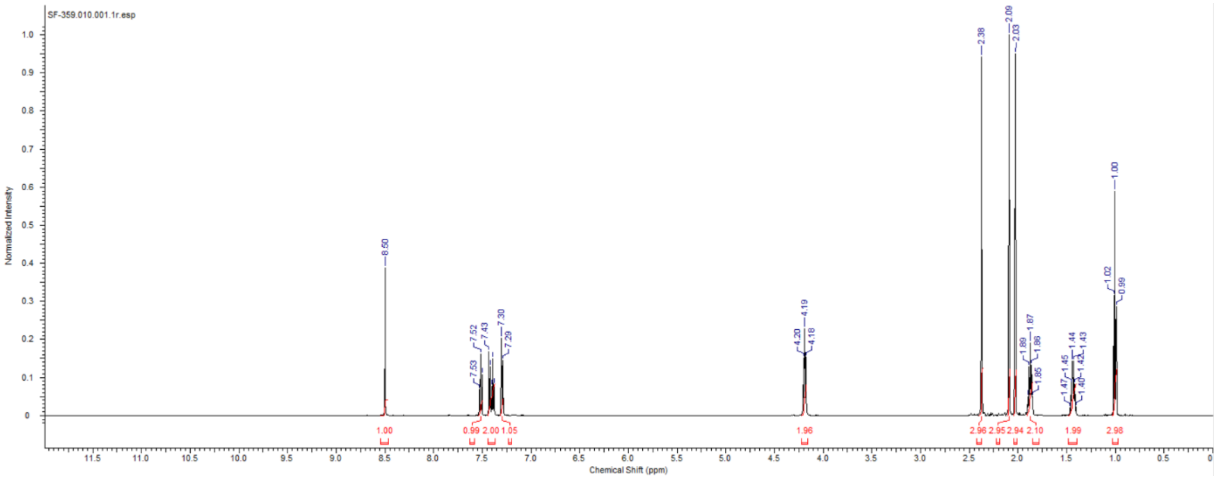

Figure S159. ^{1}H NMR of compound **55**.

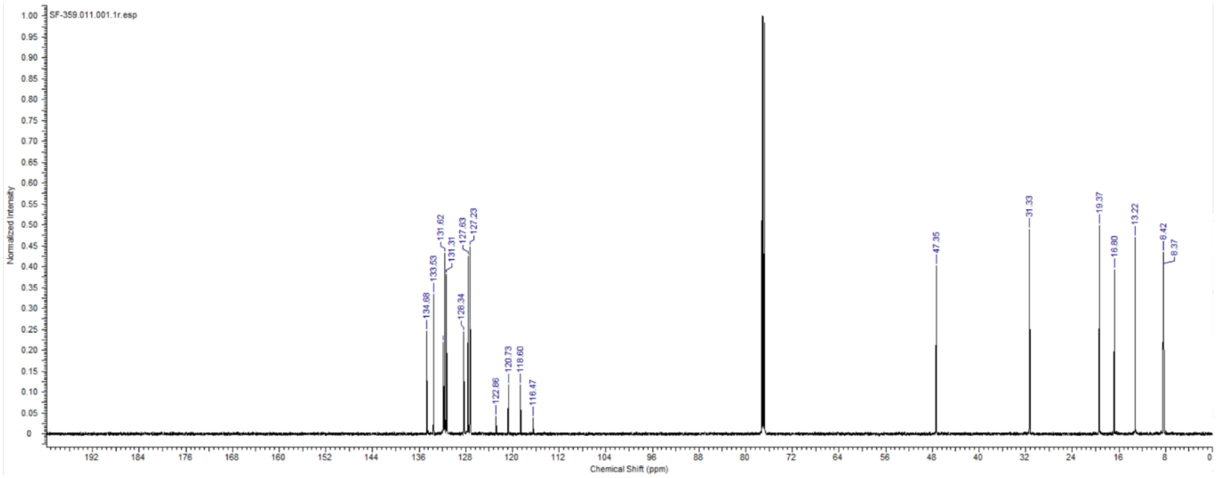

Figure S160. ^{13}C NMR of compound **55**.

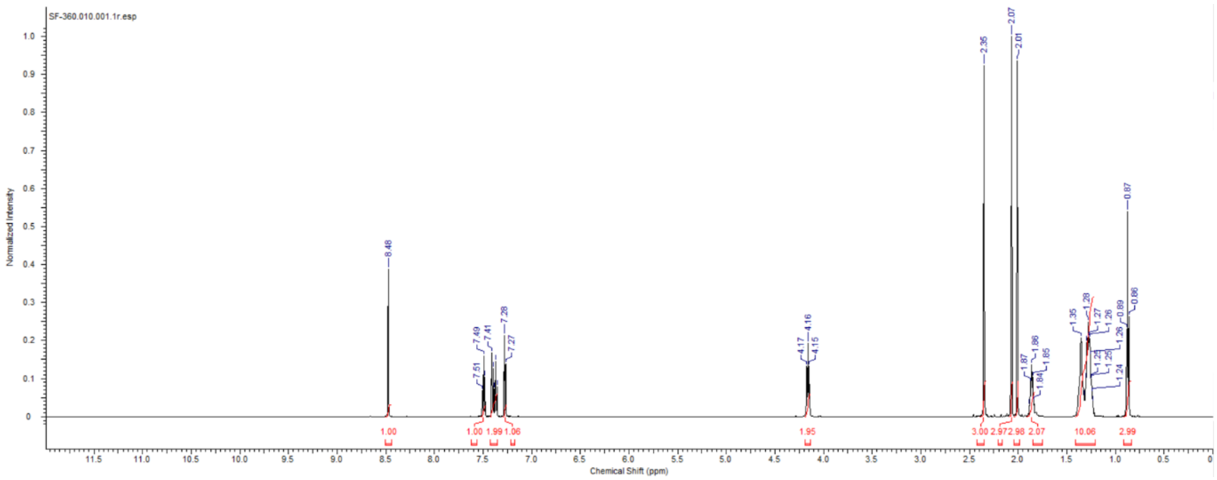

Figure S161. ^{1}H NMR of compound **56**.

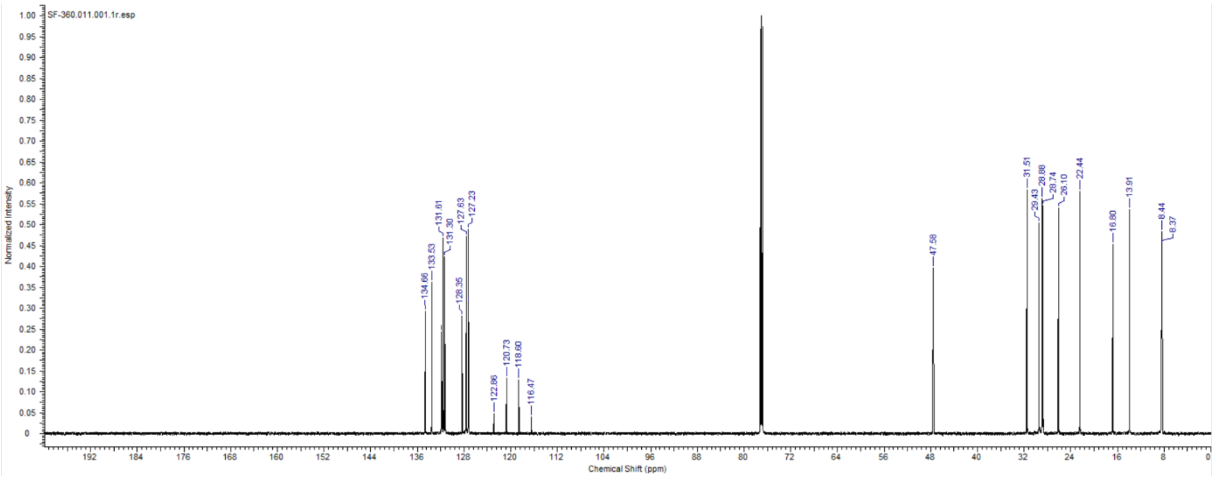

Figure S162. ^{13}C NMR of compound **56**.

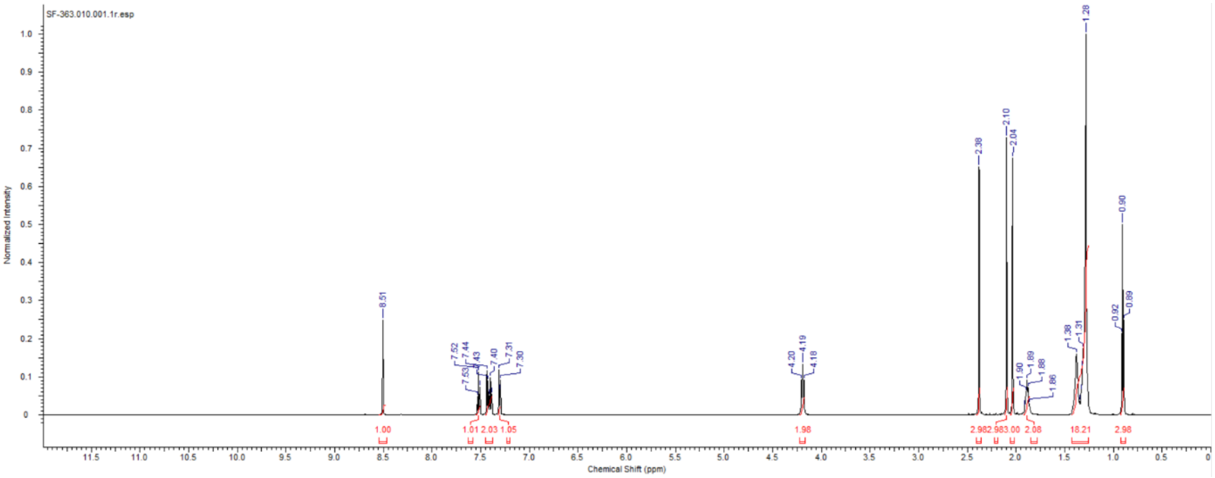

Figure S163. ^{1}H NMR of compound **57**.

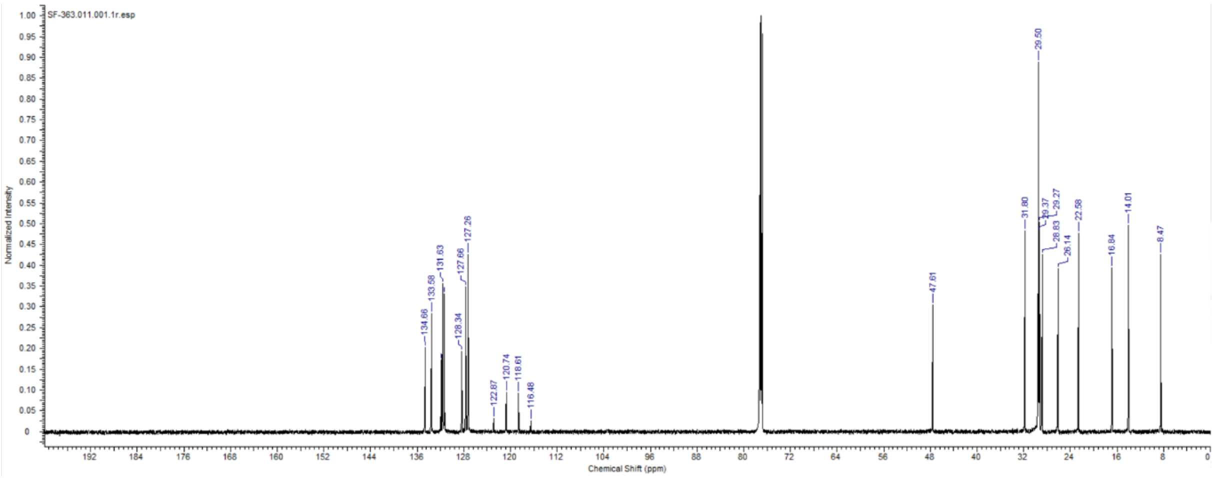

Figure S164. ^{13}C NMR of compound **57**.

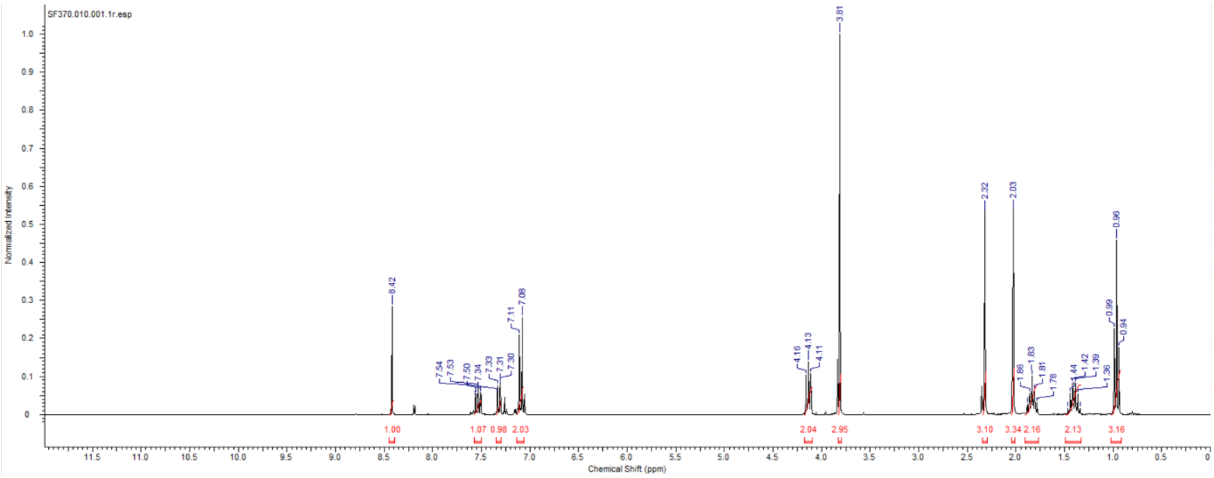

Figure S165. ^{1}H NMR of compound **58**.

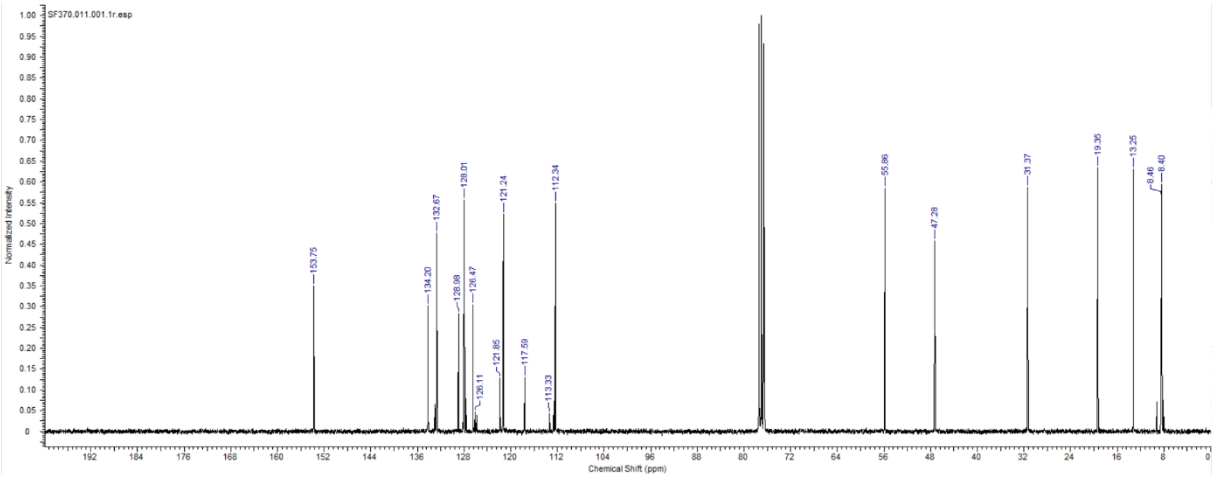

Figure S166. ^{13}C NMR of compound **58**.

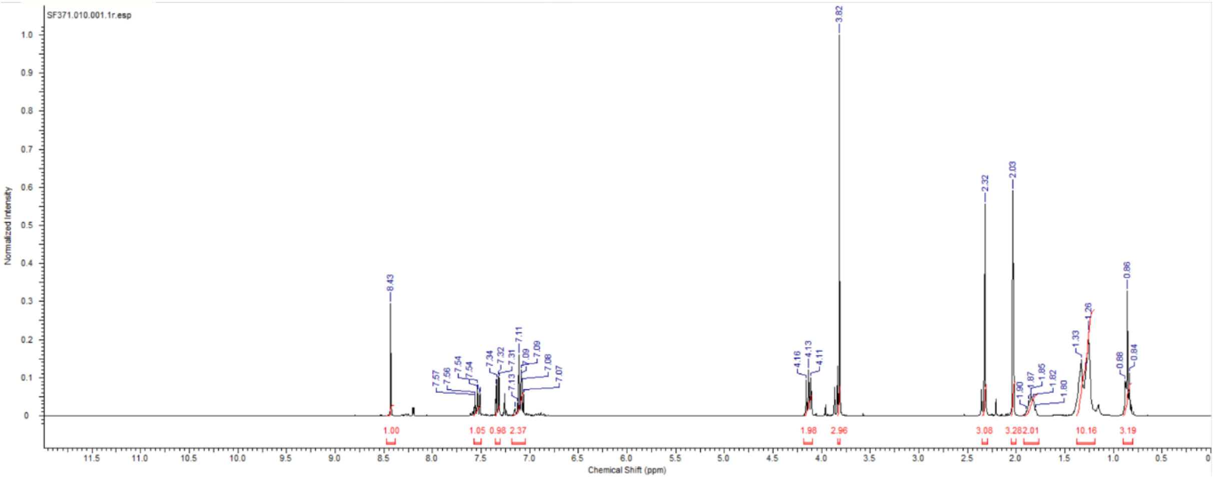

Figure S167. ^{1}H NMR of compound **59**.

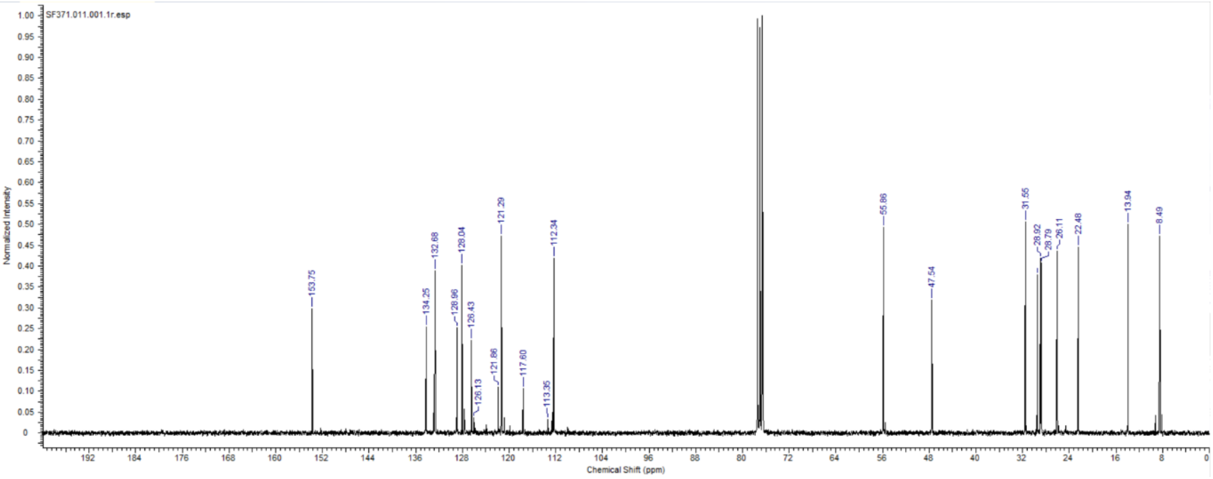

Figure S168. ^{13}C NMR of compound **59**.

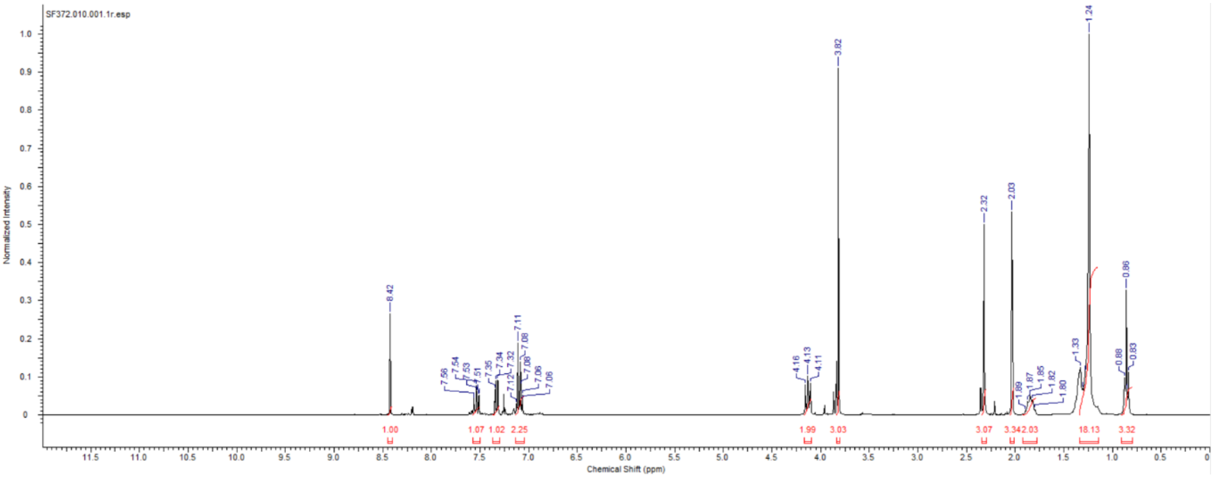

Figure S169. ^{1}H NMR of compound **60**.

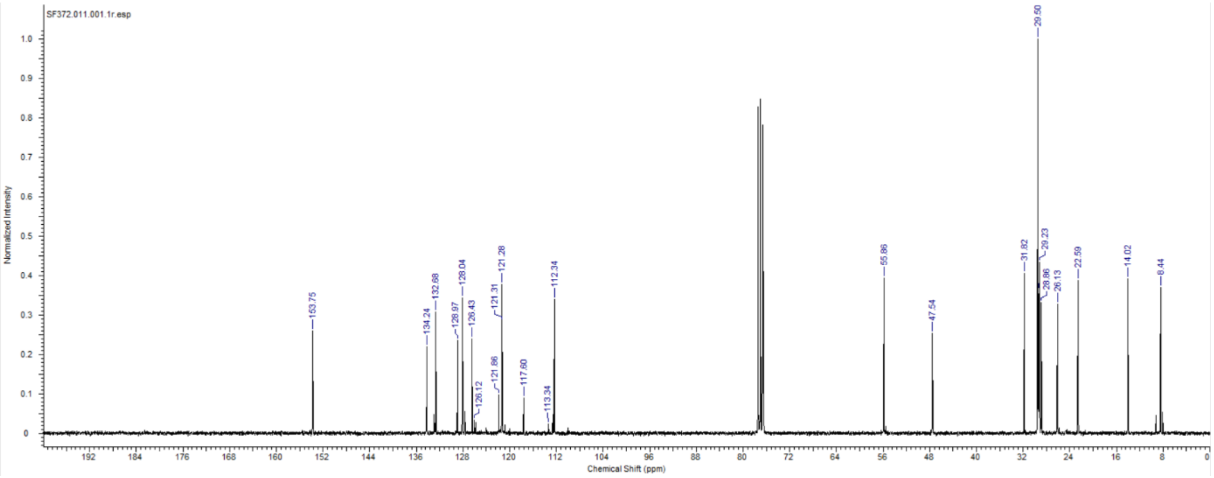

Figure S170. ^{13}C NMR of compound **60**.

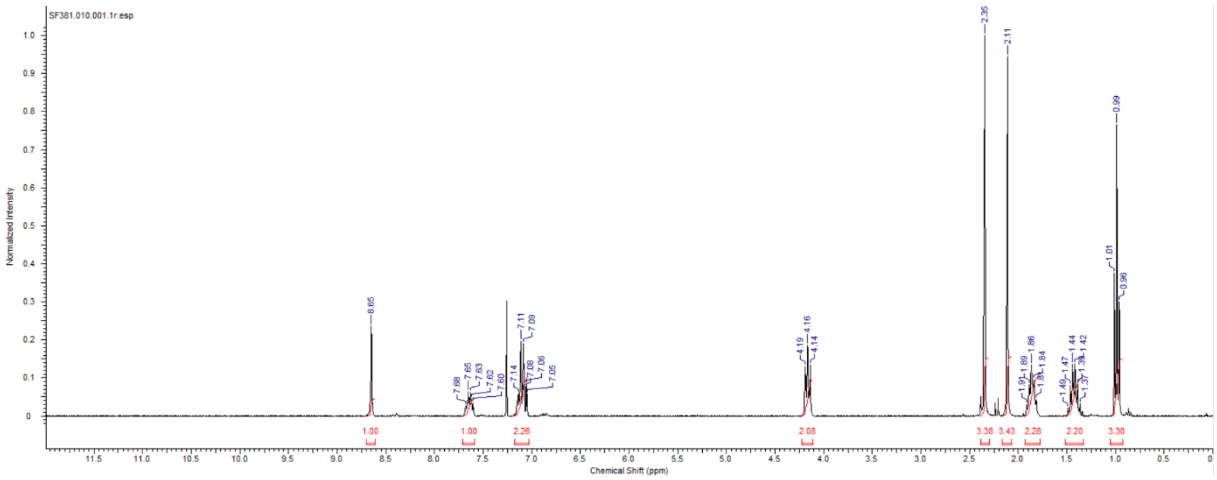

Figure S171. ^{1}H NMR of compound **61.**

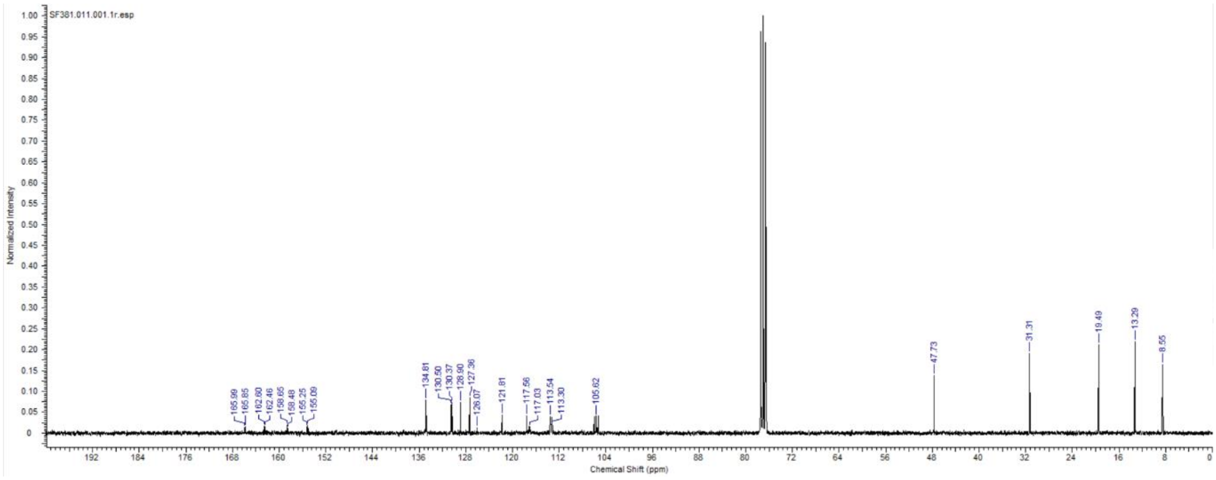

Figure S172. ^{13}C NMR of compound **61**.

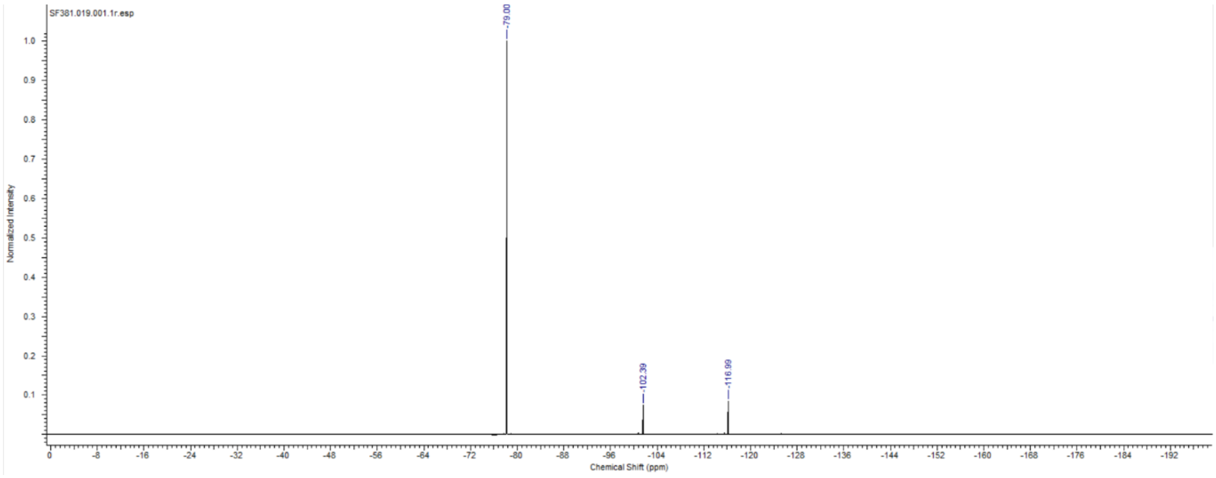

Figure S173. ^{19}F NMR of compound **61**.

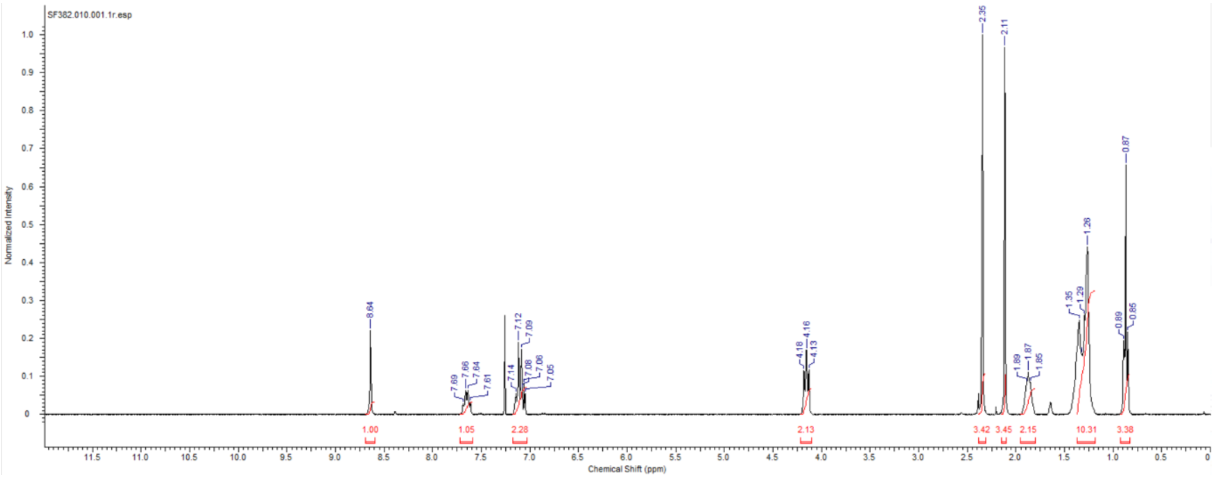

Figure S174. ^{1}H NMR of compound **62.**

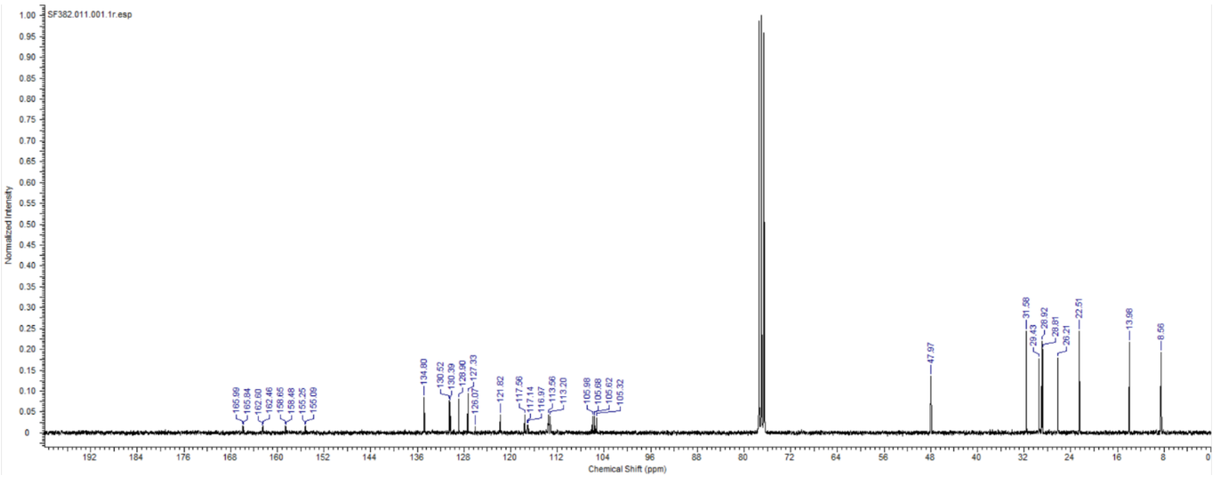

Figure S175. ^{13}C NMR of compound **62**.

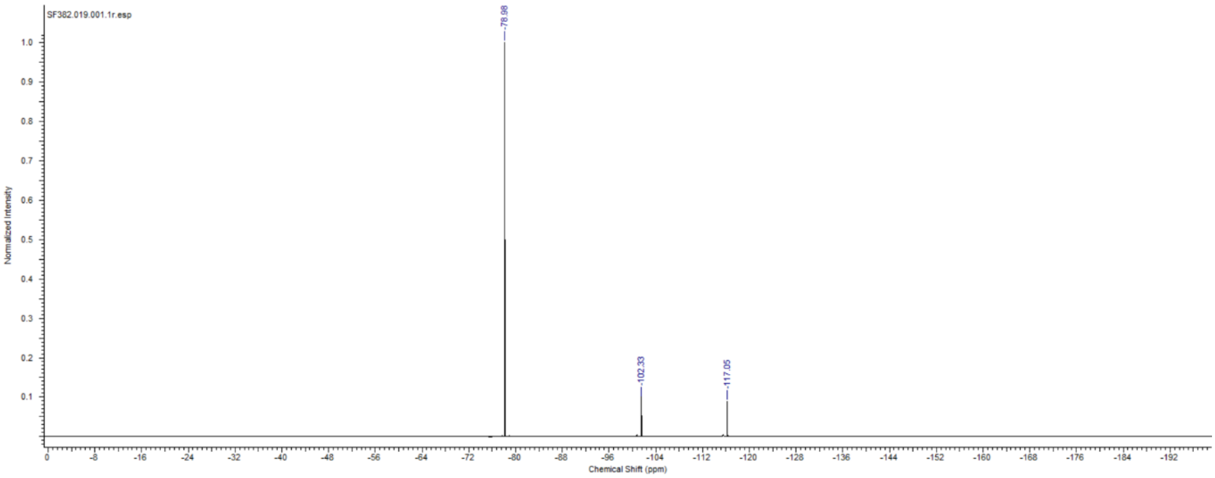

Figure S176. ^{19}F NMR of compound **62**.

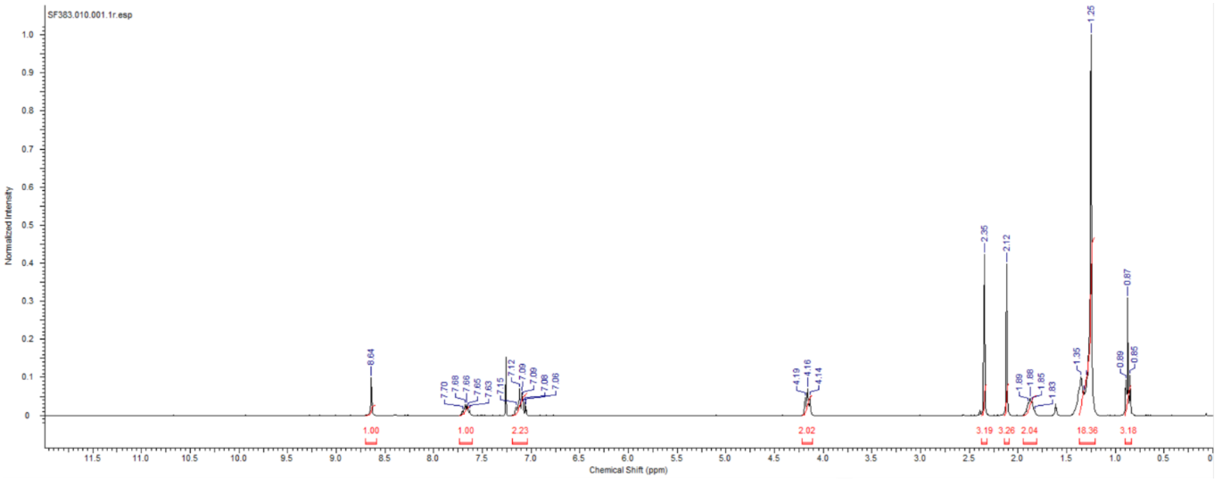

Figure S177. ^{1}H NMR of compound **63.**

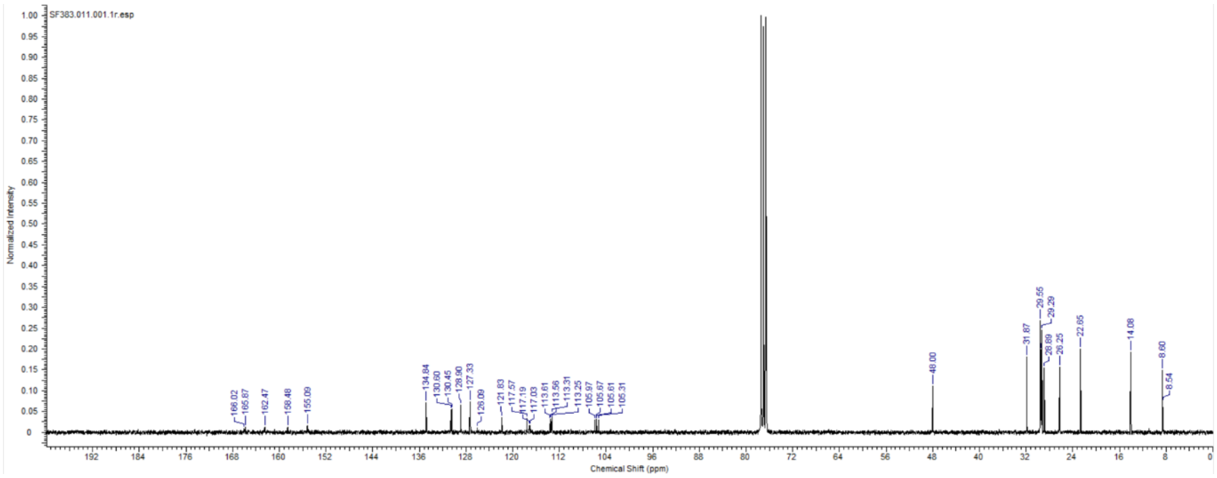

Figure S178. ^{13}C NMR of compound **63**.

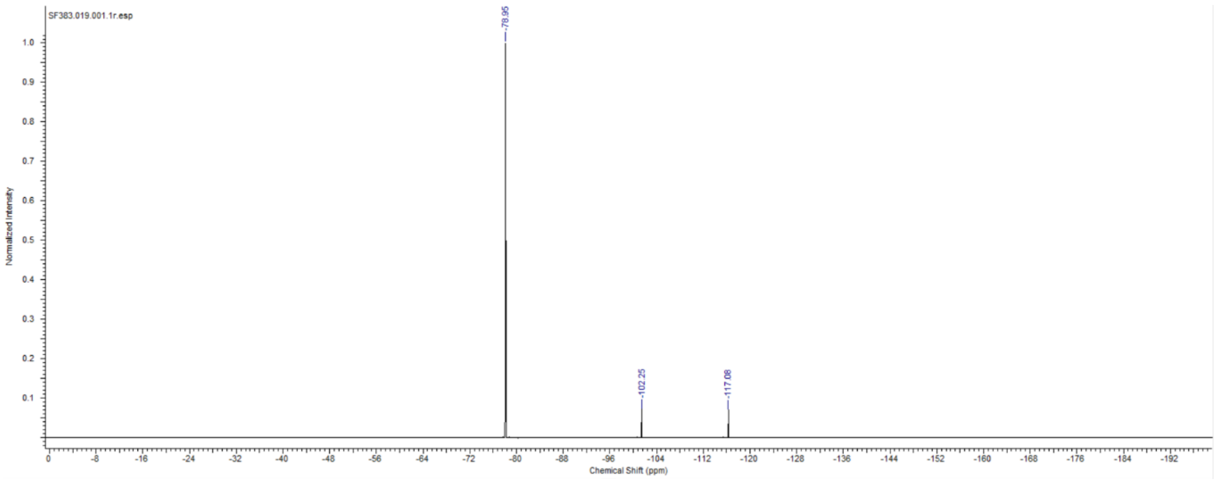

Figure S179. ^{19}F NMR of compound **63**.

S8 Cartesian coordinates of optimized structures

Cation $[PhImC_4H_9]^+$

C	-4.83860800	-0.92889800	0.01867700
C	-3.78475000	-1.80958300	-0.21698200
C	-2.47526400	-1.33864800	-0.25606300
C	-2.24486400	0.01688000	-0.04160900
C	-3.28412500	0.91058400	0.19838300
C	-4.58907000	0.42738500	0.22201900
H	-5.85583700	-1.29926200	0.04162700
H	-3.98008800	-2.86109700	-0.38521800
H	-1.65255100	-2.00881300	-0.47466400
H	-3.07734700	1.95786700	0.38199500
H	-5.40814500	1.10990700	0.41019300
N	-0.89170300	0.51231700	-0.07150800
C	-0.43145300	1.58130200	-0.82332900
C	0.13496600	0.01337700	0.63291100
C	0.89694300	1.71130300	-0.55685300
H	-1.08180300	2.13059100	-1.48100800
H	0.07292300	-0.80790800	1.32599900
H	1.62517900	2.40736900	-0.93538500
C	2.58674000	0.46404600	0.88079000
C	3.48044800	-0.22374300	-0.14925900
H	2.46726500	-0.14930100	1.77496300
H	2.99974600	1.42500100	1.19183700
C	4.88174100	-0.49117100	0.40989800
H	3.01423900	-1.16578800	-0.45766600
H	3.55341200	0.40183700	-1.04524700
C	5.79092200	-1.17927300	-0.60951900
H	5.33257500	0.45602900	0.72617200
H	4.80094500	-1.11131000	1.30967200
H	6.78146400	-1.35937300	-0.18814700
H	5.37998300	-2.14429500	-0.91858500

H	5.91685300	-0.56519600	-1.50542800
N	1.23019200	0.72516500	0.35357800

Cation **37**

C	-4.31091300	-0.97087600	-1.06662800
C	-3.00641700	-0.49835000	-1.19000800
C	-2.24742800	-0.31590000	-0.03851700
C	-2.75825300	-0.58523000	1.22726900
C	-4.06527000	-1.05091800	1.33787800
C	-4.83866400	-1.24350000	0.19378300
H	-4.91341400	-1.11946100	-1.95389000
H	-2.58713400	-0.26593000	-2.16153100
H	-2.14155500	-0.44241600	2.10616000
H	-4.47590300	-1.26864900	2.31579500
H	-5.85445700	-1.60754400	0.28537400
N	-0.89644700	0.17670000	-0.16033700
C	0.13477700	-0.53305100	-0.63287200
C	-0.45975800	1.46506900	0.15645100
H	0.08925900	-1.56157500	-0.94760400
C	0.87865200	1.50443200	-0.13950600
C	-1.38344700	2.50797800	0.67793700
H	-2.27465400	2.58736400	0.05144900
H	-1.71196700	2.28208000	1.69514000
H	-0.88694600	3.47710500	0.69007300
C	1.86266900	2.61594900	-0.03122100
H	2.27804900	2.87931800	-1.00769000
H	1.37779600	3.50307500	0.37309800
H	2.69202400	2.35879300	0.63180300
C	2.57244300	-0.20554100	-1.00784600
C	3.37633700	-0.69021600	0.19800200
H	3.06630500	0.62985700	-1.50490900
H	2.45459000	-1.00004400	-1.74634200
C	4.78892800	-1.12699300	-0.20162800

H	3.43311100	0.10695800	0.94613400
H	2.84570900	-1.52504000	0.66885800
C	5.60343700	-1.62554900	0.99300500
H	4.72398600	-1.91651400	-0.95876000
H	5.30704700	-0.28609200	-0.67621500
H	6.60464900	-1.93003700	0.68285700
H	5.71276400	-0.84479300	1.75087300
H	5.12487200	-2.48751900	1.46598800
N	1.21834800	0.24433700	-0.63441300

Cation **40**

C	4.54467400	-0.83461400	0.10087000
C	3.74374700	-0.74494200	1.24669800
C	2.40743000	-0.37228400	1.16421500
C	1.86481300	-0.08081000	-0.08303900
C	2.63414400	-0.15276600	-1.23872400
C	3.96886600	-0.53305100	-1.13804500
H	4.17091800	-0.97373200	2.21612400
H	1.79272500	-0.31409900	2.05439200
H	2.19985100	0.09599500	-2.19976300
H	4.57261100	-0.58871600	-2.03619100
N	0.48286400	0.32270600	-0.17734600
C	-0.03488200	1.56943800	0.18050500
C	-0.50425400	-0.44000900	-0.66036200
C	-1.37617800	1.52863000	-0.10161200
H	-0.39388400	-1.45361900	-1.00577400
C	-2.96233700	-0.26453600	-1.00389400
C	-3.71996300	-0.83423000	0.19475800
H	-2.80044600	-1.02916500	-1.76525500
H	-3.51564800	0.54946300	-1.47312200
C	-5.10495700	-1.35224600	-0.20426400
H	-3.13047300	-1.64473400	0.63722900
H	-3.82097200	-0.06344300	0.96555300

C	-5.87176400	-1.93668900	0.98299900
H	-5.68258100	-0.53461000	-0.64994500
H	-4.99648000	-2.11433200	-0.98420000
H	-6.85423900	-2.29779600	0.67362100
H	-5.33232100	-2.77802900	1.42672600
H	-6.02360800	-1.18628900	1.76386600
N	-1.63727700	0.26329400	-0.62987900
C	0.82445400	2.65472600	0.72465200
H	1.18818300	2.41452700	1.72647900
H	1.69569700	2.81787800	0.08636900
H	0.26285700	3.58596300	0.78208100
C	-2.43048300	2.56866200	0.04800400
H	-2.87224900	2.83261600	-0.91669300
H	-3.23441800	2.23788200	0.70971700
H	-2.00110800	3.47374600	0.47468700
C	5.98226500	-1.26844300	0.20077500
H	6.44357100	-0.90596900	1.12108200
H	6.05137800	-2.36093300	0.20718700
H	6.56808000	-0.90561600	-0.64481100

Cation **49**

C	-3.57189400	-0.29728000	-0.01566800
C	-2.81462300	-0.25705700	-1.18588800
C	-1.45156200	0.00638800	-1.11758000
C	-0.86931000	0.22715300	0.12633900
C	-1.61522900	0.19368700	1.29955600
C	-2.97785700	-0.07712800	1.22512600
H	-3.28664600	-0.42979200	-2.14405500
H	-0.84851700	0.03137800	-2.01644500
H	-1.14202900	0.38752200	2.25419100
H	-3.57608800	-0.10312600	2.12623200
N	0.54192900	0.51052800	0.20061600
C	1.16285300	1.70270400	-0.18206700

C	1.46455200	-0.33116300	0.68439200
C	2.49843600	1.54552200	0.08590300
H	1.27071200	-1.32704400	1.04480500
C	3.93250800	-0.36989300	0.99423300
C	4.61815000	-1.02184900	-0.20579300
H	3.71375900	-1.10552500	1.76972100
H	4.56174400	0.39910900	1.44299900
C	5.95717800	-1.65569200	0.18354100
H	3.95259800	-1.78302300	-0.62758400
H	4.77656800	-0.27436600	-0.98976900
C	6.65143600	-2.32309400	-1.00463100
H	6.61152900	-0.88652200	0.60890000
H	5.79222200	-2.39385700	0.97640700
H	7.60192400	-2.76614600	-0.70215000
H	6.03292600	-3.11917000	-1.42816700
H	6.85880500	-1.60050500	-1.79880100
N	2.65313300	0.26896600	0.62898800
C	0.39796200	2.85516700	-0.72976000
H	0.01194200	2.64453600	-1.72995400
H	-0.45234700	3.09986500	-0.08920500
H	1.03872600	3.73323300	-0.79399500
C	3.63861800	2.48585100	-0.09009600
H	4.11152900	2.72400500	0.86643000
H	4.40285600	2.07413500	-0.75327100
H	3.28712800	3.41872600	-0.52792200
C	-5.04254100	-0.63788000	-0.09185900
F	-5.73208100	-0.09309800	0.92717300
F	-5.22663300	-1.97497900	-0.03553500
F	-5.59204500	-0.20442500	-1.24164800

S9 References

1. Kuriyama, M.; Matsuo, S.; Shinozawa, M.; Onomura, O. *Org. Lett*. **2013**, *15*, 2716–2719. doi:10.1021/ol4010189

2. Szpunar, M.; Loska, R. *Eur. J. Org. Chem*. **2015**, *10*, 2133–2137. doi:10.1002/ejoc.201500072

3. Mityanov, V. S.; Perevalov, V. P.; Tkach, I. I. *Chem. Heterocycl. Comp.* **2013,** *48*, 1793–1800. doi:10.1007/s10593-013-1210-8

4. Kuriyama, M.; Kujirada, S.; Tsukuda, K.; Onomura, O. *Adv. Synth. Catal.* **2017**, *359*, 1043–1048. doi:10.1002/adsc.201601105
